# Arylazobenzimidazoles: versatile visible-light photoswitches with tuneable *Z*-isomer stability†

**DOI:** 10.1039/d3sc05246j

**Published:** 2024-03-05

**Authors:** Sophie A. M. Steinmüller, Magdalena Odaybat, Giulia Galli, Davia Prischich, Matthew J. Fuchter, Michael Decker

**Affiliations:** a Pharmazeutische und Medizinische Chemie, Institut für Pharmazie und Lebensmittelchemie, Julius-Maximilians-Universität Würzburg Am Hubland 97074 Würzburg Germany michael.decker@uni-wuerzburg.de; b Department of Chemistry, Molecular Sciences Research Hub, White City Campus, Imperial College London London W12 0BZ UK m.fuchter@imperial.ac.uk

## Abstract

Benzimidazole heterocycles are of great importance in medicinal chemistry due to their applicability to a wide range of pharmacological targets, therefore representing a prototypical “privileged structure”. In photopharmacology, azoheteroarene photoswitches have emerged as valuable tools for a variety of applications due to the high tuneability of their photophysical properties. Benzimidazole-based photoswitches could therefore enable the optically-controlled investigation of many pharmacological targets and find application in materials science. Here we report a combined experimental and computational investigation of such arylazobenzimidazoles, which allowed us to identify derivatives with near-quantitative bidirectional photoswitching using visible light and highly tuneable *Z*-isomer stability. We further demonstrate that arylazobenzimidazoles bearing a free benzimidazole N–H group not only exhibit efficient bidirectional photoswitching, but also excellent thermal *Z*-isomer stability, contrary to previously reported fast-relaxing *Z*-isomers of N–H azoheteroarenes. Finally, we describe derivatives which can be reversibly isomerized with cyan and red light, thereby enabling significantly “red-shifted” photocontrol over prior azoheteroarenes. The understanding gained in this study should enable future photopharmacological efforts by employing photoswitches based on the privileged benzimidazole structure.

## Abstract 2

Arylazobenzimidazoles are photoswitchable privileged structures in medicinal chemistry. Experimental and theoretical studies identified compounds with visible light near-quantitative bidirectional photoswitching and highly tunable *Z*-isomer stability.

## Graphical abstract

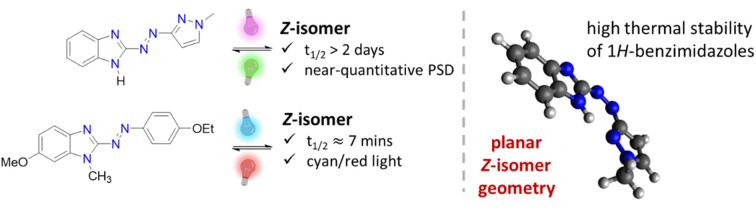

## Introduction

Photoswitchable molecules have undisputedly become vital tools for controlling molecular properties and functions in a large area of applications with unprecedented spatiotemporal resolution.^1–4^ Such photoswitches can be reversibly isomerized between a thermodynamically stable isomer (generally the *trans* or *E*-isomer for azoarenes) and a metastable isomer (usually the *cis* or *Z*-isomer for azoarenes) by irradiation with light of suitable wavelengths. In general, two characteristics of photoswitch performance stand out as being key to applications: (1) the completeness of isomerization at a given wavelength of light – high photostationary distribution (PSD) in the respective photostationary state (PSS), and (2) the thermal stability of the metastable photoisomer.^5–7^ The desired thermal isomerization half-life is dependent on the application of interest. For example, a short *Z*-isomer half-life can be desirable for some applications^8^ and unfavorable for others.^9^ For many biological events, a *Z*–*E* isomerization *t*_1/2_ from seconds to hours complies with the timescale of these processes.^10,11^ In materials science, longer thermal *Z*-isomer stability might be beneficial, and is ultimately required for energy or data storage.^12,13^ Azobenzenes are still the most prominent scaffold of photoswitches due to their comparatively uncomplicated synthesis and useful photoswitching properties, such as high quantum yields and fatigue resistance.^6,14,15^ While modulation of photophysical properties has been thoroughly investigated for the azobenzene scaffold, heteroarene azo photoswitches have only emerged over the last decade due to their high tuneability and unique properties.^5,16,17^ So far a variety of heterocycles have been explored including azopyrroles,^18,19^ azopyrazoles,^19–22^ azothiophenes,^23^ azothiazoles,^24,25^ azoimidazoles^26–28^ and azoindoles,^13,29^ as well as azoindazole,^30^ azopyrimidine^8^ and azopyridine.^31,32^ Such systems have been used to tune *Z*-isomer thermal half-lives from fast-relaxing systems (ns range)^8,13^ to highly stable *Z*-isomers with lifetimes *τ* > 46 years,^22^ or to optimize PSDs for both photoisomers.^19^ As almost 60% of FDA-approved drug molecules contain nitrogen heterocyclic structures,^33^ such azoheteroarenes are particularly interesting in photopharmacology to design photoswitchable derivatives of these candidates and obtain optical control over diverse biological targets.^3,5,34^

Benzimidazoles are known to be a “privileged structure” due to their wide spectrum of biological activities dependent on different substitution patterns at the core structure.^35,36^ Many pharmacological targets can be addressed with benzimidazole-derived structures, ranging from G protein-coupled receptors^37–41^ to enzymes,^42,43^ with such compounds exhibiting antiviral^44^ or anticancer^45^ activity. Moreover, several marketed drugs contain a benzimidazole nucleus.^36,46^ Although the core structure of 2-arylazobenzimidazoles and their salts were first described in 1970,^47–49^ surprisingly, their application in photopharmacology has only recently been described by us.^41^ We developed the first photoswitchable arylazobenzimidazole as β-arrestin2 pathway-biased cannabinoid 2 receptor ligands.^41^

Herein, we could show, that applying this interesting photoswitchable arylazobenzimidazole unit containing a “privileged structure” in medicinal chemistry offers unique opportunities to access photoswitchable biologically active molecules with visible-light irradiation.^41^

**Fig. 1:**
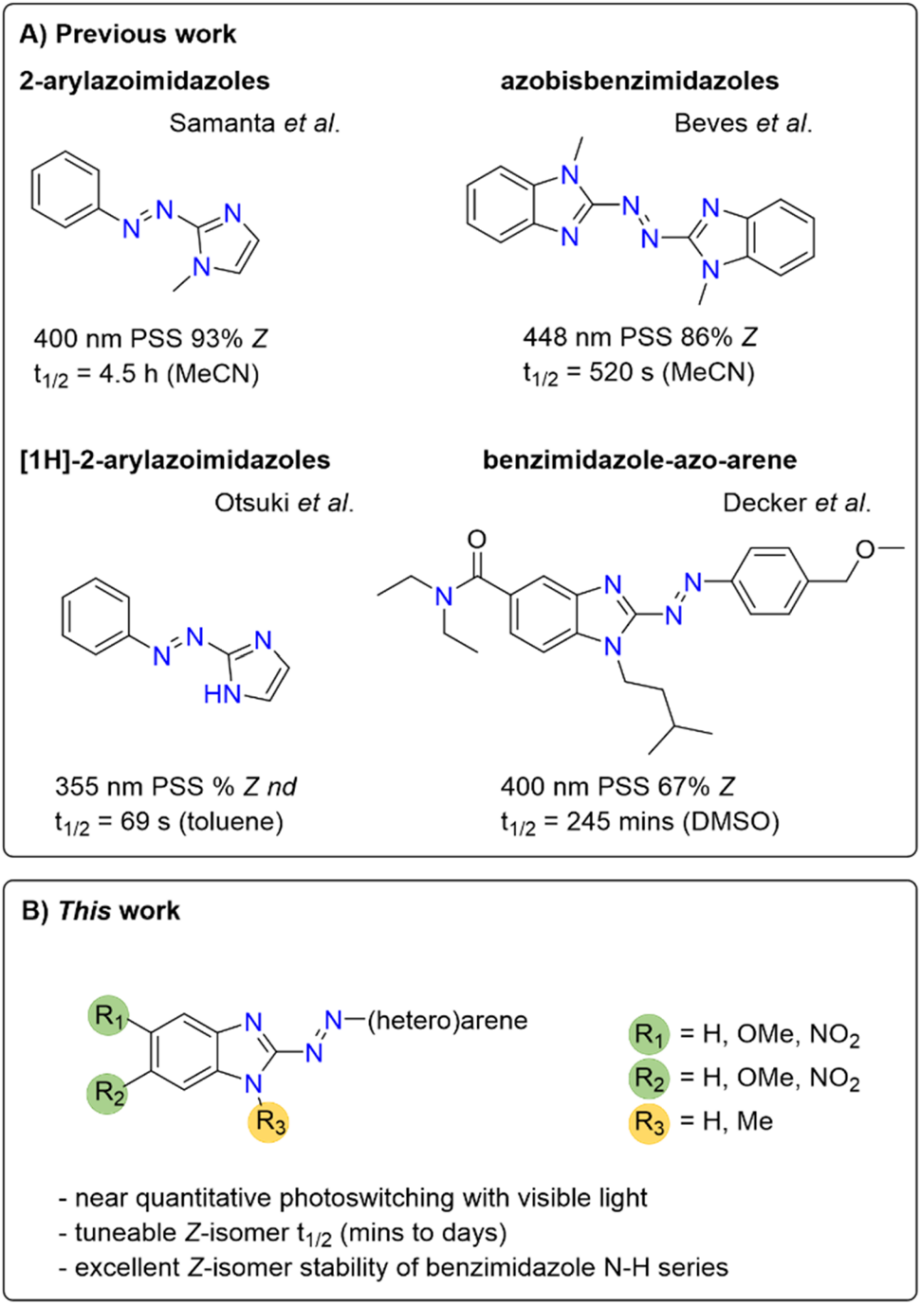
Previously reported imidazole and benzimidazole-containing photoswitchable molecules^50^ and the arylazobenzimidazoles synthesized in this study. nd = not determined.

Separately, Beves and co-workers reported azobisbenzimidazoles as visible-light photoswitches, which further highlights the potential usefulness of benzimidazole in azo photoswitches (Fig. 1).^50^

In the present work, we sought to develop a solid understanding of the photoswitching performance of this highly promising scaffold, laying the foundation for future studies in photopharmacology as well as for application across other disciplines, *e.g.* in smart materials. We aimed to specifically address the limited PSD observed for our cannabinoid 2 receptor ligands.^41^ Herein we report a combined experimental and computational study on arylazobenzimidazoles with highly tuneable isomerization properties, thermal half-lives and PSDs. We identified analogues with near-quantitative bidirectional photoswitching using visible light and discovered the excellent *Z*-isomer stability of the benzimidazole scaffold with a free N–H functionality. Thereby, we provide a broader and general understanding of this novel class of heteroarene photoswitches to pave the way to their rational application in future developments in life and material sciences.

## Results and discussion

### Synthesis

**Scheme 1:**
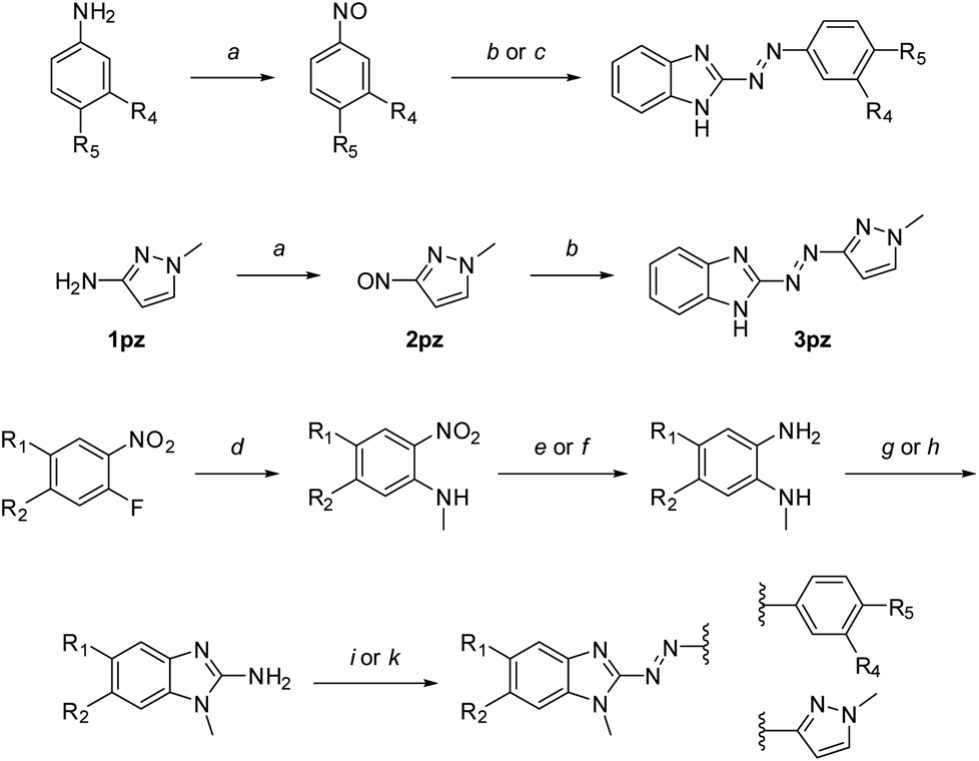
Synthesis of arylazobenzimidazoles.^*a*^ Synthesized derivatives are specified in Table 1. Reagents and conditions: (a) oxone®, water, CH_2_Cl_2_, rt, 1–12 h; (b) 1*H*-benzo[*d*]imidazole-2-amine or 5-methoxy-1*H*-benzo[*d*]imidazole-2-amine, toluene/40% NaOH, aq. (4 mL mmol^−1^), 80–85 °C, 2–6 h; (c) 2-amino-benzimidazole, toluene/DMSO (2 mL mmol^−1^), 1 mL mmol^−1^ 40% NaOH, aq., 65 °C, 30–60 min. (d) CH_3_NH_2_, NEt_3_, EtOH, rt or 55 °C, overnight; (e) H_2_, Pd/C, THF, rt, overnight; (f) Na_2_S·H_2_O, NaHCO_3_, MeOH, reflux, 1 h; (g) BrCN, CH_2_Cl_2_, RT, overnight; (h) BrCN, MeCN/H_2_O, 55 °C, 3 h, then RT, overnight; (i) 2a–f or 2pz, toluene/40% NaOH, aq. (4 mL mmol^−1^), 80–85 °C, 2–6 h; (k) 2a–d or 2pz, toluene/DMSO (2 mL mmol^−1^), 1 mL mmol^−1^ 40% NaOH, aq., 65 °C, 30–60 min.

We synthesized 28 arylazobenzimidazole derivatives from diverse 2-aminobenzimidazoles and a variety of nitroso compounds in a modified Baeyer–Mills reaction, previously shown as a versatile method for synthesizing azoheteroarenes (Scheme 1, Table 1).^18,41^

**Table 1:** Synthesized-arylazobenzimidazoles

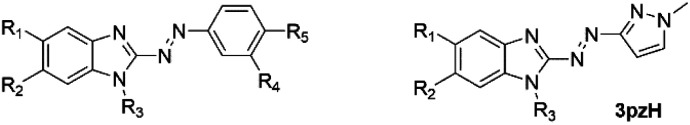					
Compound	R_1_	R_2_	R_3_	R_4_	R_5_
3a	H	H	H	H	H
3b	H	H	H	H	OEt
3c	H	H	H	OEt	H
3d	H	H	H	H	Cl
3pz	H	H	H	3pzH	
8a	H	H	CH_3_	H	H
8b	H	H	CH_3_	H	OEt
8d	H	H	CH_3_	H	Cl
8e	H	H	CH_3_	H	NO_2_
8pz	H	H	CH_3_	3pzH	
13aH	OMe	H	H	H	H
13a	OMe	H	CH_3_	H	H
13b	OMe	H	CH_3_	H	OEt
13c	OMe	H	CH_3_	OEt	H
13d	OMe	H	CH_3_	H	Cl
13e	OMe	H	CH_3_	H	NO_2_
13f	OMe	H	CH_3_	NO_2_	H
13pz	OMe	H	CH_3_	3pzH	
18a	H	OMe	CH_3_	H	H
18b	H	OMe	CH_3_	H	OEt
18d	H	OMe	CH_3_	H	Cl
18pz	H	OMe	CH_3_	3pzH	
23a	NO_2_	H	CH_3_	H	H
23b	NO_2_	H	CH_3_	H	OEt
23c	NO_2_	H	CH_3_	OEt	H
23d	NO_2_	H	CH_3_	H	Cl
26a	H	NO_2_	CH_3_	H	H
26b	H	NO_2_	CH_3_	H	OEt

Unsubstituted NH-benzimidazole derivatives were synthesized in one step using the respective nitrosobenzene derivatives or 1-methyl-3-nitroso-1*H*-pyrazole (“pz” compounds) and commercially available 2-aminobenzimidazoles. For *N*-methyl benzimidazole-azo-arenes, the synthesis started from commercially available 1-fluoro-2-nitrobenzenes, which were reacted with methylamine in a nucleophilic aromatic substitution reaction. Reduction of the nitro-group was achieved with hydrogen over Pd/C. For compounds carrying more than one nitro-substituent (to yield 5-nitrobenzimidazole derivatives), selective reduction was carried out as previously described.^51^ Ring formation of the 2-aminobenzimidazole precursor was achieved with cyanogen bromide using either a previously described method in methylene chloride,^41^ or using a mixture of acetonitrile/water for obtaining substituted benzimidazoles. The final Baeyer–Mills reaction with different nitrosobenzene derivatives was either carried out as previously described (in toluene/40% NaOH, aq. at 80 °C)^41^ or in an adapted procedure using a solvent mixture additionally containing DMSO to increase solubility.

Importantly, reactions using DMSO were closely monitored, as reaction times over 1 h led to the formation of many side-products, thereby hampering purification, which was especially observed for 6-nitrobenzimidazole derivatives. Yields for the basic Baeyer–Mills reactions were dependent on the substitution patterns, as for example *p*-ethoxy nitrosobenzene or nitro-substituents on either building block resulted in generally lower yields. The respective preferred method is described in the ESI.†

### UV/vis characterization and photophysical properties

The synthesized compounds were analyzed by UV/vis spectroscopy at 30 μM in either DMSO or tris(hydroxymethyl)aminomethane (TRIS)-buffer (pH = 7.4; containing 25% DMSO for solubility). Light of different wavelengths between 365 nm and 617 nm was used to determine the ideal switching wavelengths and to confirm reversible isomerization.

**Fig. 2:**
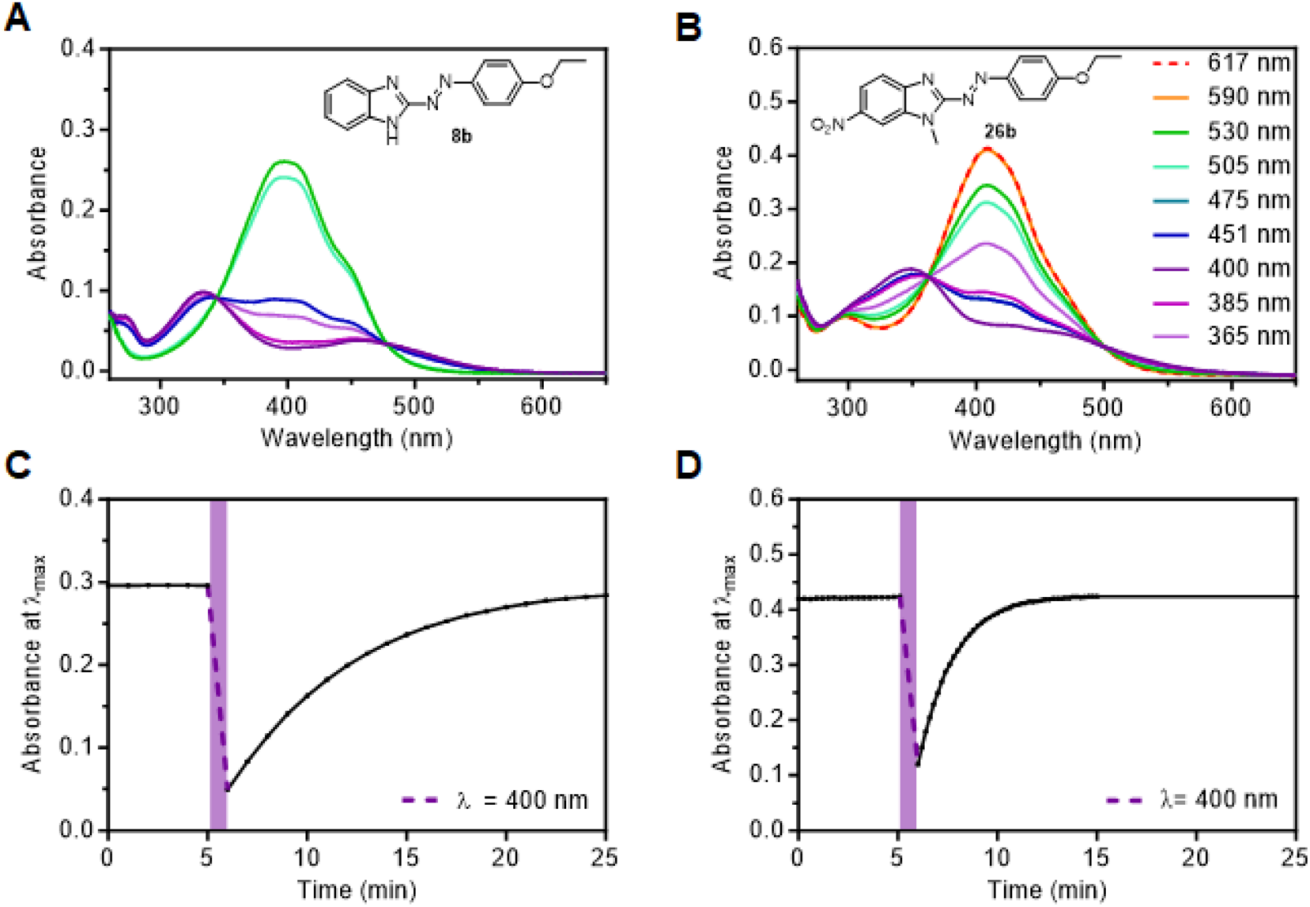
UV/vis spectra of arylazobenzimidazoles 8b (A) and 26b (B) after irradiation with a variety of different wavelengths as shown in the legend (only wavelengths that reached a consistent change in absorption after irradiation <1 min are included). Thermal stability measurement of 30 μM compound after switching to the respective *Z*-isomer with 400 nm for (C) 8b (in 1 : 3 DMSO/TRIS-buffer (pH = 7.4)) and (D) 26b (in 1 : 1 TRIS-buffer (pH = 7.4)), both at 37 °C.

Spectra of representative compounds 8b and 26b are shown in Fig. 2 and a complete overview of photocharacterization is given in Fig. S1–S28 (ESI).† In general, *E*-isomers exhibited strong broad absorption bands spanning ∼360–500 nm region, which were assigned to a π → π* transition.^15,26^ The *E*-isomer absorption bands for arylazobenzimidazoles are red-shifted compared to *E*-azobenzene, which displays a large π → π* absorption band around 320 nm.^15^ Alkoxy-residues in 5- and 6-position of the benzimidazole induced a further red-shift of the *E*-isomer π → π* absorption band. For example, the *λ*_max_ of *E*-8a (383 nm) undergoes 32 nm red-shift upon addition of a methoxy group (*E*-18a); almost a 100 nm red-shift compared to *E*-azobenzene. This can be attributed to the strong positive mesomeric effect (+M) of the OMe group, as has been observed for azobenzene.^52,53^ Such an effect is particularly strong for conjugated OR groups (compound 18b) and only slightly attenuated for cross-conjugated systems (compound 13b).

Generally, substitution of the phenyl ring further contributed to red-shifting of the *E*-isomer π → π* absorption compared to unsubstituted derivatives, with *p*-nitro (8e and 13e), being the most effective single substitution, followed by *p*-ethoxy derivatives (8b, 13b, 18b, 23b). Notably, the combination of OR and NO_2_ groups gives rise to push–pull compounds which display a strong red-shift, which is consistent in azobenzene.^53,54^ For example, *E*-13e has the most red-shifted *λ*_max_ of the series, at 441 nm. Methylation of the benzimidazole nitrogen (for example, 8a, 8b and 8d*versus*3a, 3b and 3d) only caused a negligible shift (<5 nm), as expected. For 5-methoxy benzimidazoles (13a–f, 13pz) a large overlap of the *E*- and *Z*-isomer absorption bands was noticeable. Consistent with literature,^24,25^ pyrazole-substitution does not cause a red-shift of π → π* absorption, and arylazobenzimidazoles showed a similar absorption for the unsubstituted phenyl ring compared to 3pzH derivatives. Compound 18pz exhibits the most blue-shifted *E*-isomer π → π* absorption maximum at 366 nm. Assigning n → π* transitions to both *E* and *Z* arylazobenzimidazoles was not trivial due to such peaks being absent/weak or obscured by overlapping absorptions. The *Z*-isomer n → π* absorption band, for derivatives where this could be assigned, was also red-shifted compared to regular azobenzene (*e.g.* compound 8b and 23a).^15^ Overall, it seems substituent effects on azobenzene are largely transferrable onto the arylazobenzimidazole scaffold. Furthermore, TD-DFT calculations (PBE0-GD3BJ/6-31G(d,p)) on select representative arylazobenzimidazole derivatives were found to reproduce the key trends observed experimentally (see ESI†).

All compounds apart from 3pz could be isomerized to their respective *Z*-isomer by visible light (Table 2), thereby showing red-shifted behavior for *E* → *Z* switching compared to regular azobenzenes, for which the highest *Z*-conversion can only be achieved by irradiation with UV light (*λ* = 320–360 nm).^9^ For most compounds, violet light (*λ* = 400 nm) was used to obtain the largest *Z*-isomer PSD, while the highest *Z*-conversion for compounds 13d, 13e, 13f, 18a, 18b and 18d was achieved with cyan light (*λ* = 475 nm). Photoisomerization back to the *E*-photoisomers of arylazobenzimidazoles was achieved with either green (*λ* = 530 nm), orange (*λ* = 590 nm) or red light (*λ* = 617 nm), enabled by the long wavelength absorption tail of the respective *Z*-isomer.

**Table 2:** UV/vis data, PSDs and thermal relaxation half-life of the *Z*-isomer in different solvents

Compound	*E* isomer π–π*, *λ*_max_/nm	*Z* isomer π–π*, *λ*_max_/nm	*Z* isomer n–π*, *λ*_max_/nm	Max. achievable ratios		*t*_1/2_ (*Z* → *E*) [min]	
				PSS *Z*^a^ [%] (*λ*_irr_)	PSS *E*^b^ [%] (*λ*_irr_)	DMSO^c^	Buffer^d^
3a	380	348	462	82 ± 3 (400)	95 (530)	397	415
3b	397	333	455	92 ± 3 (400)	100 (530)	139	4.4
3c	387	346	nd	82 ± 3 (400)	94 (530)	124	96
3d	386	346	447	85 ± 3 (400)	96 (530)	449	164
3pz	381	nd	432	91 ± 3 (385)	95 (530)	∼2.3d^e^	409^f^
8a	383	349	463	84 ± 3 (400)	90 (530)	350	89
8b	400	338	469	92 ± 3 (400)	100 (617)	22	1.8
8d	391	351	463	83 ± 3 (400)	>99 (590)	246	38
8e	408	382	nd	49 ± 4 (400)	100 (617)	27	14
8pz	381	nd	440	91 ± 3 (400)	87 (530)	697^e^	321
13aH	415	388	nd	66 ± 4 (400)	100 (590)	6.1	0.2
13a	408	379	nd	69 ± 4 (400)	>99 (590)	94	12
13b	420	342	476	80 ± 4 (400)	100 (617)	25	1.5
13c	413	387	nd	63 ± 4 (400)	>99 (590)	76	10
13d	416	388	nd	61 ± 4 (475)	>99 (590)	59	7.8^f^
13e	441	nd	nd	29 ± 4 (475)	98 (617)	44	6.5^f^
13f	419	389	nd	41 ± 4 (475)	>99 (590)	99	7.5
13pz	404	370	451	80 ± 4 (400)	100 (590)	490^e^	41
18a	415	380	nd	69 ± 4 (475)	>99 (617)	33	4.2
18b	430	341	479	80 ± 4 (475)	100 (617)	7.0	0.8
18d	429	390	457	66 ± 4 (475)	>99 (617)	21	2.3
18pz	366	339	437	80 ± 4 (400)	>99 (590)	190	12
23a	377	308	461	82 ± 3 (400)	89 (530)	742	213
23b	402	334	481	87 ± 3 (400)	100 (617)	97	8.2
23c	381	307	467	77 ± 4 (400)	94 (530)	424	187^g^
23d	385	307	465	84 ± 3 (400)	99 (590)	189	61
26a	381	346	454	78 ± 4 (400)	97 (590)	115	65
26b	409	349	nd	84 ± 4 (400)	100 (617)	24	1.2

a *Z*-isomer PSDs were determined as previously described (*c.f.* ESI).^19^

b *E*-isomer PSDs were obtained *via* LC/MS measurements in MeOH; *λ*_irr_ = irradiation wavelength to achieve max. PSS [nm].

c Measured at 22 °C.

d Measured at 37 °C, buffer = TRIS-buffer (pH = 7.4, containing 25% DMSO for solubility).

e Half-life was extrapolated.

f 1 : 1 DMSO/TRIS-buffer (pH = 7.4).

g 3 : 1 DMSO/TRIS-buffer (pH = 7.4); nd = not determinable; d = days.

Arylazobenzimidazoles therefore show excellent visible-light photoswitching and surpass the recently reported phenylazothiazoles, 2-arylazoimidazoles and currently most red-shifted dialkylamino arylazopyrazoles in terms of visible-light photoswitching.^4,24,55,56^

No photo-fatigue was detected in ten cycles of repeated *E*/*Z*-isomerization. For two representative compounds, 3pz and 8a, irradiation with 385/400 nm was carried out continuously over 1 h to further analyze potential photo-degradation. No significant photo-degradation was observed (see Fig. S29–S31, ESI†). This demonstrates the high photo-stability of both *N*-methylated arylazobenzimidazoles as well as derivatives carrying a free benzimidazole N–H.

Some trends were apparent with respect to the effect of substitution on *Z* → *E* photoswitching (Table 2). No significant difference in the PSD was observed for free N–H *vs. N*-methyl derivatives (3b*vs.*8b) using violet light (*λ* = 400 nm), which resulted in near-quantitative bidirectional photoswitching (>92% *Z*-isomer in both cases). Nitro-substitution in the *meta*- and *para*-position of the phenyl ring resulted in less complete *Z*-isomer photoswitching due to the overlap of the *E*- and *Z*-isomer π → π* and n → π* absorption bands (compound 8e and 13e/f). The push–pull derivative 13e exhibited the lowest *Z*-isomer PSD of the study with 29% *Z*, due to an almost complete band overlap in both photoisomers. In contrast, benzimidazole nitro-substitution was well-tolerated (23a–d, 26a/b), especially in combination with electron-donating substituents on the benzene and despite the occurrence of push–pull effects. Irradiation of compound 26b (Fig. 1) with violet light achieved 84% *Z*-isomer and irradiation into the tail of the n → π* absorbance at 617 nm switched it back to ∼100% *E*-isomer. Overall, the compounds synthesized in this study show tuneable and significantly improved PSDs compared to our previously reported arylazobenzimidazole cannabinoid 2 receptor ligands.^41^

Quantum yield (*φ*) was determined for two representative compounds, 3pz (*φ*_EZ_ = 0.22 and *φ*_ZE_ = 0.18) and 8a (*φ*_EZ_ = 0.17 and *φ*_ZE_ = 0.15) at 365 nm. The values obtained are comparable to the previously reported 2-arylazoimidazoles^4^ and other azo switches.^24^

*Z*-isomer thermal relaxation kinetics were determined using UV/vis spectroscopy, with thermal half-lives in a measurable range from 15 s to 7 h. The data was measured in DMSO at room temperature (22 °C), but also in TRIS-buffer (pH = 7.4) at 37 °C. The latter conditions were included to allow assessment under conditions relevant to cell- or enzyme-based assays.

For arylazobenzimidazoles that are methylated on the benzimidazole nitrogen (Table 1, R_3_ = Me), a number of trends are apparent. When inspecting the *t*_1/2_ values in DMSO, it is clear that electron-rich derivatives (*i.e.*, those bearing OR substituents) have shorter *Z*-isomer half-lives than less electron-rich derivatives. For example, 8b bearing an OEt on the phenyl ring has a *t*_1/2_ value in DMSO (22 min) >15 times shorter than parent molecule 8a (350 min). Consistently, the most electron-rich derivative in the study (18b) has the second shortest half-life in DMSO (7 min) after 13aH (see below for further discussion of 13aH). Interestingly, some derivatives bearing a nitro group in the 5-position, especially 23a, have an increased *Z*-isomer half-life in DMSO (742 min) over the parent molecule 8a (350 min).

Previously, arylazoimidazoles have been proposed to isomerize *via* the inversion pathway,^26,29^ which seems to be largely consistent with our arylazobenzimidazoles, given the *t*_1/2_ values obtained. However, the comparison between the kinetics in DMSO and buffer is instructive. While a degree of acceleration would be expected for the *Z*–*E* isomerization of all compounds in buffer, since the measurements were carried out at 37 °C instead of 22 °C (DMSO), some compounds undergo a much larger acceleration. For compounds with such a large *Z*–*E* isomerization acceleration, this may suggest the contribution of a competing rotational pathway for *Z*–*E* isomerization, which has a more polar transition state and would in turn be stabilized in a more polar solvent (*i.e.*, buffer). Such an effect has previously been observed for push–pull azobenzenes in polar solvents.^13,29^ The most obvious examples are arylazobenzimidazoles with *para*-OR groups on the phenyl ring, which result in a 12–20 times decrease in *Z*–*E* half-life in buffer over DMSO.

Consistent with previous studies,^22^ replacing the phenyl ring of the arylazobenzimidazoles with a pyrazole ring gave derivatives that exhibited the longest *Z*-isomer thermal isomerization half-lives. For example, the half-life of compound 13pz in DMSO is >5 times longer (490 min) than the equivalent phenyl derivative (13a, 94 min). Benzimidazole-pyrazole hybrids 8pz, 13pz and 18pz could also be isomerized with visible light and exhibited excellent bidirectional switching. This result further highlights the beneficial photophysical properties previously reported for pyrazole-containing photoswitches.^19,22,57^

Perhaps the most important observation with respect to *Z*-isomer stability came from analysis of arylazobenzimidazoles with a free benzimidazole N–H (Table 1, R_3_ = H). Generally speaking, heteroazoarenes with free N–H functionality can be subject to very fast *Z*–*E* relaxation due to the presence of a tautomerization-based isomerization mechanism.^29^ For example, structurally related 2- and 3-phenylazo[1*H*]indole photoswitches exhibit a *Z*–*E* thermal half-life in the milli-to nanosecond range in 1 : 1 DMSO/water, with 11 ns and 188 μs, respectively.^13,29^ Furthermore, arylazoimidazoles bearing a free imidazole-NH generally showed a more than 400 times faster thermal *Z*–*E* isomerization (*t*_1/2_ = 69 s in toluene) than the relatively stable methylated form.^4,26,58^ In contrast, for 1*H*-arylazobenzimidazole 3a the *t*_1/2_ is ∼7 h in buffer and DMSO. This comparably long thermal half-life makes the tautomerization isomerization mechanism highly unlikely. Even in the presence of an electron-donating substituent (13aH), the *Z*–*E* half-life in DMSO is still 6 min, *i.e*., orders of magnitude longer than the N–H arylazoimidazoles and arylazoindoles.

Using the arylazoindoles as a case study, König and co-workers have previously computationally assessed the likelihood of a tautomerization-based isomerization mechanism for a range of N–H azo heterocycles.^29^ They showed that where aromaticity is lost upon formation of the hydrazone – the key intermediate in such an isomerization mechanism – the energetics of tautomerization are unfavorable, associated with the formation of quinoid-like structures. Consistently, they also showed that five-membered heterocycles possessing lower aromaticity are more prone to tautomerize.

**Fig. 3:**
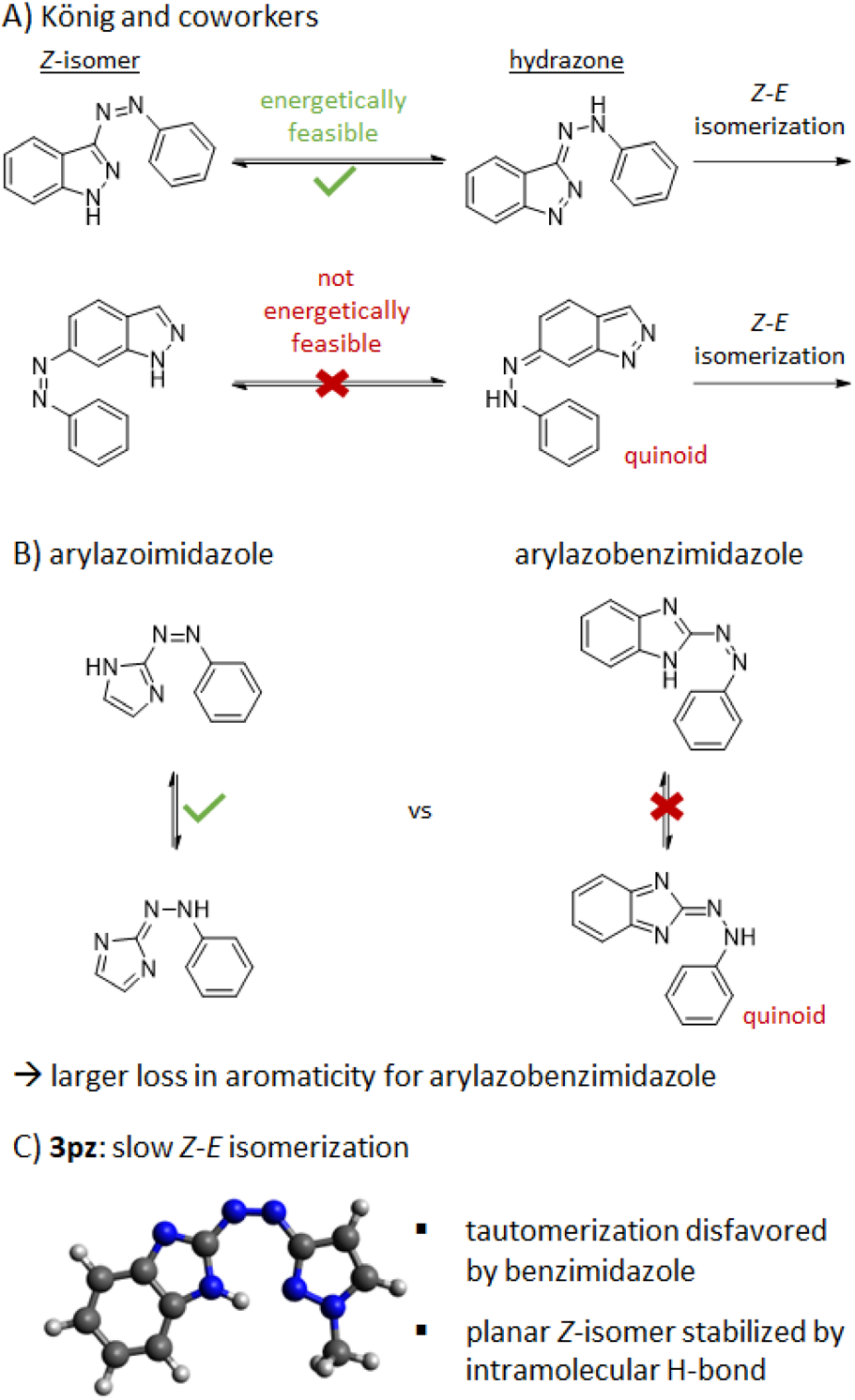
Feasibility of tautomerization-based isomerization mechanisms for selected azoheteroarenes.

These general rules appear to hold true when comparing N–H arylazoimidazoles^26,58^ (isomerization in s) to the N–H arylazobenzimidazoles (isomerization in min to hours) in this study. This is shown pictorially in Fig. 3. However, it would seem that additional factors are at play for benzimidazole-pyrazole hybrid 3pz, which has a *Z*-isomer half-life of 2.3 days in DMSO despite a free N–H group (*i.e.*, >4 orders of magnitude over N–H arylazoimidazoles).^26^ To understand the origin of this extraordinary difference, the computed ground state conformations of select *E* and *Z*-isomers from this study were assessed (PBE0-GD3BJ/6-31G(d,p)). Generally, speaking, the *E*-isomers were found to have a planar conformation whereas the *Z*-isomers were found to be twisted. One key exception was 3pz which showed a planar *Z*-isomer conformation due to an intramolecular hydrogen bond between the benzimidazole N–H and the pyrazole N. While it has previously been reported that (benz)imidazole heteroazoarenes can be protonated under acidic conditions and undergo intramolecular H-bonding,^27,50^ compound 3pz appears to be a case where such a mechanism is operative in the neutral form. The role of H-bonding in the stabilization of the *Z*-isomer is further supported by the *Z*–*E* rate acceleration in the more polar aqueous buffer (Table 2).

## Conclusions

In summary, we have demonstrated high tuneability of the arylazobenzimidazoles through the analysis of 28 synthesized derivatives. Through introduction of 5- and 6-methoxy-substituents at the benzimidazole-core, reversible photoswitching with cyan and red-light was enabled for compounds 13d–f, 18a, 18b and 18d. Furthermore, 1*H*-benzimidazoles and methylated derivatives azo-coupled to *p*-ethoxyphenyl (3b and 8b) or 3pzH (3pz and 8pz) enabled near-quantitative bidirectional isomerization with visible light. *Z*-isomer thermal relaxation half-life was found to be tuneable from several seconds to hours and even days, depending on substitution pattern and solvent system used. Importantly, we show that arylazobenzimidazoles bearing a free benzimidazole N–H show exceptional *Z*-isomer thermal stability in DMSO and aqueous TRIS-buffer solution (pH = 7.4), as well as efficient bidirectional visible-light induced photoisomerization. The reported data provides an overview of structure–property relationships for this novel photoswitchable scaffold and is anticipated to serve as a useful guide for application of azobenzimidazole photoswitches in photopharmacology and materials science.

## Abbreviations

ACN: Acetonitrile
DMSO: Dimethylsulfoxide
PSD: Photostationary distribution
PSS: Photostationary state
TD-DFT: Time-dependent density functional theory
TRIS: tris(hydroxymethyl)aminomethane

## Data availability

Computational data can be obtained at the following DOI: https://10.6084/m9.figshare.25374331. For raw data associated with the characterisation in the ESI,† please contact the corresponding authors.

## Author contributions

S. A. M. Steinmüller performed chemical synthesis and tested photophysical properties of the target compounds. M. Odaybat performed DFT and TD-DFT calculations. G. Galli assisted with photophysical characterization. D. Prischich determined quantum yields. M. J. Fuchter and M. Decker were responsible for the overall supervision of the project and funding acquisition. The manuscript was written through contributions of S. A. M. Steinmüller, M. Odaybat, M. J. Fuchter and M. Decker. All authors have given approval to the final version of the manuscript.

## Conflicts of interest

There are no conflicts to declare.

## Supplementary Material

SC-015-D3SC05246J-s001
