# Correction: Arylazobenzimidazoles: versatile visible-light photoswitches with tuneable *Z*-isomer stability

**DOI:** 10.1039/d5sc90173a

**Published:** 2025-08-06

**Authors:** Sophie A. M. Steinmüller, Magdalena Odaybat, Giulia Galli, Davia Prischich, Matthew J. Fuchter, Michael Decker

**Affiliations:** a Pharmazeutische und Medizinische Chemie, Institut für Pharmazie und Lebensmittelchemie, Julius-Maximilians-Universität Würzburg Am Hubland 97074 Würzburg Germany michael.decker@uni-wuerzburg.de; b Department of Chemistry, Molecular Sciences Research Hub, White City Campus, Imperial College London London W12 0BZ UK m.fuchter@imperial.ac.uk

## Abstract

Correction for ‘Arylazobenzimidazoles: versatile visible-light photoswitches with tuneable *Z*-isomer stability’ by Sophie A. M. Steinmüller *et al.*, *Chem. Sci.*, 2024, **15**, 5360–5367, https://doi.org/10.1039/D3SC05246J.

We recently became aware of inconsistent results obtained with two 475 nm lamps, that were used for excitation of the synthesized photoswitches in this study. This led us to directly measure the emission spectra of the lamps for validation. The measurements revealed that one of the lamps, although supplied to give maximal emission at 475 nm, in fact emitted at 440 nm due to incorrect supplier information (Fig. 1).

**Fig. 1:**
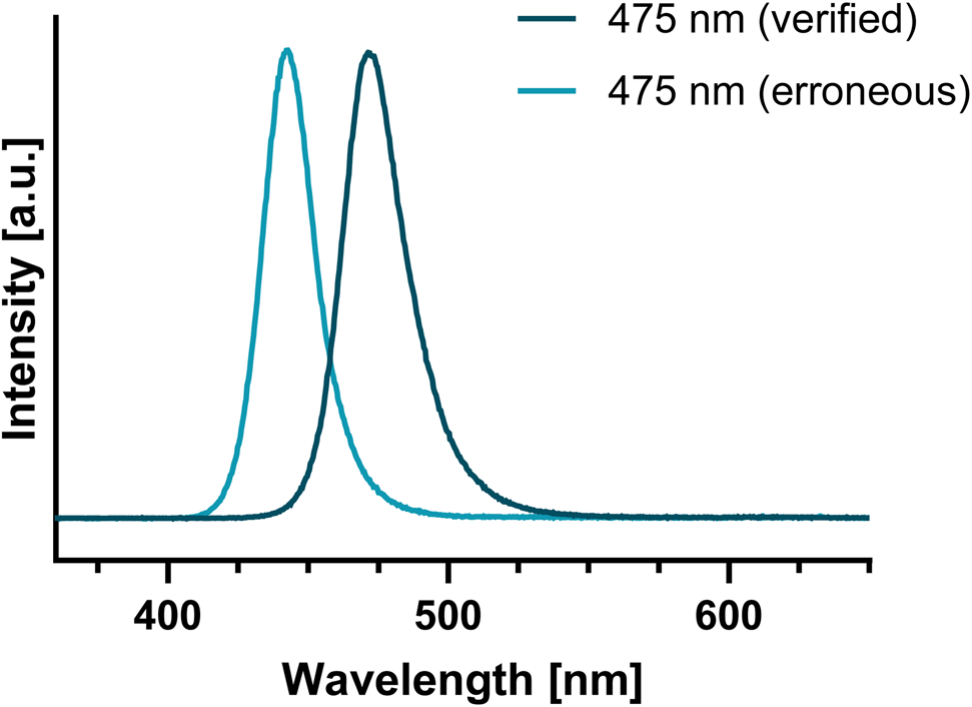
Emission spectra of the verified ‘Cree® XLamp® XP-E2’ *vs.* the erroneous ‘OSLON® SSL 80’ 475 nm LED module used for sample irradiation.

Following this, we recorded emission spectra of all other lamps employed within this publication and verified that no other wavelengths were affected. After reviewing all our experimental data, we repeated the measurements of the directly affected arylazobenzimidazoles, *i.e.*, those previously addressed using the ‘475 nm’ LED to achieve *Z*-isomer photoswitching (compounds **13d,e,f** and **18a,b,d**). For compounds where 475 nm light (using a verified lamp) still produced the highest *Z*-isomer conversion, we retained the original values and figures. For those that had shown highest conversion using the erroneous 475 nm LED, we have retained the reported *Z*/*E* ratio listed in Table 2 of the original publication (as it remains the best performance observed) but corrected the corresponding wavelength to 440 nm (according to the verified LED used) in the tables and figures, including those in the ESI. For the affected compounds (**13d** and **18a,b,d**), the emission of the 440 nm LED overlaps more effectively with the respective *E*-isomer's absorption band, leading to greater excitation and isomerization efficiency. Additionally, for these compounds, irradiation with 440 nm light minimizes excitation of the respective *Z*-isomer, thereby favouring a higher *Z*-population at the photostationary state. The overall findings and conclusions of our paper are not affected. To ensure all information provided in this publication is correct, we revise it as follows:

“For most compounds, violet light (*λ* = 400 nm) was used to obtain the largest *Z*-isomer PSD, while the highest *Z*-conversion for compounds **13d**, **18a**, **18b** and **18d** was achieved with blue light (*λ* = 440 nm). For compounds **13e** and **13f** the highest *Z*-conversion was achieved with cyan light (*λ* = 475 nm).”

**Table 2:** UV/Vis data, PSDs and thermal relaxation half-life of the *Z*-isomer in different solvents (corrections in bold)

Compound	*E* isomer π–π*, *λ*_max_/nm	*Z* isomer π–π*, *λ*_max_/nm	*Z* isomer n–π*, *λ*_max_/nm	Max. achievable ratios		*t*_1/2_ (*Z* → *E*) [min]	
				PSS *Z*^a^ [%] (*λ*_irr_)	PSS *E*^b^ [%] (*λ*_irr_)	DMSO^c^	Buffer^d^
**3a**	380	348	462	82 ± 3 (400)	95 (530)	397	415
**3b**	397	333	455	92 ± 3 (400)	100 (530)	139	4.4
**3c**	387	346	nd	82 ± 3 (400)	94 (530)	124	96
**3d**	386	346	447	85 ± 3 (400)	96 (530)	449	164
**3pz**	381	nd	432	91 ± 3 (385)	95 (530)	∼2.3d^e^	409^f^
**8a**	383	349	463	84 ± 3 (400)	90 (530)	350	89
**8b**	400	338	469	92 ± 3 (400)	100 (617)	22	1.8
**8d**	391	351	463	83 ± 3 (400)	>99 (590)	246	38
**8e**	408	382	nd	49 ± 4 (400)	100 (617)	27	14
**8pz**	381	nd	440	91 ± 3 (400)	87 (530)	697^e^	321
**13aH**	415	388	nd	66 ± 4 (400)	100 (590)	6.1	0.2
**13a**	408	379	nd	69 ± 4 (400)	>99 (590)	94	12
**13b**	420	342	476	80 ± 4 (400)	100 (617)	25	1.5
**13c**	413	387	nd	63 ± 4 (400)	>99 (590)	76	10
**13d**	416	388	nd	61 ± 4 (**440**)	>99 (590)	59	7.8^f^
**13e**	441	nd	nd	29 ± 4 (475)	98 (617)	44	6.5^f^
**13f**	419	389	nd	41 ± 4 (475)	>99 (590)	99	7.5
**13pz**	404	370	451	80 ± 4 (400)	100 (590)	490^e^	41
**18a**	415	380	nd	69 ± 4 (**440**)	>99 (617)	33	4.2
**18b**	430	341	479	80 ± 4 (**440**)	100 (617)	7.0	0.8
**18d**	429	390	457	66 ± 4 (**440**)	>99 (617)	21	2.3
**18pz**	366	339	437	80 ± 4 (400)	>99 (590)	190	12
**23a**	377	308	461	82 ± 3 (400)	89 (530)	742	213
**23b**	402	334	481	87 ± 3 (400)	100 (617)	97	8.2
**23c**	381	307	467	77 ± 4 (400)	94 (530)	424	187^g^
**23d**	385	307	465	84 ± 3 (400)	99 (590)	189	61
**26a**	381	346	454	78 ± 4 (400)	97 (590)	115	65
**26b**	409	349	nd	84 ± 4 (400)	100 (617)	24	1.2

a *Z*-Isomer PSDs were determined as previously described (*c.f.* ESI).^19^

b *E*-Isomer PSDs were obtained *via* LC/MS measurements in MeOH; *λ*_irr_ = irradiation wavelength to achieve max. PSS [nm].

c Measured at 22 °C.

d Measured at 37 °C, buffer = Tris-buffer (pH = 7.4, containing 25% DMSO for solubility).

e Half-life was extrapolated.

f 1 : 1 DMSO/Tris-buffer (pH = 7.4).

g 3 : 1 DMSO/Tris-buffer (pH = 7.4); nd = not determinable; d = days

## Conclusion

The adapted conclusion therefore reads: “Through introduction of 5- and 6-methoxy-substituents at the benzimidazole-core, reversible photoswitching with blue or cyan and red-light was enabled for compounds **13d–f**, **18a,b** and **18d**.”

This erratum also provides an updated ESI for the original paper, where the wavelengths in the spectra have been corrected according to the actual lamp used. Generally, the reported changes further refine the tuneability of the class of arylazobenzimidazole photoswitches in this work, which does by no means change the overall conclusions.

The Royal Society of Chemistry apologises for these errors and any consequent inconvenience to authors and readers.

