# Supplementary material for: Arylazobenzimidazoles: versatile visible-light photoswitches with tuneable Z-isomer stability
Source: Chem Sci. 2024 Mar 5;15(14):5360–7. doi: 10.1039/d3sc05246j (PMC10988581; doi:10.1039/d3sc05246j)
Supplement: SC-015-D3SC05246J-s001 [file SC-015-D3SC05246J-s001.pdf]

Electronic Supplementary Material (ESI) for Chemical Science.
This journal is © The Royal Society of Chemistry 2024

Arylazobenzimidazoles: Versatile Visible-Light Photoswitches with Tuneable *Z*-isomer Stability

Sophie A. M. Steinmüller[a], Magdalena Odaybat[b], Giulia Galli[a], Matthew J. Fuchter[b,*] and Michael Decker[a,*] Davia Prischich

[a]Pharmazeutische und Medizinische Chemie, Institut für Pharmazie und Lebensmittelchemie, Julius-Maximilians-Universität Würzburg, Am Hubland, 97074 Würzburg, Germany

[b]Department of Chemistry, Molecular Sciences Research Hub, White City Campus, Imperial College London, London SW7 2AZW12 0BZ, United Kingdom.

Electronic Supplementary Information

Table of Contents

Photochemistry 2
General Methods 2
UV/Vis Characterization 2
E/*Z* Compositions of PSSs 17
LC/MS Data 17
UV/Vis Spectra 31
Long-Term Stability 41
Quantum Yield Measurements 43
Synthesis 47
General Methods 47
Experimental 48
NMR Spectra 61
Computational 89
General Methods 89
Optimised Geometries 89
Time-Dependent DFT 118
References 122

Photochemistry

General Methods

UV/Vis experiments were performed with a Varian Cary 50 Bio UV/Vis spectrophotometer using Hellma (Type 100-QS) quartz glass Suprasil cuvettes (10 mm light path). Absorption spectra were measured at room temperature and analyzed using CaryWinUV software. Samples were irradiated using LEDs of Seoulviosys, Cree, Lumileds and LedEngin emitting the monochromatic wavelengths (365 nm, 385 nm, 400 nm, 450 nm, 475 nm, 505 nm, 530 nm, 590 nm and 617 nm). Only spectra where irradiation of the solutions with the respective wavelength for 90 seconds yielded maximal conversion to the corresponding equilibrium are shown. Wavelengths not allowing for full conversion in the given time frame were excluded. Thermal relaxation was measured by switching the photochromic compounds into the thermodynamically meta-stable *Z*-isomer using either 400 nm (violet) or 475 nm (cyan) light. Afterwards, *Z*-isomer thermal relaxation was determined by monitoring the change of absorption at the respective λ_{max} at room temperature (22°C) in DMSO and at 37°C in TRIS-buffer (pH = 7.4), containing 25% DMSO for solubility. Half-lives were analyzed through nonlinear regression (curve fit)-plateau followed by one phase decay with Graphpad Prism 9. If a compound did not thermally isomerize back within 15 h, LEDs were used to switch it back to the *E*-isomer and the half-life was extrapolated. Absence of photo-fatigue was analyzed either using a Varian Cary 50 Bio UV/Vis spectrophotometer and Hellma (Type 100-QS) quartz glass Suprasil cuvettes (10 mm light path), or by measuring several compounds requiring similar irradiation wavelengths simultaneously using a 96-well plate and a Spectramax 250 absorbance microplate reader (molecular devices). Compounds were irradiated alternatingly with the indicated wavelengths and absorption of the solutions was checked to be constant for each PSS.

UV/Vis Characterization

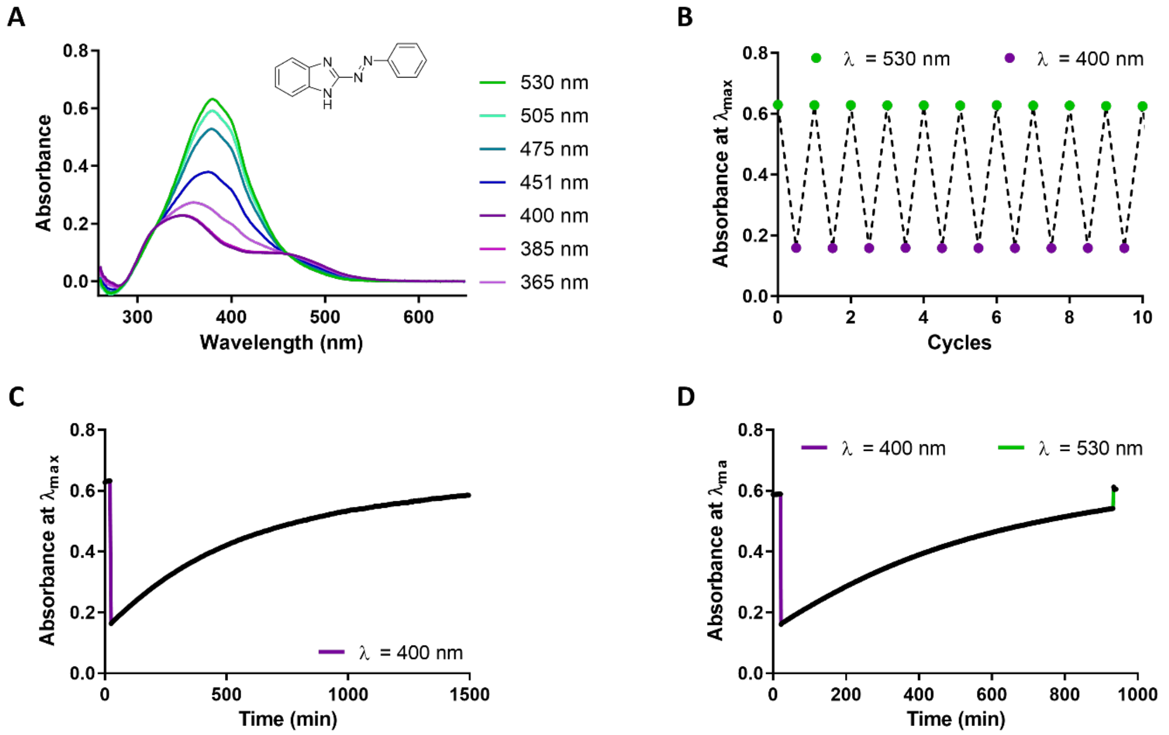

*Figure S 1. Photophysical properties of compound **3a**. Absorption spectra: (A) after irradiation with different wavelengths of 30 µM compound in DMSO; (B) repeated E/Z-isomerization by alternating irradiation with 400 nm and 530 nm for 1 min in DMSO; (C) stability of Z-**3a** in DMSO in the dark after switching with 400 nm, measured at 22°C; (D) stability of Z-**3a** in TRIS-buffer (containing 25 % DMSO, pH= 7.4) in the dark after switching with 400 nm, measured at 37°C.*

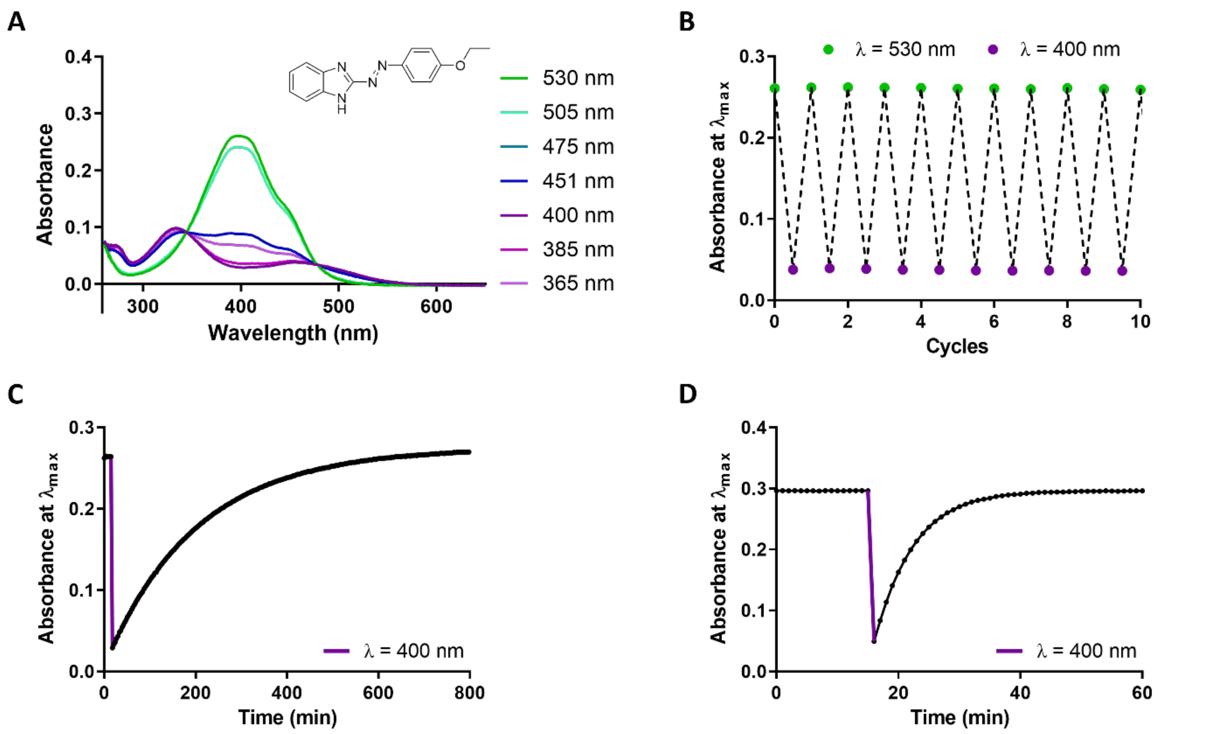

*Figure S 2. Photophysical properties of compound **3b**. Absorption spectra: (A) after irradiation with different wavelengths of 30 µM compound in DMSO; (B) repeated E/Z-isomerization by alternating irradiation with 400 nm and 530 nm for 1 min in DMSO; (C) stability of Z-**3b** in DMSO in the dark after switching with 400 nm, measured at 22°C; (D) stability of Z-**3b** in TRIS-buffer (containing 25 % DMSO, pH= 7.4) in the dark after switching with 400 nm, measured at 37°C.*

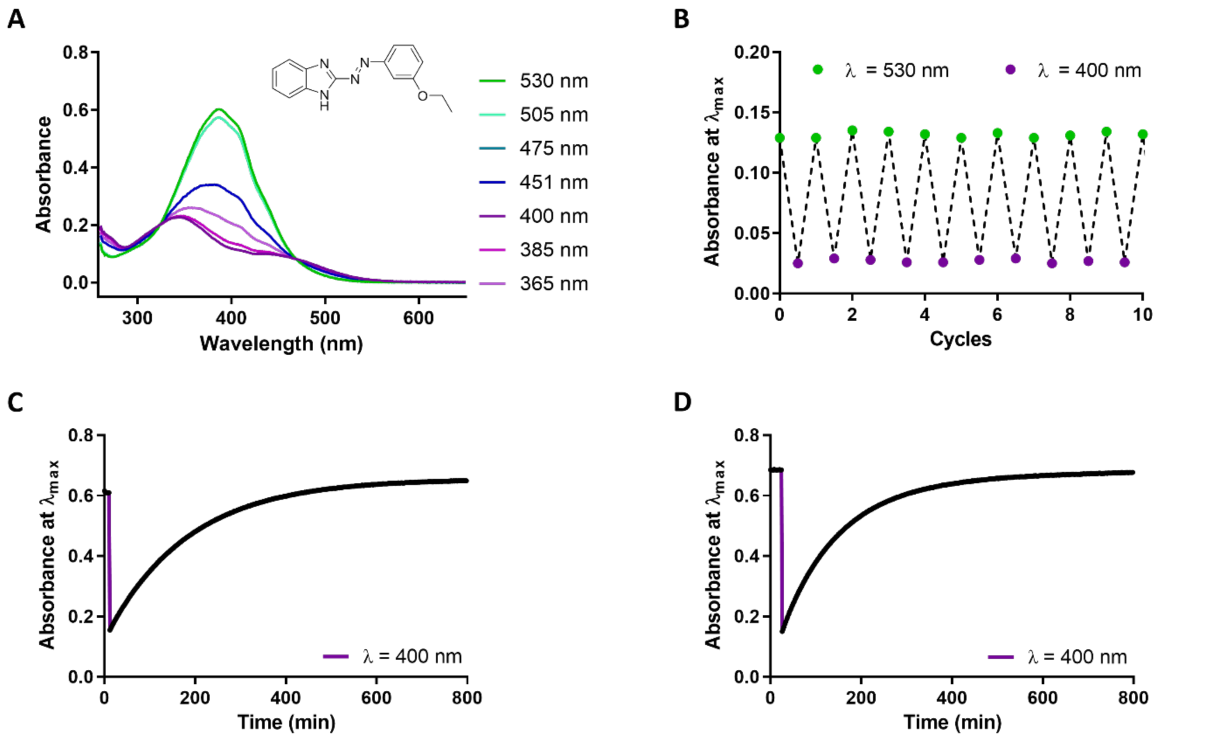

*Figure S 3. Photophysical properties of compound **3c**. Absorption spectra: (A) after irradiation with different wavelengths of 30 µM compound in DMSO; (B) repeated E/Z-isomerization by alternating irradiation with 400 nm and 530 nm for 1 min in DMSO; (C) stability of Z-**3c** in DMSO in the dark after switching with 400 nm, measured at 22°C; (D) stability of Z-**3c** in TRIS-buffer (containing 25 % DMSO, pH= 7.4) in the dark after switching with 400 nm, measured at 37°C.*

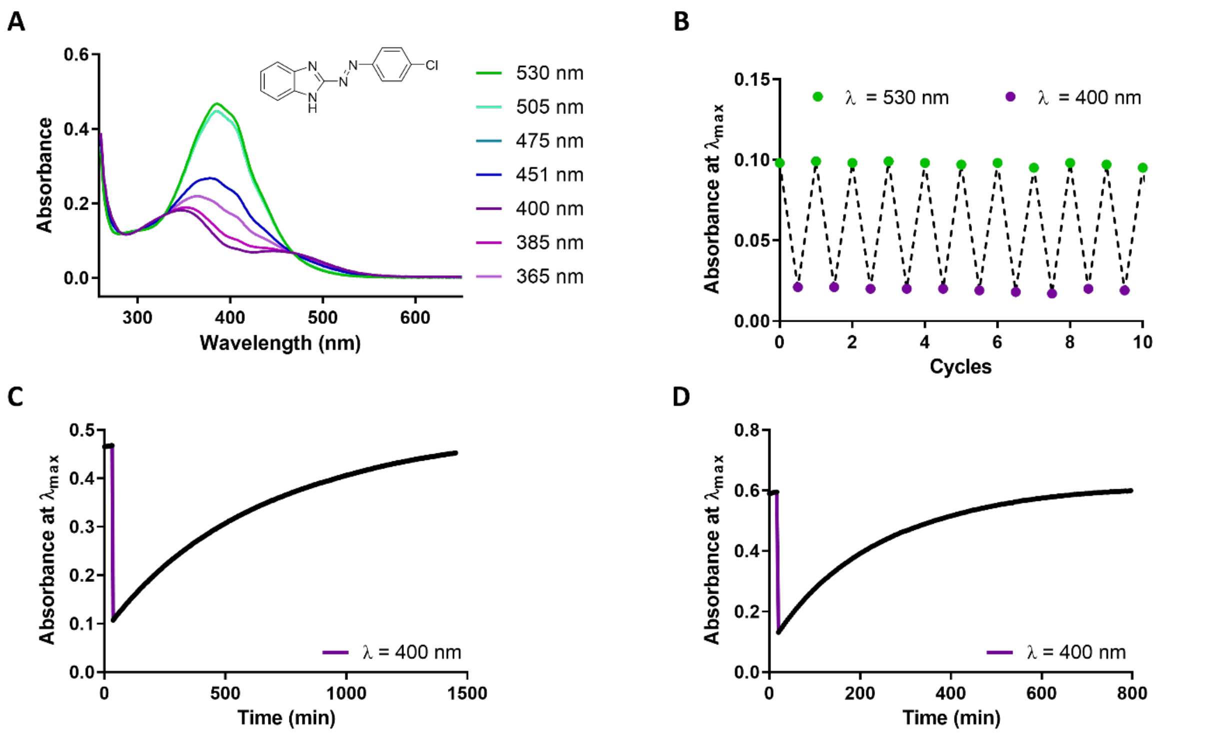

*Figure S 4. Photophysical properties of compound **3d**. Absorption spectra: (A) after irradiation with different wavelengths of 30 µM compound in DMSO; (B) repeated E/Z-isomerization by alternating irradiation with 400 nm and 530 nm for 1 min in DMSO; (C) stability of Z-**3d** in DMSO in the dark after switching with 400 nm, measured at 22°C; (D) stability of Z-**3d** in TRIS-buffer (containing 25 % DMSO, pH= 7.4) in the dark after switching with 400 nm, measured at 37°C.*

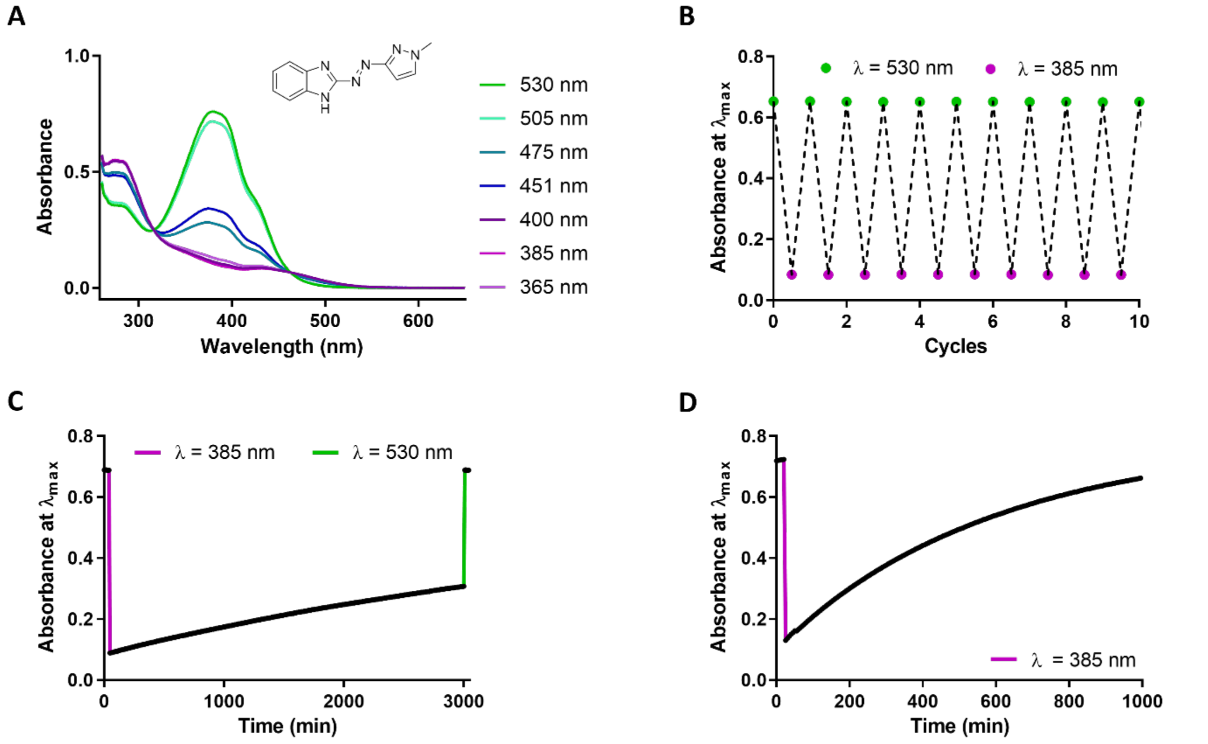

*Figure S 5. Photophysical properties of compound **3pz**. Absorption spectra: (A) after irradiation with different wavelengths of 30 µM compound in DMSO; (B) repeated E/Z-isomerization by alternating irradiation with 385 nm and 530 nm for 1 min in DMSO; (C) stability of Z-**3pz** in DMSO in the dark after switching with 385 nm, measured at 22°C; (D) stability of Z-**3pz** in TRIS-buffer (containing 50 % DMSO, pH= 7.4) in the dark after switching with 385 nm, measured at 37°C.*

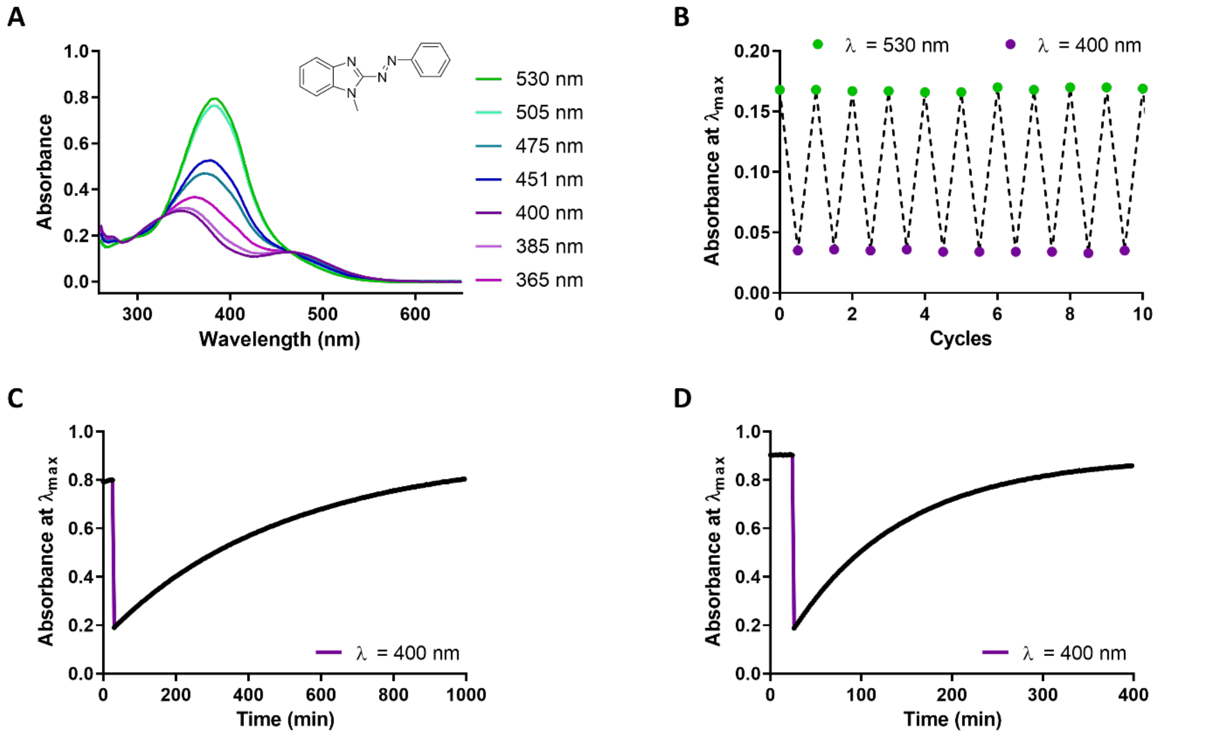

*Figure S 6. Photophysical properties of compound **8a**. Absorption spectra: (A) after irradiation with different wavelengths of 30 µM compound in DMSO; (B) repeated E/Z-isomerization by alternating irradiation with 400 nm and 530 nm for 1 min in DMSO; (C) stability of Z-**8a** in DMSO in the dark after switching with 400 nm, measured at 22°C; (D) stability of Z-**8a** in TRIS-buffer (containing 25 % DMSO, pH= 7.4) in the dark after switching with 400 nm, measured at 37°C.*

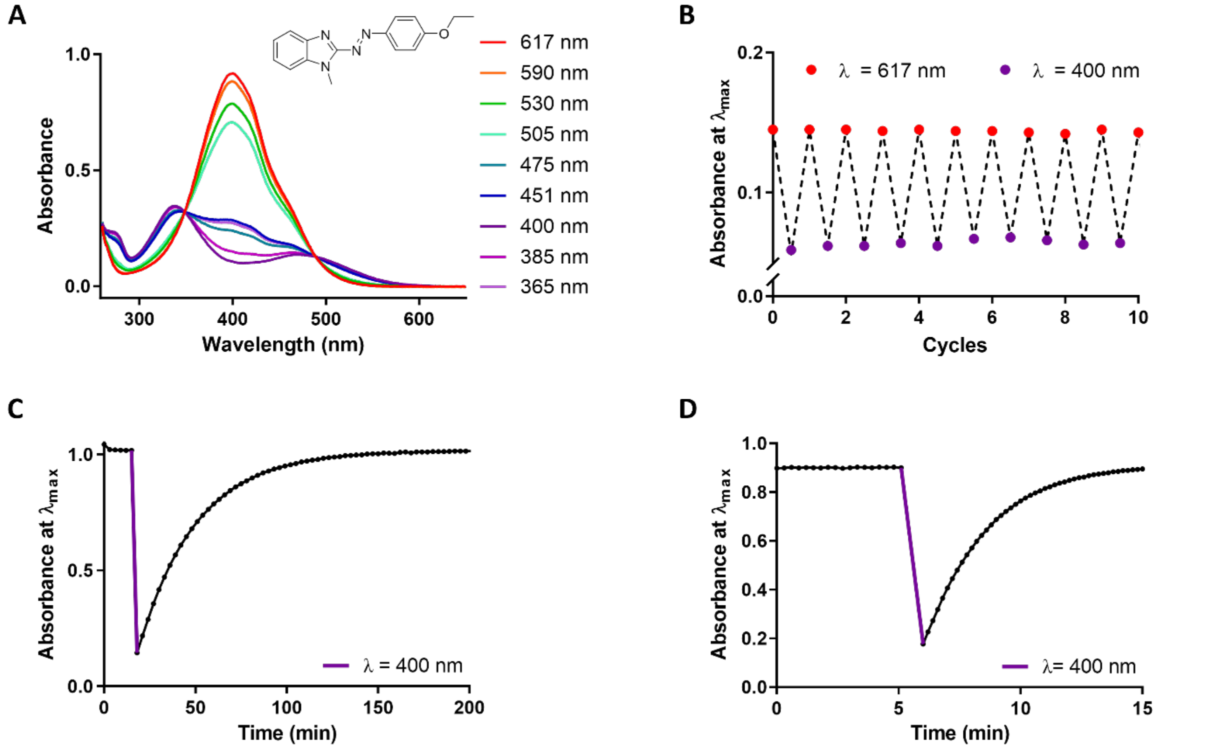

*Figure S 7. Photophysical properties of compound 8**b**. Absorption spectra: (A) after irradiation with different wavelengths of 30 µM compound in DMSO; (B) repeated E/Z-isomerization by alternating irradiation with 400 nm and 617 nm for 1 min in DMSO; (C) stability of Z-**8b** in DMSO in the dark after switching with 400 nm, measured at 22°C; (D) stability of Z-**8b** in TRIS-buffer (containing 25 % DMSO, pH= 7.4) in the dark after switching with 400 nm, measured at 37°C.*

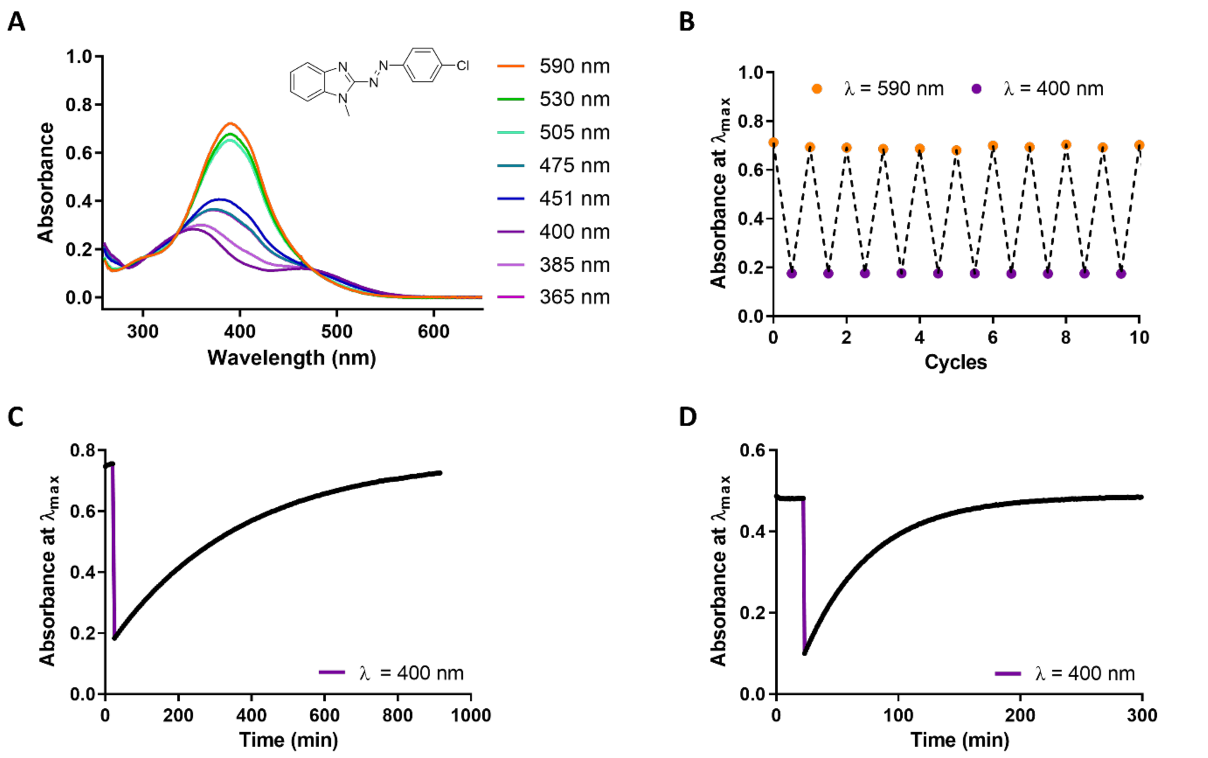

*Figure S 8. Photophysical properties of compound **8d**. Absorption spectra: (A) after irradiation with different wavelengths of 30 µM compound in DMSO; (B) repeated E/Z-isomerization by alternating irradiation with 400 nm and 590 nm for 1 min in DMSO; (C) stability of Z-**8d** in DMSO in the dark after switching with 400 nm, measured at 22°C; (D) stability of Z-**8d** in TRIS-buffer (containing 25 % DMSO, pH= 7.4) in the dark after switching with 400 nm, measured at 37°C.*

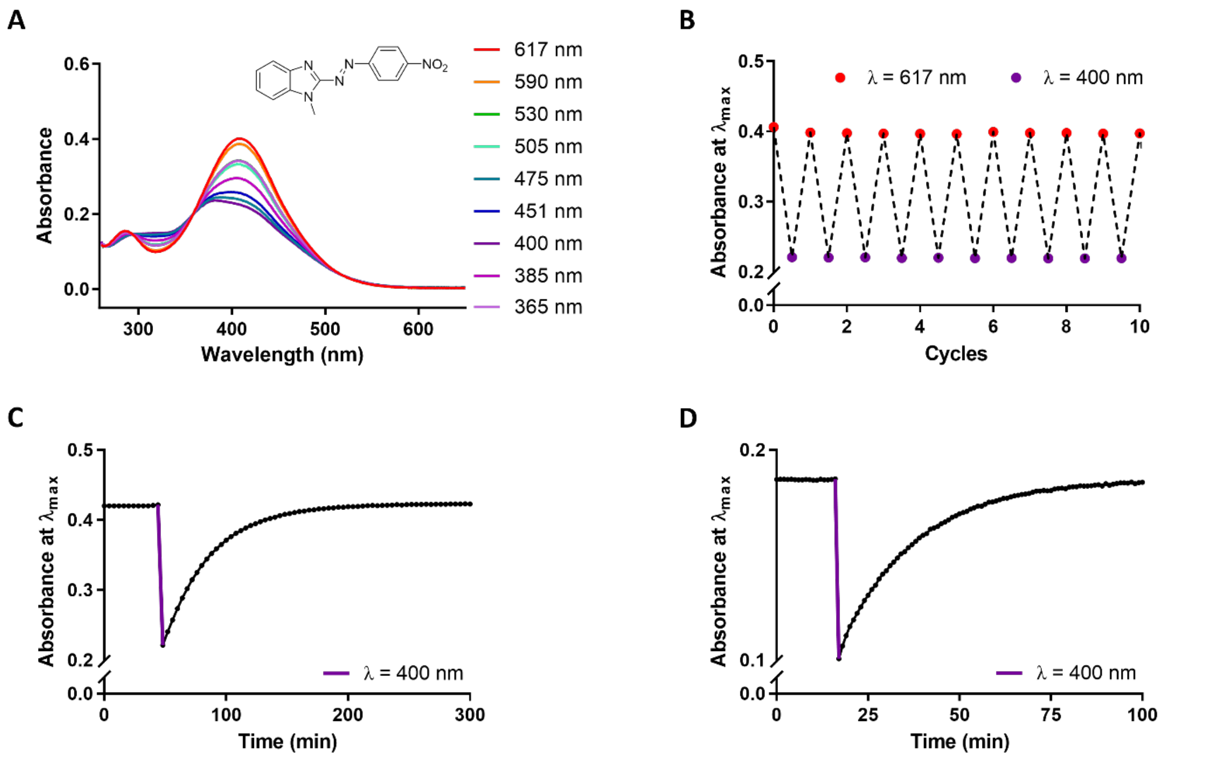

*Figure S 9. Photophysical properties of compound **8e**. Absorption spectra: (A) after irradiation with different wavelengths of 30 µM compound in DMSO; (B) repeated E/Z-isomerization by alternating irradiation with 400 nm and 617 nm for 1 min in DMSO; (C) stability of Z-**8e** in DMSO in the dark after switching with 400 nm, measured at 22°C; (D) stability of Z-**8e** in TRIS-buffer (containing 25 % DMSO, pH= 7.4) in the dark after switching with 400 nm, measured at 37°C.*

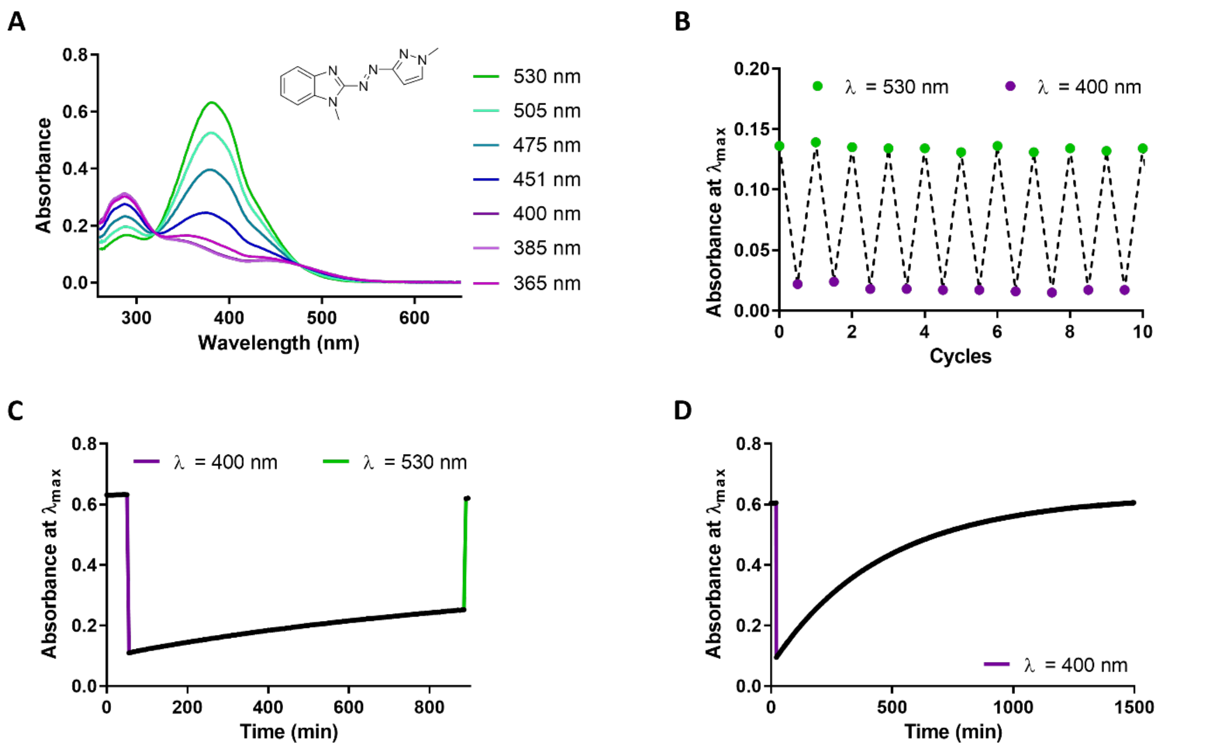

*Figure S 10. Photophysical properties of compound **8pz**. Absorption spectra: (A) after irradiation with different wavelengths of 30 µM compound in DMSO; (B) repeated E/Z-isomerization by alternating irradiation with 400 nm and 530 nm for 1 min in DMSO; (C) stability of Z-**8pz** in DMSO in the dark after switching with 400 nm, measured at 22°C; (D) stability of Z-**8pz** in TRIS-buffer (containing 25 % DMSO, pH= 7.4) in the dark after switching with 400 nm, measured at 37°C.*

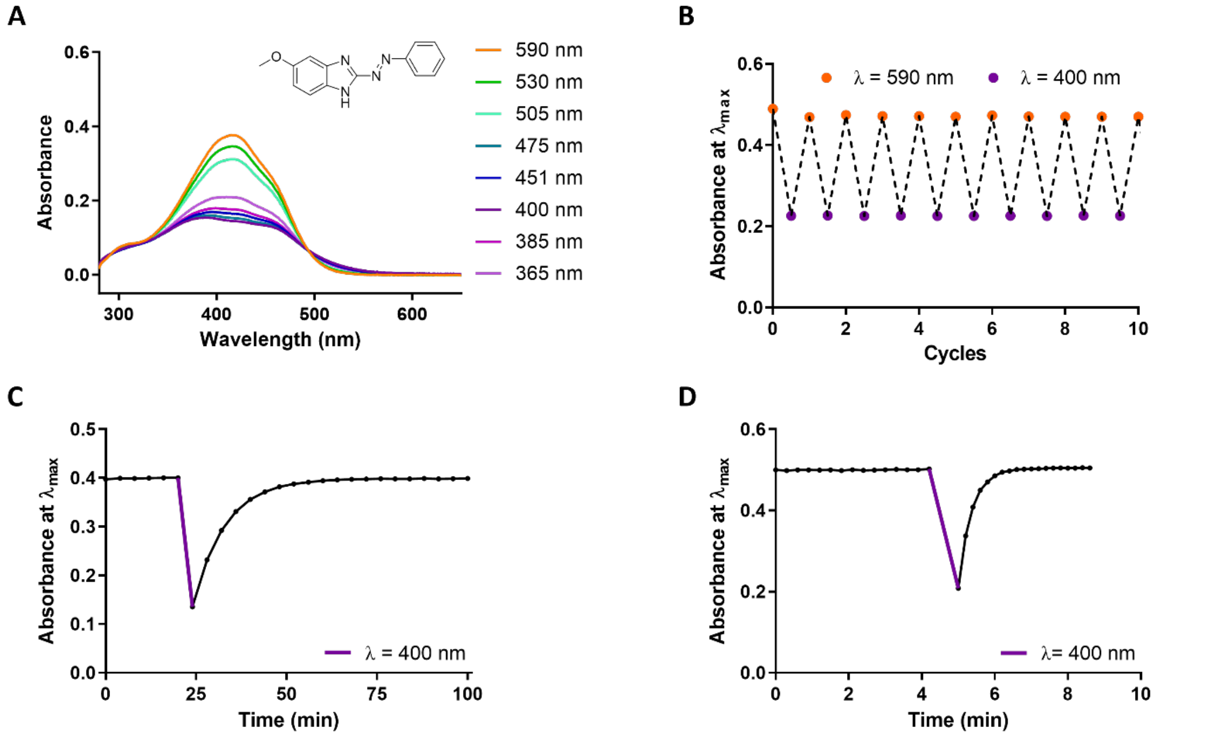

*Figure S 11. Photophysical properties of compound **13aH**. Absorption spectra: (A) after irradiation with different wavelengths of 30 µM compound in DMSO; (B) repeated E/Z-isomerization by alternating irradiation with 400 nm and 590 nm for 1 min in DMSO; (C) stability of Z-**13aH** in DMSO in the dark after switching with 400 nm, measured at 22°C; (D) stability of Z-**13aH** in TRIS-buffer (containing 25 % DMSO, pH= 7.4) in the dark after switching with 400 nm, measured at 37°C.*

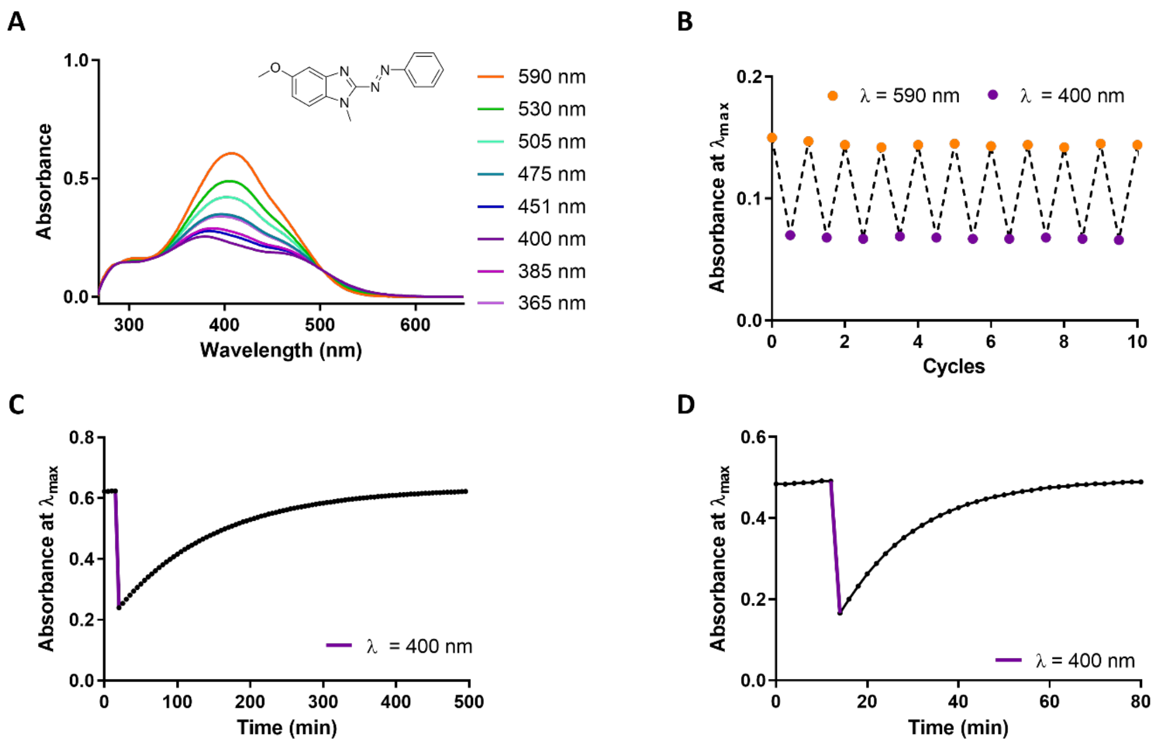

*Figure S 12. Photophysical properties of compound **13a**. Absorption spectra: (A) after irradiation with different wavelengths of 30 µM compound in DMSO; (B) repeated E/Z-isomerization by alternating irradiation with 400 nm and 590 nm for 1 min in DMSO; (C) stability of Z-**13a** in DMSO in the dark after switching with 400 nm, measured at 22°C; (D) stability of Z-**13a** in TRIS-buffer (containing 25 % DMSO, pH= 7.4) in the dark after switching with 400 nm, measured at 37°C.*

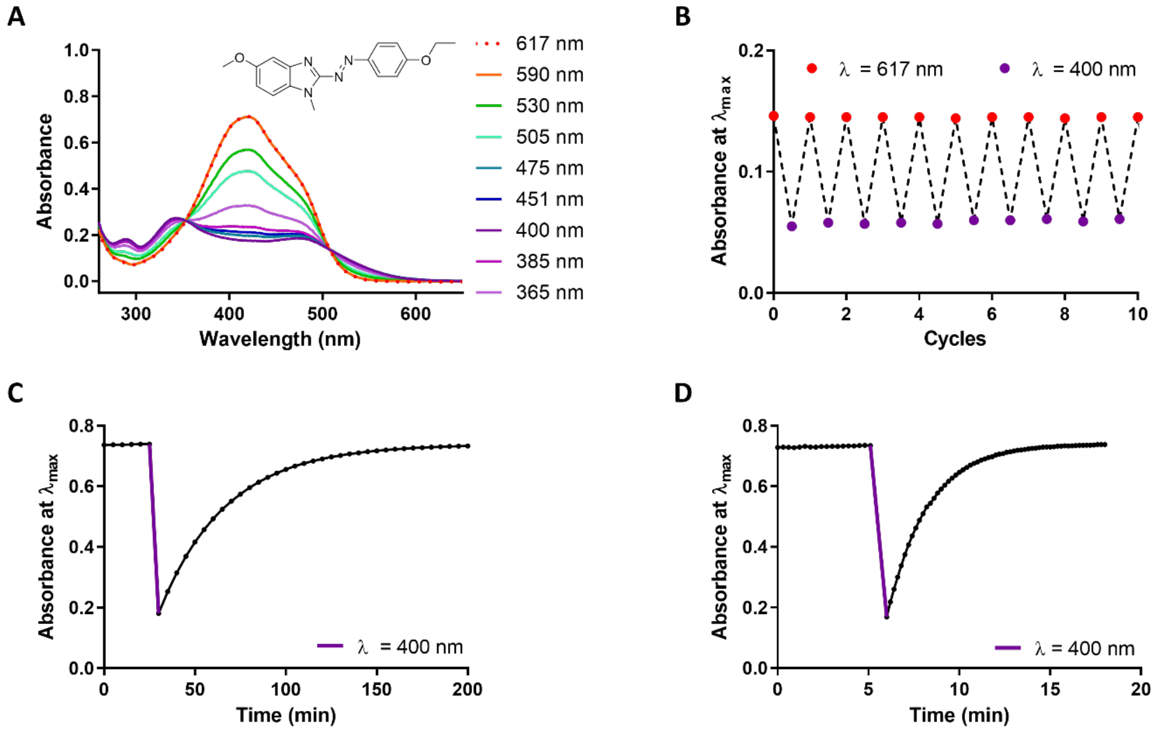

*Figure S 13. Photophysical properties of compound **13b**. Absorption spectra: (A) after irradiation with different wavelengths of 30 µM compound in DMSO; (B) repeated E/Z-isomerization by alternating irradiation with 400 nm and 617 nm for 1 min in DMSO; (C) stability of Z-**13b** in DMSO in the dark after switching with 400 nm, measured at 22°C; (D) stability of Z-**13b** in TRIS-buffer (containing 25 % DMSO, pH= 7.4) in the dark after switching with 400 nm, measured at 37°C.*

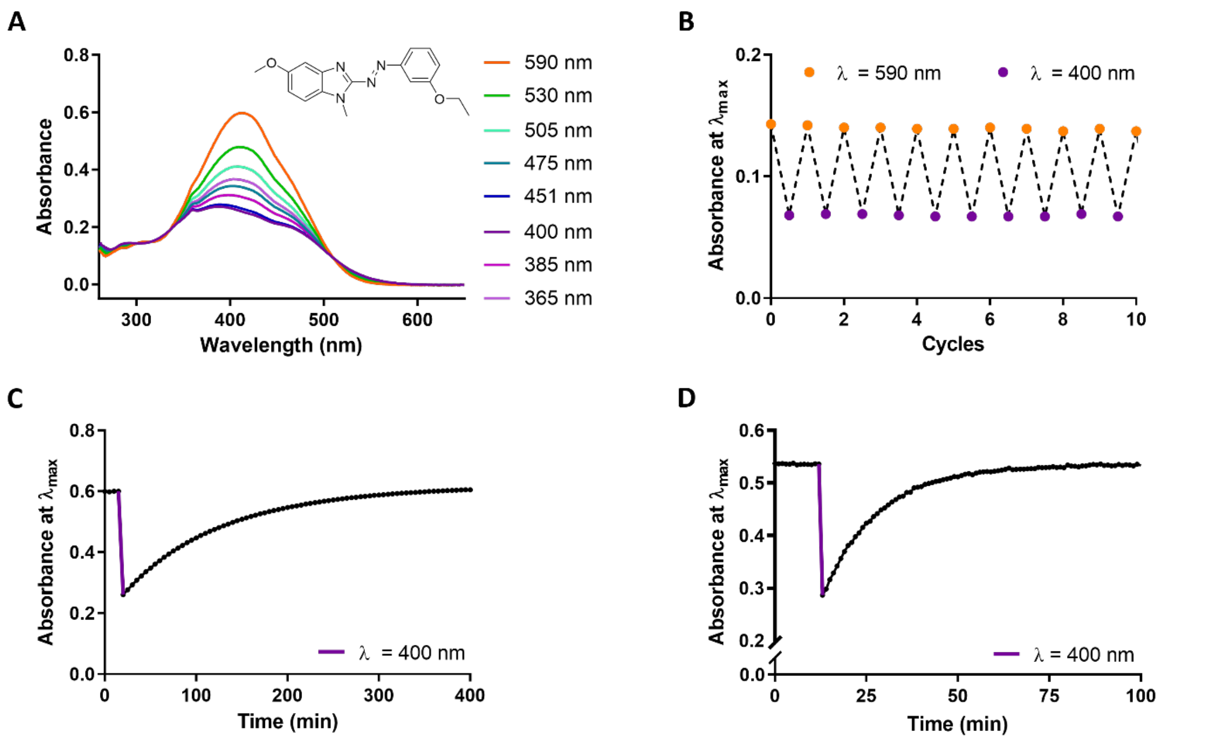

*Figure S 14. Photophysical properties of compound **13c**. Absorption spectra: (A) after irradiation with different wavelengths of 30 µM compound in DMSO; (B) repeated E/Z-isomerization by alternating irradiation with 400 nm and 590 nm for 1 min in DMSO; (C) stability of Z-**13c** in DMSO in the dark after switching with 400 nm, measured at 22°C; (D) stability of Z-**13c** in TRIS-buffer (containing 25 % DMSO, pH= 7.4) in the dark after switching with 400 nm, measured at 37°C.*

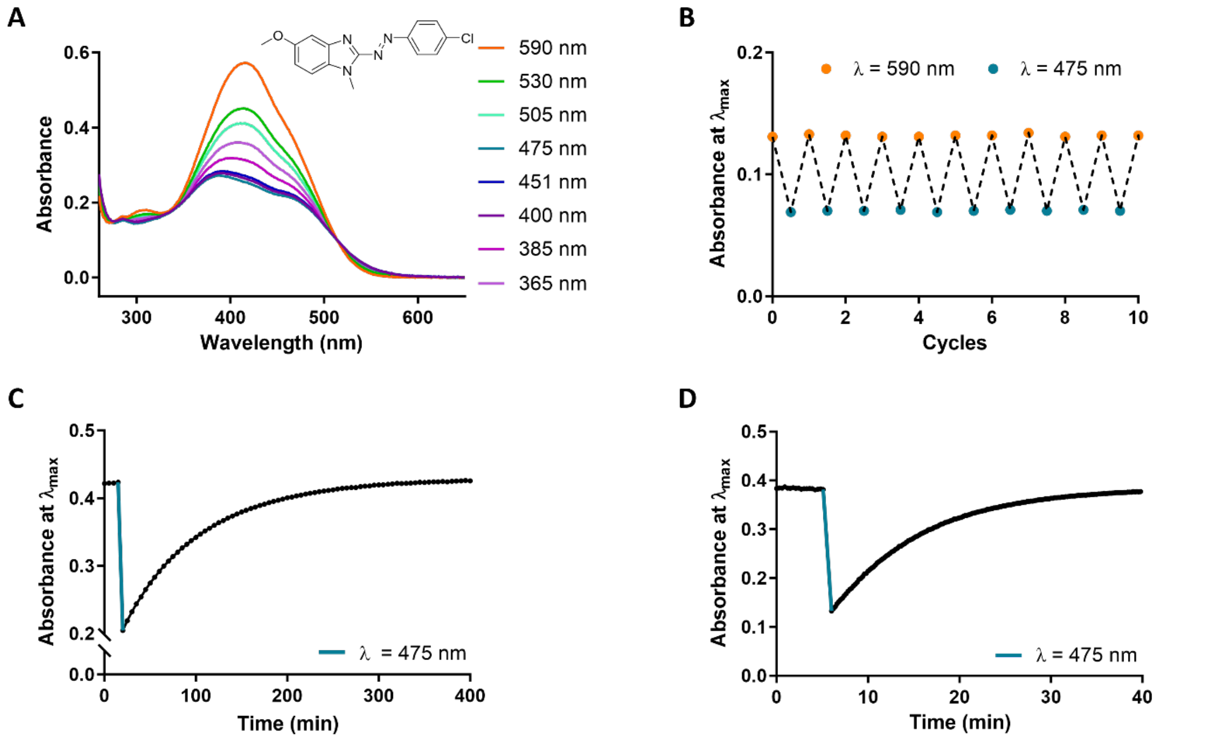

*Figure S 15. Photophysical properties of compound **13d**. Absorption spectra: (A) after irradiation with different wavelengths of 30 µM compound in DMSO; (B) repeated E/Z-isomerization by alternating irradiation with 475 nm and 590 nm for 1 min in DMSO; (C) stability of Z-**13d** in DMSO in the dark after switching with 475 nm, measured at 22°C; (D) stability of Z-**13d** in TRIS-buffer (containing 50 % DMSO, pH= 7.4) in the dark after switching with 475 nm, measured at 37°C.*

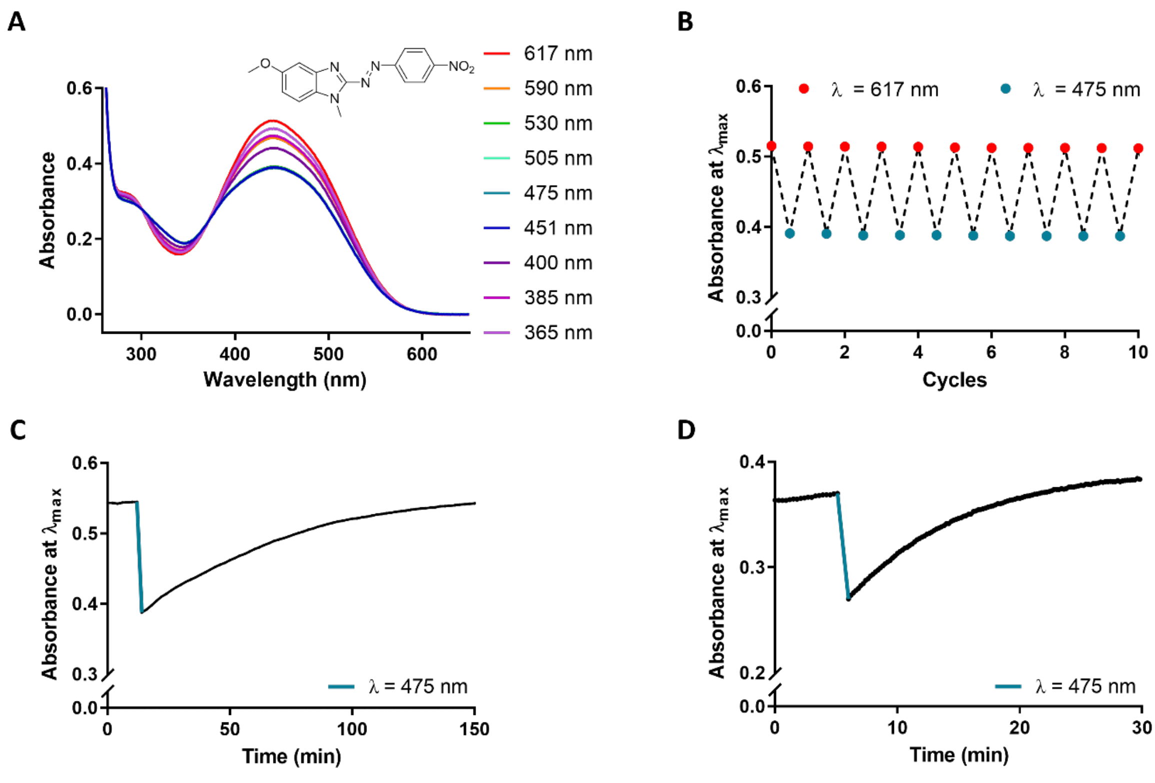

*Figure S 16. Photophysical properties of compound **13e**. Absorption spectra: (A) after irradiation with different wavelengths of 30 µM compound in DMSO; (B) repeated E/Z-isomerization by alternating irradiation with 475 nm and 617 nm for 1 min in DMSO; (C) stability of Z-**13e** in DMSO in the dark after switching with 475 nm, measured at 22°C; (D) stability of Z-**13e** in TRIS-buffer (containing 50 % DMSO, pH= 7.4) in the dark after switching with 475 nm, measured at 37°C.*

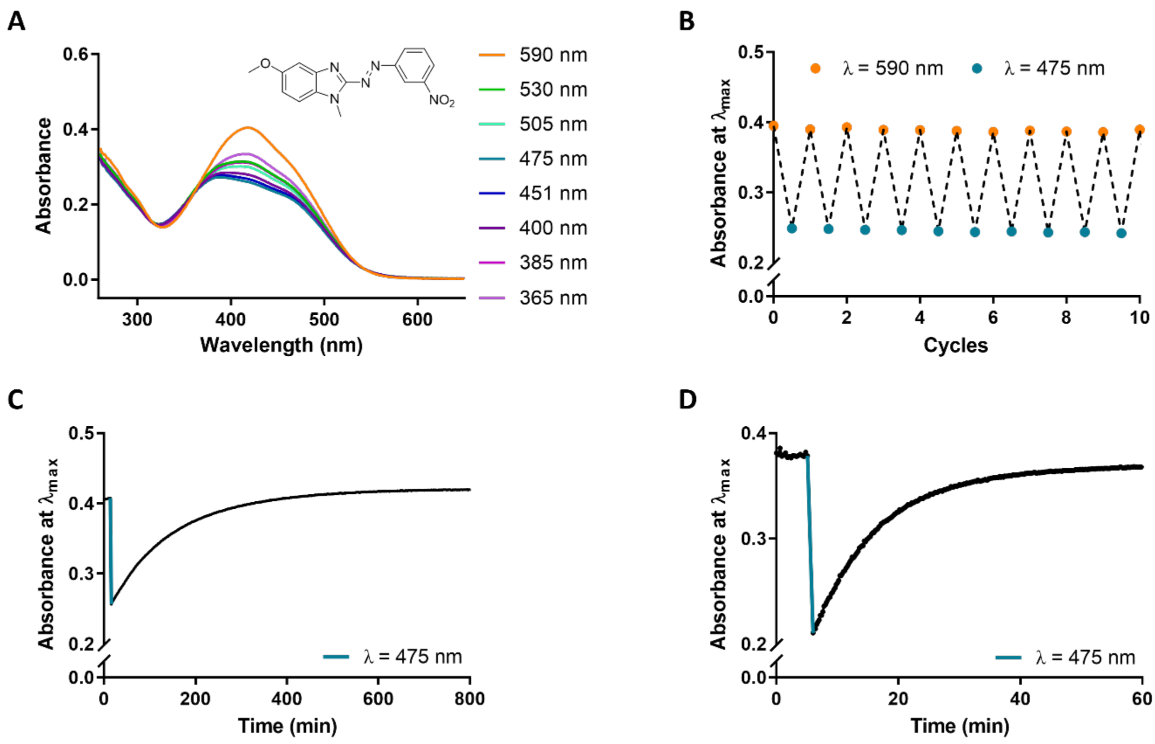

*Figure S 17. Photophysical properties of compound **13f**. Absorption spectra: (A) after irradiation with different wavelengths of 30 µM compound in DMSO; (B) repeated E/Z-isomerization by alternating irradiation with 475 nm and 590 nm for 1 min in DMSO; (C) stability of Z-**13f** in DMSO in the dark after switching with 475 nm, measured at 22°C; (D) stability of Z-**13f** in TRIS-buffer (containing 25 % DMSO, pH= 7.4) in the dark after switching with 475 nm, measured at 37°C.*

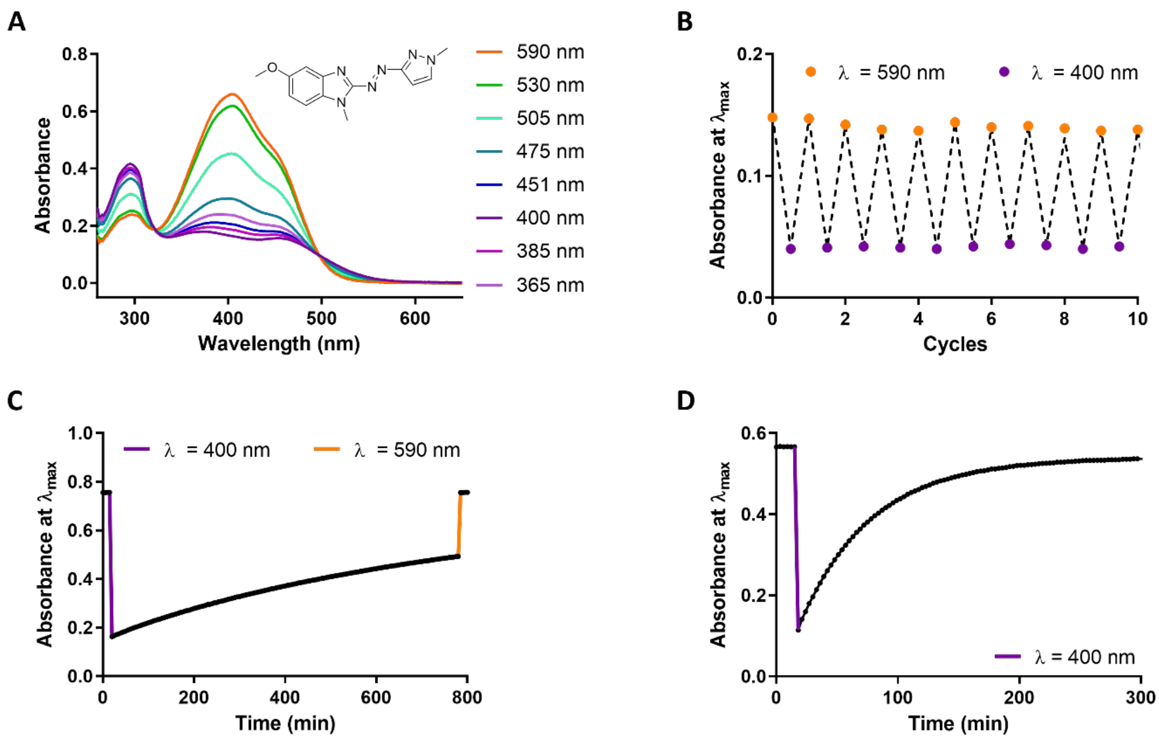

*Figure S 18. Photophysical properties of compound **13pz**. Absorption spectra: (A) after irradiation with different wavelengths of 30 µM compound in DMSO; (B) repeated E/Z-isomerization by alternating irradiation with 400 nm and 590 nm for 1 min in DMSO; (C) stability of Z-**13pz** in DMSO in the dark after switching with 400 nm, measured at 22°C; (D) stability of Z-**13pz** in TRIS-buffer (containing 25 % DMSO, pH= 7.4) in the dark after switching with 400 nm, measured at 37°C.*

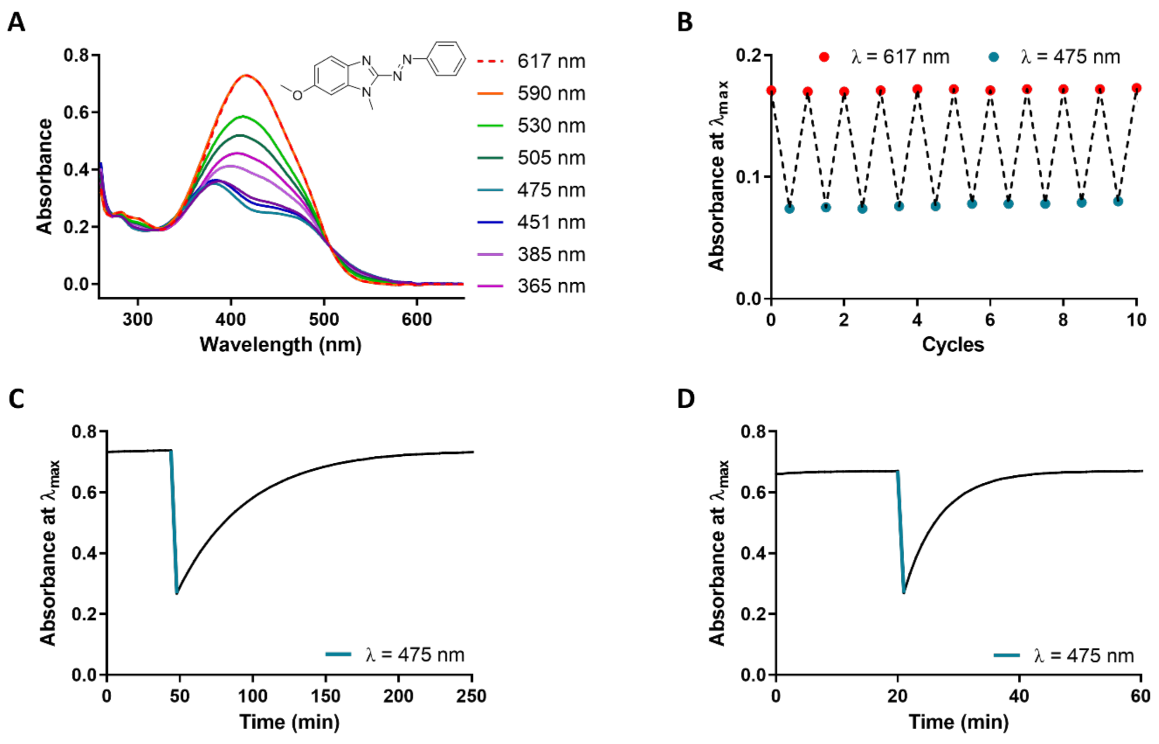

*Figure S 19. Photophysical properties of compound **18a**. Absorption spectra: (A) after irradiation with different wavelengths of 30 µM compound in DMSO; (B) repeated E/Z-isomerization by alternating irradiation with 475 nm and 617 nm for 1 min in DMSO; (C) stability of Z-**18a** in DMSO in the dark after switching with 475 nm, measured at 22°C; (D) stability of Z-**18a** in TRIS-buffer (containing 25 % DMSO, pH= 7.4) in the dark after switching with 475 nm, measured at 37°C.*

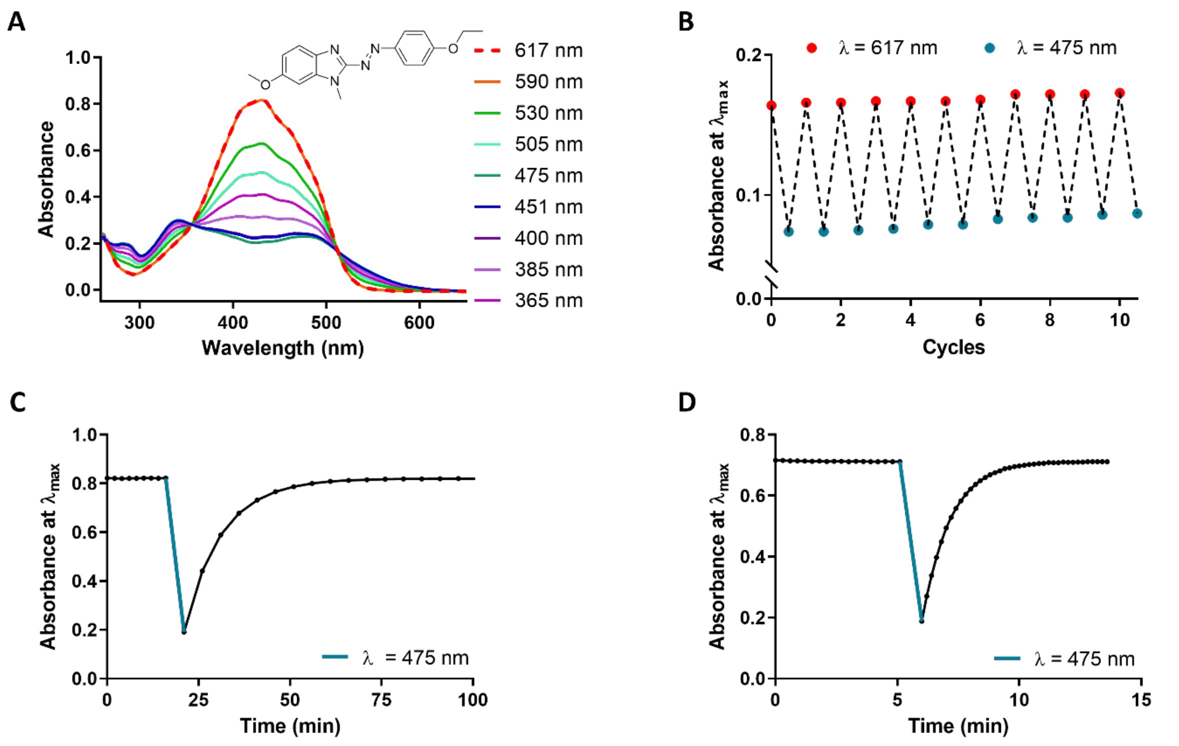

*Figure S 20. Photophysical properties of compound **18b**. Absorption spectra: (A) after irradiation with different wavelengths of 30 µM compound in DMSO; (B) repeated E/Z-isomerization by alternating irradiation with 475 nm and 617 nm for 1 min in DMSO; (C) stability of Z-**18b** in DMSO in the dark after switching with 475 nm, measured at 22°C; (D) stability of Z-**18b** in TRIS-buffer (containing 25 % DMSO, pH= 7.4) in the dark after switching with 475 nm, measured at 37°C.*

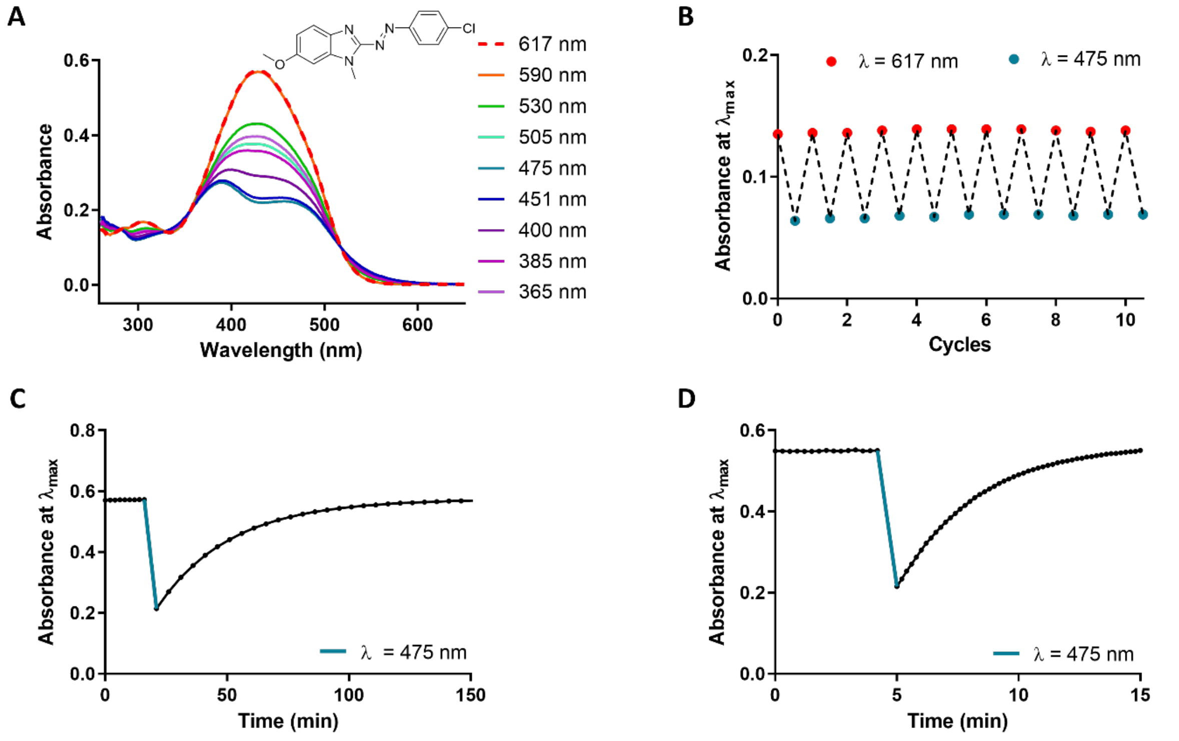

*Figure S 21. Photophysical properties of compound **18d**. Absorption spectra: (A) after irradiation with different wavelengths of 30 µM compound in DMSO; (B) repeated E/Z-isomerization by alternating irradiation with 475 nm and 617 nm for 1 min in DMSO; (C) stability of Z-**18d** in DMSO in the dark after switching with 475 nm, measured at 22°C; (D) stability of Z-**18d** in TRIS-buffer (containing 25 % DMSO, pH= 7.4) in the dark after switching with 475 nm, measured at 37°C.*

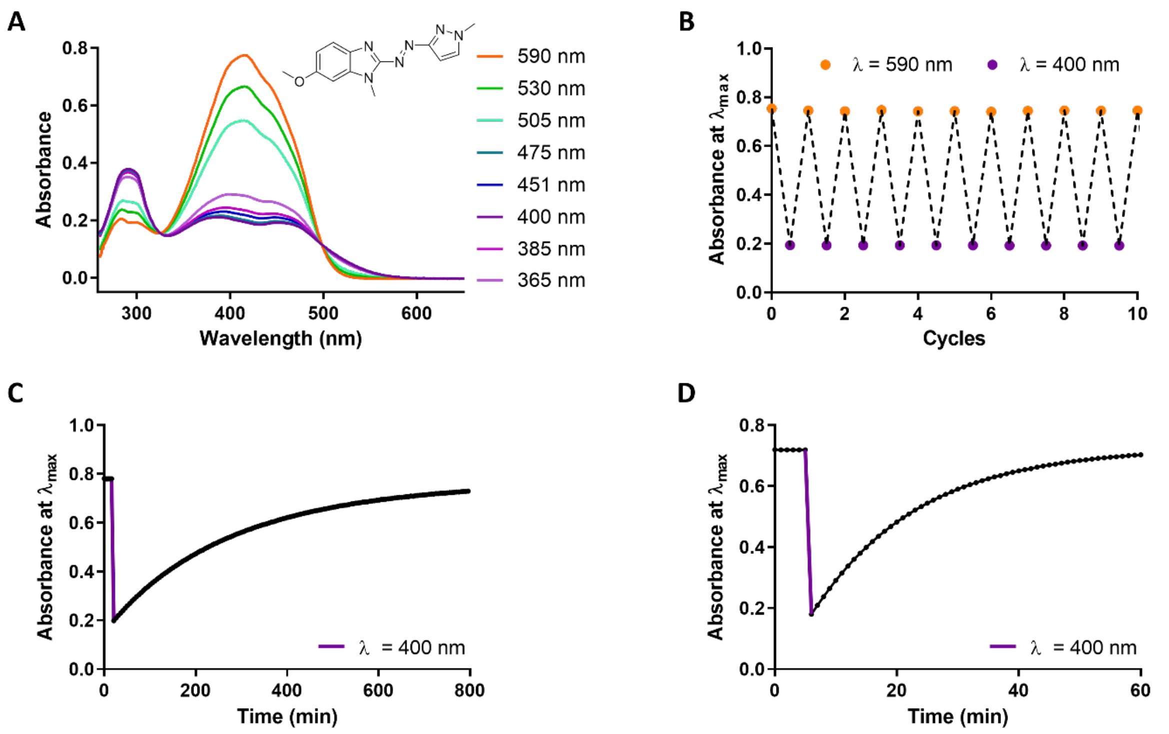

*Figure S 22. Photophysical properties of compound **18pz**. Absorption spectra: (A) after irradiation with different wavelengths of 30 µM compound in DMSO; (B) repeated E/Z-isomerization by alternating irradiation with 400 nm and 590 nm for 1 min in DMSO; (C) stability of Z-**18pz** in DMSO in the dark after switching with 400 nm, measured at 22°C; (D) stability of Z-**18pz** in TRIS-buffer (containing 25 % DMSO, pH= 7.4) in the dark after switching with 400 nm, measured at 37°C.*

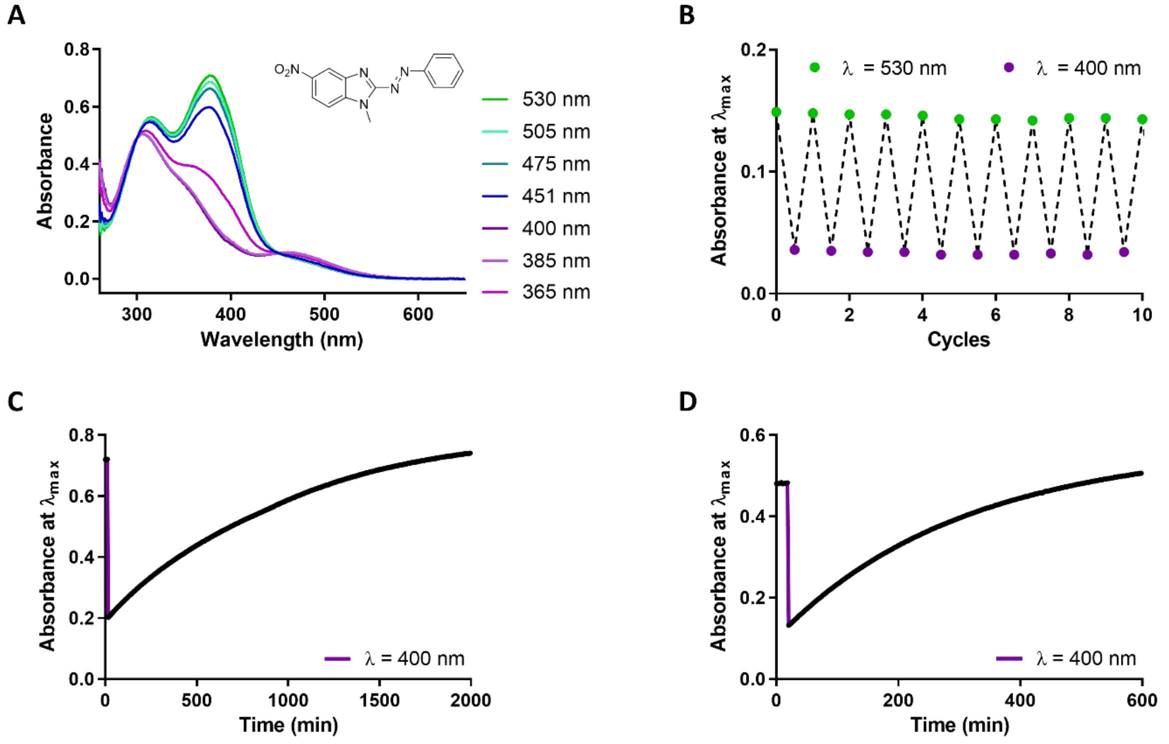

*Figure S 23. Photophysical properties of compound **23a**. Absorption spectra: (A) after irradiation with different wavelengths of 30 µM compound in DMSO; (B) repeated E/Z-isomerization by alternating irradiation with 400 nm and 530 nm for 1 min in DMSO; (C) stability of Z-**23a** in DMSO in the dark after switching with 400 nm, measured at 22°C; (D) stability of Z-**23a** in TRIS-buffer (containing 25 % DMSO, pH= 7.4) in the dark after switching with 400 nm, measured at 37°C.*

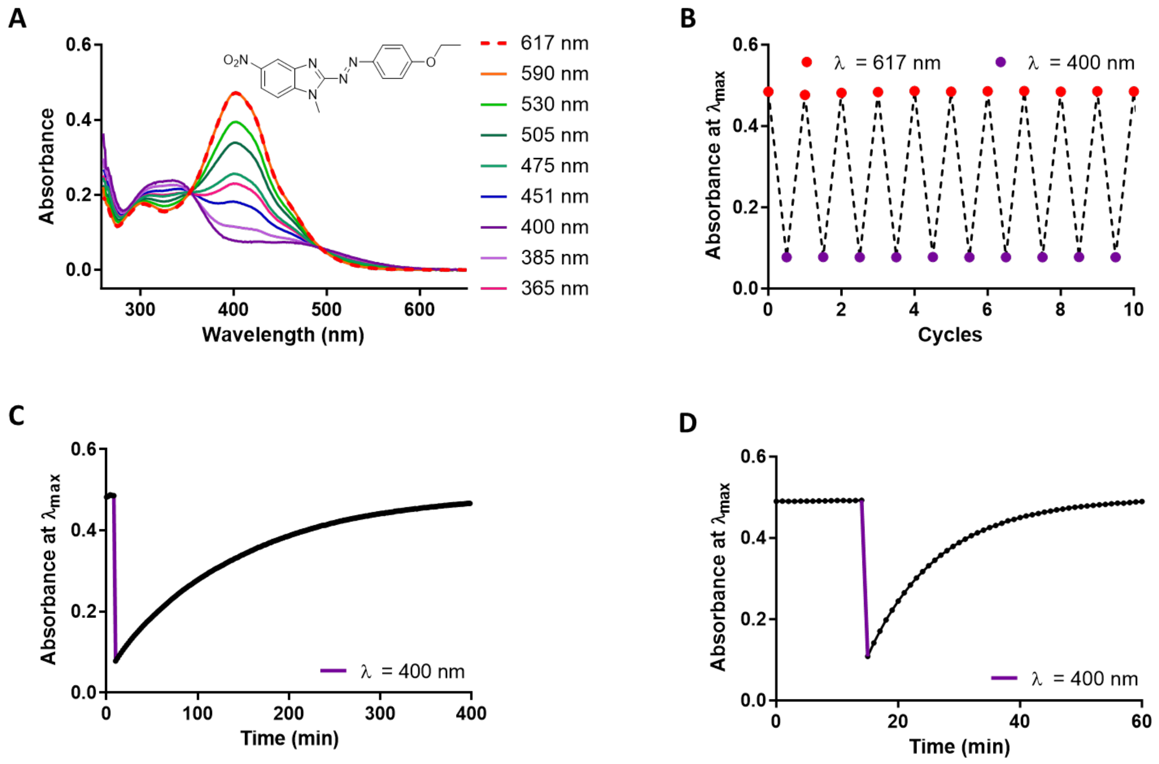

*Figure S 24. Photophysical properties of compound **23b**. Absorption spectra: (A) after irradiation with different wavelengths of 30 µM compound in DMSO; (B) repeated E/Z-isomerization by alternating irradiation with 400 nm and 617 nm for 1 min in DMSO; (C) stability of Z-**23a** in DMSO in the dark after switching with 400 nm, measured at 22°C; (D) stability of Z-**23a** in TRIS-buffer (containing 25 % DMSO, pH= 7.4) in the dark after switching with 400 nm, measured at 37°C.*

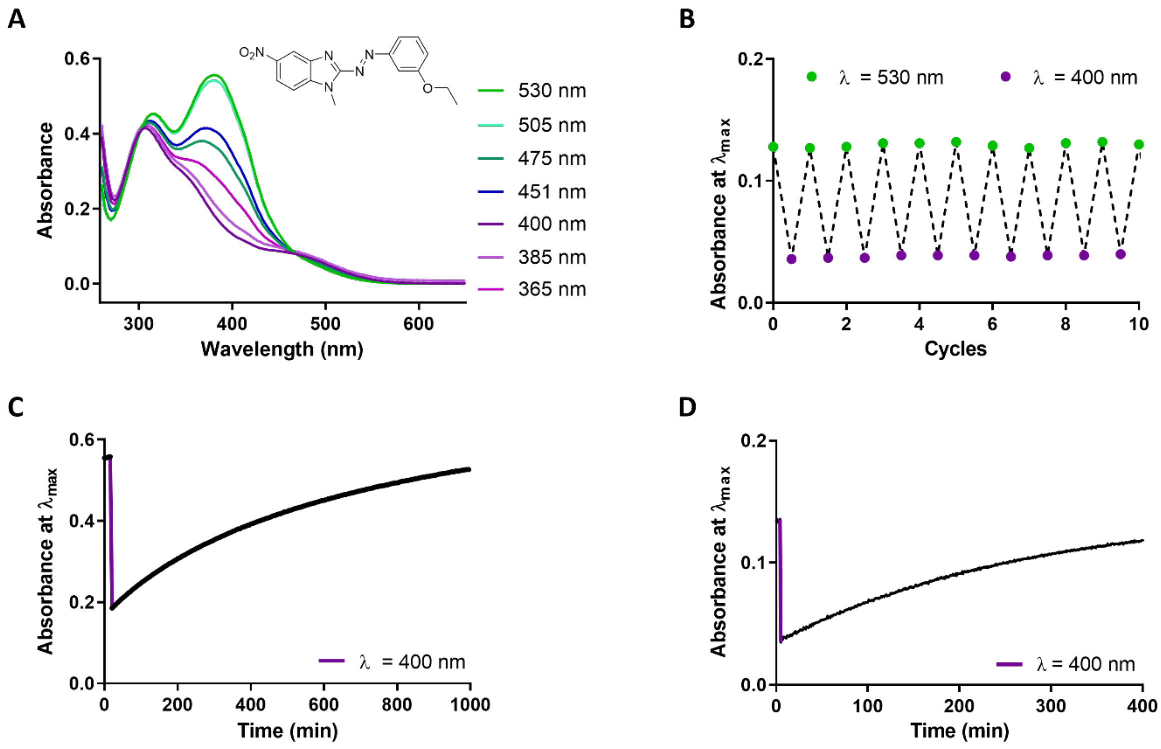

*Figure S 25. Photophysical properties of compound **23d**. Absorption spectra: (A) after irradiation with different wavelengths of 30 µM compound in DMSO; (B) repeated E/Z-isomerization by alternating irradiation with 400 nm and 530 nm for 1 min in DMSO; (C) stability of Z-**23c** in DMSO in the dark after switching with 400 nm, measured at 22°C; (D) stability of Z-**23a** in 1:3 TRIS-buffer/ DMSO (pH= 7.4) in the dark after switching with 400 nm, measured at 37°C.*

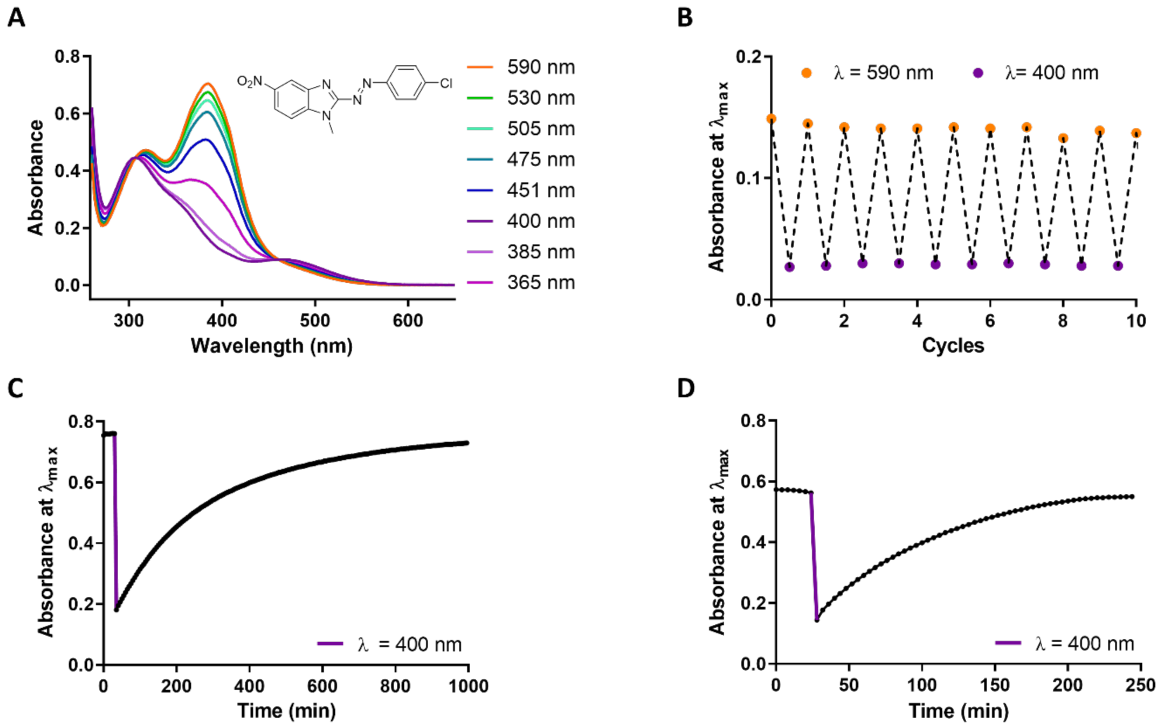

*Figure S 26. Photophysical properties of compound **23d**. Absorption spectra: (A) after irradiation with different wavelengths of 30 µM compound in DMSO; (B) repeated E/Z-isomerization by alternating irradiation with 400 nm and 590 nm for 1 min in DMSO; (C) stability of Z-**23d** in DMSO in the dark after switching with 400 nm, measured at 22°C; (D) stability of Z-**23d** in TRIS-buffer (containing 25 % DMSO, pH= 7.4) in the dark after switching with 400 nm, measured at 37°C.*

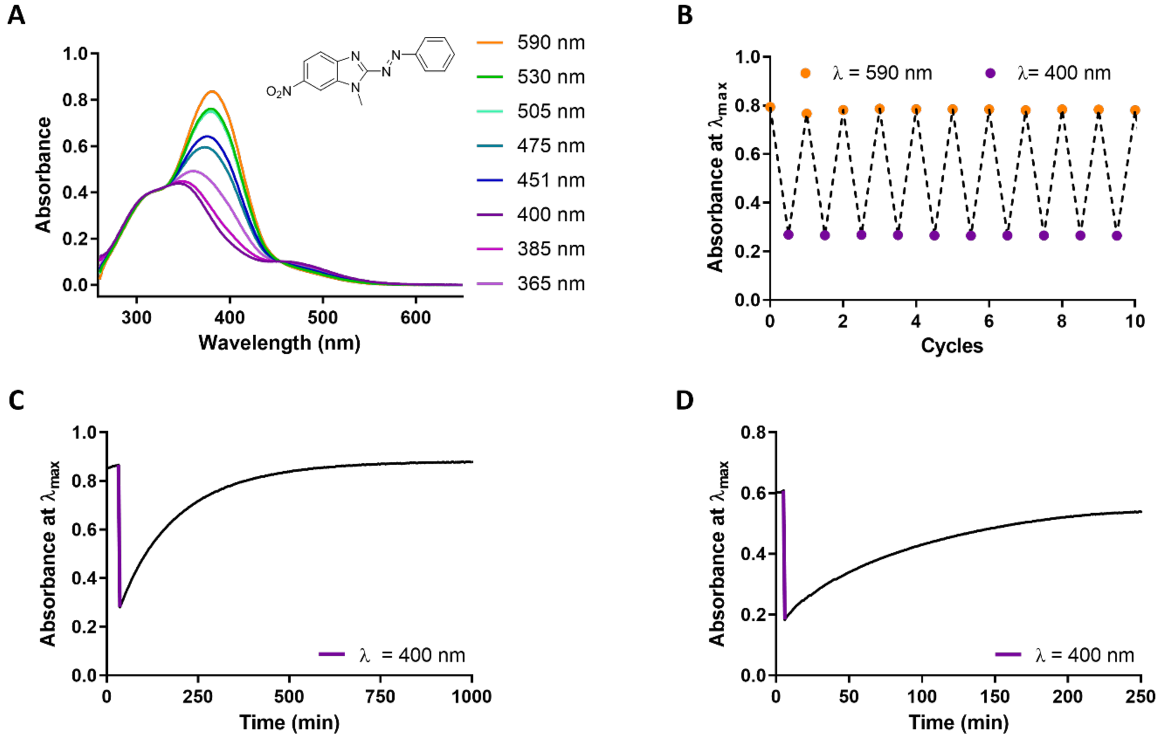

*Figure S 27. Photophysical properties of compound **26a**. Absorption spectra: (A) after irradiation with different wavelengths of 30 µM compound in DMSO; (B) repeated E/Z-isomerization by alternating irradiation with 400 nm and 590 nm for 1 min in DMSO; (C) stability of Z-**26a** in DMSO in the dark after switching with 400 nm, measured at 22°C; (D) stability of Z-**26a** in TRIS-buffer (containing 25 % DMSO, pH= 7.4) in the dark after switching with 400 nm, measured at 37°C.*

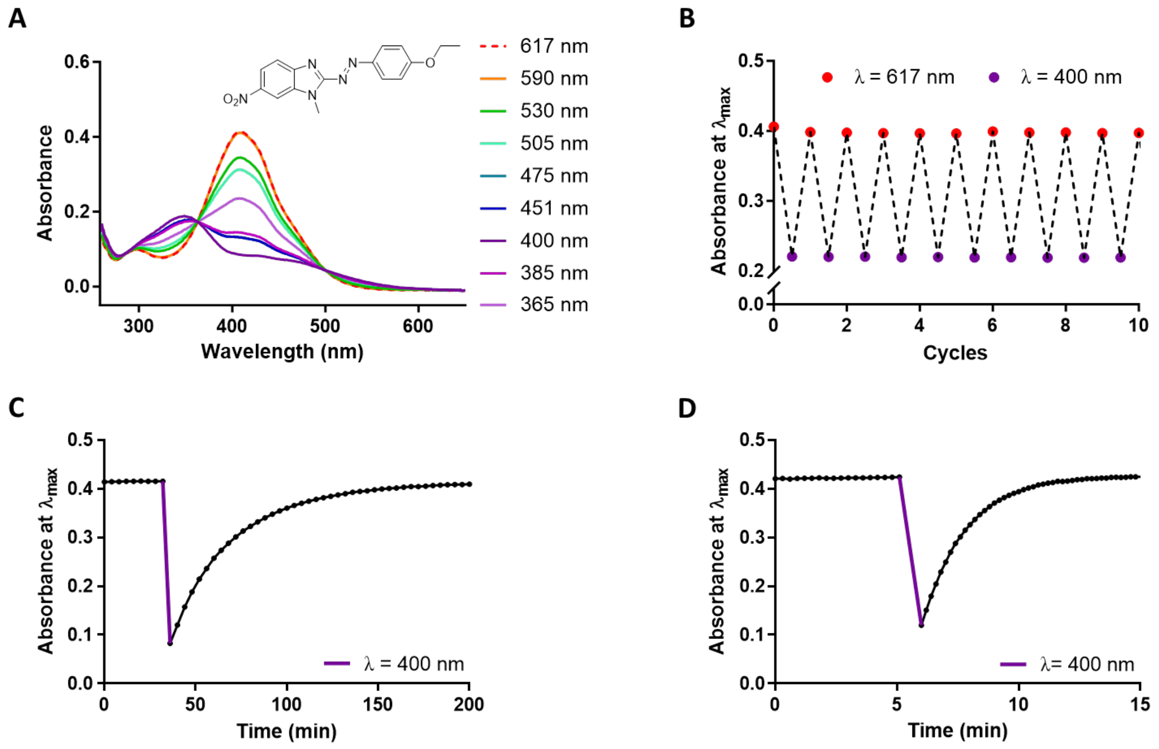

*Figure S 28. Photophysical properties of compound **26b** Absorption spectra: (A) after irradiation with different wavelengths of 30 µM compound in DMSO; (B) repeated E/Z-isomerization by alternating irradiation with 400 nm and 617 nm for 1 min in DMSO; (C) stability of Z-**26b** in DMSO in the dark after switching with 400 nm, measured at 22°C; (D) stability of Z-**26b** in TRIS-buffer (containing 25 % DMSO, pH= 7.4) in the dark after switching with 400 nm, measured at 37°C.*

E/*Z* Compositions of PSSs

The photostationary distribution (PSD) of the photostationary states (PSS) for *E*-isomers were determined by LC-MS. The amount of *Z*- and *E*-isomer was quantified by integration of the respective peak in the LC trace. Absorption was measured at the respective isosbestic point. Due to several *Z*-isomers displaying half-lives in the second to minute range, *Z*-isomer PSDs were too fast to be accurately investigated using LC-MS or NMR spectroscopy. Therefore, *Z*-isomer PSDs were estimated as previously described.[1] The respective *Z*-isomer PSD ratios were estimated based on the PSS with the highest concentration of *Z*-isomer.

Initially, the residual *E*-isomer in this PSS was estimated from the absorbance at the respective *E*-isomer absorption maximum (λ_{max} π→π*). Using this estimate, the spectrum of the pure *E*-isomer was subtracted from that PSS spectrum. The estimate for the residual *E*-isomer fraction was varied to obtain a range that predicted a sensible UV/vis spectrum for the pure *Z*-isomer. The maximum fraction of residual *E*-isomer represents the highest value that ensured that the absorbance remained positive at all wavelengths. The minimum fraction of residual *E*-isomer was determined as the lowest value which ensured no obvious remaining *E*-isomer π- π* absorbance in the spectrum. The centre point of this range was used to assign the respective *Z*-isomer PSD. From this, the authentic Z-isomer UV/vis spectrum was extrapolated with an approximate 3-4% absolute uncertainty in absorbance. To ensure comparability of PSDs across the scaffold, all PSDs were analyzed using the same methods.

LC/MS Data

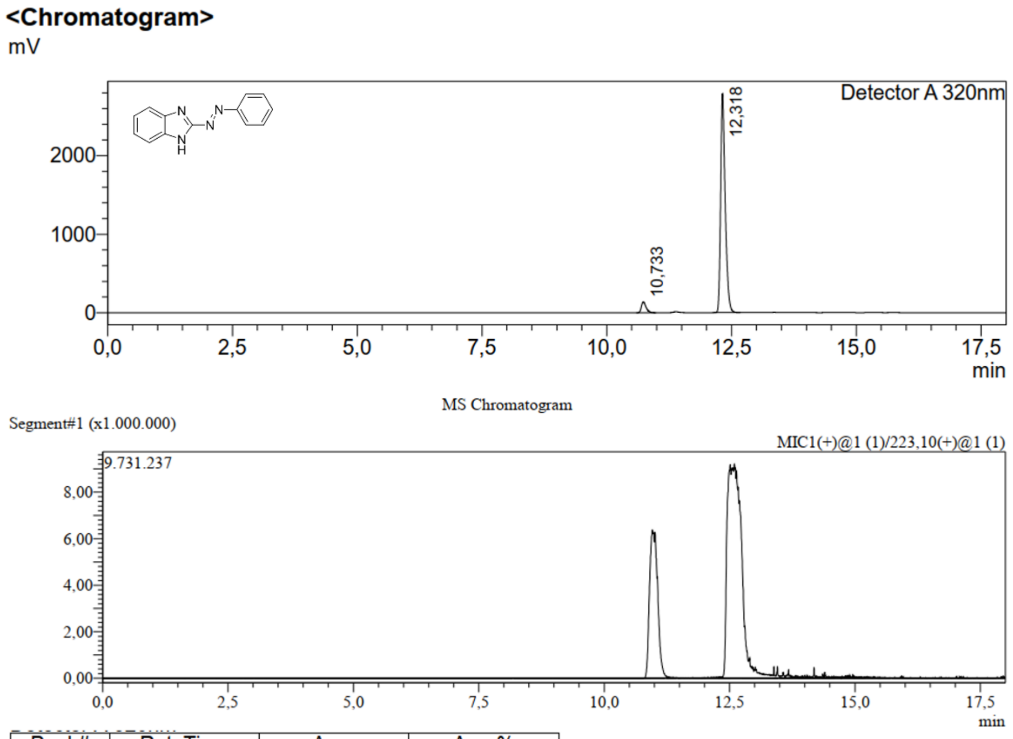

Peak#	Ret. Time	Area	Area%
1	10,733	899184	4,861
2	12,318	17598895	95,139
Total		18498079	100,000

<Chromatogram>

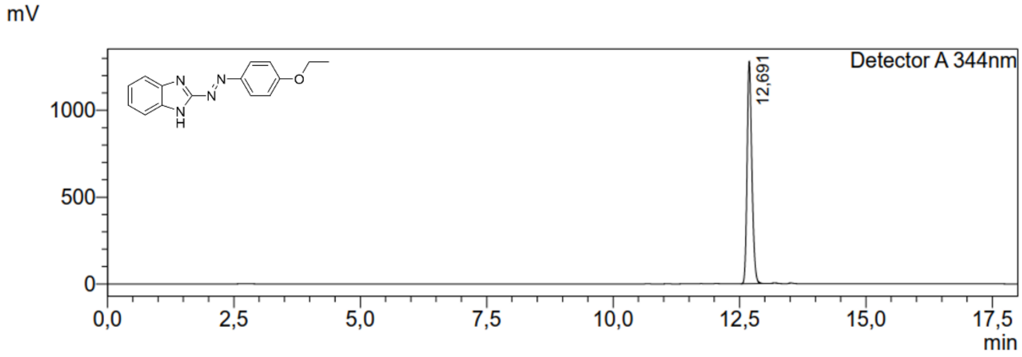

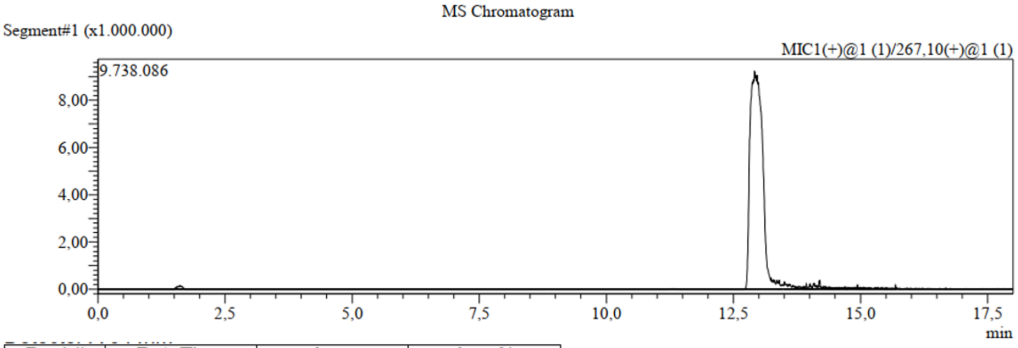

Peak#	Ret. Time	Area	Area%
1	12,691	8367641	100,000
Total		8367641	100,000

<Chromatogram>

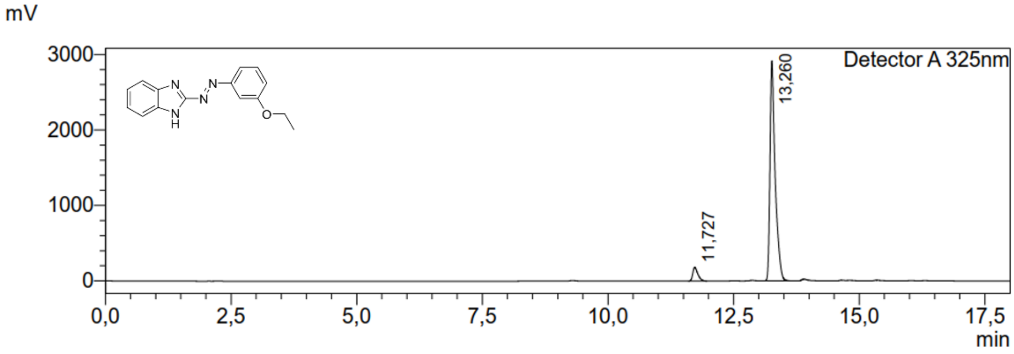

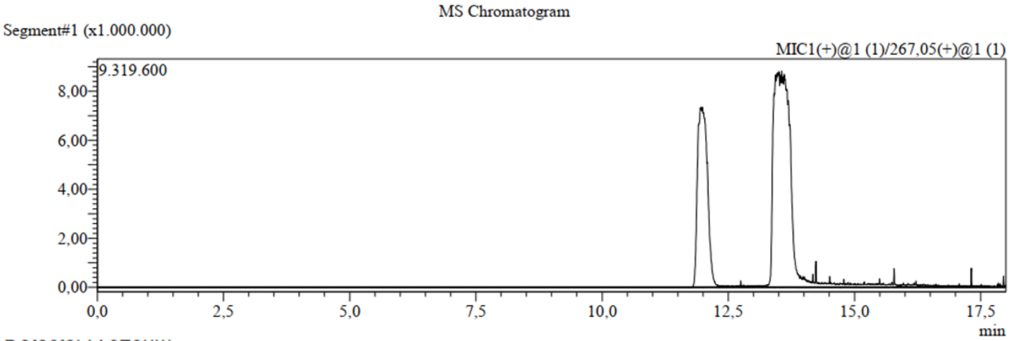

Peak#	Ret. Time	Area	Area%
1	11,727	1262204	5,690
2	13,260	20921799	94,310
Total		22184003	100,000

<Chromatogram>

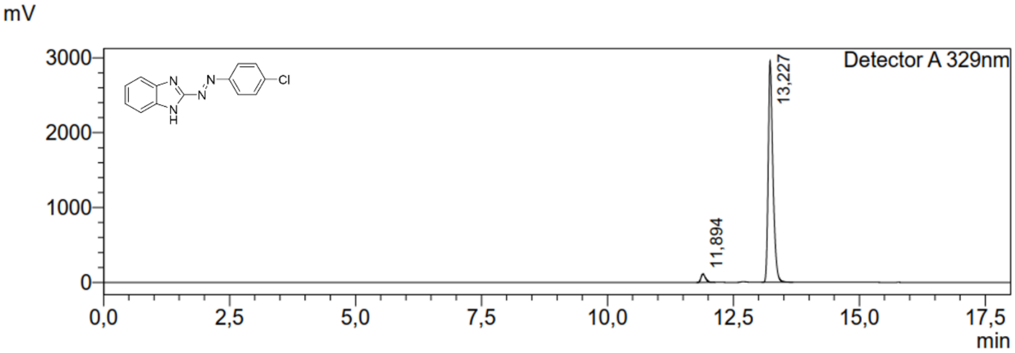

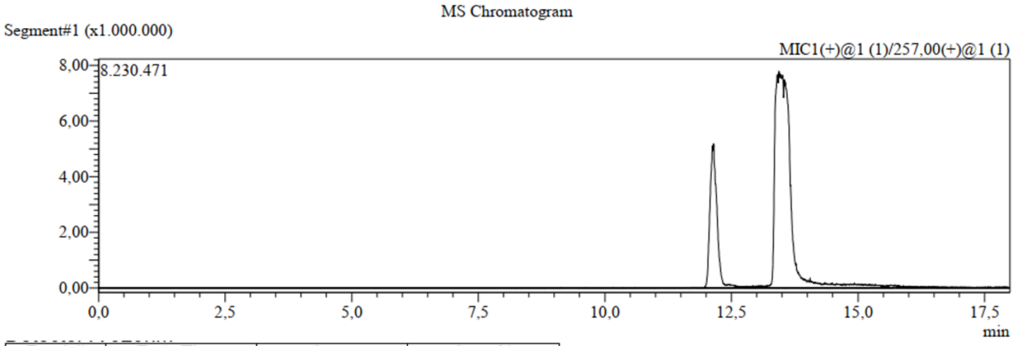

Peak#	Ret. Time	Area	Area%
1	11,894	702580	3,545
2	13,227	19118006	96,455
Total		19820586	100,000

<Chromatogram>

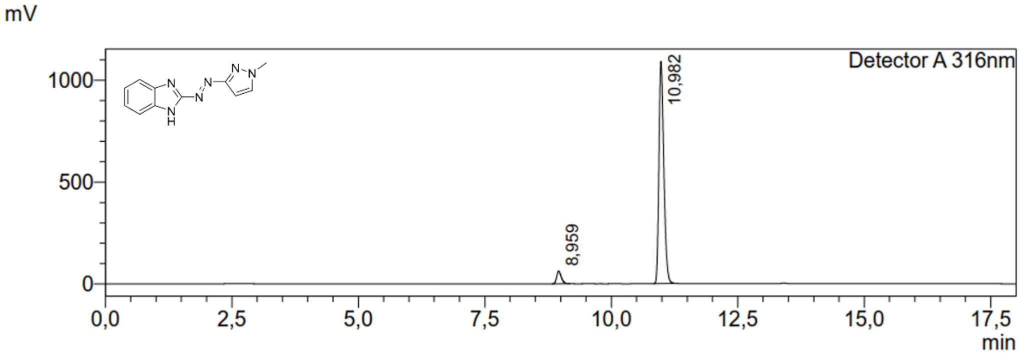

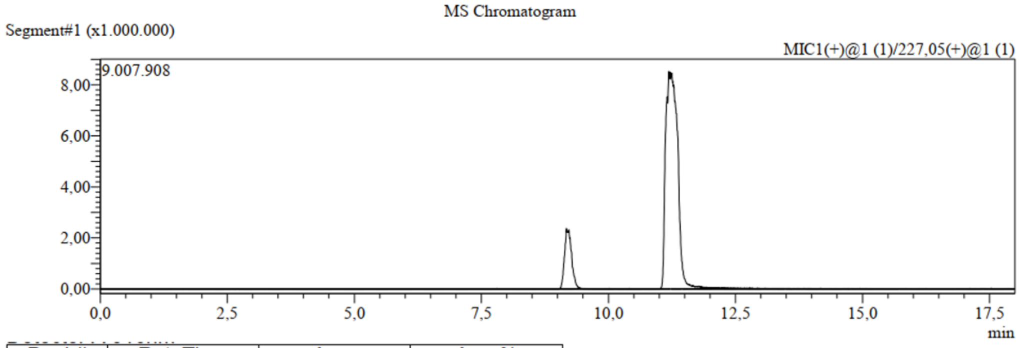

Peak#	Ret. Time	Area	Area%
1	8,959	401688	5,201
2	10,982	7321195	94,799

<Chromatogram>

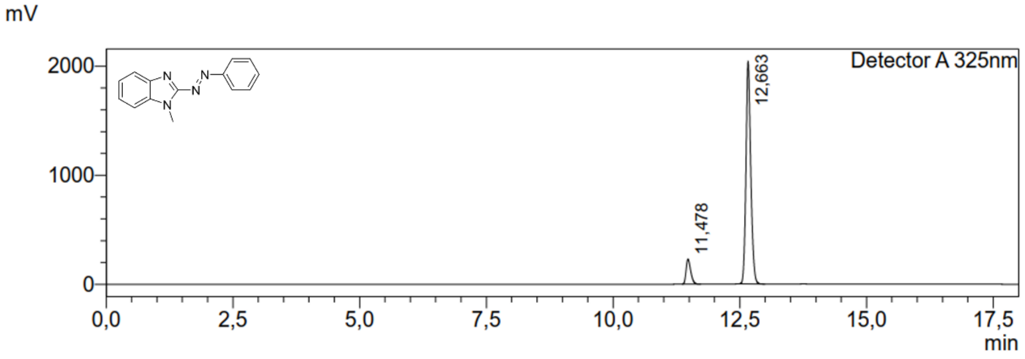

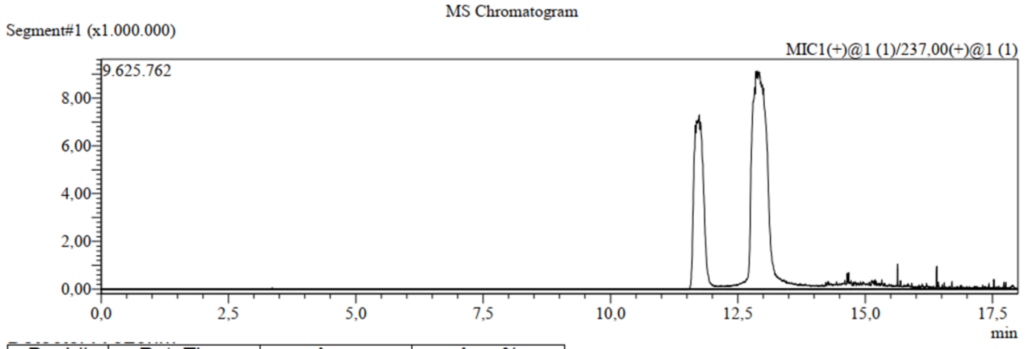

Peak#	Ret. Time	Area	Area%
1	11,478	1455174	9,835
2	12,663	13340311	90,165
Total		14795485	100,000

<Chromatogram>

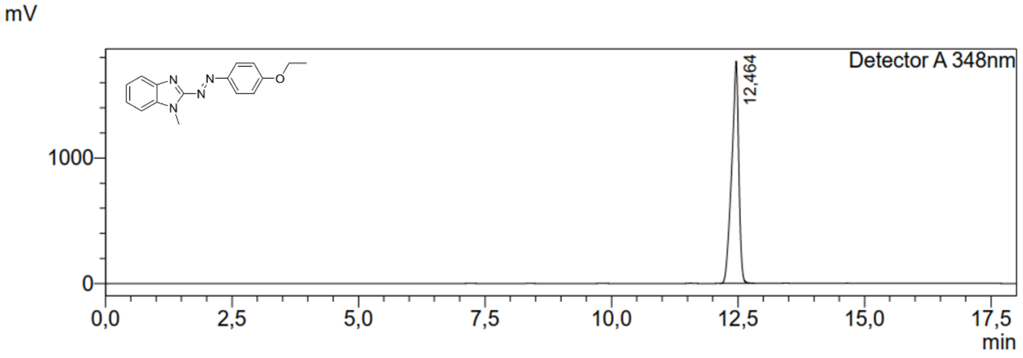

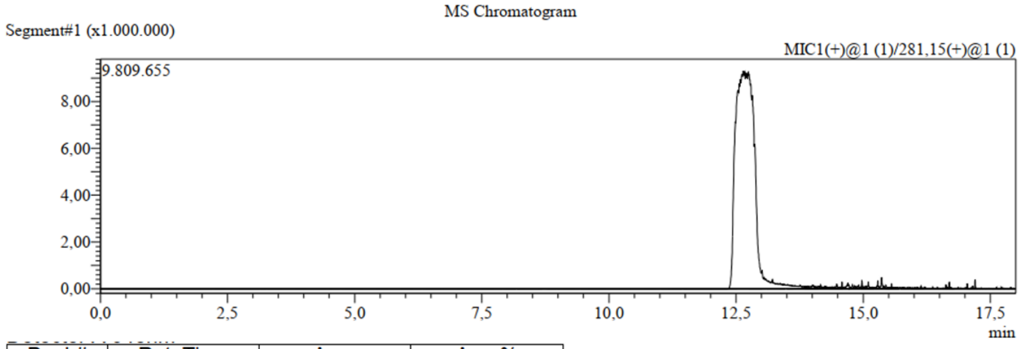

Peak#	Ret. Time	Area	Area%
1	12,464	17193564	100,000
Total		17193564	100,000

<Chromatogram>

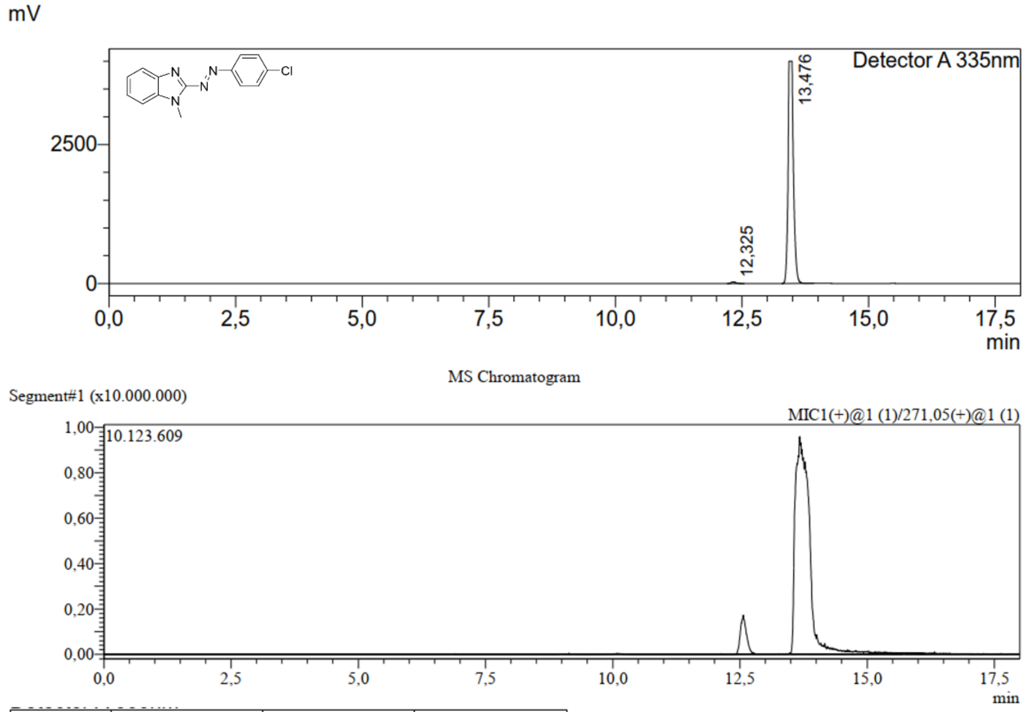

Peak#	Ret. Time	Area	Area%
1	12,325	169599	0,578
2	13,476	29169414	99,422
Total		29339012	100,000

<Chromatogram>

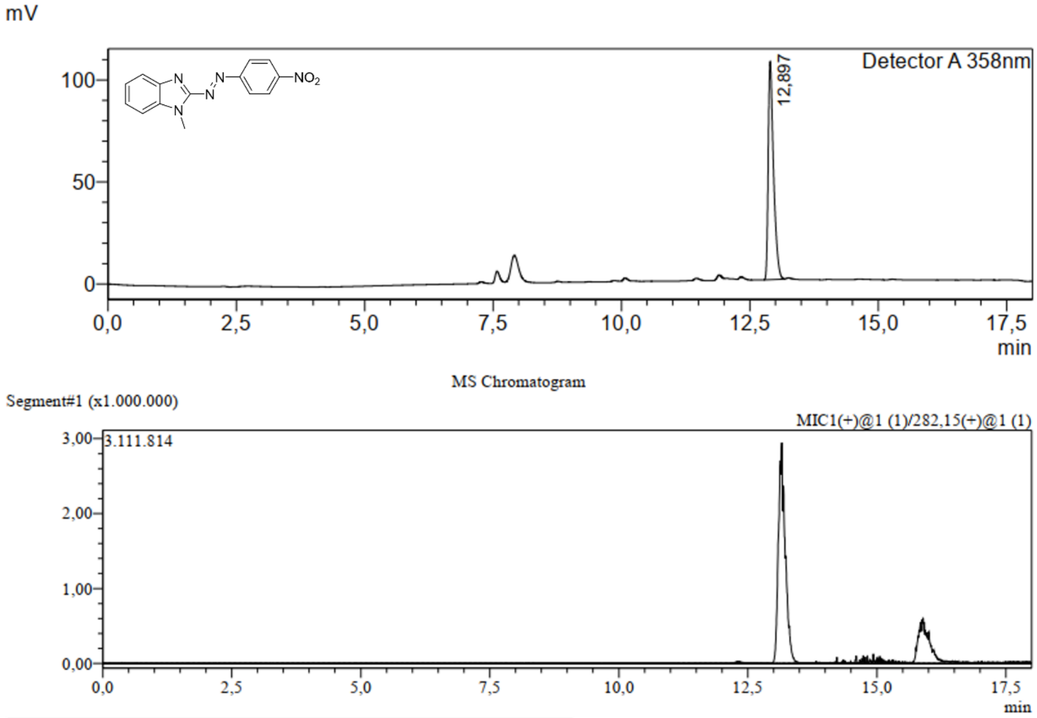

Peak#	Ret. Time	Area	Area%
1	12,897	770649	100,000
Total		770649	100,000

<Chromatogram>

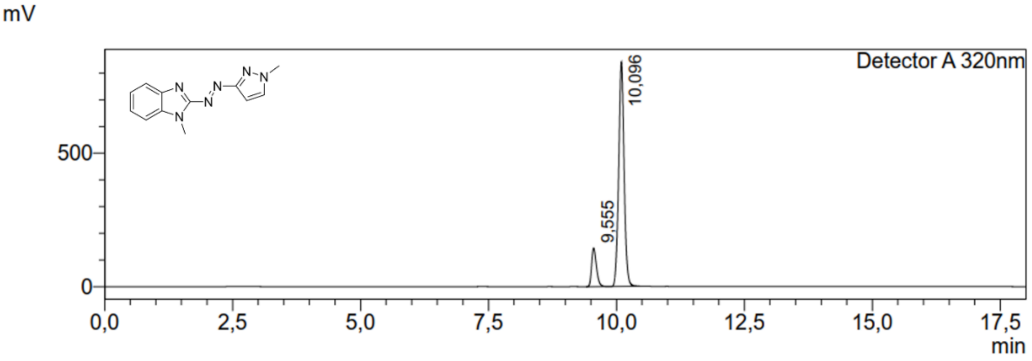

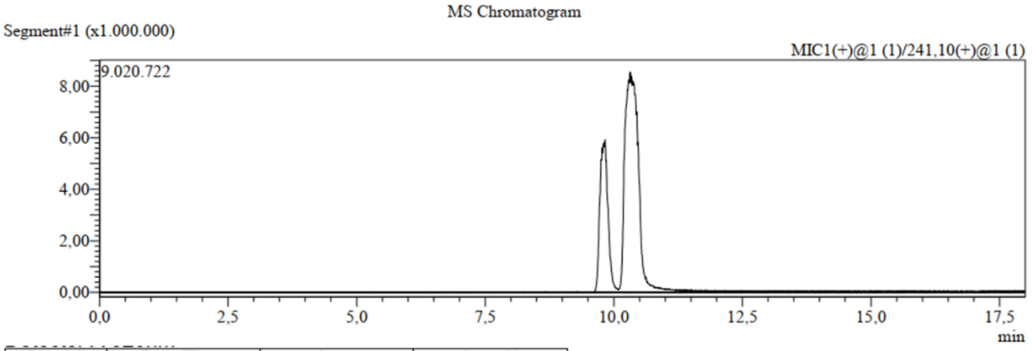

Peak#	Ret. Time	Area	Area%
1	9,555	943410	13,299
2	10,096	6150177	86,701
Total		7093587	100,000

<Chromatogram>

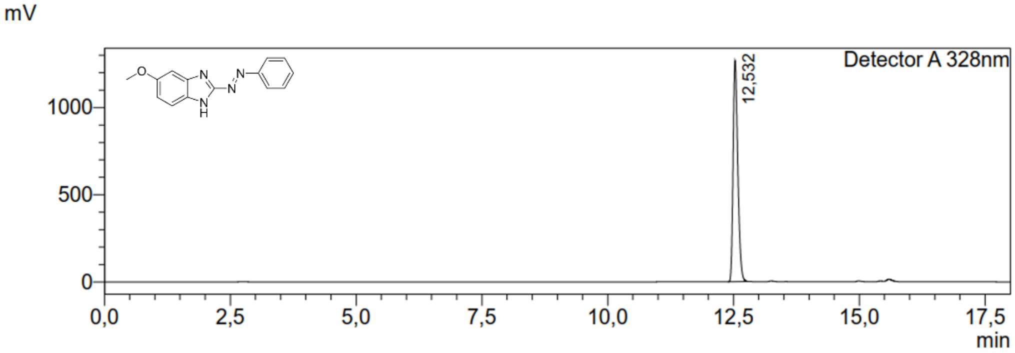

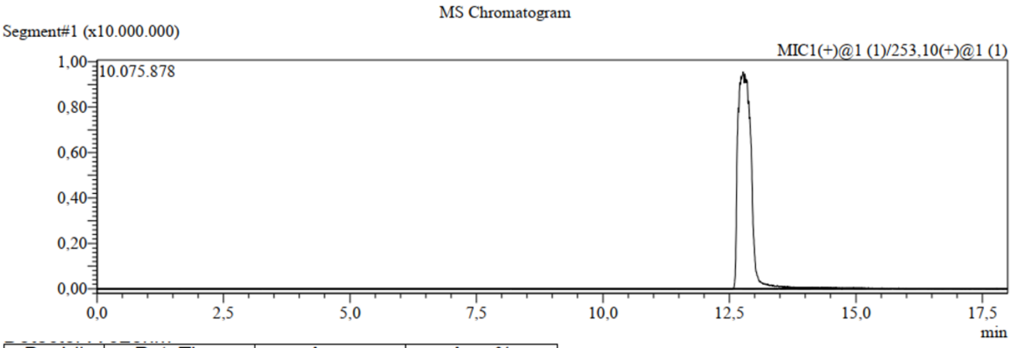

Peak#	Ret. Time	Area	Area%
1	12,532	8164613	100,000
Total		8164613	100,000

<Chromatogram>

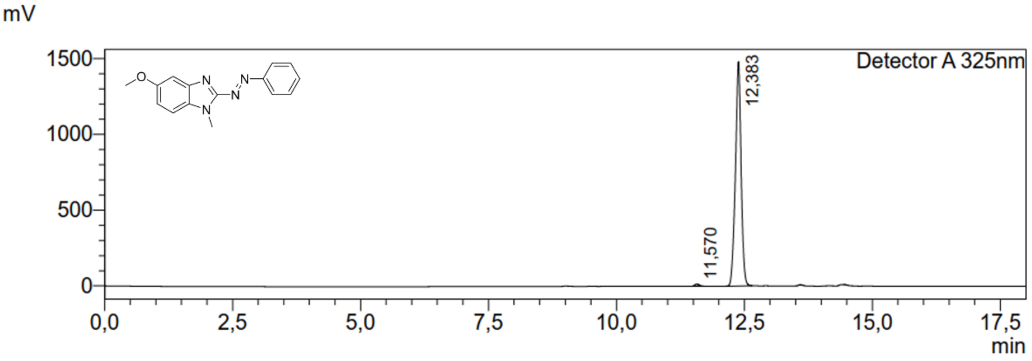

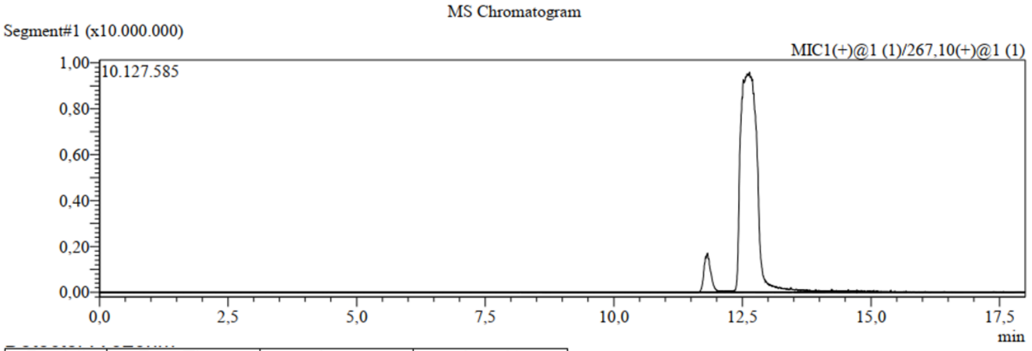

Peak#	Ret. Time	Area	Area%
1	11,570	86740	0,754
2	12,383	11418339	99,246
Total		11505079	100,000

<Chromatogram>

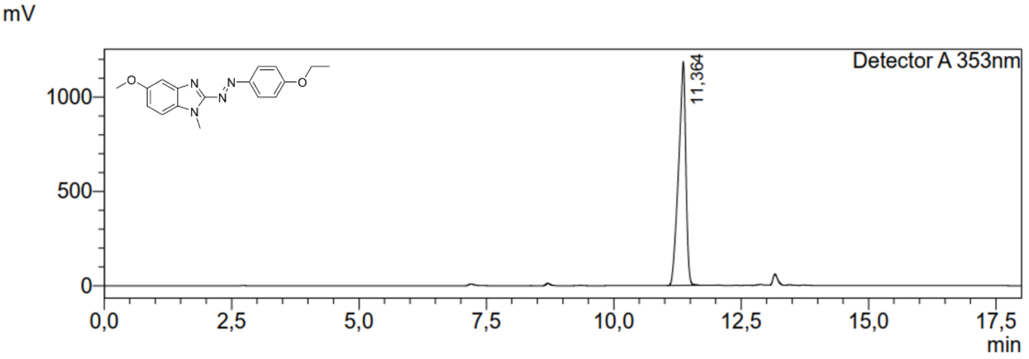

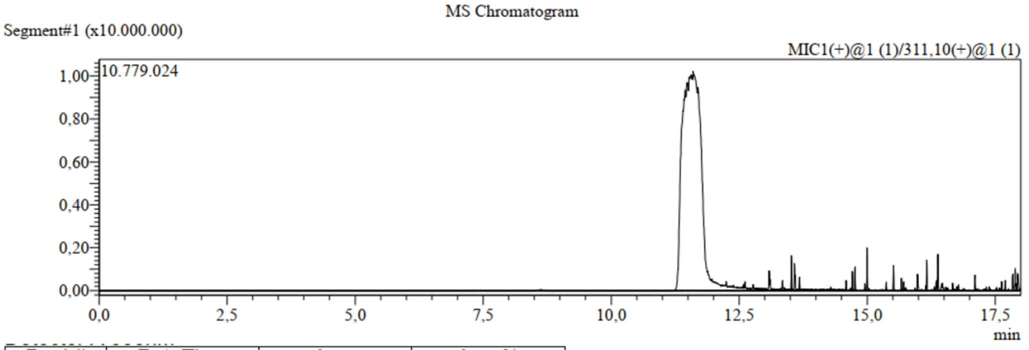

Peak#	Ret. Time	Area	Area%
1	11,364	11702007	100,000
Total		11702007	100,000

<Chromatogram>

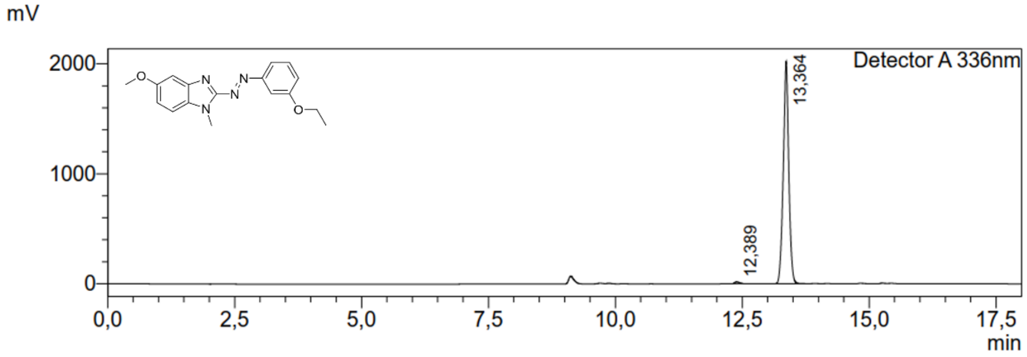

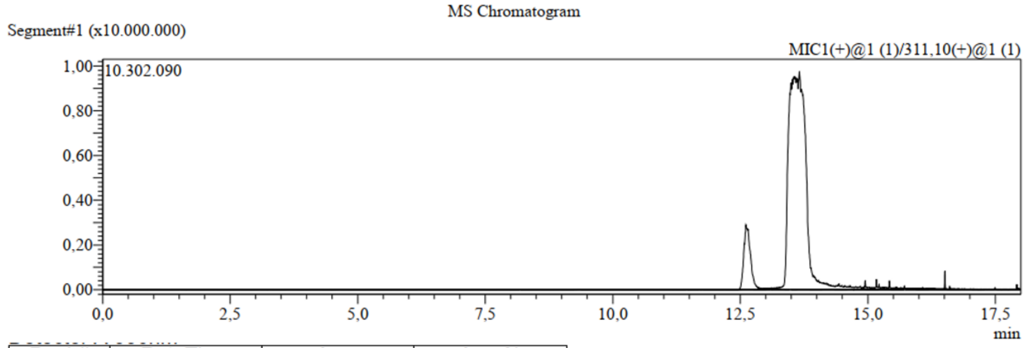

Peak#	Ret. Time	Area	Area%
1	12,389	97994	0,642
2	13,364	15160476	99,358
Total		15258470	100,000

<Chromatogram>

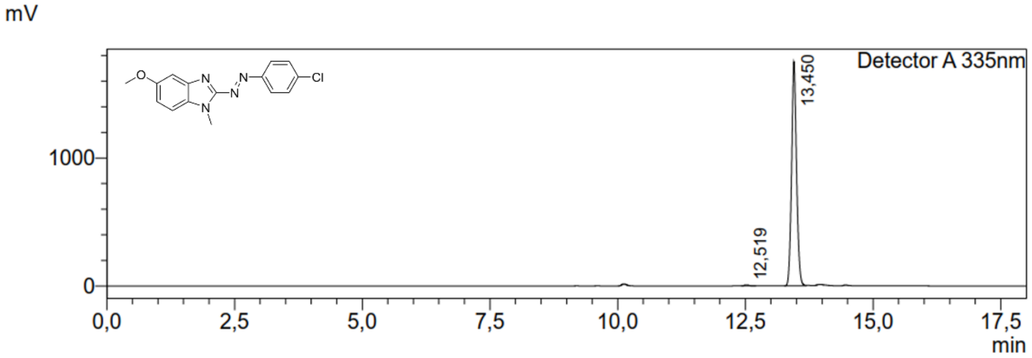

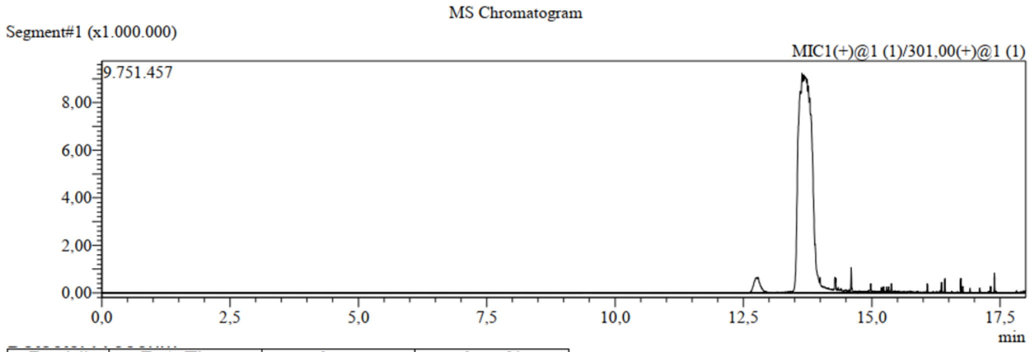

Peak#	Ret. Time	Area	Area%
1	12,519	49896	0,424
2	13,450	11722968	99,576
Total		11772865	100,000

<Chromatogram>

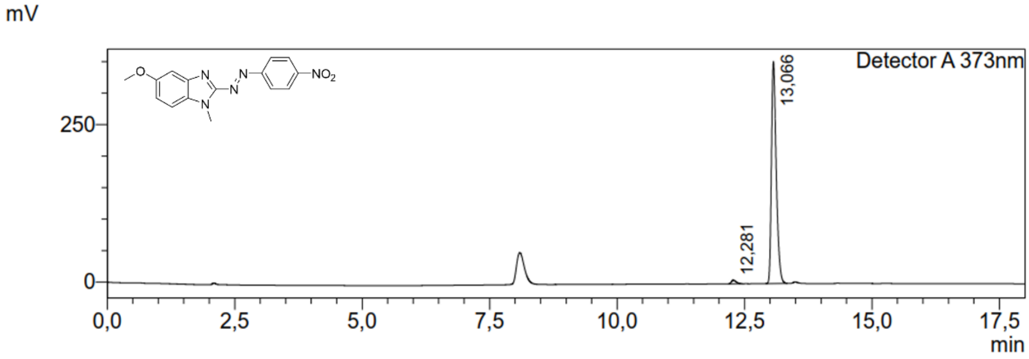

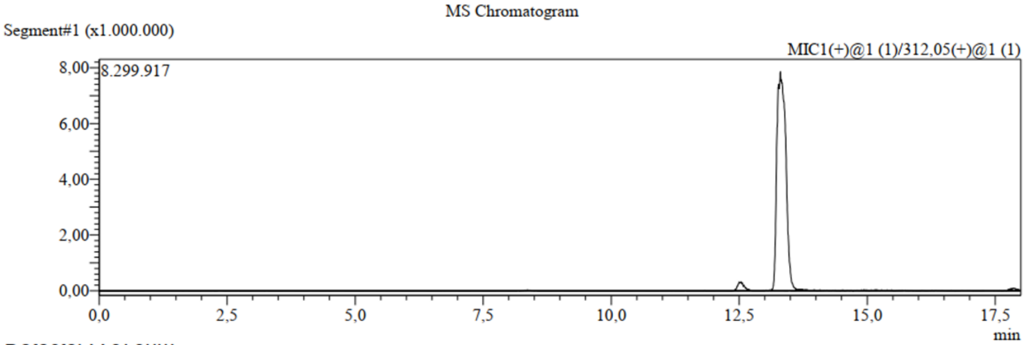

Peak#	Ret. Time	Area	Area%
1	12,281	37136	1,600
2	13,066	2284417	98,400
Total		2321553	100,000

<Chromatogram>

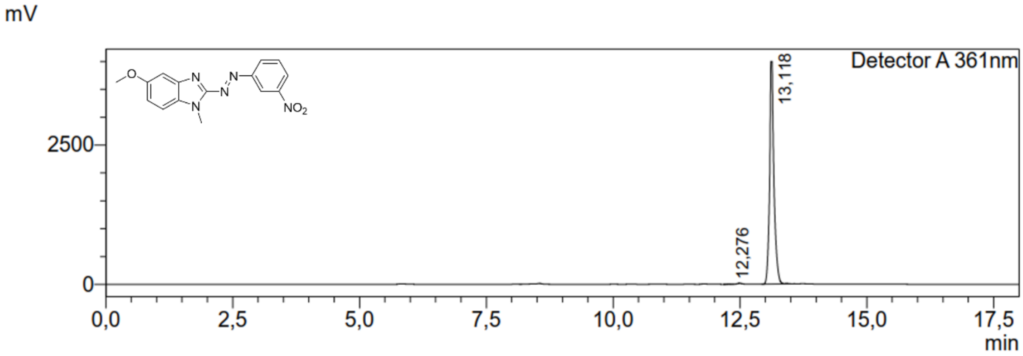

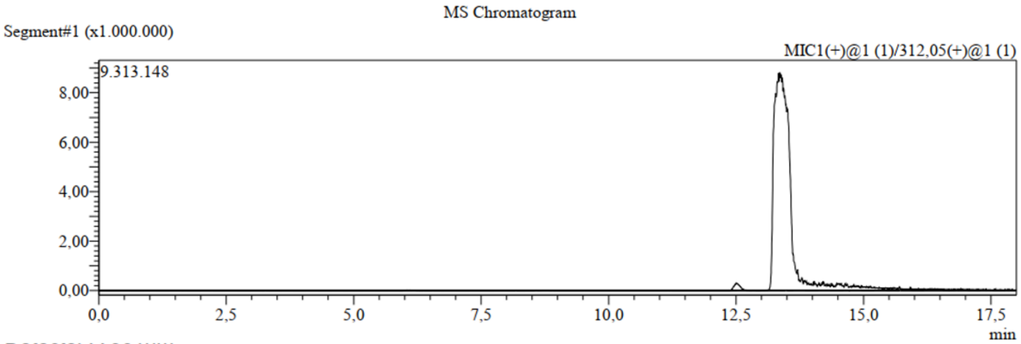

Peak#	Ret. Time	Area	Area%
1	12,276	32384	0,136
2	13,118	23734351	99,864
Total		23766735	100,000

<Chromatogram>

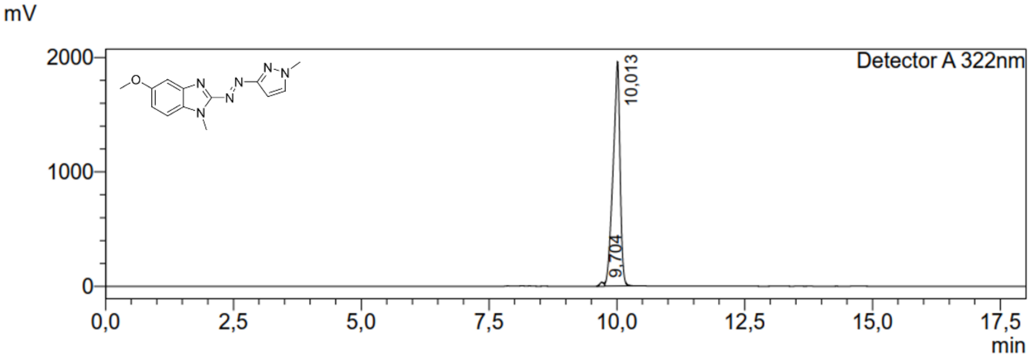

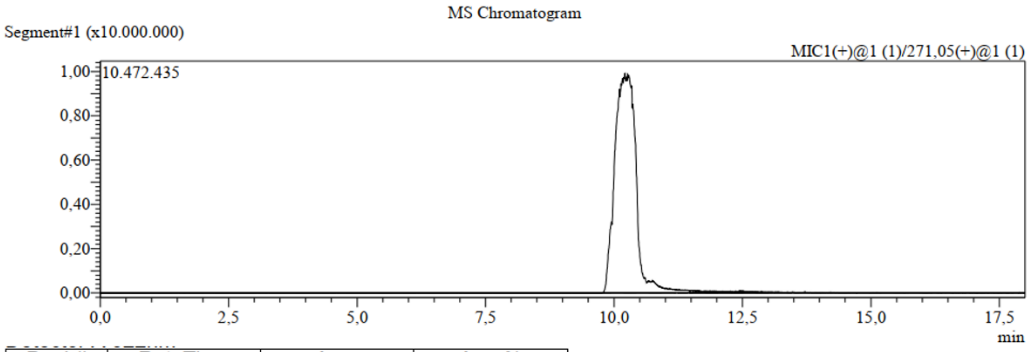

Peak#	Ret. Time	Area	Area%
1	9,704	207490	1,105
2	10,013	18576045	98,895
Total		18783535	100,000

<Chromatogram>

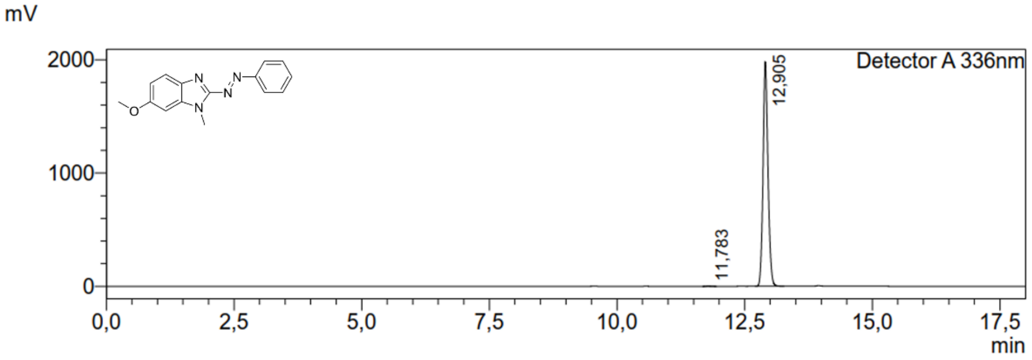

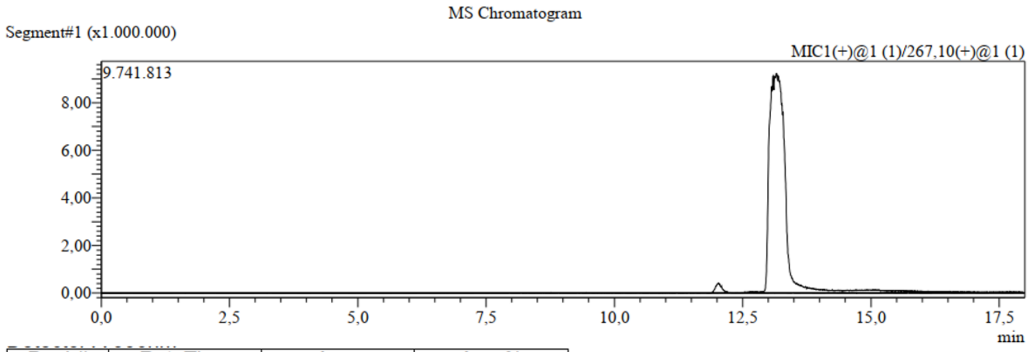

Peak#	Ret. Time	Area	Area%
1	11,783	20122	0,151
2	12,905	13284990	99,849
Total		13305112	100,000

<Chromatogram>

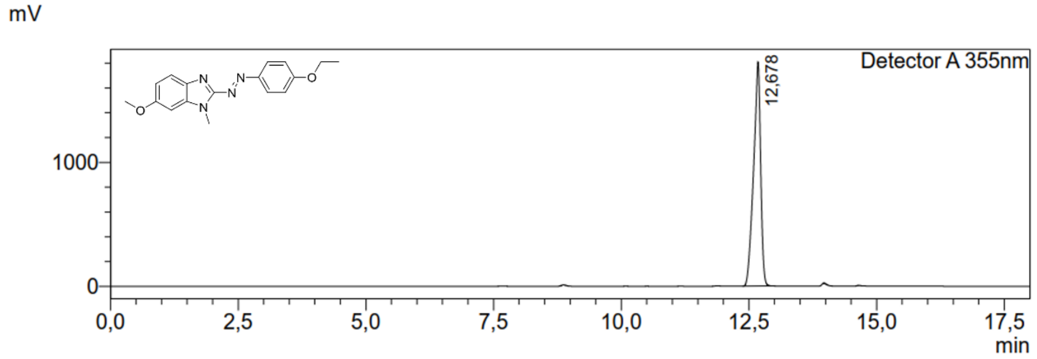

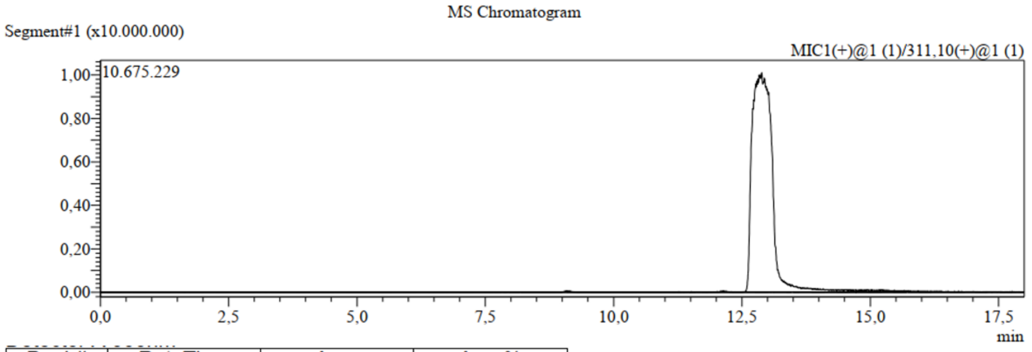

Peak#	Ret. Time	Area	Area%
1	12,678	17367160	100,000
Total		17367160	100,000

<Chromatogram>

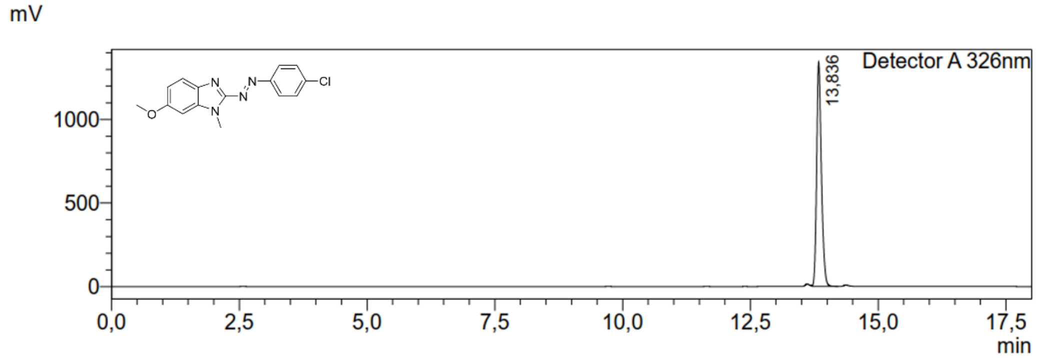

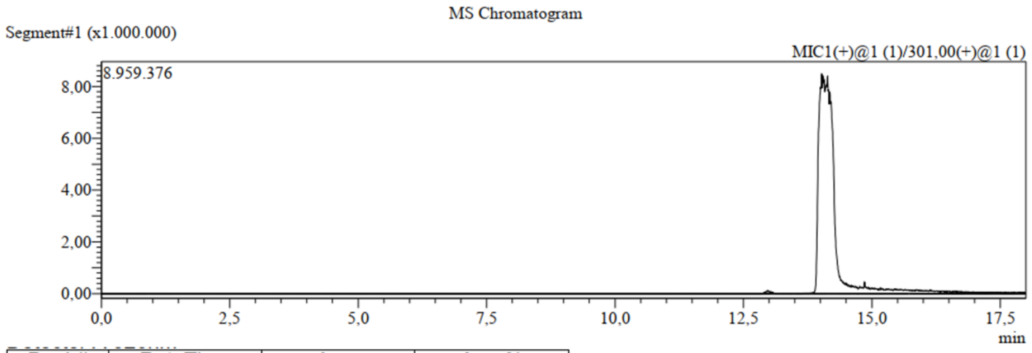

Peak#	Ret. Time	Area	Area%
1	13,836	8621767	100,000
Total		8621767	100,000

<Chromatogram>

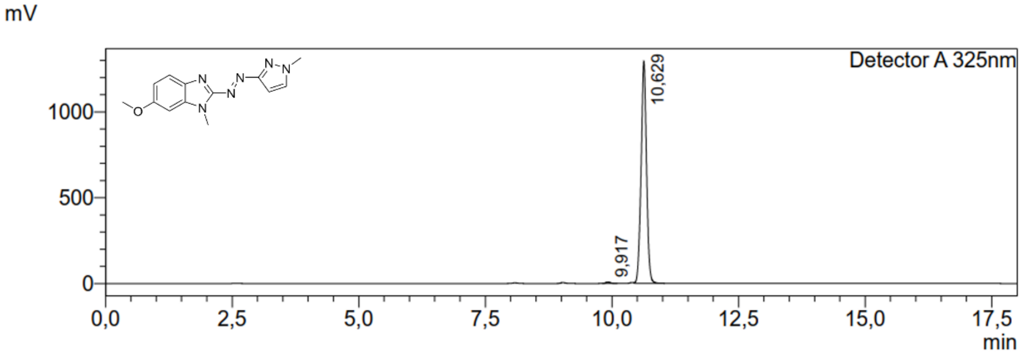

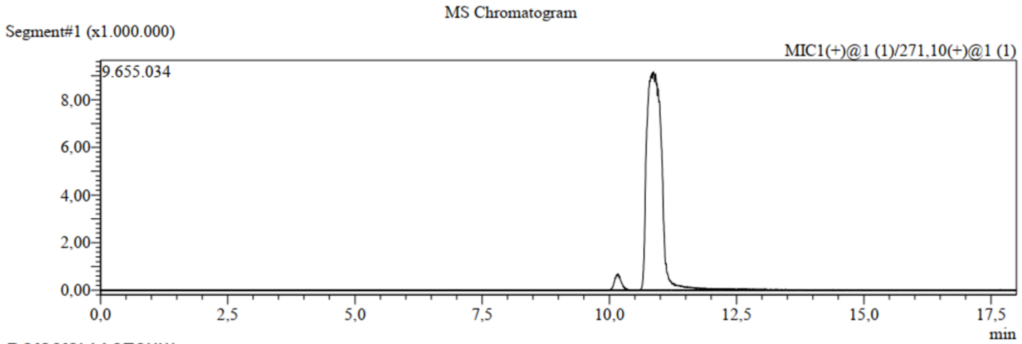

Peak#	Ret. Time	Area	Area%
1	9,917	49151	0,509
2	10,629	9599630	99,491
Total		9648782	100,000

<Chromatogram>

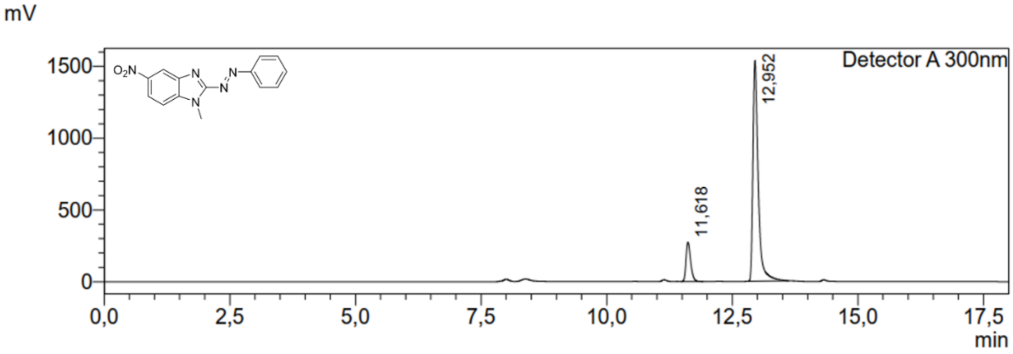

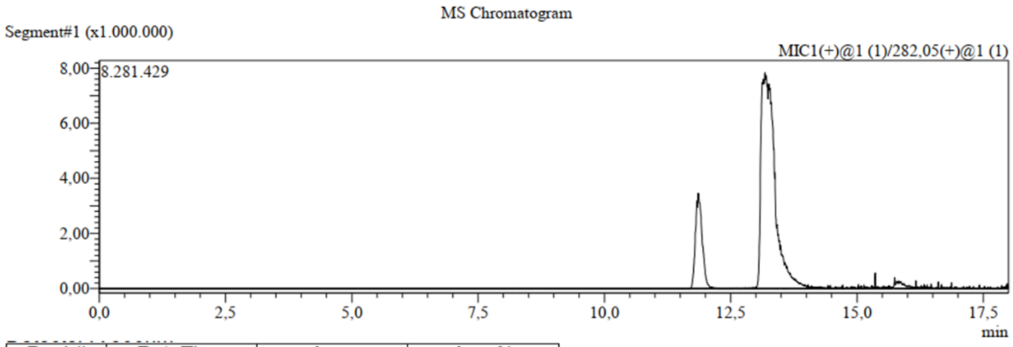

Peak#	Ret. Time	Area	Area%
1	11,618	1746595	13,486
2	12,952	11204592	86,514
Total		12951188	100,000

<Chromatogram>

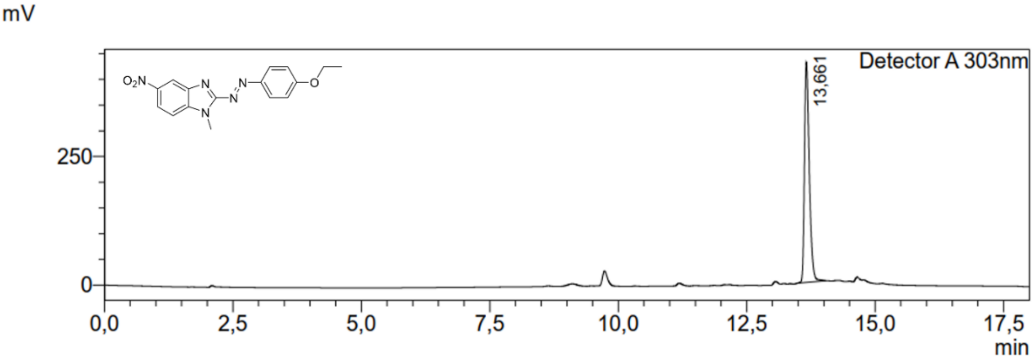

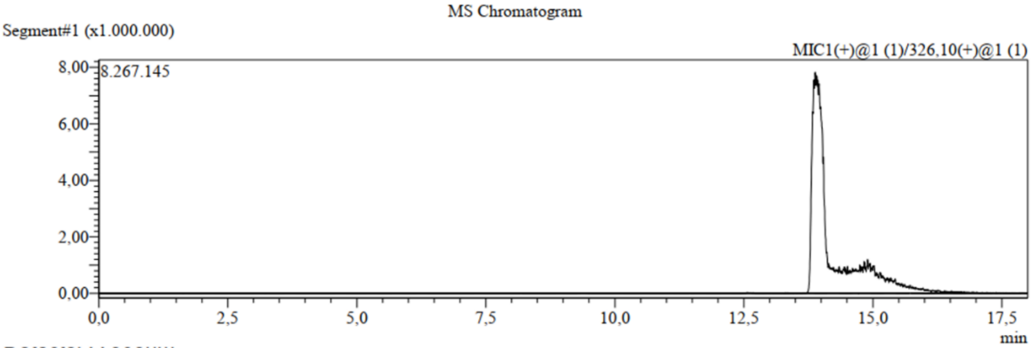

Peak#	Ret. Time	Area	Area%
1	13,661	2797942	100,000
Total		2797942	100,000

<Chromatogram>

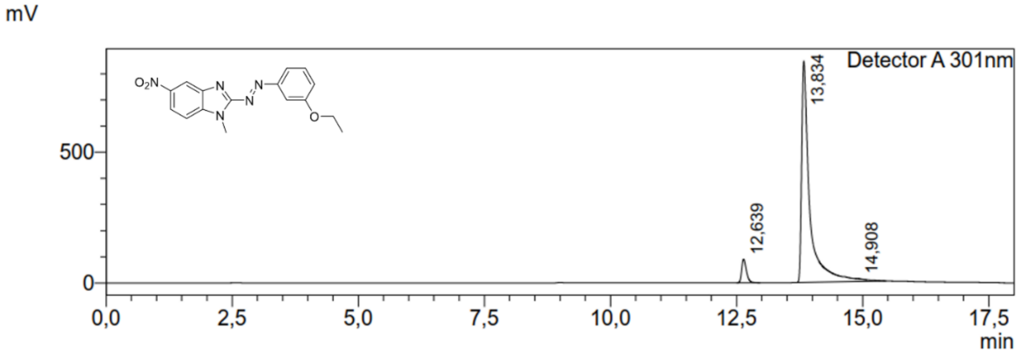

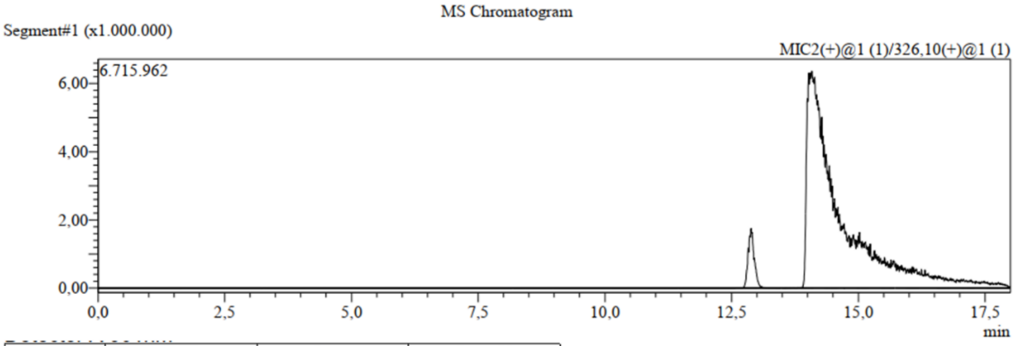

Peak#	Ret. Time	Area	Area%
1	12,639	581549	5,789
2	13,834	9453686	94,114
3	14,908	9683	0,096
Total		10044919	100,000

<Chromatogram>

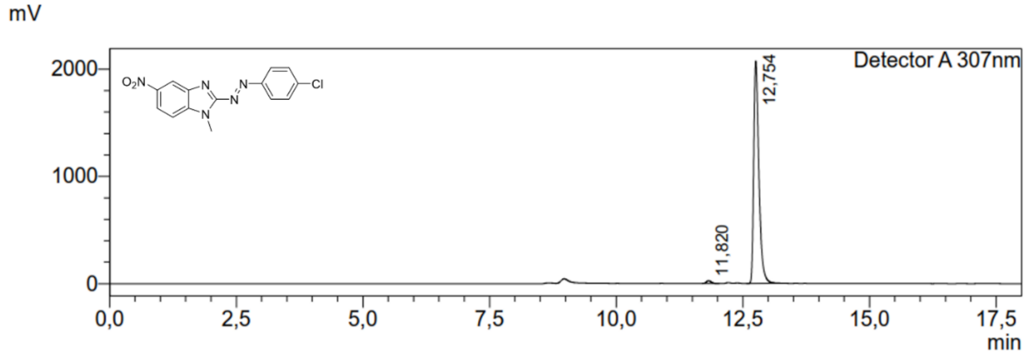

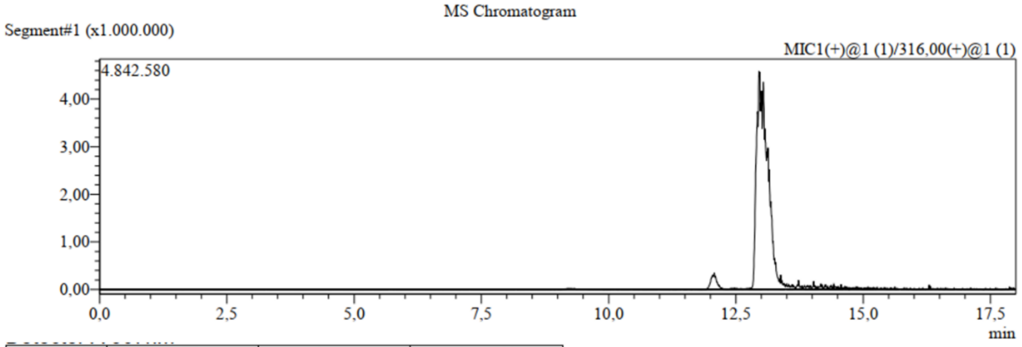

Peak#	Ret. Time	Area	Area%
1	11,820	165180	1,109
2	12,754	14731986	98,891
Total		14897167	100,000

<Chromatogram>

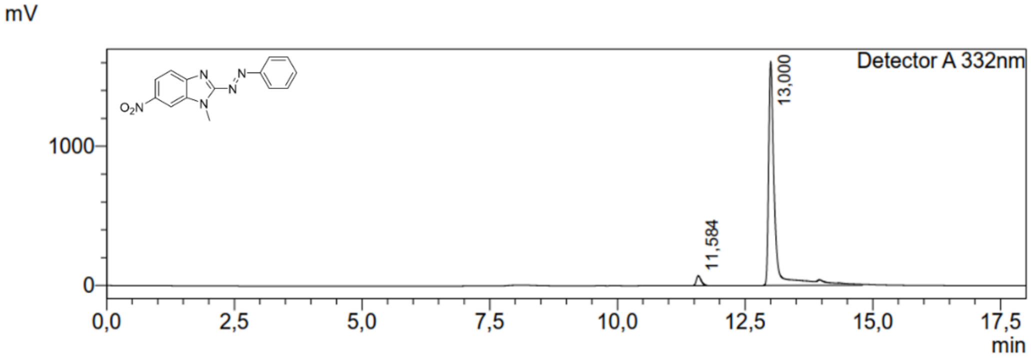

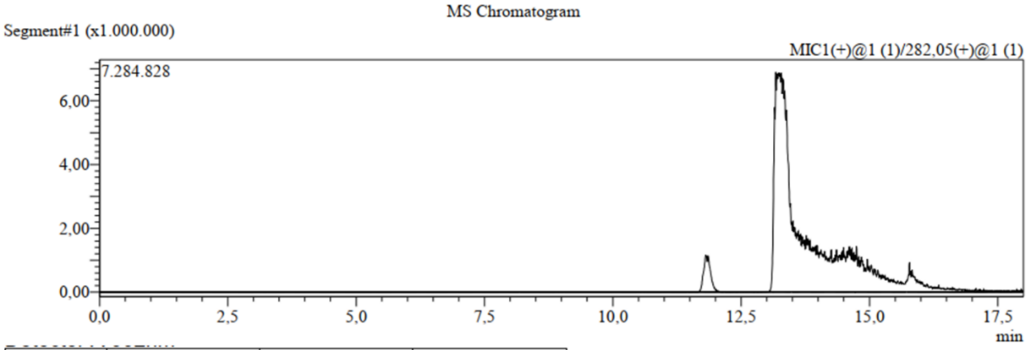

Peak#	Ret. Time	Area	Area%
1	11,584	477889	3,421
2	13,000	13490430	96,579
Total		13968319	100,000

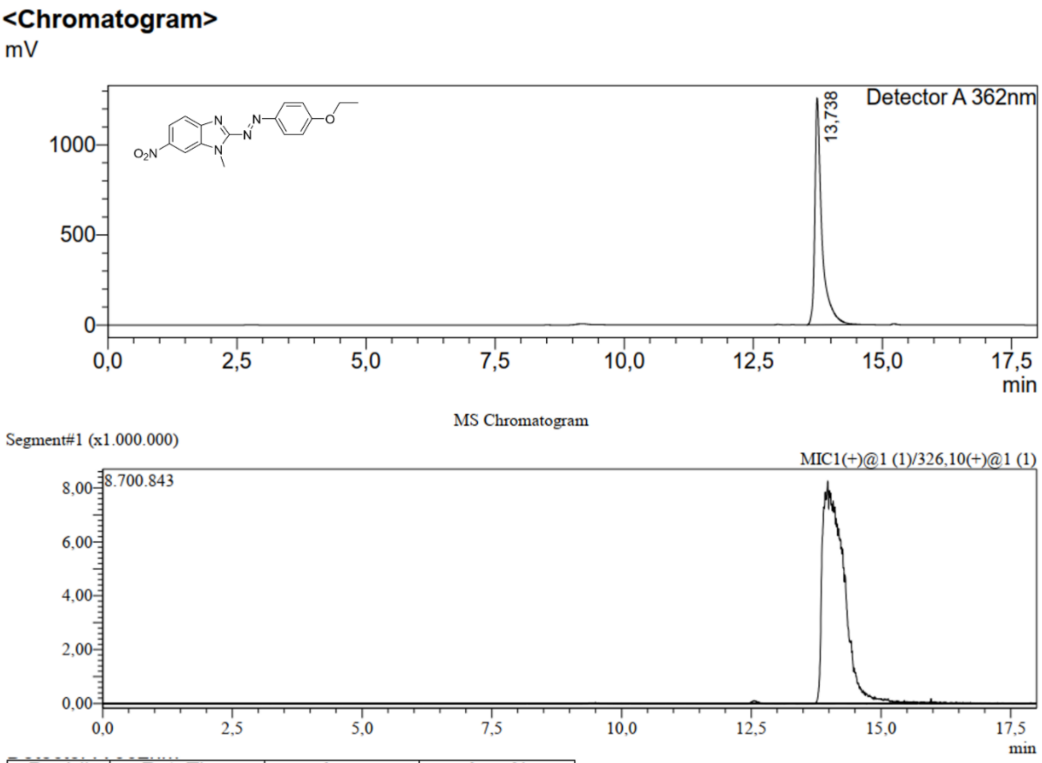

Peak#	Ret. Time	Area	Area%
1	13,738	12013717	100,000
Total		12013717	100,000

UV/Vis Spectra

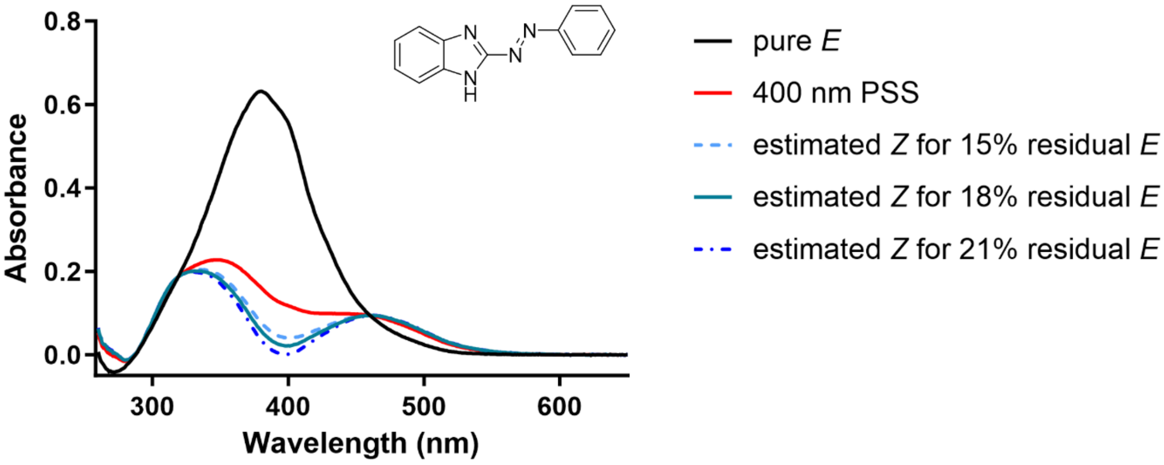

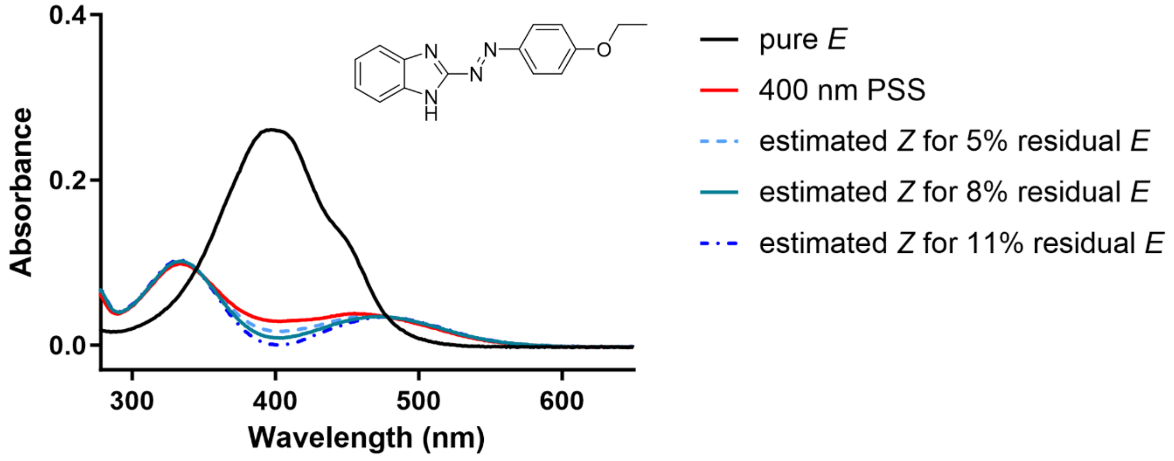

N
N
N
N
H
O
Absorbance
0.0
0.2
0.4
300
400
500
600
Wavelength (nm)
pure E
400 nm PSS
estimated Z for 5% residual E
estimated Z for 8% residual E
estimated Z for 11% residual E

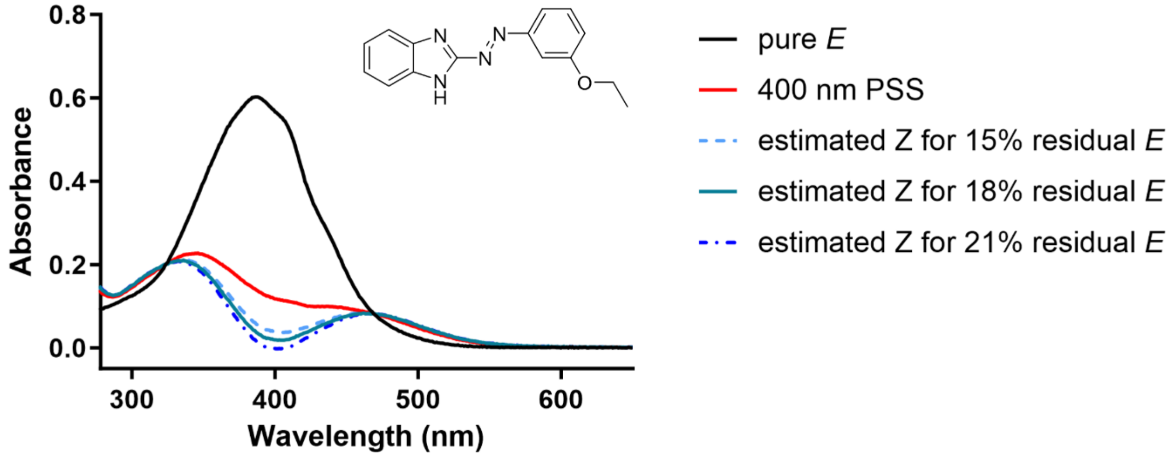

N
N
N
N
H
O
Absorbance
0.0
0.2
0.4
0.6
0.8
300
400
500
600
Wavelength (nm)
pure E
400 nm PSS
estimated Z for 15% residual E
estimated Z for 18% residual E
estimated Z for 21% residual E

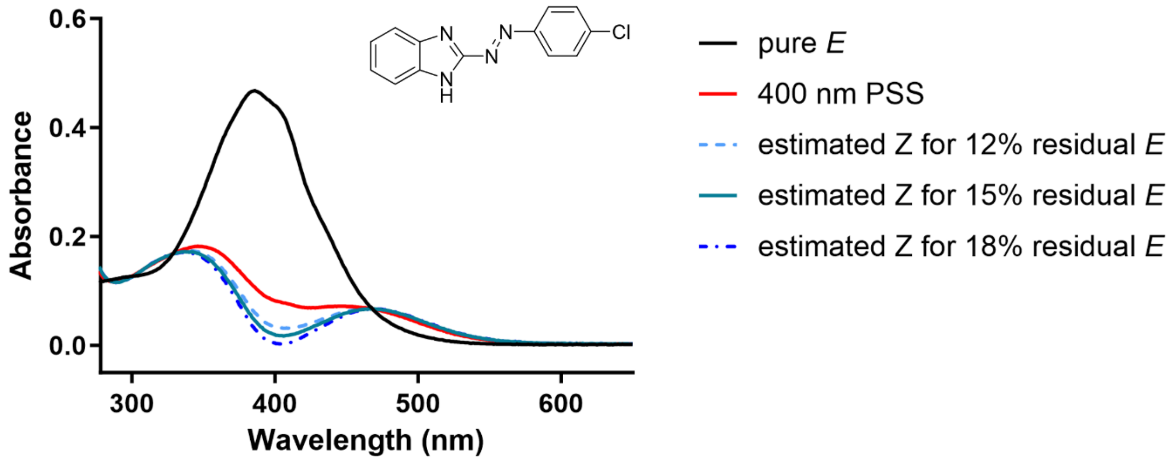

N
N
N
N
H
Cl
Absorbance
0.0
0.2
0.4
0.6
300
400
500
600
Wavelength (nm)
pure E
400 nm PSS
estimated Z for 12% residual E
estimated Z for 15% residual E
estimated Z for 18% residual E

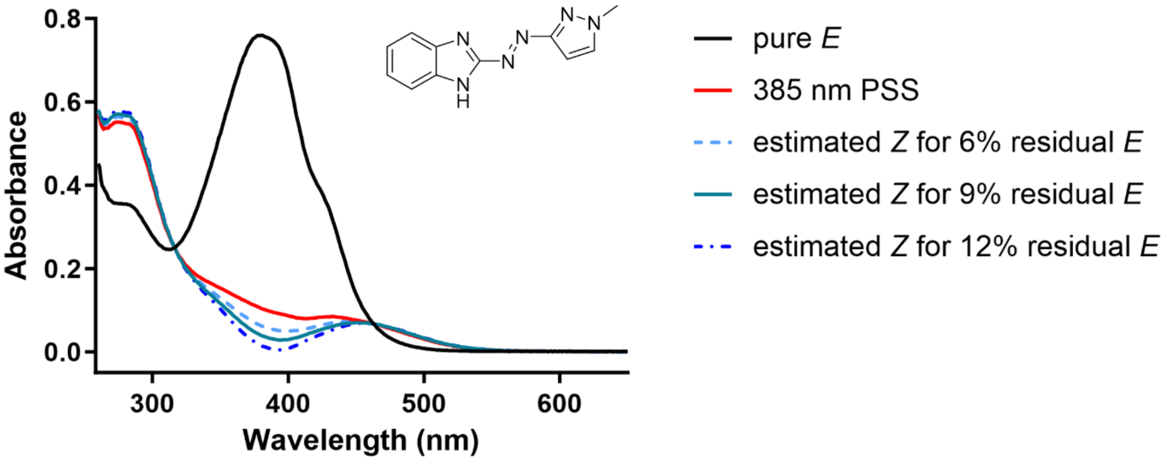

0.8
0.6
0.4
0.2
0.0
Absorbance
300
400
500
600
Wavelength (nm)
pure E
385 nm PSS
estimated Z for 6% residual E
estimated Z for 9% residual E
estimated Z for 12% residual E

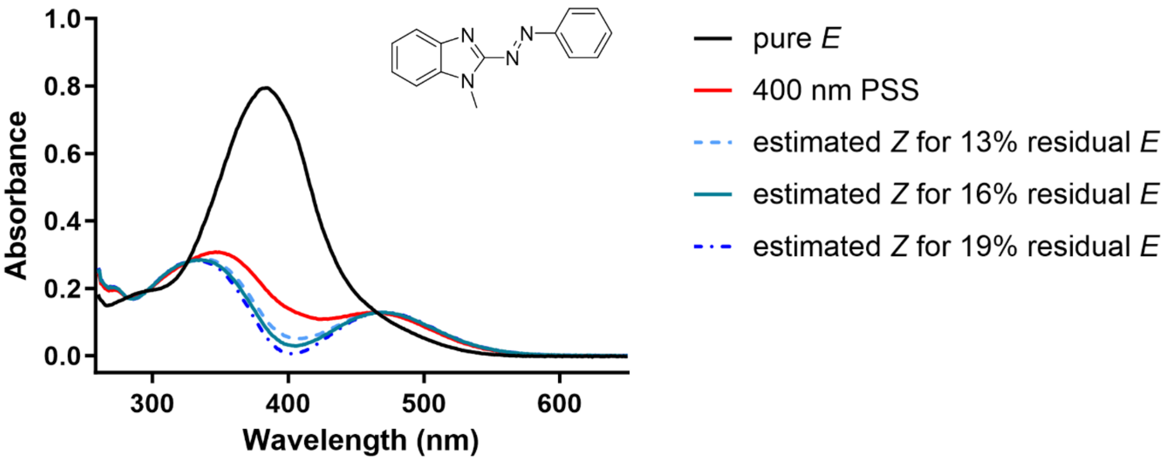

1.0
0.8
0.6
0.4
0.2
0.0
Absorbance
300
400
500
600
Wavelength (nm)
pure E
400 nm PSS
estimated Z for 13% residual E
estimated Z for 16% residual E
estimated Z for 19% residual E

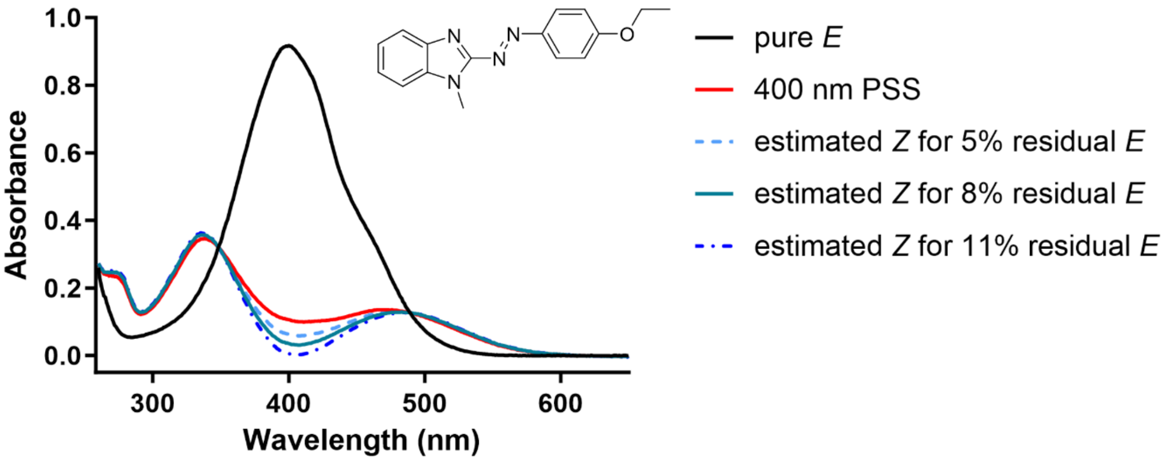

1.0
0.8
0.6
0.4
0.2
0.0
Absorbance
300
400
500
600
Wavelength (nm)
pure E
400 nm PSS
estimated Z for 5% residual E
estimated Z for 8% residual E
estimated Z for 11% residual E

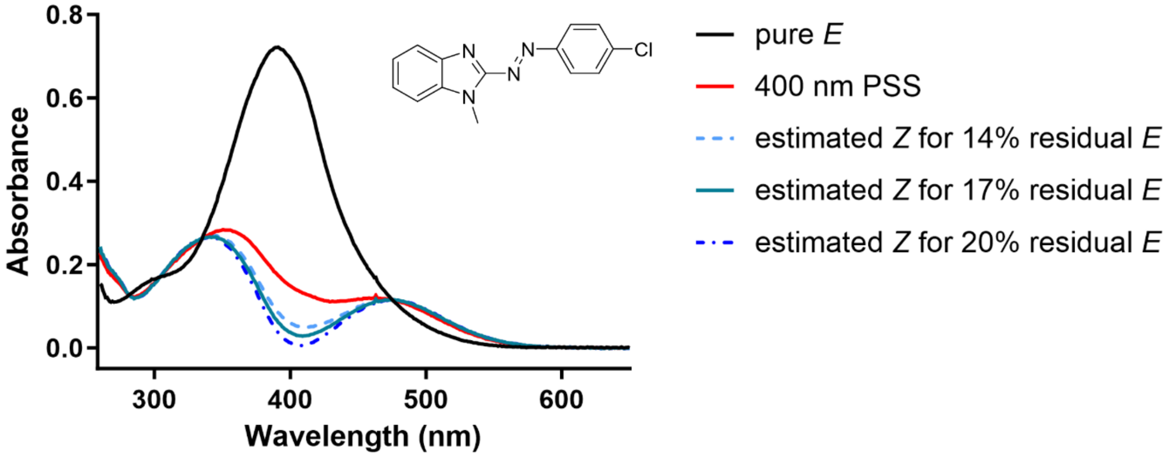
Absorbance
Wavelength (nm)
N
N
N
N
Cl
pure E
400 nm PSS
estimated Z for 14% residual E
estimated Z for 17% residual E
estimated Z for 20% residual E

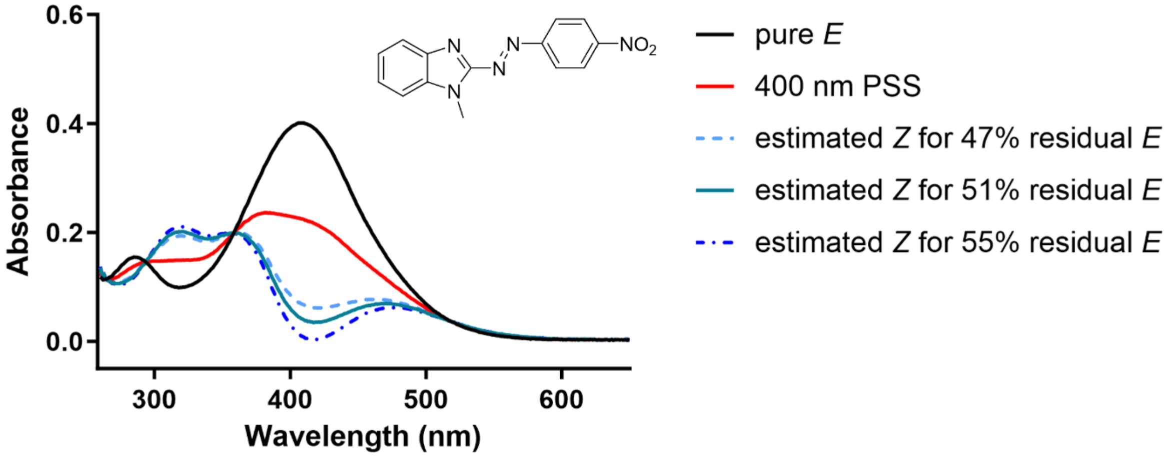
Absorbance
Wavelength (nm)
NO2
pure E
400 nm PSS
estimated Z for 47% residual E
estimated Z for 51% residual E
estimated Z for 55% residual E

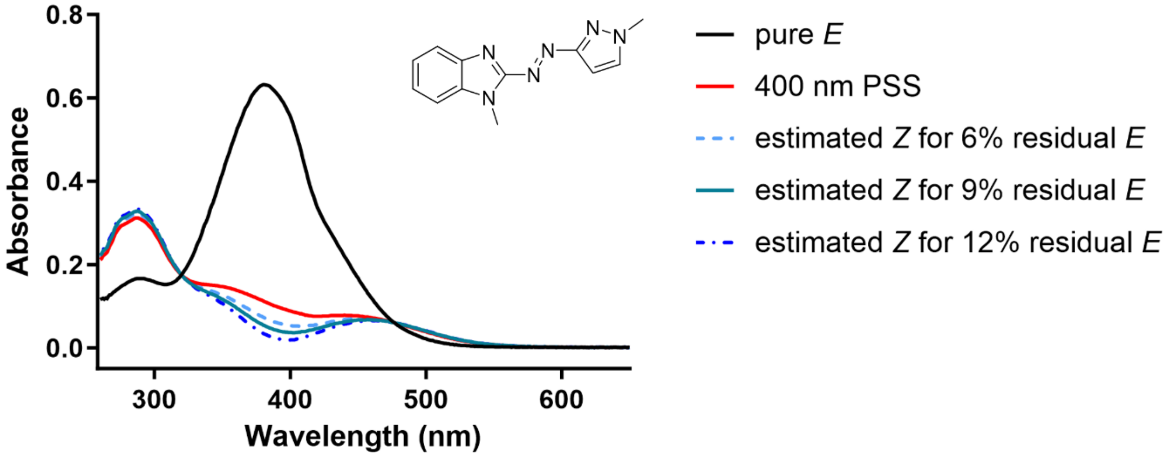
Absorbance
Wavelength (nm)
pure E
400 nm PSS
estimated Z for 6% residual E
estimated Z for 9% residual E
estimated Z for 12% residual E

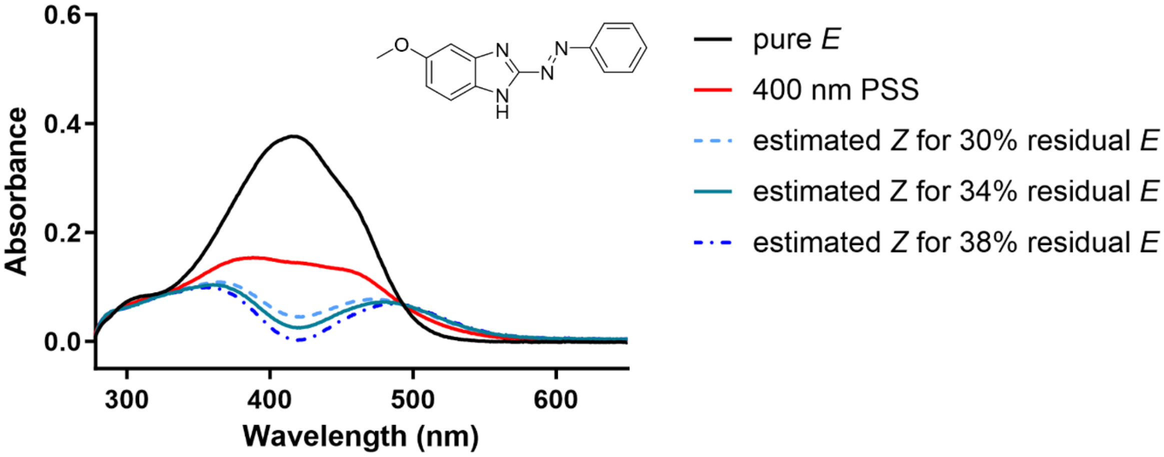

0.6
0.4
0.2
0.0
Absorbance
300
400
500
600
Wavelength (nm)
pure E
400 nm PSS
estimated Z for 30% residual E
estimated Z for 34% residual E
estimated Z for 38% residual E

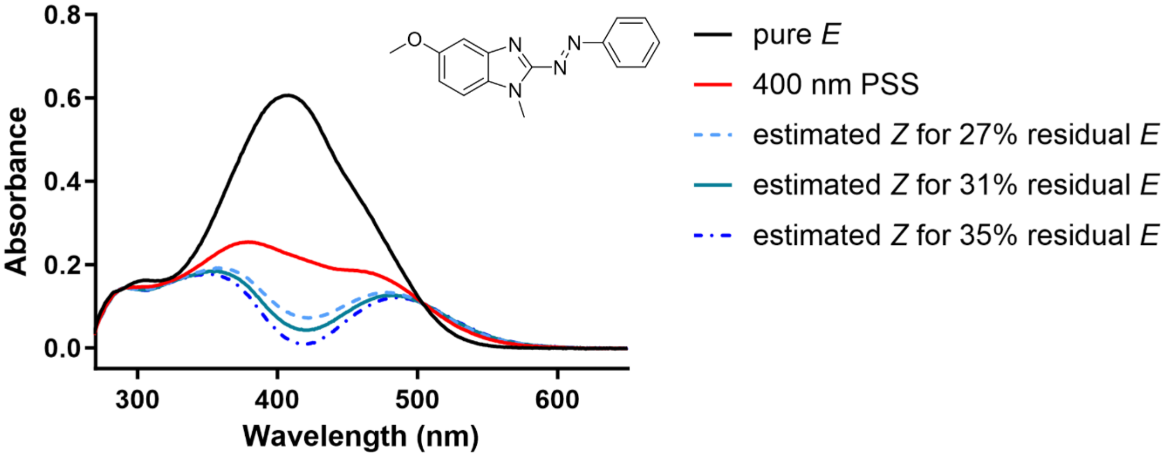

0.8
0.6
0.4
0.2
0.0
Absorbance
300
400
500
600
Wavelength (nm)
pure E
400 nm PSS
estimated Z for 27% residual E
estimated Z for 31% residual E
estimated Z for 35% residual E

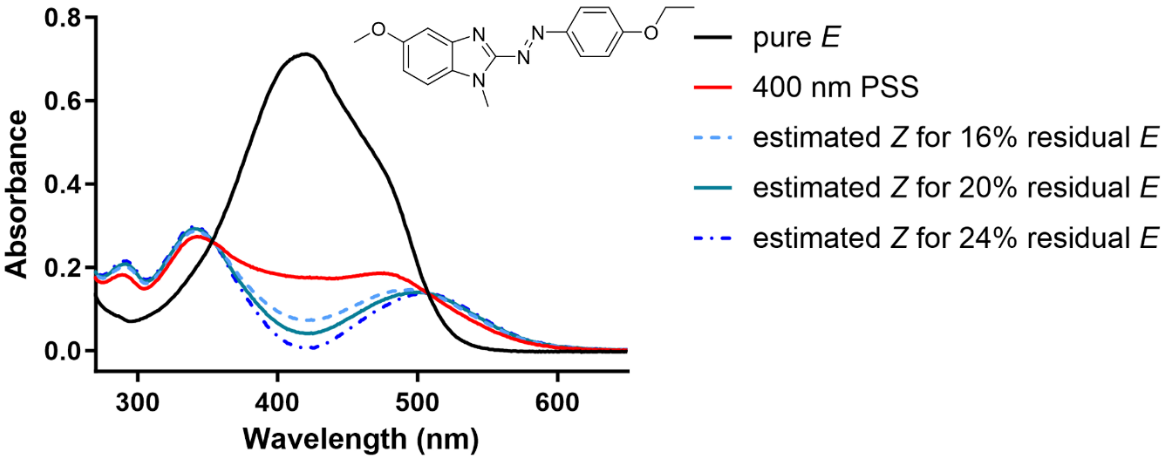

0.8
0.6
0.4
0.2
0.0
Absorbance
300
400
500
600
Wavelength (nm)
pure E
400 nm PSS
estimated Z for 16% residual E
estimated Z for 20% residual E
estimated Z for 24% residual E

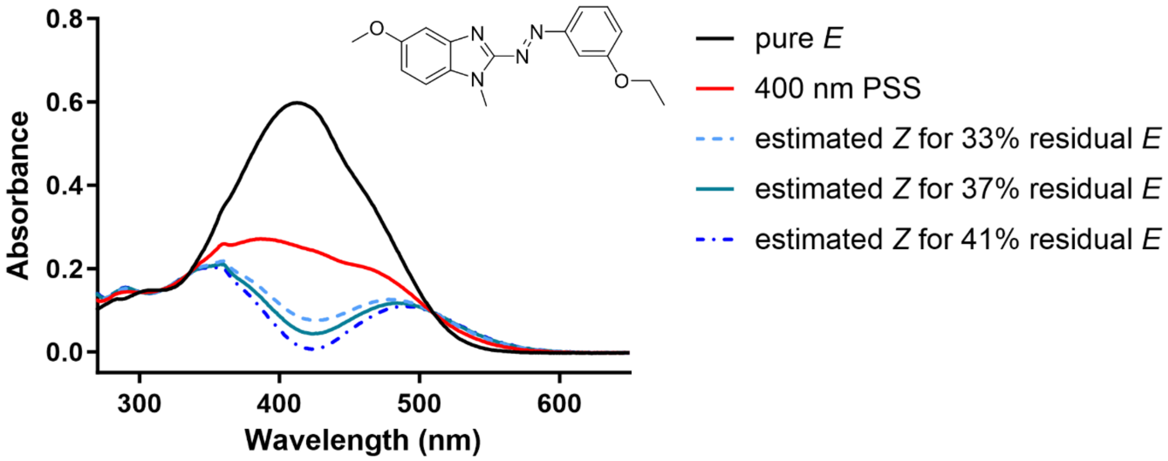
0.8
0.6
0.4
0.2
0.0
Absorbance
300
400
500
600
Wavelength (nm)
N
N
N
N
O
O
pure E
400 nm PSS
estimated Z for 33% residual E
estimated Z for 37% residual E
estimated Z for 41% residual E

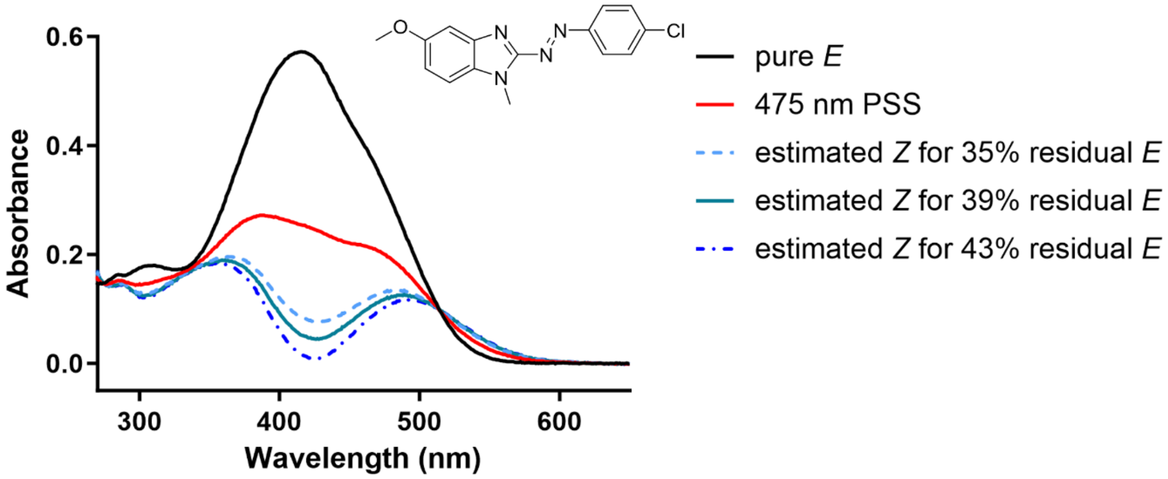
0.6
0.4
0.2
0.0
Absorbance
300
400
500
600
Wavelength (nm)
O
N
N
N
N
Cl
pure E
475 nm PSS
estimated Z for 35% residual E
estimated Z for 39% residual E
estimated Z for 43% residual E

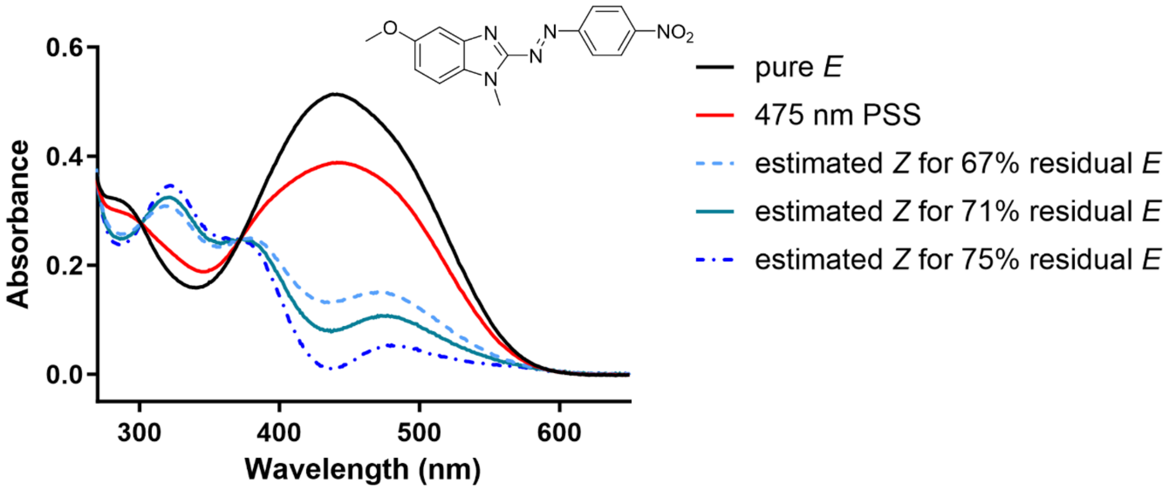
0.6
0.4
0.2
0.0
Absorbance
300
400
500
600
Wavelength (nm)
O
N
N
N
N
NO2
pure E
475 nm PSS
estimated Z for 67% residual E
estimated Z for 71% residual E
estimated Z for 75% residual E

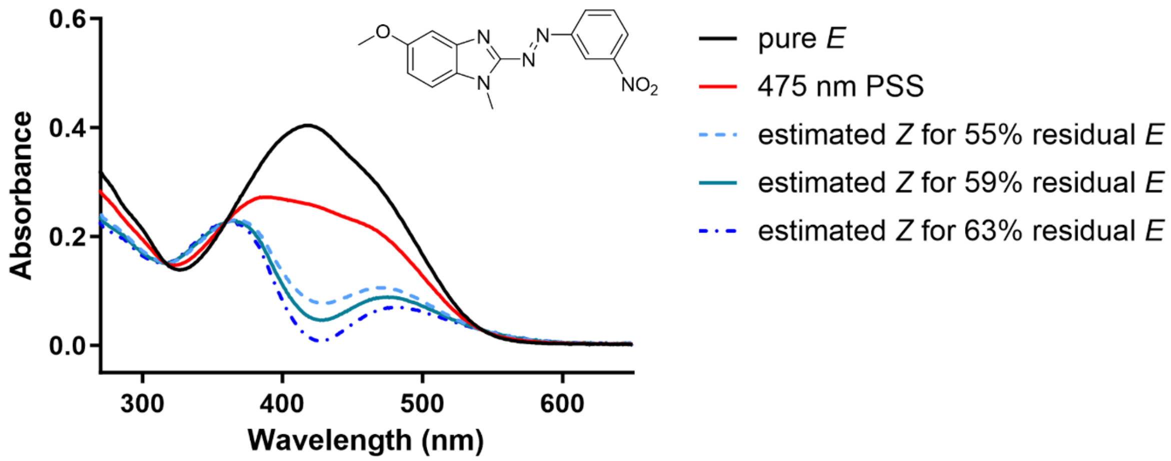
0.6
0.4
0.2
0.0
Absorbance
300
400
500
600
Wavelength (nm)
O
N
N
N
N
NO2
pure E
475 nm PSS
estimated Z for 55% residual E
estimated Z for 59% residual E
estimated Z for 63% residual E

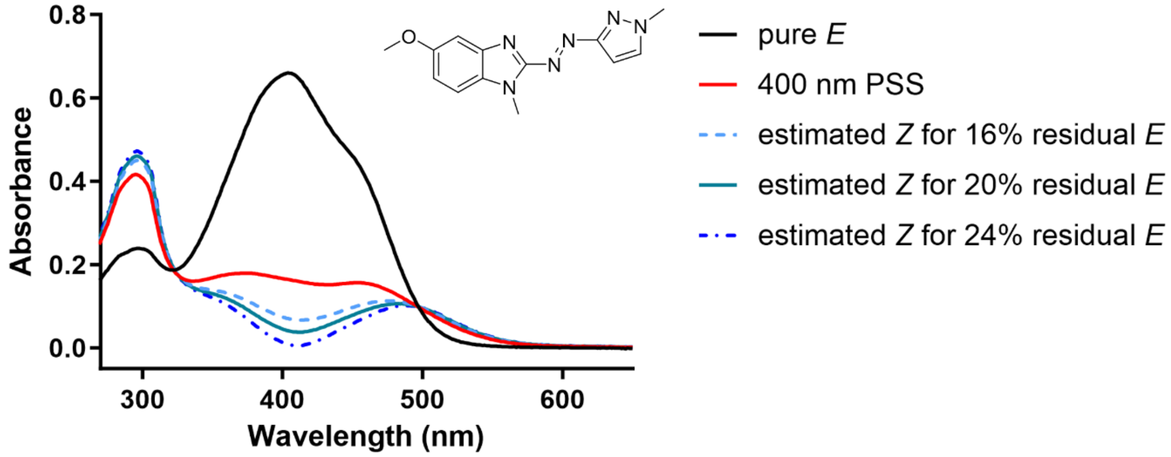
0.8
0.6
0.4
0.2
0.0
Absorbance
300
400
500
600
Wavelength (nm)
O
N
N
N
N
N
N
pure E
400 nm PSS
estimated Z for 16% residual E
estimated Z for 20% residual E
estimated Z for 24% residual E

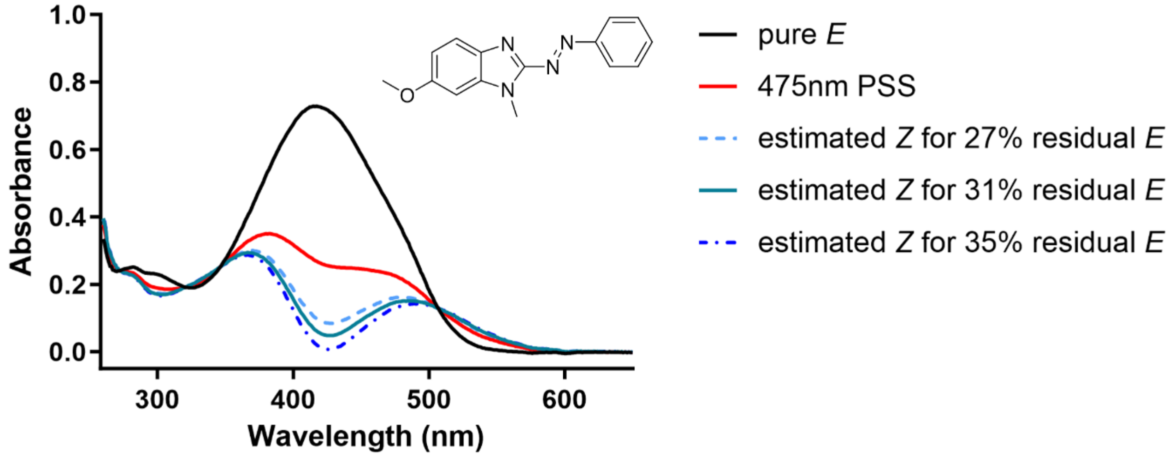
1.0
0.8
0.6
0.4
0.2
0.0
Absorbance
300
400
500
600
Wavelength (nm)
O
N
N
N
N
pure E
475nm PSS
estimated Z for 27% residual E
estimated Z for 31% residual E
estimated Z for 35% residual E

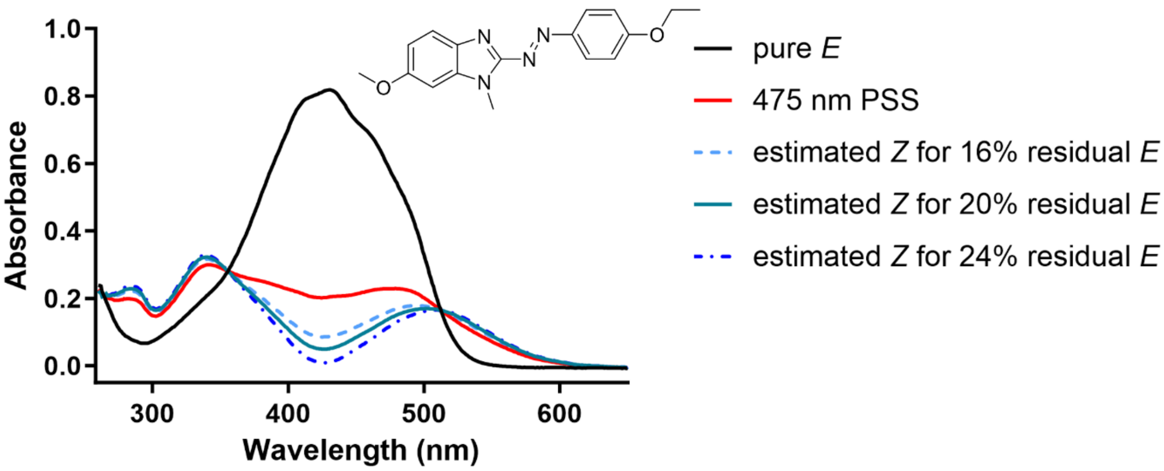
Absorbance
Wavelength (nm)
pure E
475 nm PSS
estimated Z for 16% residual E
estimated Z for 20% residual E
estimated Z for 24% residual E

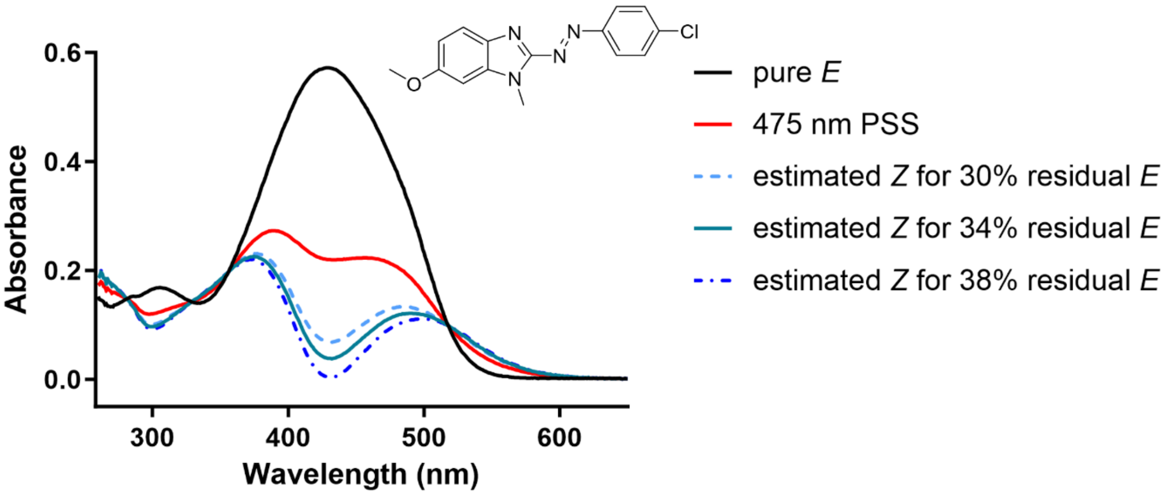
Absorbance
Wavelength (nm)
pure E
475 nm PSS
estimated Z for 30% residual E
estimated Z for 34% residual E
estimated Z for 38% residual E

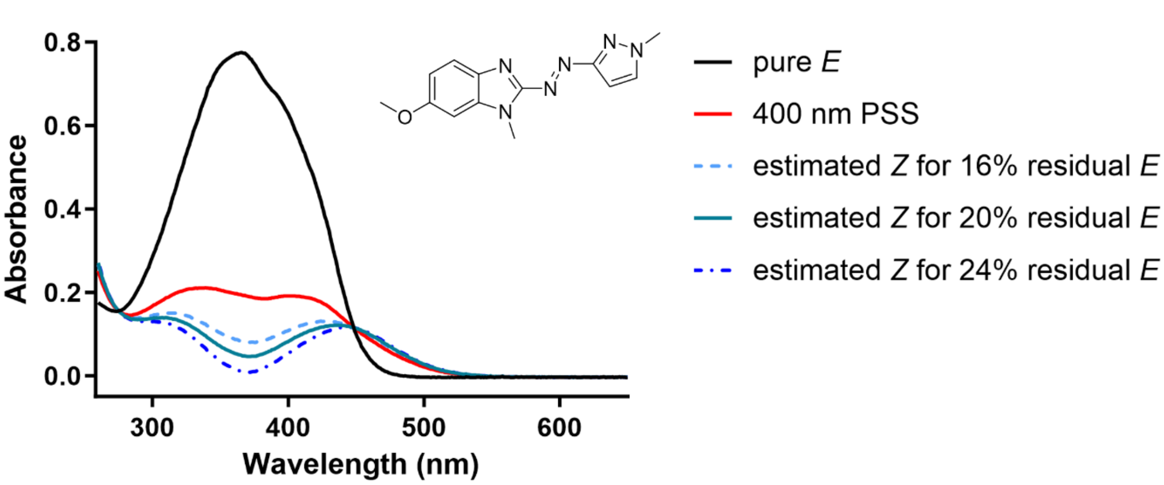
Absorbance
Wavelength (nm)
pure E
400 nm PSS
estimated Z for 16% residual E
estimated Z for 20% residual E
estimated Z for 24% residual E

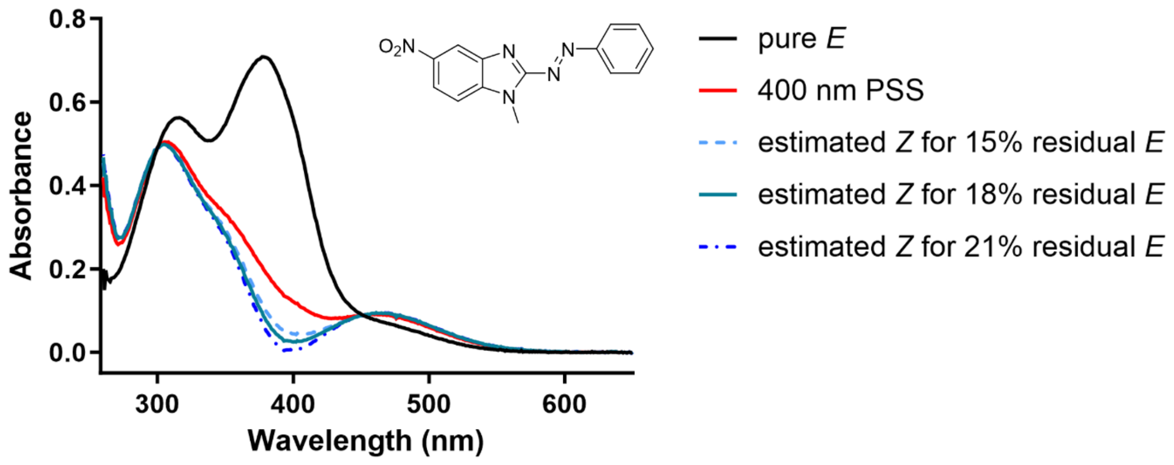

0.8
0.6
0.4
0.2
0.0
Absorbance
300
400
500
600
Wavelength (nm)
O_2N
N
N
N
N
pure E
400 nm PSS
estimated Z for 15% residual E
estimated Z for 18% residual E
estimated Z for 21% residual E

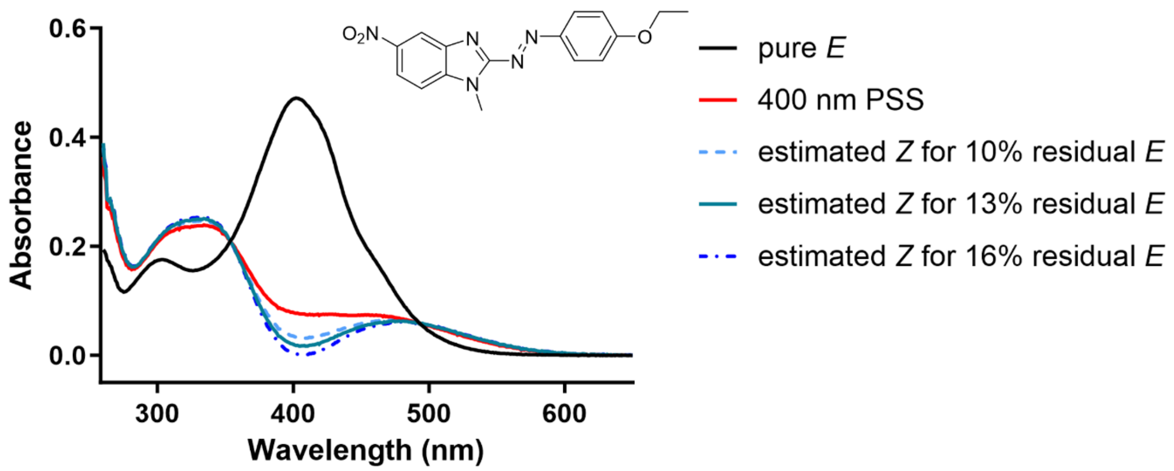

0.6
0.4
0.2
0.0
Absorbance
300
400
500
600
Wavelength (nm)
O_2N
N
N
N
N
O
pure E
400 nm PSS
estimated Z for 10% residual E
estimated Z for 13% residual E
estimated Z for 16% residual E

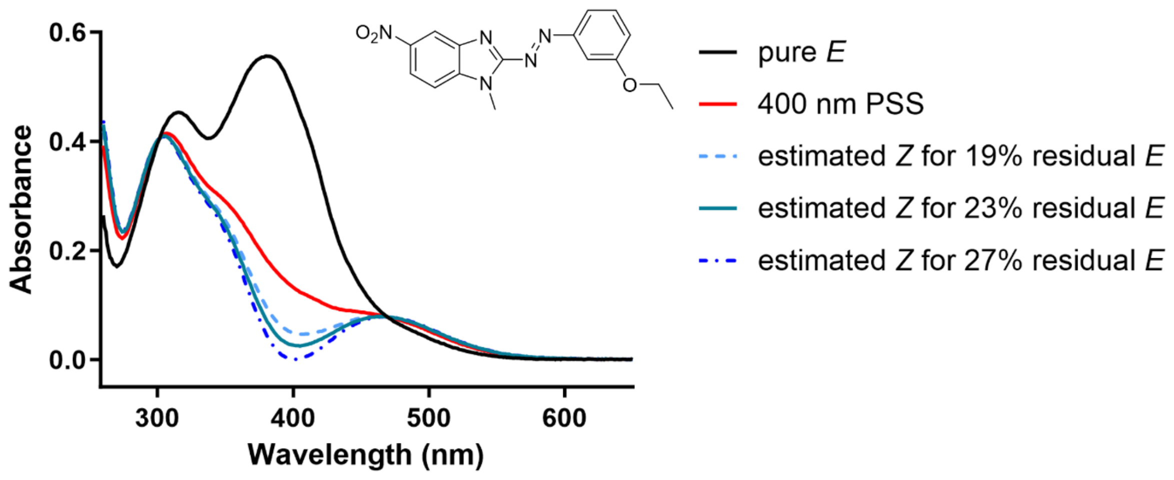

0.6
0.4
0.2
0.0
Absorbance
300
400
500
600
Wavelength (nm)
O_2N
N
N
N
N
O
pure E
400 nm PSS
estimated Z for 19% residual E
estimated Z for 23% residual E
estimated Z for 27% residual E

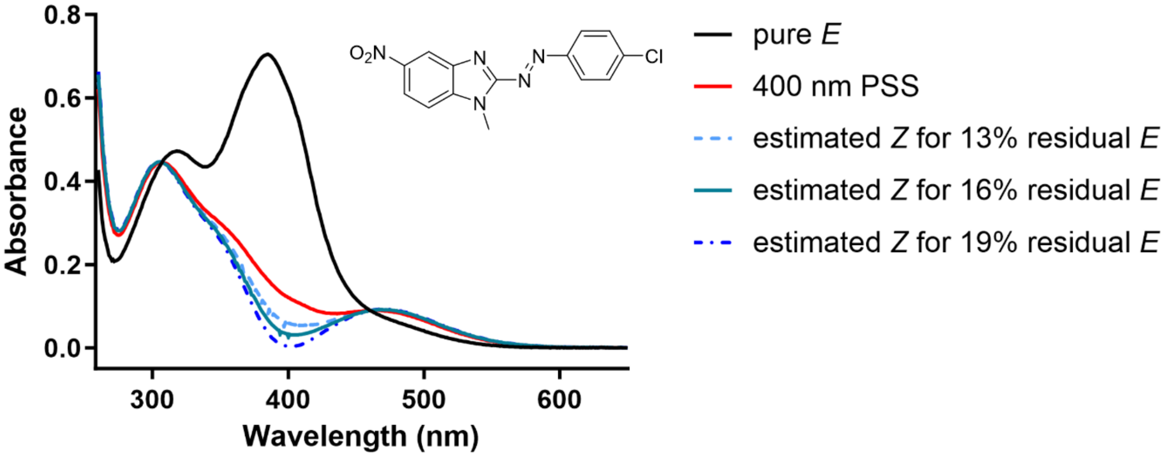

0.8
0.6
0.4
0.2
0.0
Absorbance
300
400
500
600
Wavelength (nm)
O2N
N
N
N
N
Cl
pure E
400 nm PSS
estimated Z for 13% residual E
estimated Z for 16% residual E
estimated Z for 19% residual E

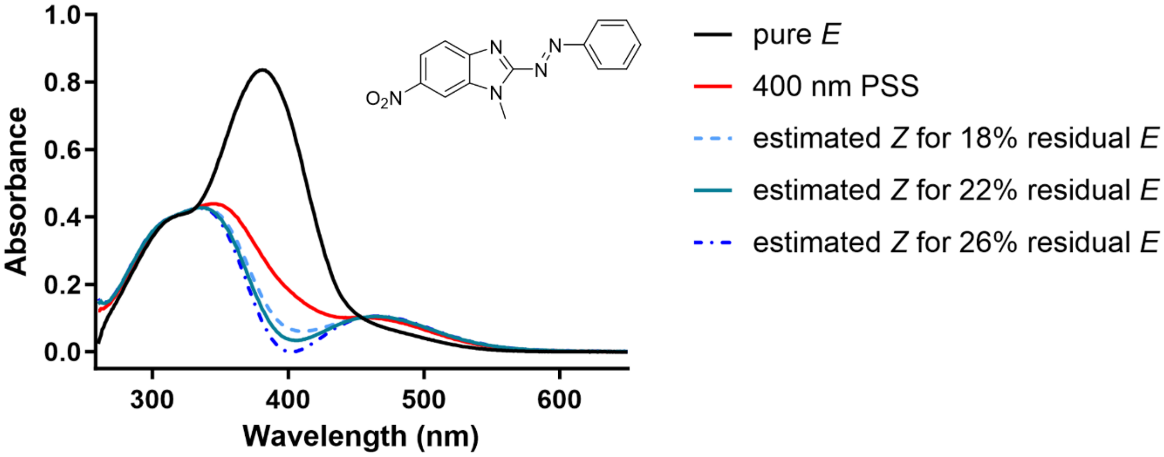

1.0
0.8
0.6
0.4
0.2
0.0
Absorbance
300
400
500
600
Wavelength (nm)
O2N
N
N
N
N
pure E
400 nm PSS
estimated Z for 18% residual E
estimated Z for 22% residual E
estimated Z for 26% residual E

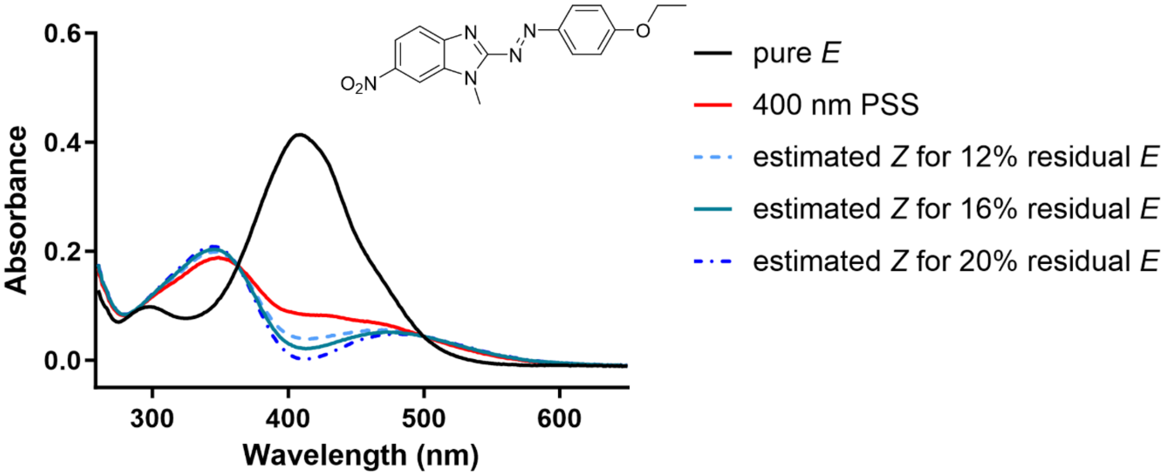

0.6
0.4
0.2
0.0
Absorbance
300
400
500
600
Wavelength (nm)
O2N
N
N
N
N
O
pure E
400 nm PSS
estimated Z for 12% residual E
estimated Z for 16% residual E
estimated Z for 20% residual E

Long-Term Stability

Additionally to investigating photo-fatigue, compounds **3pz** and **8a** were chosen for evaluating stability towards long-term light exposure using both an *N*-methylated arylazobenzimidazole derivative and a derivative with a free benzimidazole-NH.
Continuous irradiation was carried out with the respective wavelength (used for switching to the *Z*-isomer) for 30 mins. Absorption was measured every 5 mins. No noticeable photo-fatigue was observed, and the compound could be switched back to the *E*-isomer using the respective wavelength.

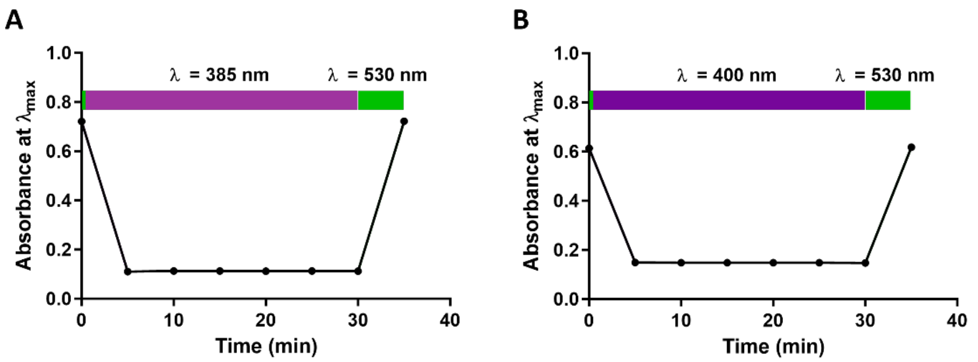

Figure S 29*. UV/vis stability measurement. Pre-irradiation with the E-isomer wavelength (λ = 530 nm) for the first data point, followed by continuous irradiation for 30 mins with the respective Z-isomer wavelength λ = 385 or 400 nm. The absorption remains stable for the duration of irradiation and switching back to the E-isomer results in similar absorption compared to the data point prior to continuous irradiation.*

To further monitor potential photodegradation, LC/MS was used as the analytical method. The respective LC trace was measured at 254 nm to determine potential impurities/degradation products. For continuous irradiation of **8a** at 400nm over 60 mins, no compound degradation was observed. The HPLC traces were comparable at prior to and after 60 mins irradiation with a purity of **8a** over 98 % (the injection peak at 2 mins results from injecting a DMSO sample and was not considered for determination of purity).

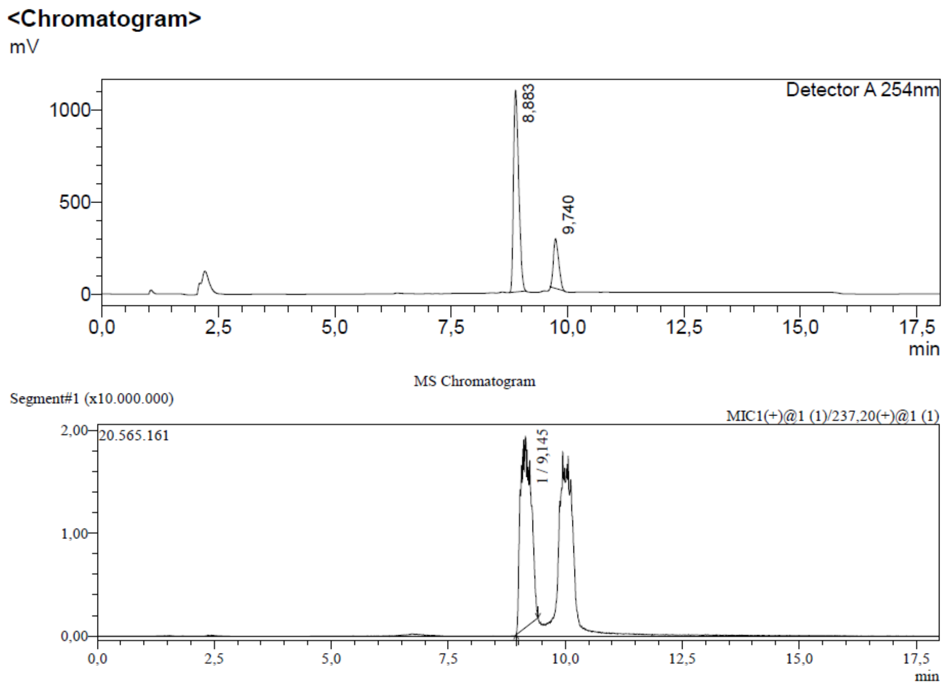

*Figure S 30. LC/MS run of compound **8a** after irradiation with λ = 400 nm for 60 mins shows both the E- and Z-isomer peaks as well as an injection peak (DMSO). No impurity peaks have appeared which verifies stability of the compound and resistance to photo-degradation. Shown is the MIC to verify the compound mass.*

For compound **3pz**, continuous irradiation was carried out at 385nm over 70 mins, which did not result in significant photo-degradation. While there was a small (<2%) impurity in the sample, the amount of this impurity does not significantly change after irradiation for 70 mins. Even if small changes in the impurity level are considered (~1-1.5 % during >1 h of continuous irradiation at 385nm), such a duration of irradiation is over 120-times longer than the irradiation time required for switching. We therefore conclude that these two representative compounds show excellent stability in terms of photodegradation.

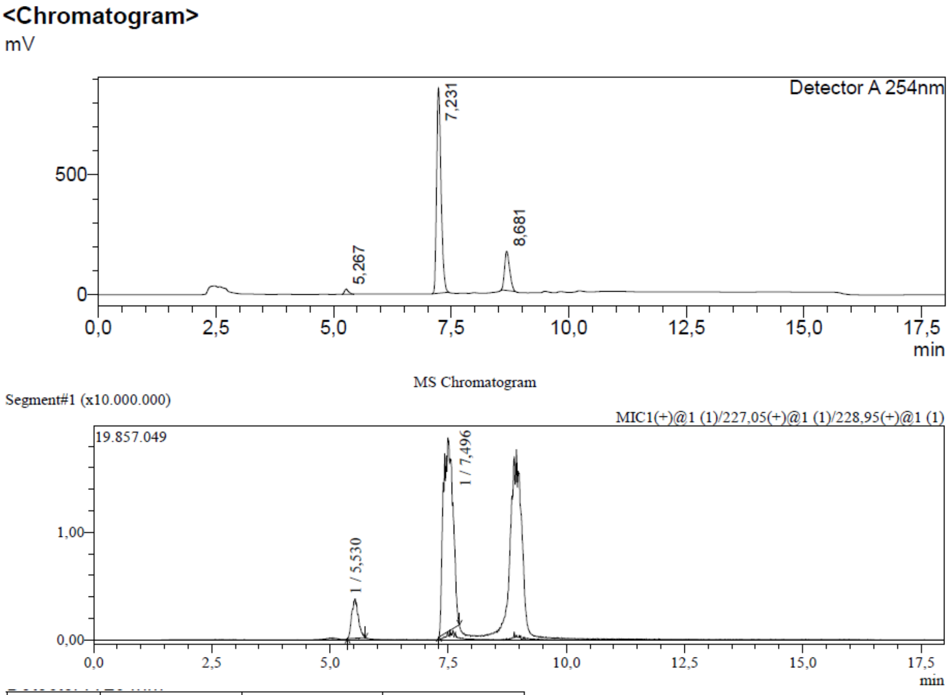

Peak#	Ret. Time	Area	Area%
1	5,267	123285	1,770
2	7,231	5615796	80,625
3	8,681	1226266	17,605
Total		6965347	100,000

*Figure S 31. LC/MS run of compound **3pz** after irradiation with λ = 385 nm for 70 mins shows both the E- and Z-isomer peaks as well as an injection peak and the small impurity at 5.27 mins. Compound **3pz** is still >98 % pure after 70 mins irradiation.*

Quantum Yield Measurements

The photoisomerization quantum yields of **3pz** and **8a** were calculated for irradiation at 365nm. The measurements were performed in dry acetonitrile and at ambient temperature (T = 22°C) to offer a direct comparison with other reported photoswitches. Spectra were recorded with an Agilent Cary60 UV/vis spectrophotometer equipped with a temperature controller and using a standard quartz cuvette (1 cm path length).

Photon Flux Determination

The photon flux of a Nichia NCSU276A LED (365 nm, 800 mW when operating at 100% power) fitted with a collimating lens was determined by ferrioxalate actinometry. The method follows a previously reported procedure and is based on the photochemical degradation of ferrioxalate to Fe^{2+}:[2, 3]

$$Fe(C_2O_4)_3{}^{3-}\, h\nu \xrightarrow{h\nu} Fe^{2+} C_2O_4{}^{\cdot -} + 2C_2O_4{}^{2-}$$

$$Fe(C_2O_4)_3{}^{3-} + C_2O_4{}^{\cdot -} \xrightarrow{\Delta} Fe^{2+} + 2CO_2 + 3C_2O_4{}^{2-}$$

A known volume of 30 mM potassium ferrioxalate in 0.2 N H_2SO_4 (V_1 = 2 ml) was irradiated under stirring in a quartz cuvette placed 3.5 cm away from the light source. After different irradiation times, an aliquot of the solution (V_2 = 0.5 ml) was mixed with 1 mL buffer (1.2 M NaOAc + 0.72 N H_2SO_4) and 2 mL 1,10-phenanthroline (6 mM) and finally diluted to 25 ml (V_3) with mQ H_2O. The formation of the tris-phenanthroline complex was measured after 1 h by recording the absorbance at 510 nm.

The absorbance data was then plotted as a function of the irradiation time and the corresponding slope was used to determine the photon flux according to the linear equation provided by Stranius and Börjesson:[3]

$$I = slope\ \frac{V_1 \cdot V_3 \cdot N_a}{V_2 \cdot \varepsilon_{510nm} \cdot l \cdot \varphi}$$

Where I is the photon flux, φ is the quantum yield of the ferrioxalate degradation at 365 nm (φ = 1.21), l is the path length of the cuvette in cm, ε_{510nm} is the molar extinction coefficient of the iron (II) tris-phenanthroline complex at 510 nm (ε_{510nm}=11,100 M^{-1} cm^{-1}), N_a is Avogadro's number, and V_1, V_2 and V_3 are the volumes of the different solutions in dm^3 to take into account dilution factors.

Using this method, the photon flux of the 365 nm LED operating at 5% power was determined to be 2.117 × 10^{16} photons/s.

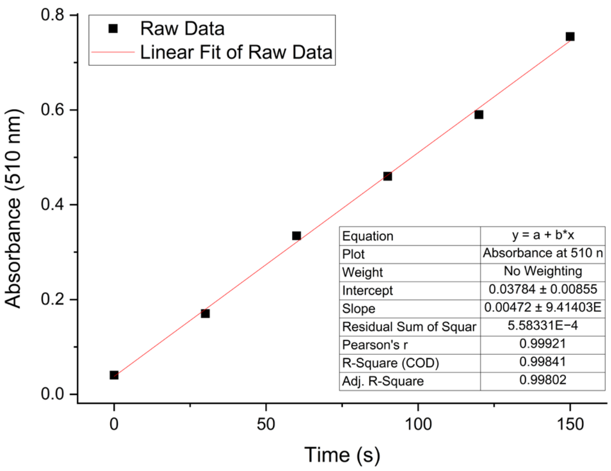

***Figure S 32**. Absorbance at 510 nm of the phenanthroline complex with iron (II) released by the photodegradation of potassium ferrioxalate after different irradiation times at 365 nm. A linear fit of the recorded data (red line and black squares, respectively) was obtained with the software OriginPro.*

Molar Extinction Coefficients

Molar extinction coefficients of the *E* isomers were determined from their UV/vis spectra recorded in the dark at various concentrations. Following the Beer-Lambert law:

$$A = \varepsilon \cdot l \cdot c$$

Where A is the absorbance at a given wavelength, l is the path length of the cuvette in cm, and c is the molar concentration of the compound (M), the molar extinction coefficients ε could be determined through a linear fitting of the absorbance against concentration plot.

Molar extinction coefficients of the *Z* isomers were estimated from their predicted pure UV/vis spectra. Pure *Z*-isomer spectra were calculated from the spectra of the pure *E* isomer and from the spectra of a mixture of known composition at the PSS. PSDs were determined according to the method reported by Fischer[4] and the isomers ratios were verified by LC-MS and/or NMR analysis (see also *E*/*Z* Compositions of PSSs).

Due to the low molar absorptivity of the compounds at 525 nm, the photoisomerization quantum yields could not be determined with sufficient accuracy at 525 nm.

Molar extinction coefficients and PSDs used to calculate the photoisomerization quantum yields at 365 nm are reported in **Table S1**.

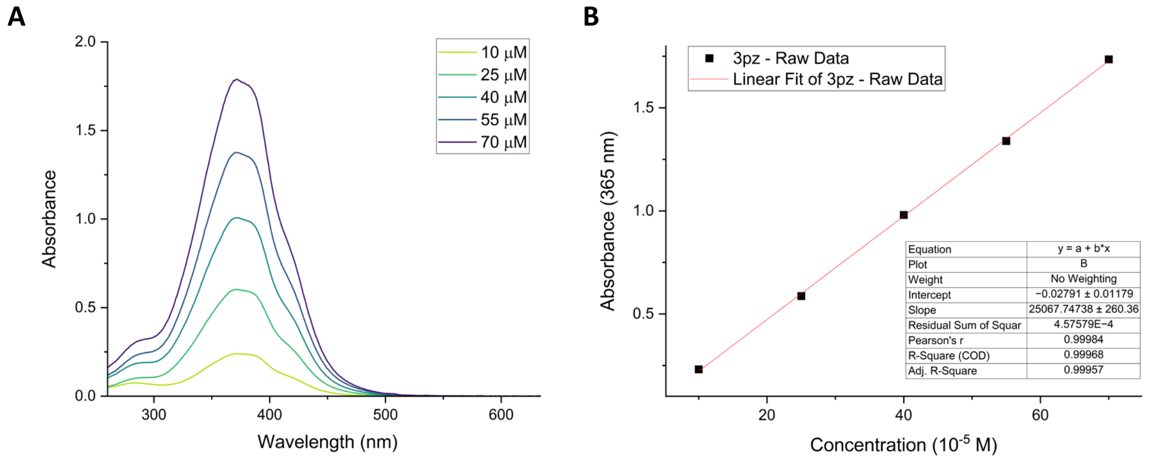

Figure S 33. *(A) UV/vis absorption spectra of **3pz** recorded at different concentrations (10-70 µM) in dry acetonitrile at 22°C. (B) Absorbance of **3pz** at 365 nm plotted against the compound concentration. A linear fitting of the data was obtained with the software OriginPro and this was used to determine the slope corresponding to the molar extinction coefficient.*

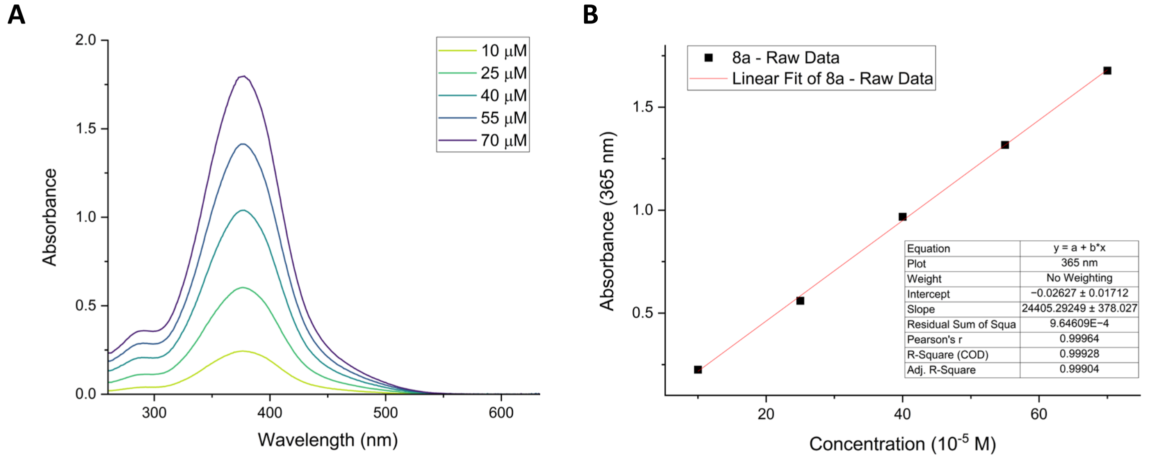

Figure S 34. *(A) UV/vis absorption spectra of **8a** recorded at different concentrations (10-70 µM) in dry acetonitrile at 22°C. (B) Absorbance of **8a** at 365 nm plotted against the compound concentration. A linear fitting of the data was obtained with the software OriginPro and this was used to determine the slope corresponding to the molar extinction coefficient.*

Photoisomerization Quantum Yields

Photoisomerization quantum yields (φ_{EZ} and φ_{ZE}) at 365 nm were determined using a previously reported literature procedure. All samples were prepared in dry acetonitrile with a final volume of 3 ml. Irradiation was performed at room temperature (T = 22°C), with the sample positioned at 3.5 cm from the light source and under continuous stirring. The quantum yields were calculated using the software provided by Stranius and Börjesson, which fits the change in absorbance over time of the *E* isomer to the equation below:[3]

$$\frac{d[E]}{dt} = -\frac{\varphi_E \cdot I \cdot \beta_E(t)}{N_a \cdot V} + \frac{\varphi_Z \cdot I \cdot \beta_Z(t)}{N_a \cdot V} + k_{t,Z\rightarrow E}[Z]$$

Where φ is the photoisomerization quantum yield of a given isomer, *I* is the photon flux, β is the fraction of photons absorbed by a given isomer, *V* is the volume of the sample in dm^3, N_a is Avogadro's numbers, k_t is the rate of the spontaneous *Z*→*E* back-isomerization.

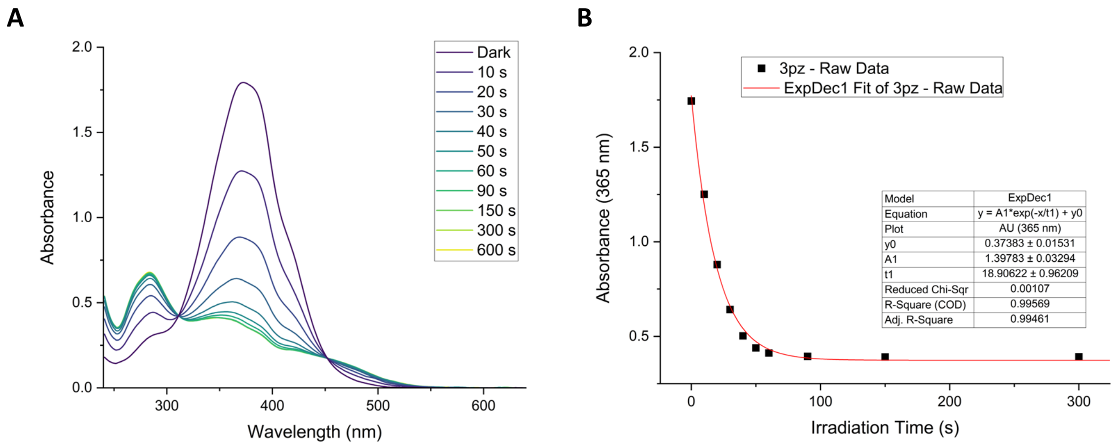

***Figure S 35**. (A) UV/vis absorption spectra of **3pz** recorded in dry acetonitrile at 22°C after increasing irradiation times at 365 nm. (B) Absorbance of **3pz** at 365 nm plotted against the irradiation time. An exponential fitting of the data was obtained with the software OriginPro.*

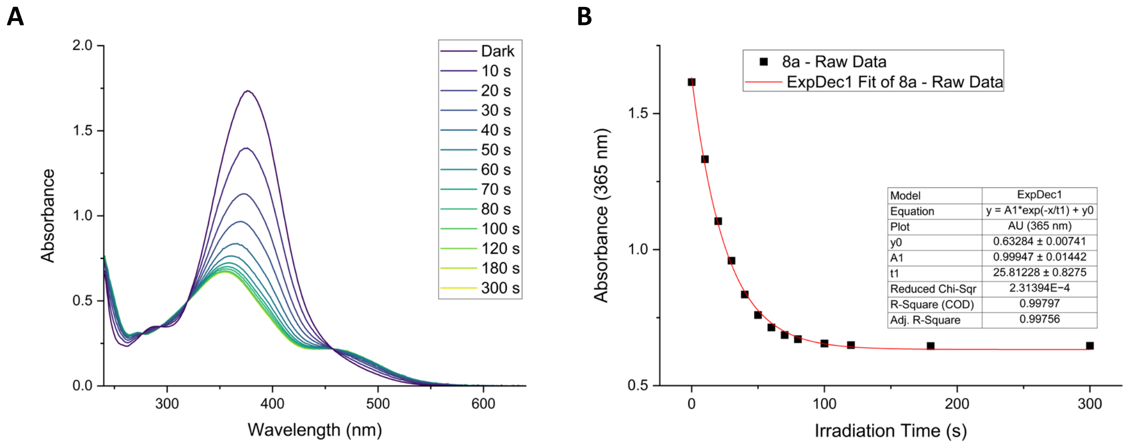

***Figure S 36**. (A) UV/vis absorption spectra of **8a** recorded in dry acetonitrile at 22°C after increasing irradiation times at 365 nm. (B) Absorbance of **8a** at 365 nm plotted against the irradiation time. An exponential fitting of the data was obtained with the software OriginPro.*

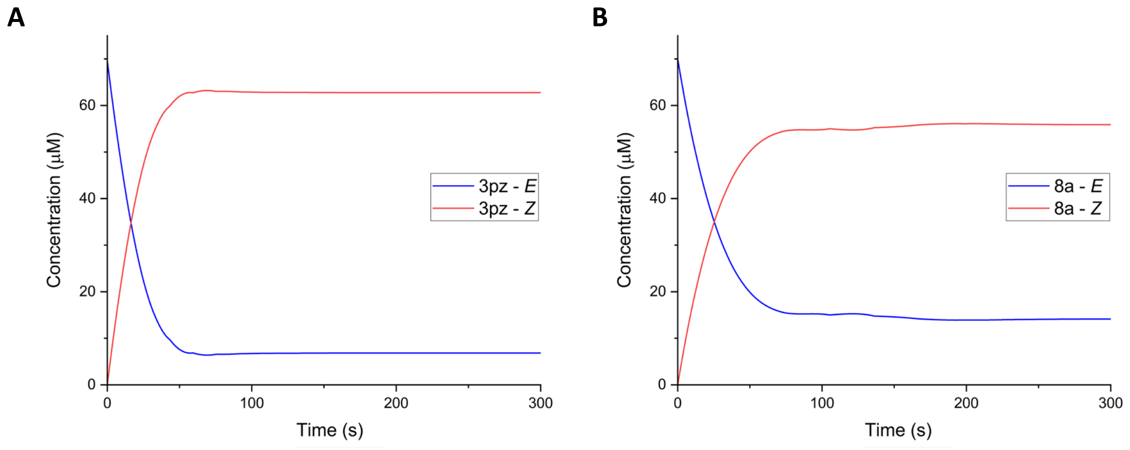

***Figure S 37**. E- and Z-isomers concentration for **3pz** (A) and **8a** (B) plotted as a function of the irradiation time at 365 nm in dry acetonitrile and at 22°C.*

Table S1. Molar extinction coefficients (ε), photostationary state distributions (PSDs) and photoisomerization quantum yields (φ) of **3pz** and **8a** at 365 nm irradiation.

	365 nm				
**Compound**	ε_E (M^{-1} cm^{-1})	ε_Z (M^{-1} cm^{-1})	PSD (% *E*)	φ_{EZ}	φ_{ZE}
**3pz**	25,068	3,630	9.7%	0.22	0.18
**8a**	24,405	5,800	19.9%	0.17	0.15

Synthesis

General Methods

Reagents and Solvents

All reagents and solvents were directly used as purchased from the following commercial suppliers: ABCR, Sigma Aldrich (Merck) and BLDPharm.

Thin-layer and Silica Column Chromatography

Reactions were monitored using analytical thin-layer chromatography (TLC) with precoated plates with silica gel 60 GF254 plates (Machery-Nagel GmbH & Co. KG). Detection was carried out by irradiation and consequent fluorescence quenching at 254 nm or excitation at 365 nm. Silica gel 60 (60 Å pore size, 40–63 µm; Macherey Nagel GmbH & Co. KG, Düren, Germany) was used as the stationary phase for compound purification by column chromatography.

Nuclear magnetic resonance (NMR) Spectroscopy

NMR ^{1}H and ^{13}C spectra were recorded with a Bruker AV-400 NMR instrument (Bruker, Karlsruhe, Germany) in deuterated solvents. Spectra were calibrated with the hydrogen signal of the respective solvent as an internal standard and chemical shifts were expressed in ppm (CD_2Cl_2: ^{1}H: 5.32 ppm, ^{13}C: 54.00 ppm; $CDCl_3$: ^{1}H: 7.26 ppm, ^{13}C: 77.16 ppm; CD_3OD: ^{1}H: 4.87 ppm, ^{13}C: 49.00 ppm, DMSO-d_6: ^{1}H: 2.50 ppm an 3.33 ppm (H_2O), ^{13}C: 39.52 ppm). *J* is the coupling constant in hertz [s^{-1}].

Liquid Chromatography Mass Spectrometry (LC-MS)

Measurements for verification and purity of the compounds were performed by LC-MS using a Shimadzu kit, equipped with a DGU-20A3R controller, a DGU-20A degasser, a LC-20AB liquid chromatograph and an SPD-20A UV/Vis detector connected to an LCMS-2020 mass spectrometer (ESI ionization). The stationary phase was a Synergi 4U fusion-RP 80A (150 × 4.6 mm) column, and a MeOH/H_2O gradient containing 0.1% formic acid was used as the mobile phase. The compounds were dissolved in MeOH and filtered through syringe filters. Data are reported as mass-to-charge ratio (m/z) of the corresponding positively charged molecular ions. All target compounds were purified to ≥ 95% evaluated by integration of peaks in the chromatogram measured at 254 nm.

Method:

Parameters: Mobile phase A: H_2O (0.1% HCOOH), mobile phase B: MeOH (0.1% HCOOH)

Flow rate: 1.0 mL/min, Detection: 254 nm; Scan range: 60-1000 *m/z*;

Gradient: 0-11 min 5% → 95% B, 11-15 min 95% B, 15-16 min 95% → 5% B, 16-18min 5% B.

Reversed-Phase Column Chromatography

Purification of target compounds was done using reversed-phase column chromatography with an Interchim PuriFlash 430 instrument (Ultra Performance Flash Purification) connected to an Interchim Flash ELSD (Detection: 200-600 nm). A Flash Pure Select C18 30 µM spherical 4g column was used with a flow rate of 10.0 mL/min.

Mobile phase A: H_2O, mobile phase B: MeOH.

Standard method:

0-7 min 60% B, 7-15 min 60% → 72% B, 15-22 min 72% B, 22-30 min 72% → 95%, 30-35 min 95% B

The gradient was manually held at the current percentage upon appearance and for the duration of a peak.

Experimental

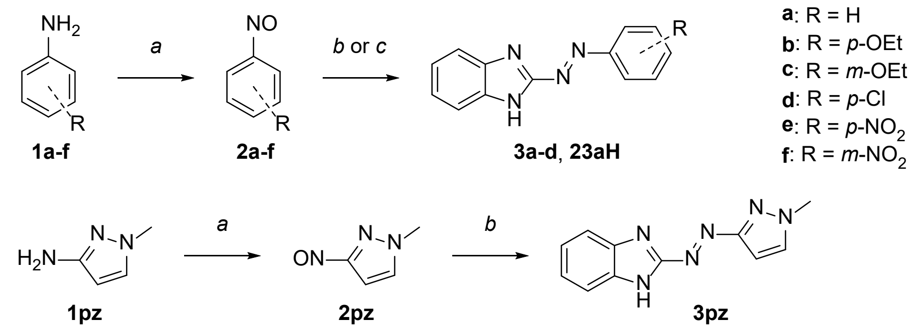

***Figure S 38.** Synthesis of arylazobenzimidazoles bearing an NH in the heterocyclic ring. Reagents and conditions: (a) oxone®, water, CH2Cl2, rt, 1-12 h; (b) 1H-benzo[d]imidazol-2-amine or 5-methoxy-1H-benzo[d]imidazol-2-amine, toluene/40% NaOH, aq. (4mL/mmol), 80-85°C, 2-6h (general procedure II); (c) 2-amino-benzimidazole, toluene/DMSO (2mL/mmol), 1 mL/mmol 40% NaOH, aq., 65°C, 30-60min (general procedure III).*

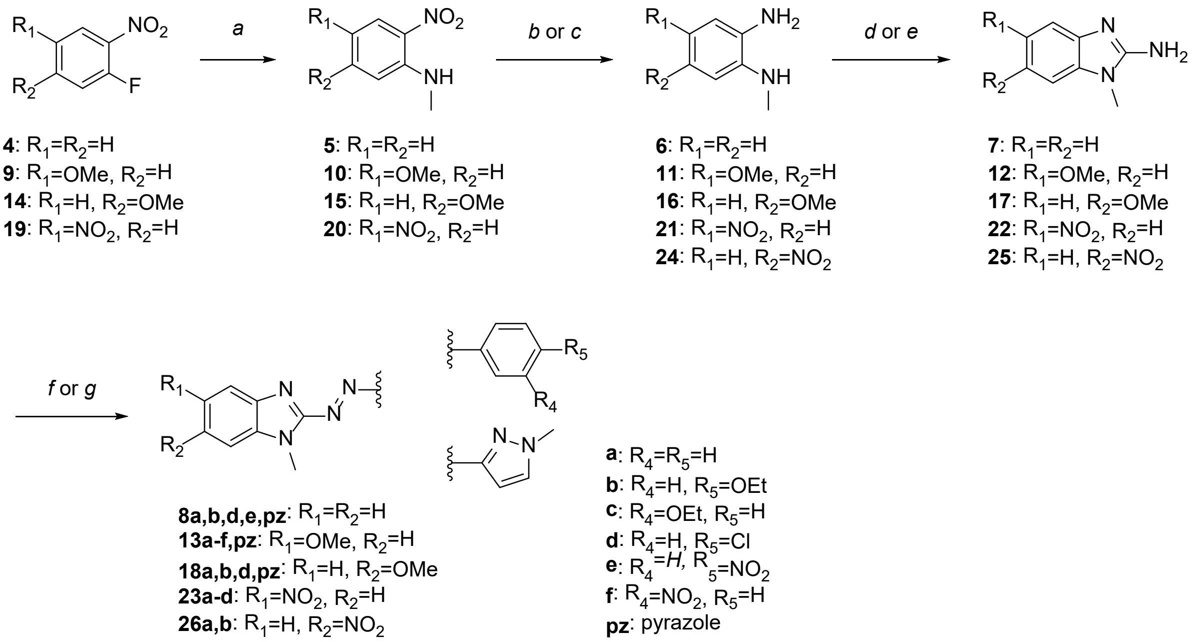

***Figure S 39.** Synthesis of methylated arylazobenzimidazole derivatives. Reagents and conditions: (a) CH_3NH_2, NEt_3, EtOH, rt or 55°C, overnight; (b) H_2, Pd/C, THF, rt, overnight; (c) Na_2SxH_2O, $NaHCO_3$, MeOH, reflux, 1h; (d) BrCN, CH_2Cl_2, RT, overnight; (e) BrCN, MeCN/H_2O, 55°C, 3h, then RT, overnight; (f) **2a-f** or **2pz**, toluene/40% NaOH, aq. (4mL/mmol), 80-85°C, 2-6h (general procedure II); (g) **2a-d** or **2pz**, toluene/DMSO (2mL/mmol), 1 mL/mmol 40% NaOH, aq., 65°C, 30-60min (general procedure III).*

General Procedure I for Oxidation to Nitroso Compounds*:* The aniline compound (1 eq.) was dissolved in dichloromethane and Oxone® (2 eq.) was dissolved in an equal amount of water. The solutions were mixed and stirred vigorously at ambient temperature for 2 – 16 h until consumption of the staring material. The reaction was quenched by addition of sat. $NaHCO_3$ solution (aq.). The layers were separated and the aqueous layer was extracted with dichloromethane (2x). The combined organic layers were dried over Na_2SO_4, filtered and evaporated under reduced pressure to yield the crude nitroso derivatives. Crude nitroso derivatives were purified by column chromatography as indicated. The respective turquoise spots were collected and used without characterization.

General Procedure II for Basic Baeyer-Mills Reaction: The respective 2-benzimidazole amine (1 eq.) and the respective nitroso compound (2 eq.) were dissolved in toluene (2 mL/mmol) and an equal amount of 40% NaOH (aq.) was added. The biphasic system was heated to 80-85°C for 2 – 6 h until consumption of the 2-benzimidazole amine derivative. The

reaction was diluted with water and the aqueous layer was extracted with EtOAc or CH_2Cl_2. The combined organic extracts were washed with brine, dried over Na_2SO_4, filtered and concentrated *in vacuo*.

General Procedure III for Basic Baeyer-Mills Reaction containing DMSO: The respective 2-benzimidazole amine (1 eq.) and the respective nitroso compound (2 eq.) were dissolved in DMSO/toluene (50:50, 2 mL/mmol) and 40% NaOH, aq. (1 mL/mmol) was added. The reaction was stirred at 65°C for 30–60 min and quenched with water. The aqueous layer was extracted with EtOAc or CH_2Cl_2 and the combined organic extracts were washed with brine (3x), dried over Na_2SO_4, filtered and concentrated *in vacuo*.

1-Ethoxy-4-nitrosobenzene (**2b**)

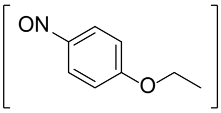

The reaction was performed according to general procedure I from commercially available 4-ethoxyaniline (280 µL, 2.19 mmol). Gravity column chromatography with 6:1 petroleum ether/EtOAc was used for purification. Compound **2b** was obtained as a turquoise solid (0.260 g, 78.7 %) and used without characterization.

1-Ethoxy-3-nitrosobenzene (**2c**)

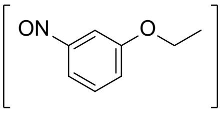

The reaction was performed according to general procedure I from commercially available 3-ethoxyaniline (467 µmL, 3.64 mmol). Gravity column chromatography with 8:1 petroleum ether/EtOAc was used for purification. Compound **2c** was obtained as a turquoise solid (229 mg, 41.6 %) and used without characterization.

1-Chloro-4-nitrosobenzene (**2d**)

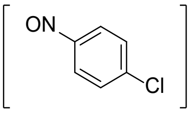

The reaction was performed according to general procedure I from commercially available 4-chloroaniline (0.401 g, 3.14 mmol). Gravity column chromatography with 1:1 petroleum ether/dichloromethane was used for purification. Compound **2d** was obtained as a turquoise solid (0.375 g, 84.5 %) and used without characterization.

1-Nitro-4-nitrosobenzene (**2e**)

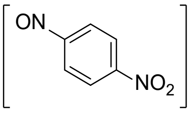

The reaction was performed according to general procedure I from commercially available 4-nitroaniline (0.400 g, 2.90 mmol). Gravity column chromatography with 2:1 petroleum ether/dichloromethane was used for purification. Compound **2e** was obtained as a turquoise solid (0.206 g, 46.8 %) and used without characterization.

1-Nitro-3-nitrosobenzene (**2f**)

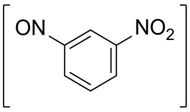

The reaction was performed according to general procedure I from commercially available 3-nitroaniline (0.400 g, 2.90 mmol). Gravity column chromatography with 3:1 petroleum ether/dichloromethane was used for purification. Compound **2f** was obtained as a turquoise solid (0.213 g, 48.4 %) and used without characterization.

1-methyl-3-nitroso-1*H*-pyrazole (**2pz**)

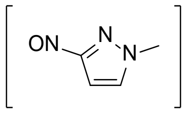

The reaction was performed according to general procedure I from commercially available 1-methyl-1H-pyrazole-3-amine (0.250 g, 2.57 mmol). Gravity column chromatography with dichloromethane was used for purification. Compound **2pz** was obtained as a turquoise solid (0.125 g, 43.7 %)) and used without characterization.

(*E*)-2-(phenyldiazenyl)-1*H*-benzo[*d*]imidazole (**3a**)

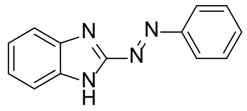

The reaction was done according to general procedure II from commercially available 1*H*-benzo[*d*]imidazol-2-amine (50.0 mg, 376 µmol) and nitrosobenzene (40.2 mg, 376 µmol). The crude product was purified by column chromatography with DCM/MeOH 50:1 + 1 % NEt_3. Compound **3a** was obtained as an orange solid (9.60 mg, 11.5 %).

^{1}H NMR (400 MHz, CD_2Cl_2) δ [ppm]: 8.07 – 7.98 (m, 2H), 7.79 – 7.69 (m, 2H), 7.64 – 7.53 (m, 3H), 7.43 – 7.35 (m, 2H). **^{13}C NMR** (101 MHz, CD_2Cl_2) δ [ppm]: 157.6, 152.7, 133.6, 130.0, 125.2, 124.2. **LC-MS analysis** (254 nm): t_r= 10.18 min (cis) and 11.65 min (trans), ESI-MS: m/z calcd for $C_{13}H_{10}N_4$, 223.09; found, 223.00.

(*E*)-2-((4-ethoxyphenyl)diazenyl)-1*H*-benzo[*d*]imidazole (**3b**)

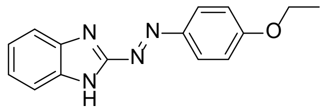

The reaction was done according to general procedure III from 1*H*-benzo[*d*]imidazol-2-amine (40.0 mg, 300 µmol) and 4-ethoxynitrosobenzene (**2b**) (90.7 mg, 600 µmol). The crude product was purified by gravity column chromatography with CH_2Cl_2 and then further purified via preparative TLC (PE/EtOAc; 7/3) to give **3b** as orange solid (7.30 mg, 9.13 %).

^{1}H NMR (400 MHz, DMSO-d_6) δ [ppm]: 12.82 (s, 1H), 8.02 – 7.95 (m, 2H), 7.80 – 7.46 (m, 2H), 7.30 (s, 2H), 7.23 – 7.15 (m, 2H), 4.19 (q, J = 6.9 Hz, 2H), 1.39 (t, J = 7.0 Hz, 3H). **^{13}C NMR** (101 MHz, DMSO-d_6) δ [ppm]: 163.1, 157.9, 146.6, 125.8, 115.9, 64.4, 15.0. **LC-MS analysis** (254 nm): t_r= 12.14 min (trans), ESI-MS: m/z calcd for $C_{15}H_{14}N_4O$, 267.12; found, 267.10.

(*E*)-2-((3-ethoxyphenyl)diazenyl)-1*H*-benzo[*d*]imidazole (**3c**)

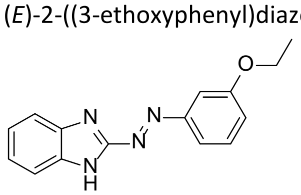

The reaction was done according to general procedure III from compound 1*H*-benzo[*d*]imidazol-2-amine (50.0 mg, 376 µmol) and compound **2c** (114 mg, 751 µmol). Purification was done using column chromatography with CH_2Cl_2/MeOH 99:1 + 0.5 % NEt_3 to give compound **3c** as an orange solid (18.6 mg, 18.6%).

^{1}H NMR (400 MHz, $CDCl_3$) δ 7.83 – 7.65 (m, 2H), 7.62 (d, *J* = 1.0 Hz, 1H), 7.52 (t, *J* = 2.2 Hz, 1H), 7.43 (t, *J* = 8.0 Hz, 1H), 7.40 – 7.33 (m, 2H), 7.14 – 7.08 (m, 1H), 4.06 (q, *J* = 7.0 Hz, 2H), 1.43 (t, *J* = 7.0 Hz, 3H). **^{13}C NMR** (101 MHz, $CDCl_3$) δ 159.8, 156.9, 153.3, 130.0, 121.1, 119.7, 104.9, 63.8, 14.7. **LC-MS analysis** (254 nm): t_r= 10.54 min (cis) and 12.09 min (trans), ESI-MS: m/z calcd for $C_{15}H_{14}N_4O$, 267.12; found, 267.05.

(*E*)-2-((4-chlorophenyl)diazenyl)-1*H*-benzo[*d*]imidazole (**3d**)

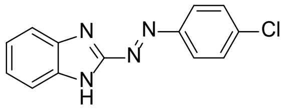

The reaction was done according to general procedure III from 1*H*-benzo[*d*]imidazol-2-amine (40.0 mg, 300 µmol) compound **2d** (90.7 mg, 600 µmol). The crude product was purified by gravity column chromatography with CH_2Cl_2 and then further purified via prep-TLC (PE/EtOAc; 7/3) to give compound **3d** as orange solid (7.30 mg, 9.13 %).

^{1}H NMR (400 MHz, DMSO-d$_6$) δ [ppm]: 13.06 (s, 1H), 8.00 (d, *J* = 8.5 Hz, 2H), 7.78 – 7.60 (m, 4H), 7.38 – 7.29 (m, 2H).**^{13}C NMR** (101 MHz, DMSO-d$_6$) δ [ppm]: 157.5, 151.0, 137.9, 130.4, 125.1. **LC-MS analysis** (254 nm): t_r= 11.36 min (cis) and 12.56 min (trans), ESI-MS: m/z calcd for $C_{13}H_9ClN_4$, 257.05; found, 257.00.

(*E*)-5-methoxy-2-(phenyldiazenyl)-1*H*-benzo[*d*]imidazole (**13aH**)

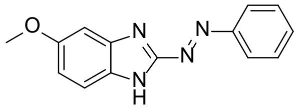

The reaction was done according to general procedure II from 5-methoxy-1*H*-benzo[*d*]imidazol-2-amine (50.0 mg, 306 µmol) and nitrosobenzene (65.6 mg, 613 µmol). Pre-purification was performed using column chromatography with petroleum ether/EtOAc 4:1. Pure **13aH** was obtained after preparative TLC with CH_2Cl_2 + 0.1 % MeOH as an orange solid (24.1 mg, 31.5%).

^{1}H NMR (400 MHz, CDCl3) δ 10.58 (s, 1H), 8.00 – 7.88 (m, 2H), 7.78 (s, 1H), 7.48 (qd, *J* = 4.4, 1.7 Hz, 3H), 6.98 (d, *J* = 8.9 Hz, 2H), 3.82 (s, 3H). **^{13}C NMR** (101 MHz, $CDCl_3$) δ 152.6, 132.9, 129.7, 124.0, 56.2. **LC-MS analysis** (254 nm): t_r= 11.93 min (*trans*), ESI-MS: m/z calcd for $C_{14}H_{12}N_4O$, 253.10; found, 253.00.

(*E*)-2-((1-methyl-1*H*-pyrazol-3-yl)diazenyl)-1*H*-benzo[*d*]imidazole (**3pz**)

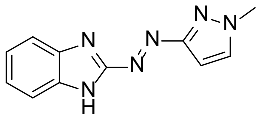

The reaction was done according to general procedure II from 1*H*-benzo[*d*]imidazol-2-amine (50 mg, 0.376 mmol) and compound **2pz** (45.9 mg, 0.413 mmol). Purification was done via column chromatography using CH_2Cl_2 + 1.5 % MeOH. Compound **3pz** was obtained as an orange solid (63.0 mg, 74.2 %).

^{1}H NMR (400 MHz, DMSO-*d$_6$*) δ [ppm]: 13.02 (s, 1H), 7.93 – 7.88 (m, 1H), 7.77 (d, J = 8.1 Hz, 1H), 7.50 (d, J = 8.0 Hz, 1H), 7.38 – 7.20 (m, 2H), 6.70 – 6.66 (m, 1H), 4.01 (d, J = 1.6 Hz, 3H). **^{13}C NMR** (101 MHz, DMSO-*d$_6$*) δ [ppm]: 163.1, 157.6, 143.6, 133.8, 133.5, 125.4, 122.5, 120.5, 112.5, 95.4. **LC-MS analysis** (254 nm): t_r= 7.57 min (cis) and 9.60 min (trans), ESI-MS: m/z calcd for $C_{11}H_{10}N_6$, 227.10; found, 227.20.

N-methyl-2-nitroaniline **(5)**

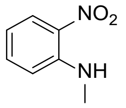

1-Fluoro-4-nitrobenzene (2.00 g, 14.2 mmol) was dissolved in EtOH and methylamine (33% in EtOH; 6.62 mL, 53.2 mmol) and triethylamine (2.40 mL, 17.1 mmol) were added. The reaction was stirred at rt for 18h. The solvent was evaporated *in vacuo* and compound **5** was directly used for the next reaction (2.16 g, quant yield).

*N*1-methylbenzene-1,2-diamine **(6)**

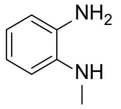

Compound **5** (2.16 g, 14.2 mmol) was dissolved in THF and Pd/C (10 %wt) was added under argon atmosphere. Then, the flask was equipped with hydrogen and the solution was stirred under hydrogen-atmosphere for 24 h.

The mixture was filtered through Celite® and the solvent was removed *in vacuo*. Compound **6** was obtained as a dark brown oil (1.73 g, quant. yield) and directly used for the next step.

1-methyl-1*H*-benzo[*d*]imidazol-2-amine (**7**)

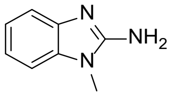

Cyanogen bromide (1.35 g, 12.8 mmol) was dissolved in CH_2Cl_2 and compound **6** (1.20 g, 9.82 mmol) was added, then the reaction was stirred at RT overnight. The solution was cooled in an ice bath for 10 mins and quenched by addition of 1N NaOH (aq.). The aqueous layer was diluted with brine and extracted with methylene chloride. The combined organic layers were washed with brine, dried over Na_2SO_4, filtered and concentrated in vacuo. The crude compound was purified by column chromatography using CH_2Cl_2 + 2% MeOH + 0.1 % NEt_3 to obtain compound **7** as a dark brown solid (0.556 g, 38.5 %).

^{1}H NMR (400 MHz, $CDCl_3$) δ [ppm]: 7.41 – 7.38 (m, 1H), 7.15 – 7.03 (m, 3H), 4.70 (brs, 2H), 3.53 (s, 3H). **^{13}C NMR** (101 MHz, $CDCl_3$) δ [ppm]: 153.8, 141.1, 134.6, 121.8, 120.1, 116.2, 107.7, 28.8.

(*E*)-1-methyl-2-(phenyldiazenyl)-1*H*-benzo[*d*]imidazole (**8a**)

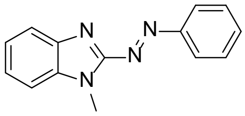

The reaction was done according to general procedure III using compound **7** (40.2 mg, 0.273 mmol) and commercially available nitrosobenzene (58.5 mg, 0.546 mmol). Purification was done via column chromatography using CH_2Cl_2. Compound **8a** was obtained as an orange solid (58.3 mg, 90.3 %).

^{1}H NMR (400 MHz, $CDCl_3$) δ 8.14 – 8.07 (m, 2H), 7.94 – 7.89 (m, 1H), 7.57 – 7.52 (m, 3H), 7.49 – 7.44 (m, 1H), 7.41 – 7.32 (m, 2H), 4.17 (s, 3H). **^{13}C NMR** (101 MHz, $CDCl_3$) δ 155.1, 153.3, 142.2, 136.2, 132.8, 129.3, 124.6, 124.1, 123.9, 122.2, 110.1, 29.9. **LC-MS analysis** (254 nm): t_r= 10.89 min (cis) and 11.91 min (trans), ESI-MS: m/z calcd for $C_{14}H_{12}N_4$, 237.11; found, 237.10.

(*E*)-2-((4-ethoxyphenyl)diazenyl)-1-methyl-1*H*-benzo[*d*]imidazole (**8b**)

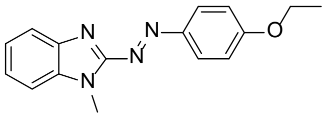

The reaction was done according to general procedure III from compound **7** (42.4 mg, 0.288 mmol) and compound **2b** (87.1 mg, 0.576 mmol). Purification was done via column chromatography using CH_2Cl_2 + 0.5% MeOH. Compound **8b** was obtained as an orange solid (23.6 mg, 29.2 %).

^{1}H NMR (400 MHz, $CDCl_3$) δ [ppm]: 8.11 (d, 2H), 7.94 – 7.85 (m, 1H), 7.51 – 7.42 (m, 1H), 7.40 – 7.30 (m, 2H), 7.01 (d, 2H), 4.19 – 4.09 (m, 5H), 1.47 (t, *J* = 1.8 Hz, 3H). **^{13}C NMR** (101 MHz, $CDCl_3$) δ [ppm]: 163.3, 155.6, 147.9, 142.3, 136.2, 126.3, 124.1, 123.9, 121.9, 115.0, 109.9, 64.2, 29.9, 14.8. **LC-MS analysis** (254 nm): t_r= 11.30 min (*trans*), ESI-MS: m/z calcd for $C_{16}H_{16}N_4O$, 281.13; found, 281.10.

(*E)*-2-((4-chlorophenyl)diazenyl)-1-methyl-1*H*-benzo[*d*]imidazole (**8d**)

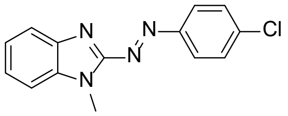

The reaction was done according to general procedure III from compound **7** (42.4 mg, 0.288 mmol) and compound **2d** (87.1 mg, 0.576 mmol). Purification was done via column chromatography using CH_2Cl_2 + 0.5% MeOH. Compound **8d** was obtained as an orange solid (62.1 mg, 89.3 %).

^{1}H NMR (400 MHz, $CDCl_3$) δ [ppm]: 8.10 – 8.04 (m, 2H), 7.95 – 7.89 (m, 1H), 7.57 – 7.47 (m, 3H), 7.45 – 7.33 (m, 2H), 4.20 (s, 3H). ^{13}C NMR (101 MHz, $CDCl_3$) δ [ppm]: 155.0, 151.7, 142.3, 139.0, 136.3, 129.7, 125.0, 124.9, 124.3,

122.2, 110.1, 30.0. **LC-MS analysis** (254 nm): t_r= 11.05 min (*cis*) and 12.26 min (*trans*), ESI-MS: m/z calcd for $C_{14}H_{11}ClN_4$, 271.07; found, 271.00.

(*E*)-1-methyl-2-((4-nitrophenyl)diazenyl)-1*H*-benzo[*d*]imidazole (**8e**)

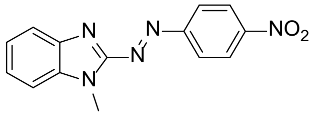

The reaction was done according to general procedure II from compound **7** (35.0 mg, 0.238 mmol) and 2e (43.4 mg, 0.285 mmol). Purification was done via preparative TLC using CH_2Cl_2 + 0.2% MeOH. Compound **8e** was obtained as an orange solid (9.42 mg, 14.1 %).

^{1}H NMR (400 MHz, $CDCl_3$) δ [ppm]: 8.47 – 8.39 (m, 2H), 8.27 – 8.18 (m, 2H), 7.94 (dt, *J* = 7.8, 1.0 Hz, 1H), 7.54 (dt, *J* = 8.4, 1.0 Hz, 1H), 7.50 – 7.36 (m, 2H), 4.26 (s, 3H). ^{13}C NMR (101 MHz, $CDCl_3$) δ [ppm]: 156.2, 154.9, 149.7, 142.5, 136.6, 125.8, 125.0, 124.4, 122.8, 110.5, 30.2. **LC-MS analysis** (254 nm): t_r= 11.34 min (*cis*) and 12.15 min (*trans*), ESI-MS: m/z calcd for $C_{14}H_{11}N_5O_2$, 282.09; found, 282.15.

(*E*)-1-methyl-2-((1-methyl-1*H*-pyrazol-3-yl)diazenyl)-1*H*-benzo[*d*]imidazole (**8pz**)

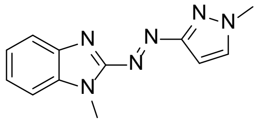

The reaction was done according to general procedure II from compound **7** (54.0 mg, 0.330 mmol) and **2pz** (40.4 mg, 0.363 mmol). Purification was done via column chromatography using CH_2Cl_2 + 2 % MeOH. Compound **8pz** was obtained as an orange solid (60.2 mg, 75.9 %).

^{1}H NMR (400 MHz, MeOD) δ [ppm]: 7.81 – 7.75 (m, 1H), 7.74 – 7.69 (m, 1H), 7.67 – 7.62 (m, 1H), 7.47 – 7.36 (m, 2H), 6.78 (dd, J = 2.5, 0.9 Hz, 1H), 4.17 (s, 3H), 4.07 (s, 3H). **^{13}C NMR** (101 MHz, MeOD) δ [ppm]: 165.6, 156.4, 142.5, 137.4, 134.4, 125.9, 125.3, 121.5, 111.8, 97.5, 40.0, 30.5. **LC-MS analysis** (254 nm): t_r= 8.19 min (*cis*) and 9.07 min (*trans*), ESI-MS: m/z calcd for $C_{12}H_{12}N_6$, 241.11; found, 241.00.

4-methoxy-*N*-methyl-2-nitroaniline (**10**)

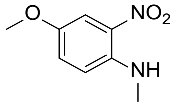

1-Fluoro-4-methoxy-2-nitrobenzene (2.00 g, 11.7 mmol) was dissolved in EtOH and methylamine (33% in EtOH; 5.46 mL, 43.8 mmol) and triethylamine (1.95 mL, 14.0 mmol) were added. The reaction was stirred at rt for 18h. The solvent was evaporated *in vacuo*. The residue was dissolved in EtOAc and washed with water, dried over Na_2SO_4, and concentrated in vacuo. Compound **10** was used for the next reaction without further purification (1.45 g, 68.1 %).

^{1}H NMR (400 MHz, $CDCl_3$) δ [ppm]: 7.97 (s, 1H), 7.63 (d, *J* = 3.0 Hz, 1H), 7.20 – 7.15 (m, 1H), 6.83 (d, *J* = 9.3 Hz, 1H), 3.80 (s, 3H), 3.02 (d, *J* = 5.2 Hz, 3H). **^{13}C NMR** (101 MHz, $CDCl_3$) δ [ppm]: 149.7, 142.5, 131.0, 127.6, 114.9, 107.2, 56.0, 30.1.

4-methoxy-*N*1-methylbenzene-1,2-diamine **(11)**

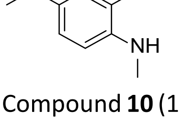

Compound **10** (1.35 g, 7.41 mmol) was dissolved in EtOAc and Pd/C (10 %wt) was added under argon atmosphere. Then, the flask was equipped with hydrogen and the solution was stirred at 40°C under hydrogen-atmosphere for 20 h. The mixture was filtered through Celite® and the solvent was removed in vacuo. Compound **11** was used for the next reaction without purification (1.12 g, quant. yield).

^{1}H NMR (400 MHz, $CDCl_3$) δ [ppm]: 7.51 (d, *J* = 8.3 Hz, 1H), 7.30 – 7.24 (m, 2H), 4.64 (s, 3H), 4.19 (s, 3H), 3.72 (s, 3H). **^{13}C NMR** (101 MHz, $CDCl_3$) δ [ppm]: 153.7, 136.8, 132.3, 113.0, 103.9, 103.3, 55.7, 31.9.

5-methoxy-1-methyl-1*H*-benzo[*d*]imidazol-2-amine (**12**)

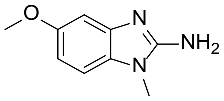

Cyanogen bromide (0.627 g, 5.92 mmol) and compound **11** (0.300 g, 1.97 mmol) were mixed and then dissolved in CAN/H_2O (1:1) and heated to 50 C for 1 h. The solution was cooled in an ice bath for 10 mins and quenched by addition of 1N NaOH (aq.). The aqueous layer was diluted with brine and extracted with EtOAc. The combined organic layers were washed with brine, dried over Na_2SO_4, filtered and concentrated *in vacuo*. Compound **12** was purified via column chromatography with a gradient of 98:2 CH_2Cl_2/MeOH + 0.5 % NEt_3 to 9:1CH_2Cl_2/MeOH + 0.5 % NEt_3 and obtained as a red solid (0.180 g, 52.0 %).

^{1}H NMR (400 MHz, MeOD) δ [ppm]: 7.29 (d, *J* = 8.8 Hz, 1H), 6.92 (d, *J* = 2.3 Hz, 1H), 6.87 (dd, *J* = 8.8, 2.3 Hz, 1H), 3.80 (s, 3H), 3.61 (s, 3H). **^{13}C NMR** (101 MHz, MeOD) δ [ppm]: 158.9, 151.8, 131.0, 126.4, 97.9, 56.5, 29.5.

(*E*)-5-methoxy-1-methyl-2-(phenyldiazenyl)-1*H*-benzo[*d*]imidazole (**13a**)

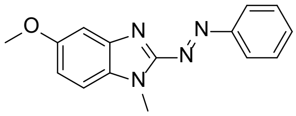

The reaction was done according to general procedure II from compound **12** (50 mg, 282 µmol) and commercially available nitrosobenzene (66.4 mg, 564 µmol). Pre-purification was performed using column chromatography with CH_2Cl_2/MeOH 95:5. Preparative TLC with CH_2Cl_2 + 0.1 % MeOH gave the pure compound **13a** (29.8 mg, 39.7%).

^{1}H NMR (400 MHz, $CDCl_3$) δ 8.14 – 8.04 (m, 2H), 7.58 – 7.49 (m, 3H), 7.35 (d, J = 8.9 Hz, 1H), 7.31 (d, J = 2.3 Hz, 1H), 7.04 (dd, J = 8.9, 2.4 Hz, 1H), 4.15 (s, 3H), 3.87 (s, 3H). **^{13}C NMR** (101 MHz, $CDCl_3$) δ 157.6, 155.3, 153.4, 143.3, 132.6, 131.2, 129.3, 123.8, 116.1, 110.7, 102.6, 55.8, 30.0. **LC-MS analysis** (254 nm): t_r= 9.71 min (*cis*) and 10.25 min (*trans*), ESI-MS: m/z calcd for $C_{15}H_{14}N_4O$, 267.12; found, 267.20.

(*E*)-2-((4-ethoxyphenyl)diazenyl)-5-methoxy-1-methyl-1*H*-benzo[*d*]imidazole (**13b**)

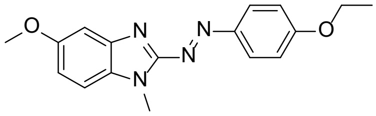

The reaction was done according to general procedure II from compound **12** (40.0 mg, 226 µmol) and **2b** (68.2 mg, 451 µmol). Pre-purification was done with column chromatography (CH_2Cl_2: MeOH 95:5). Compound **13b** (10.5 mg, 15.0%) was obtained after purification via preparative TLC (CH_2Cl_2 + 0.15% MeOH).

^{1}H NMR (400 MHz, $CDCl_3$) δ 8.13 – 8.06 (m, 2H), 7.34 (d, J = 8.9 Hz, 1H), 7.30 (d, J = 2.3 Hz, 1H), 7.04 – 6.98 (m, 3H), 4.18 – 4.09 (m, 5H), 3.87 (s, 3H), 1.47 (t, J = 7.0 Hz, 3H). **^{13}C NMR** (101 MHz, $CDCl_3$) δ 163.0, 157.4, 155.7, 147.9, 143.3, 131.1, 126.1, 115.3, 115.0, 110.5, 102.7, 64.1, 55.9, 29.9, 14.9. **LC-MS analysis** (254 nm): t_r= 10.19 min (*trans*), ESI-MS: m/z calcd for $C_{17}H_{18}N_4O_2$, 311.14; found, 311.10.

(*E*)-2-((3-ethoxyphenyl)diazenyl)-5-methoxy-1-methyl-1*H*-benzo[*d*]imidazole (**13c**)

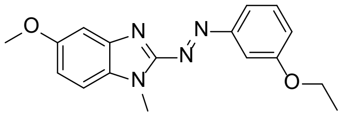

The reaction was done according to general procedure II using compound **12** (40.0 mg, 226 µmol) and **2c** (68.2 mg, 451 µmol). Pre-purification was performed using column chromatography with CH_2Cl_2/ MeOH 95:5. Purification via preparative TLC (CH_2Cl_2: MeOH 99.85:0.15) yielded compound **13c** as an orange solid (8.22 mg, 11.7%).

^{1}H NMR (400 MHz, $CDCl_3$) δ 7.76 – 7.70 (m, 1H), 7.64 – 7.60 (m, 1H), 7.44 (t, J = 8.0 Hz, 1H), 7.37 (d, J = 8.9 Hz, 1H), 7.31 (d, J = 2.3 Hz, 1H), 7.12 – 7.07 (m, 1H), 7.05 (dd, J = 8.9, 2.4 Hz, 1H), 4.20 – 4.10 (m, 5H), 3.88 (s, 3H), 1.47 (t, J = 7.0 Hz, 3H). **^{13}C NMR** (101 MHz, $CDCl_3$) δ 159.8, 157.7, 154.6, 143.3, 131.3, 130.0, 119.8, 117.2, 116.2,

110.7, 108.1, 102.7, 63.9, 55.9, 30.1, 14.9. **LC-MS analysis** (254 nm): t_r= 10.43 min (*cis*) and 11.22 min (*trans*), ESI-MS: m/z calcd for $C_{17}H_{18}N_4O_2$, 311.14; found, 311.10.

(*E*)-2-((4-chlorophenyl)diazenyl)-5-methoxy-1-methyl-1*H*-benzo[*d*]imidazole **(13d)**

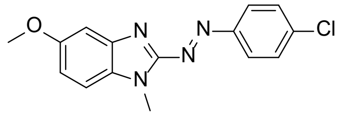

The reaction was done according to general procedure II from compound **12** (50.0 mg, 282 µmol) and **2d** (79.9 mg, 564 µmol). Pre-purification was done via column chromatography (CH_2Cl_2: MeOH 98:2). Reversed-phase flash column chromatography yielded compound **13d** as an orange solid (38.9 mg, 45.8 %).
^{1}H NMR (400 MHz, $CDCl_3$) δ [ppm]: 8.06 – 7.95 (m, 2H), 7.51 – 7.44 (m, 2H), 7.32 (d, *J* = 8.9 Hz, 1H), 7.27 (d, *J* = 2.3 Hz, 1H), 7.02 (dd, *J* = 8.9, 2.4 Hz, 1H), 4.11 (s, 3H), 3.86 (s, 3H). **^{13}C NMR** (101 MHz, $CDCl_3$) δ [ppm]: 157.74, 155.09, 151.70, 143.31, 138.59, 131.23, 129.64, 124.90, 116.39, 110.76, 102.52, 55.80, 29.98. **LC-MS analysis** (254 nm): t_r= 11.80 min (*cis*) and 12.73 min (*trans*), ESI-MS: m/z calcd for $C_{15}H_{13}ClN_4O$, 301.08; found, 301.05.

(*E*)-5-methoxy-1-methyl-2-((4-nitrophenyl)diazenyl)-1*H*-benzo[*d*]imidazole (**13e**)

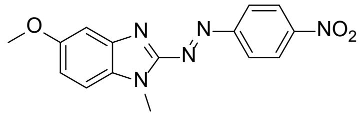

The reaction was done according to general procedure II from compound **12** (50 mg, 282 µmol) and **2e** (85.8 mg, 564 µmol). Pre-purification was performed with flash column chromatography (CH_2Cl_2: MeOH 99:1). Preparative TLC with CH_2Cl_2 + 0.5% MeOH yielded compound **13e** (7.89 mg, 8.98 %) as an orange solid.
^{1}H NMR (400 MHz, CD_2Cl_2) δ 8.5 – 8.4 (m, 2H), 8.2 – 8.1 (m, 2H), 7.5 (d, *J* = 9.0 Hz, 1H), 7.3 (d, *J* = 2.4 Hz, 1H), 7.1 (dd, *J* = 9.0, 2.4 Hz, 1H), 4.2 (s, 3H), 3.9 (s, 3H). **^{13}C NMR** (101 MHz, CD_2Cl_2) δ [ppm]:158.6, 156.9, 149.9, 144.0, 125.4, 124.6, 118.0, 111.8, 102.6, 56.3, 30.8. **LC-MS analysis** (254 nm): t_r= 12.62 min (*trans*), ESI-MS: m/z calcd for $C_{15}H_{13}N_5O_3$, 312.10; found, 312.10.

(*E*)-5-methoxy-1-methyl-2-((3-nitrophenyl)diazenyl)-1*H*-benzo[*d*]imidazole (**13f**)

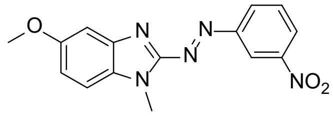

The reaction was done according to general procedure II from compound **12** (50.0 mg, 282 µmol) and **2f** (64.4 mg, 423 µmol). Pre-purification was performed with column chromatography (CH_2Cl_2: MeOH 99:1). Reversed-phase column chromatography yielded compound **13f** as an orange solid (2.81 mg, 3.20 %).
^{1}H NMR (400 MHz, CD_2Cl_2) δ [ppm]: 8.73 (t, *J* = 2.1 Hz, 1H), 8.37 – 8.33 (m, 1H), 8.33 – 8.28 (m, 1H), 7.71 (t, *J* = 8.0 Hz, 1H), 7.37 (d, *J* = 8.9 Hz, 1H), 7.18 (d, *J* = 2.4 Hz, 1H), 7.02 (dd, *J* = 8.9, 2.4 Hz, 1H), 4.12 (s, 3H), 3.81 (s, 3H). **^{13}C NMR** (101 MHz, $CDCl_3$) δ 158.2, 153.7, 149.3, 143.6, 131.7, 131.6, 130.5, 126.1, 117.6, 115.8, 111.1, 102.5, 55.9, 30.3. **LC-MS analysis** (254 nm): tr= 10.33 min (*cis*) and 11.20 min (*trans*), ESI-MS: m/z calcd for $C_{15}H_{13}N_5O_3$, 312.10; found, 312.05.

(*E*)-5-methoxy-1-methyl-2-((1-methyl-1*H*-pyrazol-3-yl)diazenyl)-1*H*-benzo[*d*]imidazole (**13pz**)

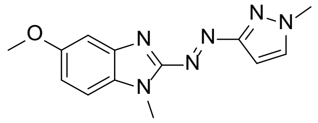

The reaction was done according to general procedure II using compound **12** (25.0 mg, 141 µmol) and **2pz** (31.4 mg, 282 µmol). Pre-purification was done with column chromatography (CH_2Cl_2: MeOH 98:2). Compound **13pz** was obtained as an orange solid (15.7 mg, 41.2 %) after purification via reversed-phase column chromatography.
^{1}H NMR (400 MHz, $CDCl_3$) δ [ppm]: 7.42 (d, *J* = 2.4 Hz, 1H), 7.33 (d, *J* = 8.9 Hz, 1H), 7.29 (d, *J* = 2.3 Hz, 1H), 7.03 (dd, *J* = 8.9, 2.4 Hz, 1H), 6.82 (d, *J* = 2.4 Hz, 1H), 4.12 (s, 3H), 4.04 (s, 3H), 3.87 (s, 3H). **^{13}C NMR** (101 MHz, $CDCl_3$)

δ [ppm]: 164.1, 157.4, 155.7, 143.4, 132.2, 131.3, 116.1, 110.7, 102.6, 99.8, 55.9, 40.1, 30.7. **LC-MS analysis** (254 nm): t_r= 8.50 min (*cis*) and 9.17 min (*trans*), ESI-MS: m/z calcd for $C_{13}H_{14}N_6O$, 271.12; found, 271.10.

5-methoxy-*N*-methyl-2-nitroaniline **(15)**

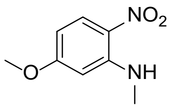

3-Fluoro-4-nitroanisole (2.00 g, 11.7 mmol) was dissolved in EtOH and methylamine (33% in EtOH; 3.49 mL, 28.1 mmol) and triethylamine (1.95 mL, 28.1 mmol) were added. The solution was stirred at rt for 18h. The solution was cooled in an ice bath for 30 min and the red crystals were collected via vacuum filtration, washed with cold EtOH and dried under vacuum (1.96 g, 92.0 %).

^{1}H NMR (400 MHz, $CDCl_3$) δ [ppm]: 8.28 (s, 1H), 8.14 (d, J = 9.5 Hz, 1H), 6.24 (dd, J = 9.5, 2.6 Hz, 1H), 6.13 (d, J = 2.6 Hz, 1H), 3.88 (s, 3H), 3.01 (d, J = 5.1 Hz, 3H). **^{13}C NMR** (101 MHz, $CDCl_3$) δ [ppm]: 166.2, 148.8, 129.4, 104.6, 95.0, 55.8, 29.8.

5-methoxy-*N*1-methylbenzene-1,2-diamine (**16**)

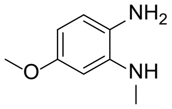

Compound **15** (1.95 g, 19.7 mmol) was dissolved in THF and Pd/C (10 %wt) was added under argon atmosphere. Then, the flask was equipped with hydrogen and the solution was stirred under hydrogen-atmosphere for 24 h. The mixture was filtered through Celite® and the solvent was removed in vacuo. Compound **16** was obtained as a dark oil (1.61 g, quant. yield) was used for the next reaction without further purification.

^{1}H NMR (400 MHz, $CDCl_3$) δ [ppm]: 6.65 (d, *J* = 8.2 Hz, 1H), 6.26 (d, *J* = 2.7 Hz, 1H), 6.19 (dd, *J* = 8.2, 2.7 Hz, 1H), 3.77 (s, 3H), 2.99 (s, 2H), 2.85 (s, 3H). **^{13}C NMR** (101 MHz, $CDCl_3$) δ [ppm]: 155.6, 141.6, 126.8, 117.8, 100.9, 98.5, 55.7, 30.9.

6-methoxy-1-methyl-1*H*-benzo[*d*]imidazol-2-amine **(17)**

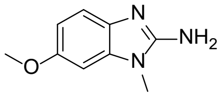

Compound **16** (0.510 g, 3.35 mmol) and cyanogen bromide (0.710 g, 6.70 mmol) were dissolved in acetonitrile/water (1:1) and heated to 50°C for 5 h. The reaction was stirred in an ice bath and quenched by addition of 1N NaOH (aq.). The aqueous layer was diluted with brine and extracted with methylene chloride. The combined organic layers were washed with brine, dried over Na_2SO_4, filtered and concentrated in vacuo. The crude compound was purified by column chromatography using CH_2Cl_2 + 2% MeOH to obtain compound **17** as a dark purple solid (0.484 g, 81.5 %).

^{1}H NMR (400 MHz, DMSO-d_6) δ [ppm]: 7.00 (d, *J* = 8.4 Hz, 1H), 6.77 (d, *J* = 2.5 Hz, 1H), 6.55 (dd, *J* = 8.4, 2.5 Hz, 1H), 6.23 (s, 2H), 3.74 (s, 3H), 3.46 (s, 3H). **^{13}C NMR** (101 MHz, DMSO-d_6) δ 154.7, 153.3, 136.3, 135.3, 114.6, 107.3, 93.7, 55.6, 28.4.

(*E*)-6-methoxy-1-methyl-2-(phenyldiazenyl)-1*H*-benzo[*d*]imidazole (**18a**)

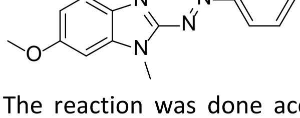

The reaction was done according to general procedure III using compound **17** (40.0 mg, 0.226 mmol) and nitrosobenzene (48.4 mg, 0.451 mmol). Purification was done using column chromatography with CH_2Cl_2 + 0.2% MeOH to give compound **18a** as an orange solid (51.1 mg, 85.0 %).

^{1}H NMR (400 MHz, CD_2Cl_2) δ [ppm]: 8.08 – 8.01 (m, 2H), 7.72 (d, *J* = 8.9 Hz, 1H), 7.60 – 7.53 (m, 3H), 6.99 (dd, *J* = 8.9, 2.5 Hz, 1H), 6.90 (d, *J* = 2.4 Hz, 1H), 4.12 (s, 3H), 3.92 (s, 3H). **^{13}C NMR** (101 MHz, CD_2Cl_2) δ [ppm]: 158.7,

155.5, 153.9, 137.7, 137.6, 132.8, 129.8, 123.9, 123.1, 115.2, 92.8, 56.4, 30.6. **LC-MS analysis** (254 nm): T_r= 11.20 min (*cis*) and 12.33 min (*trans*), ESI-MS: m/z calcd for $C_{15}H_{14}N_4O$, 267.12; found, 267.10.

(*E*)-2-((4-ethoxyphenyl)diazenyl)-6-methoxy-1-methyl-1*H*-benzo[*d*]imidazole (**18b**)

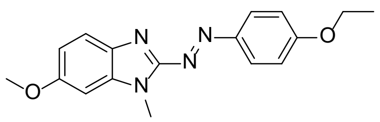

The reaction was done according to general procedure III from compound **17** (39.7 mg, 0.224 mmol) and **2b** (63.4 mg, 0.448 mmol). Purification was done using column chromatography with CH_2Cl_2 + 0.2% MeOH to give compound **18b** as an orange solid (35.9 mg, 51.2 %).

^{1}H NMR (400 MHz, $CDCl_3$) δ [ppm]: 8.06 (d, 2H), 7.75 (d, *J* = 8.9 Hz, 1H), 7.02 – 6.94 (m, 3H), 6.84 – 6.79 (m, 1H), 4.15 – 4.06 (m, 5H), 3.89 (s, 3H), 1.46 (t, *J* = 7.0 Hz, 3H). **^{13}C NMR** (101 MHz, $CDCl_3$) δ [ppm]: 162.9, 157.8, 155.3, 147.9, 137.0, 136.9, 126.0, 122.6, 115.0, 114.3, 92.4, 64.1, 55.9, 29.8, 14.8. **LC-MS analysis** (254 nm): t_r= 12.26 min (*trans*), ESI-MS: m/z calcd for $C_{17}H_{18}N_4O_2$, 311.15; found, 311.10.

(*E*)-2-((4-chlorophenyl)diazenyl)-6-methoxy-1-methyl-1*H*-benzo[*d*]imidazole (**18d**)

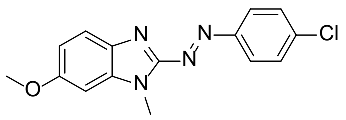

The reaction was done according to general procedure III from compound **17** (39.7 mg, 0.224 mmol) and **2d** (63.4 mg, 0.448 mmol). Purification was done using column chromatography with CH_2Cl_2 + 0.2% MeOH to give **18d** as an orange solid (50.7 mg, 75.0 %).

^{1}H NMR (400 MHz, $CDCl_3$) δ [ppm]: 8.07 – 7.99 (m, 2H), 7.78 (d, *J* = 9.0 Hz, 1H), 7.54 – 7.46 (m, 2H), 7.01 (dd, *J* = 9.0, 2.4 Hz, 1H), 6.84 (d, *J* = 2.4 Hz, 1H), 4.13 (s, 3H), 3.92 (s, 3H). **^{13}C NMR** (101 MHz, $CDCl_3$) δ [ppm]: 158.4, 154.8, 151.8, 138.4, 137.2, 129.7, 124.9, 123.1, 115.3, 92.3, 55.9, 30.0. **LC-MS analysis** (254 nm): t_r= 12.08 min (cis) and 13.08 min (trans), ESI-MS: m/z calcd for $C_{15}H_{13}ClN_4O$, 301.08; found, 301.05.

(*E*)-6-methoxy-1-methyl-2-((1-methyl-1*H*-pyrazol-3-yl)diazenyl)-1*H*-benzo[*d*]imidazole (**18pz**)

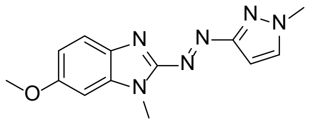

The reaction was done according to general procedure III from compound **17** (35.8 mg, 0.202 mmol) and **2pz** (44.9 mg, 0.404 mmol). Purification was done via reversed-phase flash chromatography using an isocratic gradient of water and MeOH. Compound **18pz** was obtained as an orange solid (44.7 mg, 81.9 %).

^{1}H NMR (400 MHz, CD_2Cl_2) δ [ppm]: 7.71 (d, *J* = 8.9 Hz, 1H), 7.45 (d, *J* = 2.4 Hz, 1H), 6.97 (dd, *J* = 8.9, 2.4 Hz, 1H), 6.89 (d, *J* = 2.4 Hz, 1H), 6.70 (d, *J* = 2.4 Hz, 1H), 4.07 (s, 3H), 4.04 (s, 3H), 3.92 (s, 3H). **^{13}C NMR** (101 MHz, CD_2Cl_2) δ [ppm]: 165.2, 158.7, 155.7, 137.7, 132.7, 122.9, 114.8, 96.5, 92.8, 56.4, 40.4, 30.8. **LC-MS analysis** (254 nm): t_r= 9.43 min (*cis*) and 10.20 min (*trans*), ESI-MS: m/z calcd for $C_{13}H_{14}N_6O$, 271.12; found, 271.15.

N-methyl-2,4-dinitroaniline (**20**)

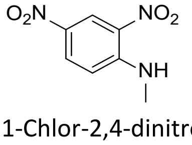

1-Chlor-2,4-dinitrobenzene (3.00 g, 14.8 mmol) was dissolved in EtOH and NEt_3 (2.50 mL, 17.8 mmol) and methylamine (33% in EtOH; 4.43 mL, 35.6 mmol) were added. The reaction was heated to 55°C overnight. The solvent was evaporated and the crude product was partially suspended in a mixture of CH_2Cl_2/EtOAc/MeOH (5:5:1). The solid was collected via vacuum filtration and dried in a desiccator. Compound **20** was obtained as a yellow solid (2.71 g, 92.8 %).

^{1}H NMR (400 MHz, DMSO-d$_6$) δ [ppm]: 8.86 (d, *J* = 2.8 Hz, 1H), 8.29 (dd, *J* = 9.6, 2.8 Hz, 1H), 7.59 (brs, 1H), 7.15 (d, *J* = 9.7 Hz, 1H), 3.06 (d, *J* = 5.0 Hz, 3H). **^{13}C NMR** (101 MHz, DMSO-d$_6$) δ [ppm]: 148.1, 134.2, 130.1, 123.6, 115.3, 30.4.

*N*1-methyl-4-nitrobenzene-1,2-diamine (**21**)

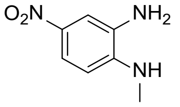

Compound **20** (2.71 g, 13.8 mmol) was dissolved in MeOH and heated to reflux. Na_2SxH_20 (10.7 g, 137 mmol) and $NaHCO_3$ (11.6 g, 137 mmol) were dissolved in water and added dropwise over 30 mins. The mixture was heated to reflux for another 30 mins and then cooled to RT. MeOH was evaporated and the aqueous phase was diluted with brine and extracted with EtOAc. The combined organic layers were washed with brine, dried over Na_2SO_4, filtered and concentrated under reduced pressure to give compound **21** as a red solid (1.70 g, 74.3 %). The compound was used for the next step without purification. **^{1}H NMR** (400 MHz, $CDCl_3$) δ [ppm]: 7.87 (dd, *J* = 8.8, 2.5 Hz, 1H), 7.63 (d, *J* = 2.5 Hz, 1H), 6.55 (d, *J* = 8.9 Hz, 1H), 2.97 (d, *J* = 5.2 Hz, 3H).

1-methyl-5-nitro-1*H*-benzo[*d*]imidazol-2-amine **(22)**

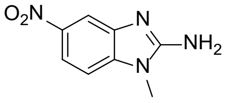

Compound **21** (500 mg, 2.99 mmol) and cyanogen bromide (950 mg, 8.97 mmol) were dissolved in acetonitrile/water (1:1) and heated to 50°C for 3 h. The reaction was quenched by addition of 1N NaOH (aq.). The aqueous phase was extracted with EtOAc and the combined organic layers were washed with 1 N NaOH and brine. The solvent was removed *in vacuo*. Purification was done using column chromatography with CH_2Cl_2/MeOH 97:3 + 0.1 % NEt_3. Compound **22** was obtained as a dark solid (236 mg, 41.1 %).
^{1}H NMR (400 MHz, DMSO-d$_6$) δ [ppm]: 7.92 (d, *J* = 2.2 Hz, 1H), 7.88 (dd, *J* = 8.6, 2.3 Hz, 1H), 7.31 (d, *J* = 8.6 Hz, 1H), 6.95 (brs, 2H), 3.58 (s, 3H).. **^{13}C NMR** (101 MHz, DMSO-d$_6$) δ [ppm]: 158.2, 142.7, 141.7, 140.3, 114.6, 109.4, 107.0, 28.9.

(*E*)-1-methyl-5-nitro-2-(phenyldiazenyl)-1*H*-benzo[*d*]imidazole (**23a**)

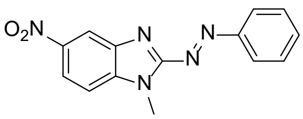

The reaction was done according to general procedure II from compound **22** (10.0 mg, 52.0 µmol) and nitrosobenzene (11.2 mg, 104 µmol). Purification was done via preparative TLC with petroleum ether/EtOAc 4:1 and yielded **23a** as an orange solid (5.57 mg, 19.6 %).
^{1}H NMR (400 MHz, $CDCl_3$) δ 8.82 (d, *J* = 2.1 Hz, 1H), 8.33 (dd, *J* = 9.0, 2.2 Hz, 1H), 8.19 – 8.12 (m, 2H), 7.68 – 7.55 (m, 4H), 4.27 (s, 3H). **^{13}C NMR** (101 MHz, $CDCl_3$) δ 157.5, 153.3, 145.0, 141.4, 139.9, 134.0, 129.6, 124.3, 120.0, 118.8, 110.3, 30.6. **LC-MS analysis** (254 nm): t_r= 9.72min (*cis*) and 11.09 min (*trans*), ESI-MS: m/z calcd for $C_{14}H_{11}N_5O_2$, 282.09; found, 282.05.

(*E*)-2-((4-ethoxyphenyl)diazenyl)-1-methyl-5-nitro-1*H*-benzo[*d*]imidazole (**23b**)

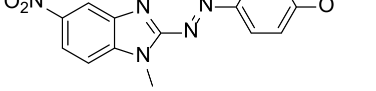

The reaction was done according to general procedure III from compound **22** (20.0 mg, 104 µmol) and **2b** (31.5 mg, 208 µmol). Purification was done via column chromatography with CH_2Cl_2 + 0.5 % MeOH and yielded **23b** as an orange solid (3.75 mg, 11.1 %).
^{1}H NMR (400 MHz, $CDCl_3$) δ [ppm]: 8.78 (d, *J* = 2.1 Hz, 1H), 8.30 (dd, *J* = 8.9, 2.1 Hz, 1H), 8.18 – 8.11 (m, 2H), 7.54 (d, *J* = 9.0 Hz, 1H), 7.09 – 7.02 (m, 2H), 4.22 (s, 3H), 4.18 (q, *J* = 7.0 Hz, 2H), 1.48 (t, *J* = 7.0 Hz, 3H). **^{13}C NMR** (101

MHz, $CDCl_3$) δ [ppm]: 164.3, 158.1, 147.9, 144.8, 141.5, 140.0, 127.0, 119.6, 118.3, 115.3, 110.0, 64.4, 30.5, 29.8, 14.8. **LC-MS analysis** (254 nm): t_r= 11.73 min (*trans*), ESI-MS: m/z calcd for $C_{16}H_{15}N_5O_3$, 326.12; found, 326.10.

(*E*)-2-((3-ethoxyphenyl)diazenyl)-1-methyl-5-nitro-1*H*-benzo[*d*]imidazole (**23c**)

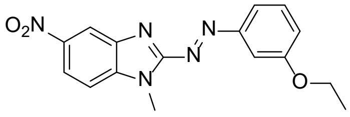

The reaction was done according to general procedure III from compound **22** (25.0 mg, 130 µmol) and **2c** (39.3 mg, 260 µmol). The crude solid was recrystallized from CH_2Cl_2/MeOH and yielded **23c** as an orange solid (14.2 mg, 33.6 %).

^{1}H NMR (400 MHz, CD_2Cl_2) δ [ppm]: 8.74 (d, *J* = 2.1 Hz, 1H), 8.31 (dd, *J* = 9.0, 2.2 Hz, 1H), 7.78 – 7.70 (m, 1H), 7.66 – 7.58 (m, 2H), 7.51 (t, *J* = 8.0 Hz, 1H), 7.22 – 7.16 (m, 1H), 4.22 (s, 3H), 4.16 (q, *J* = 6.9 Hz, 2H), 1.46 (t, *J* = 7.0 Hz, 3H). **^{13}C NMR** (101 MHz, CD2Cl2) δ [ppm]: 160.5, 158.1, 155.0, 145.2, 141.9, 140.6, 130.7, 121.3, 120.2, 118.8, 118.4, 111.0, 108.2, 64.6, 31.2, 15.1. **LC-MS analysis** (254 nm): t_r= 11.97 min (*cis*)and 13.08 min (*trans*), ESI-MS: m/z calcd for $C_{16}H_{15}N_5O_3$, 326.12; found, 326.10.

(*E*)-2-((4-chlorophenyl)diazenyl)-1-methyl-5-nitro-1*H*-benzo[*d*]imidazole (**23d**)

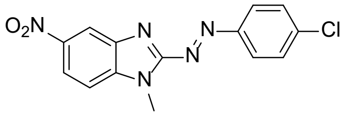

The reaction was done according to general procedure III from compound **22** (25.0 mg, 130 µmol) and **2d** (36.8 mg, 260 µmol). Purification was done via preparative TLC with CH_2Cl_2 + 0.5 % MeOH and yielded **23d** as an orange solid (16.2 mg, 39.4 %).

^{1}H NMR (400 MHz, CD_2Cl_2) δ [ppm]: 8.73 (d, *J* = 2.2 Hz, 1H), 8.30 (dd, *J* = 9.0, 2.0 Hz, 1H), 8.10 – 8.04 (m, 2H), 7.64 – 7.56 (m, 3H), 4.21 (s, 3H). **^{13}C NMR** (101 MHz, CD_2Cl_2) δ [ppm]: 158.0, 152.2, 145.2, 141.8, 140.6, 140.4, 130.4, 125.8, 120.3, 118.9, 111.1, 31.2. **LC-MS analysis** (254 nm): t_r= 12.19 min (*cis*) and 13.09 min (*trans*), ESI-MS: m/z calcd for $C_{14}H_{10}ClN_5O_2$, 316.05; found, 316.00.

1-methyl-6-nitro-1H-benzo[d]imidazol-2-amine (**25**)

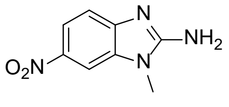

N1-methyl-5-nitrobenzene-1,2-diamine (151 mg, 903 µmol mmol) and cyanogen bromide (197 mg, 1.86 mmol) were dissolved in acetonitrile/water (1:1) and heated to 55°C for 3 h and then continued to stir at RT overnight. The reaction was quenched by addition of 1N NaOH (aq.). The formed precipitate was collected via vacuum filtration and washed with water and CH_2Cl_2 and was dried in a desiccator overnight. Compound **25** was obtained as a yellow solid (104 mg, 59.9 %).

^{1}H NMR (400 MHz, DMSO-d_6) δ [ppm]: 8.05 (d, *J* = 2.3 Hz, 1H), 7.93 (dd, *J* = 8.7, 2.4 Hz, 1H), 7.25 – 7.14 (m, 3H), 3.59 (s, 3H). **^{13}C NMR** (101 MHz, DMSO-d_6) δ [ppm]: 159.6, 149.6, 138.8, 134.6, 117.8, 113.4, 103.5, 28.7.

(*E*)-1-methyl-6-nitro-2-(phenyldiazenyl)-1*H*-benzo[*d*]imidazole (**26a**)

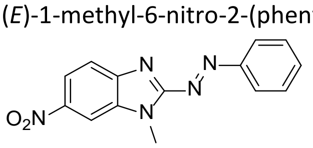

The reaction was done according to general procedure II using compound **25** (31.9 mg, 166 µmol) and nitrosobenzene (22.2 mg, 207 µmol). Purification was done via preparative TLC with CH_2Cl_2 + 2 % EtOAc and compound **26a** was obtained as an orange solid (10.6 mg, 22.7 %).

^{1}H NMR (400 MHz, $CDCl_3$) δ [ppm]: 8.51 (d, *J* = 2.2 Hz, 1H), 8.28 (dd, *J* = 9.0, 2.2 Hz, 1H), 8.20 – 8.13 (m, 2H), 7.98 (d, *J* = 8.9 Hz, 1H), 7.67 – 7.57 (m, 3H), 4.31 (s, 3H). **^{13}C NMR** (101 MHz, $CDCl_3$) δ [ppm]: 153.4, 146.4, 144.6, 135.4,

134.2, 129.7, 124.4, 122.3, 119.7, 107.3, 30.6. **LC-MS analysis** (254 nm): t_r= 11.00 min (*cis*) and 13.12 min (*trans*), ESI-MS: m/z calcd for $C_{14}H_{11}N_5O_2$, 282.09; found, 282.05.

(*E*)-2-((4-ethoxyphenyl)diazenyl)-1-methyl-6-nitro-1*H*-benzo[*d*]imidazole (**26b**)

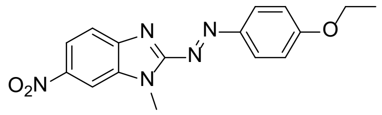

The reaction was done according to general procedure III using compound **25** (29.7 mg, 155 µmol) and **2b** (46.7 mg, 309 µmol). Purification was done via preparative TLC with CH_2Cl_2 + 0.25 % MeOH and **So-223** was obtained as an orange solid (1.25 mg, 2.49 %).

^{1}H NMR (400 MHz, $CDCl_3$) δ [ppm]: 8.47 (d, *J* = 2.2 Hz, 1H), 8.26 (dd, *J* = 9.0, 2.2 Hz, 1H), 8.18 – 8.13 (m, 2H), 7.94 (d, *J* = 9.0 Hz, 1H), 7.08 – 7.02 (m, 2H), 4.26 (s, 3H), 4.19 (q, *J* = 7.0 Hz, 2H), 1.49 (t, *J* = 7.0 Hz, 3H).). **^{13}C NMR** (101 MHz, $CDCl_3$) δ [ppm]: 164.29, 147.80, 146.52, 126.93, 121.62, 119.30, 115.19, 106.86, 64.26, 30.27, 14.67. **LC-MS analysis** (254 nm): t_r= 11.85 min (*cis*) and 12.90 min (*trans*), ESI-MS: m/z calcd for $C_{16}H_{15}N_5O_3$, 326.12; found, 326.10.

NMR Spectra

(*E*)-2-(phenyldiazenyl)-1*H*-benzo[*d*]imidazole (**3a**)

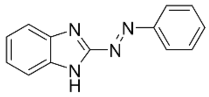

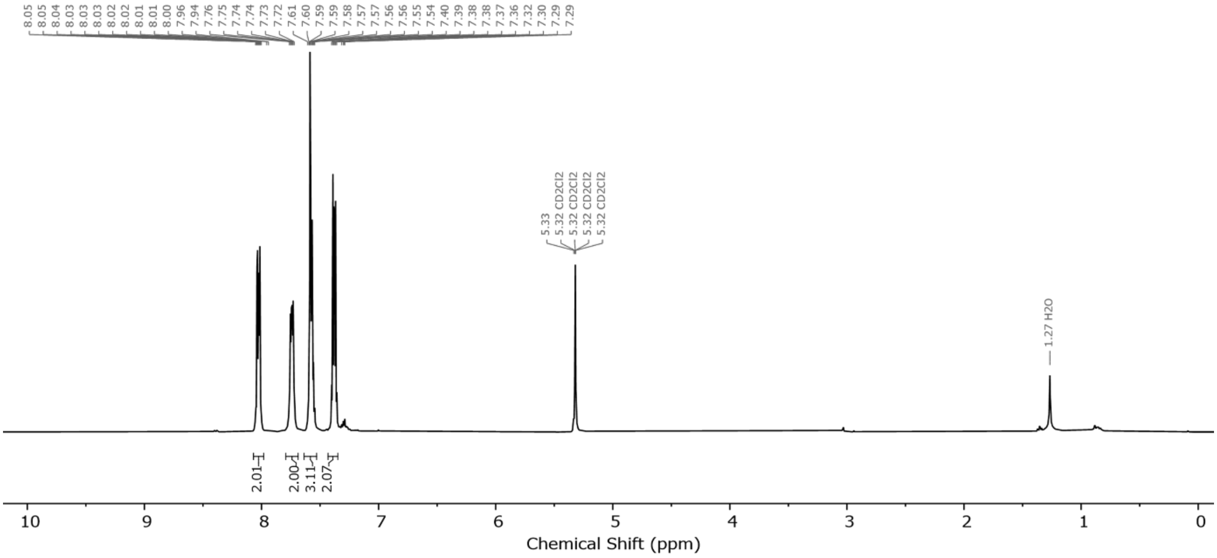

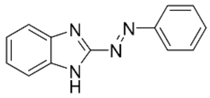

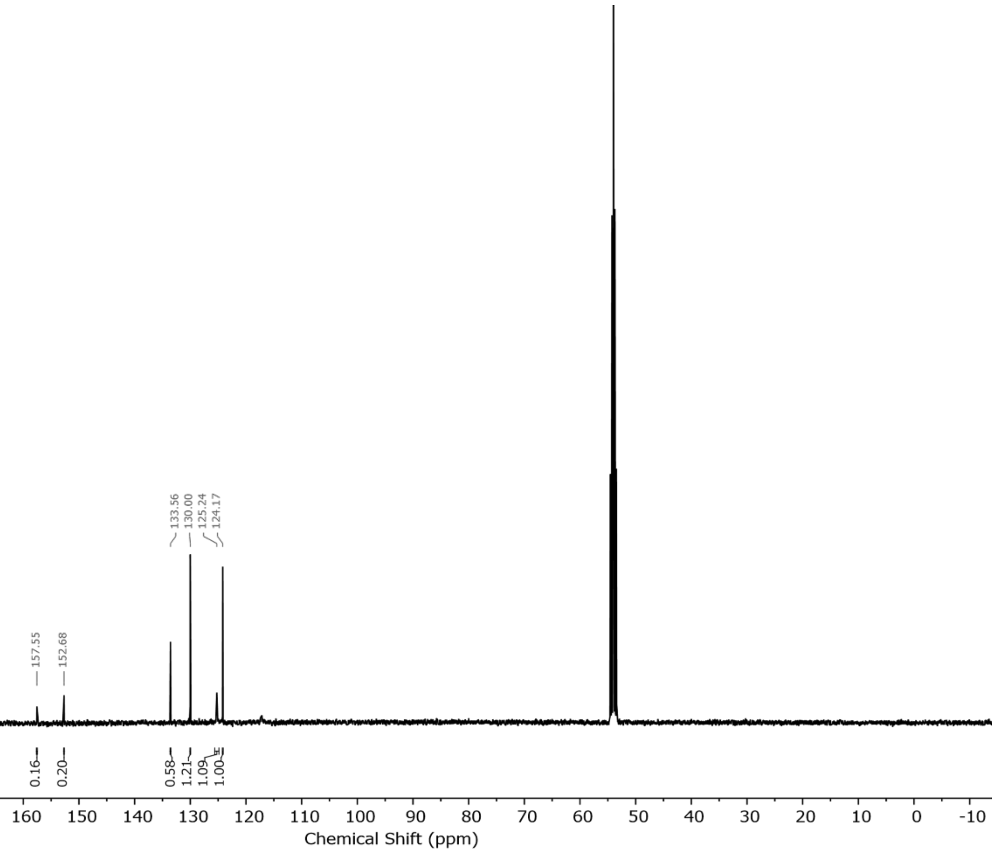

(*E*)-2-((4-ethoxyphenyl)diazenyl)-1*H*-benzo[*d*]imidazole (**3b**)

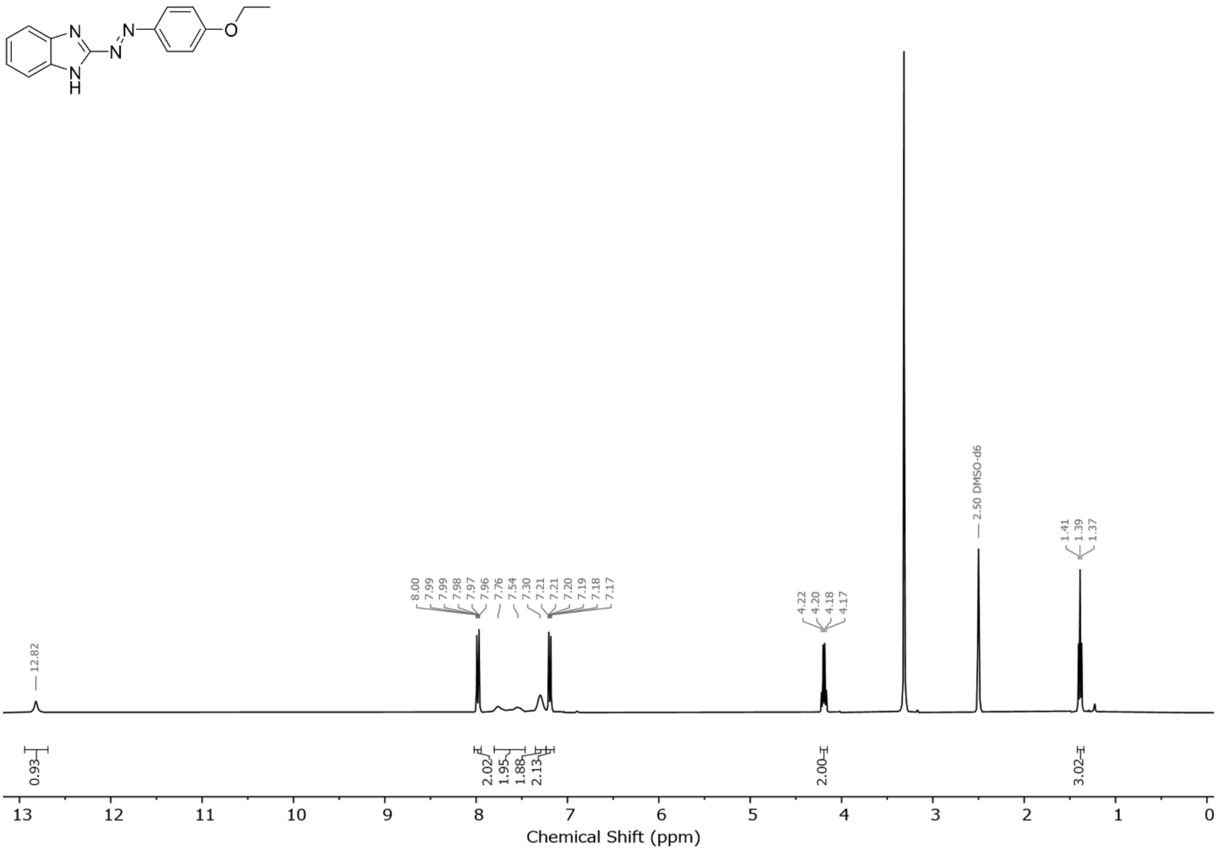

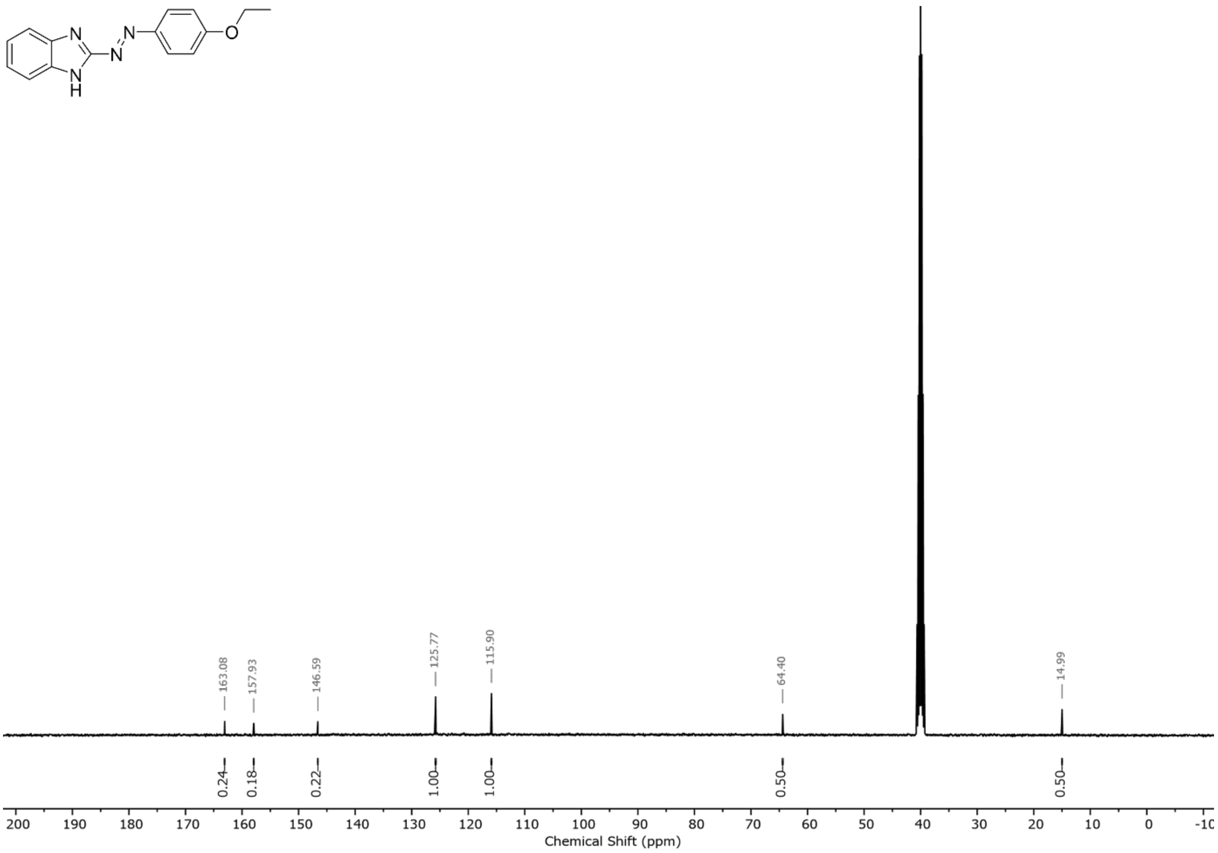

(*E*)-2-((3-ethoxyphenyl)diazenyl)-1*H*-benzo[*d*]imidazole (**3c**)

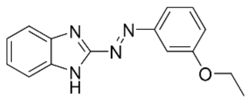

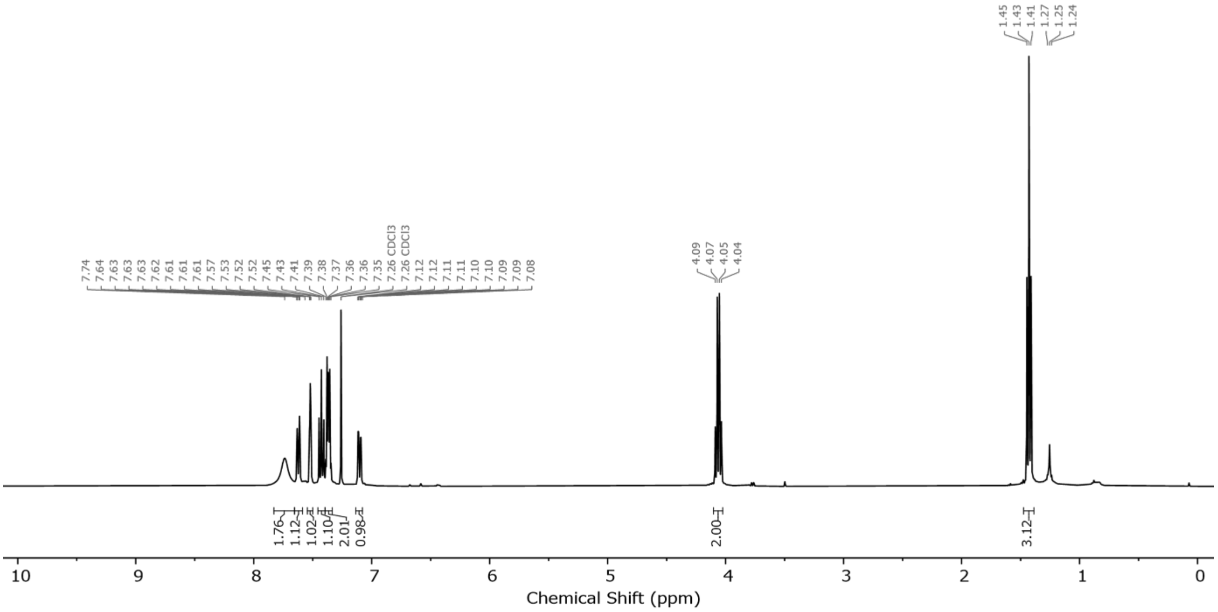

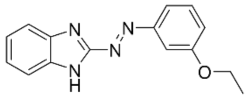

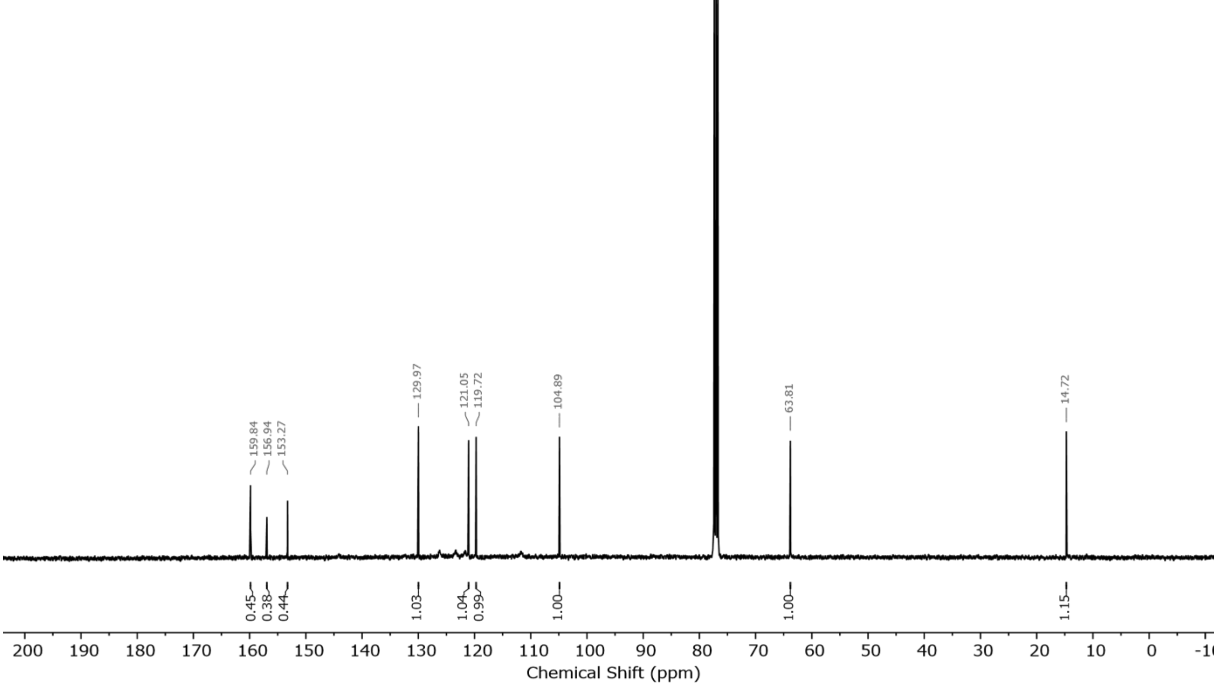

(*E*)-2-((4-chlorophenyl)diazenyl)-1*H*-benzo[*d*]imidazole (**3d**)

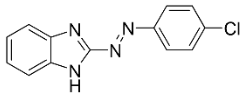

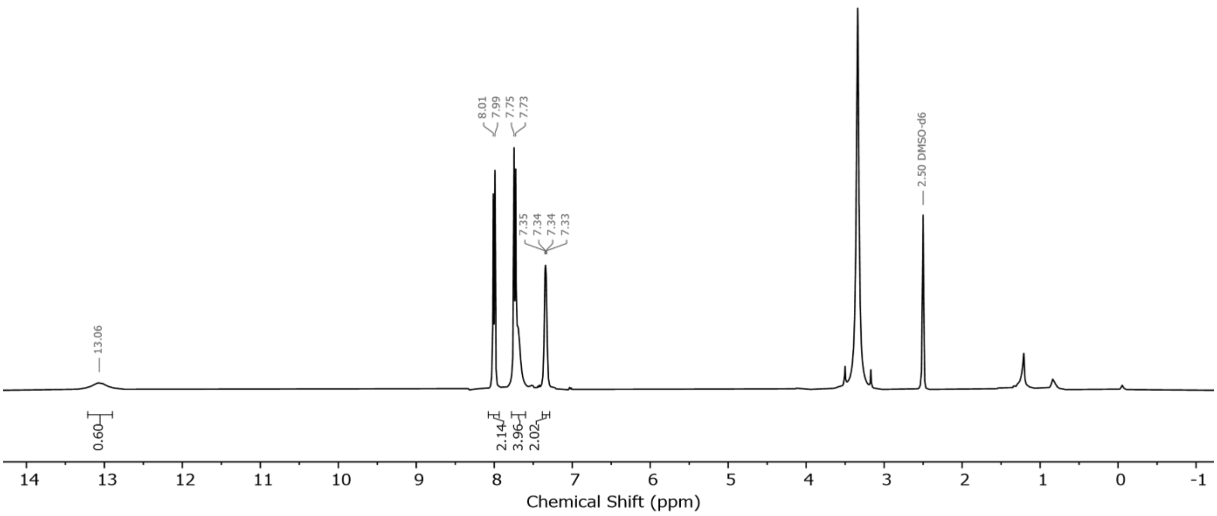

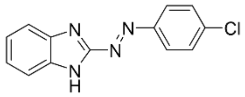

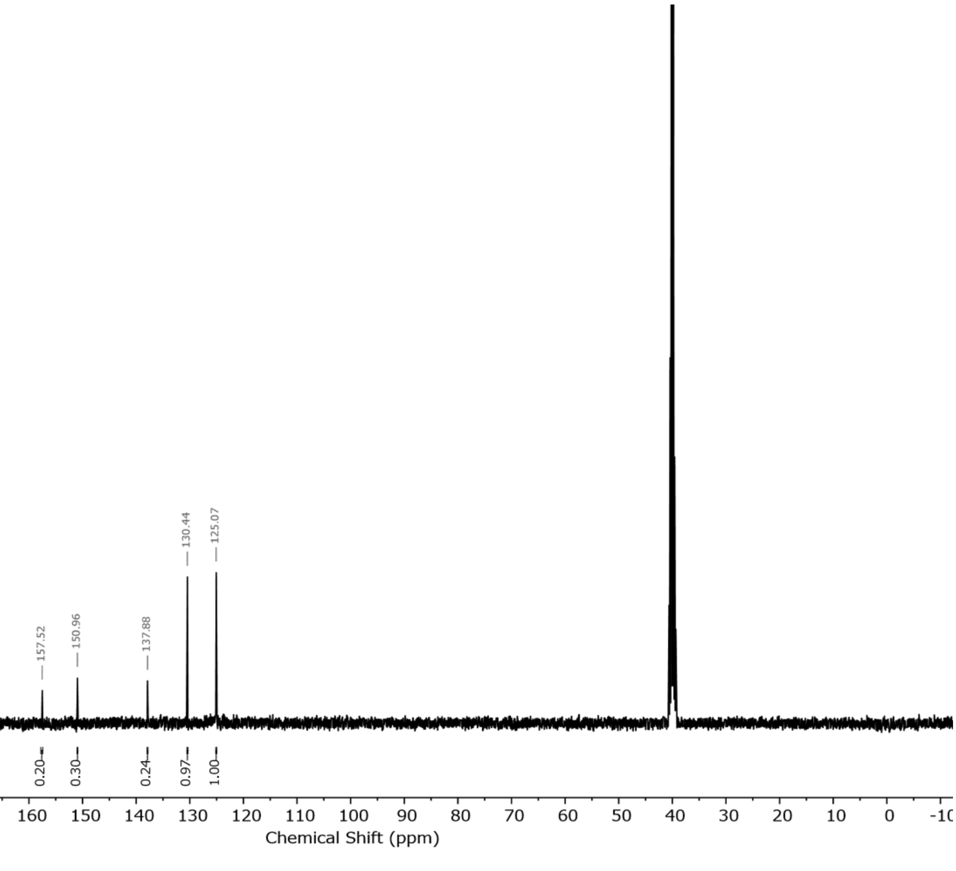

(*E*)-5-methoxy-2-(phenyldiazenyl)-1*H*-benzo[*d*]imidazole (**13aH**)

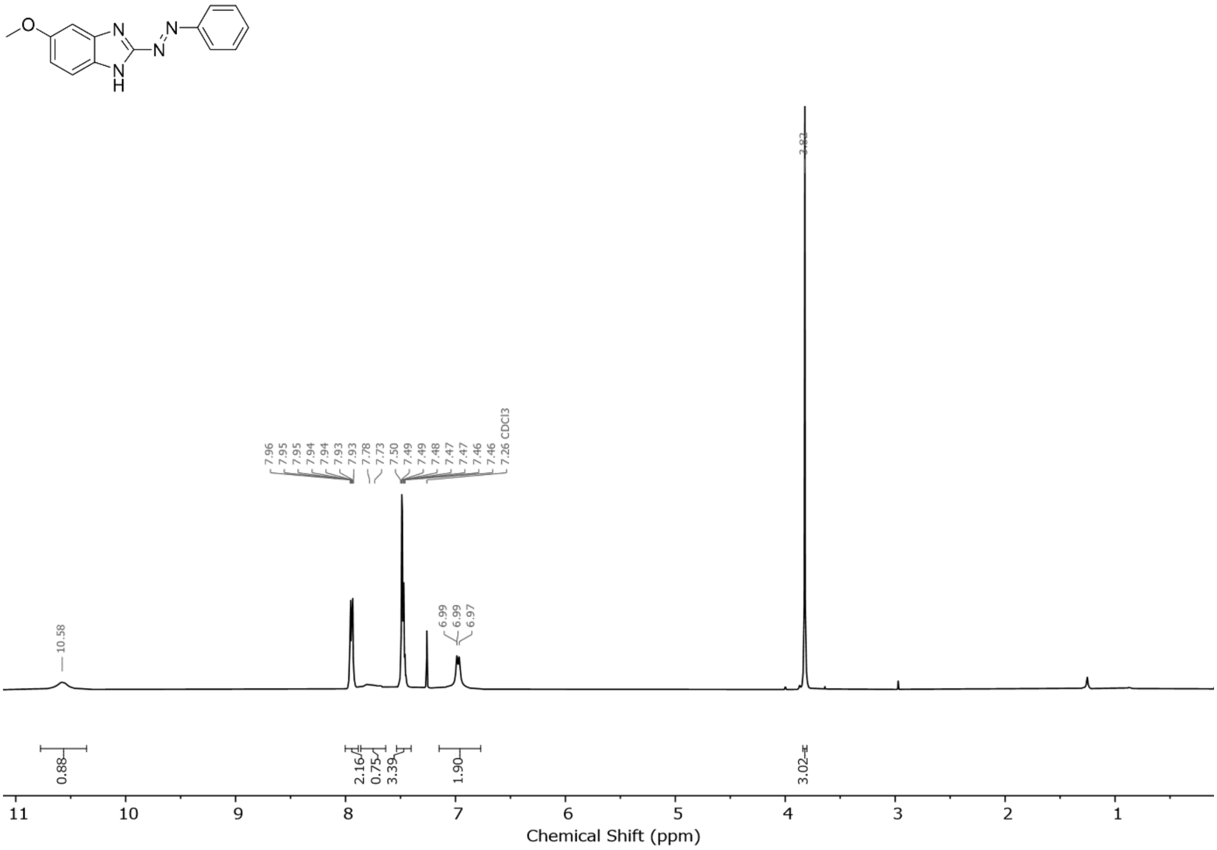

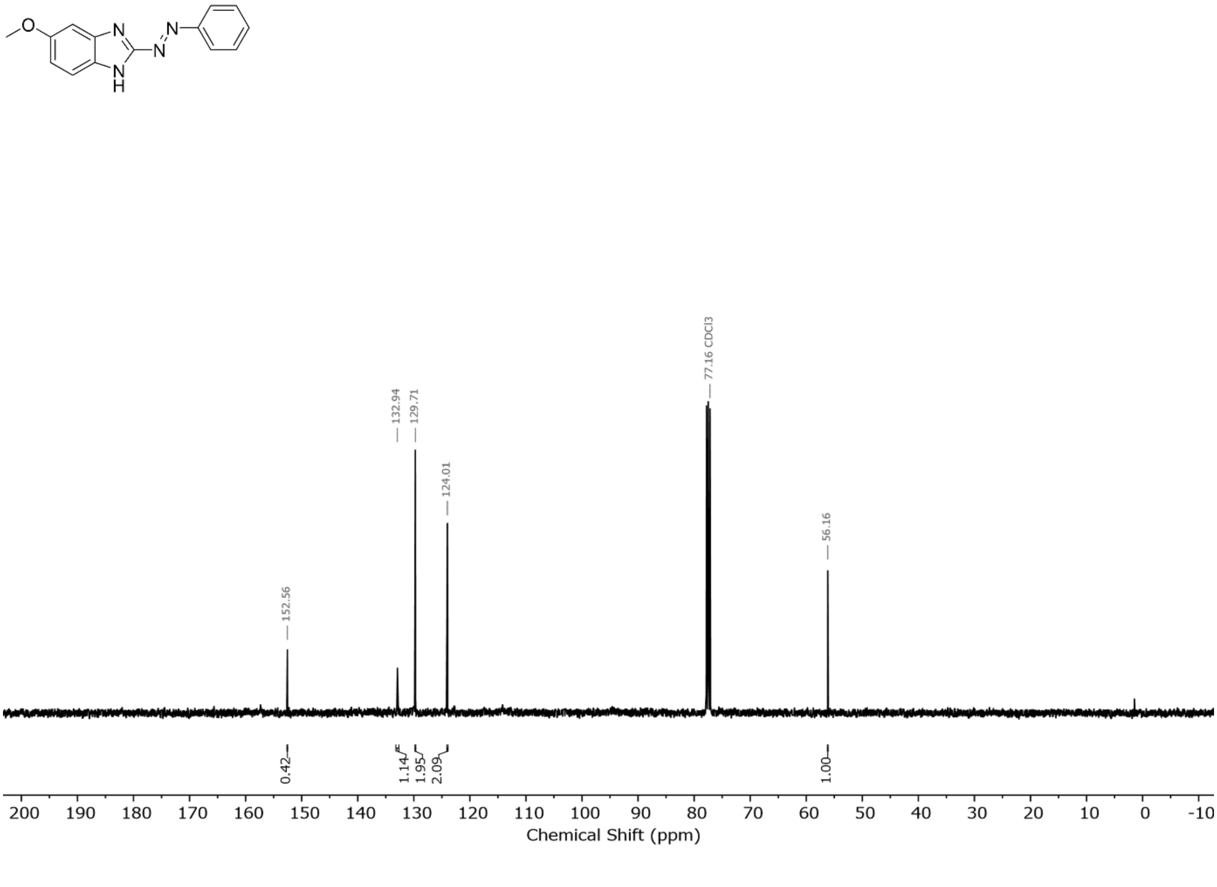

(*E*)-2-((1-methyl-1*H*-pyrazol-3-yl)diazenyl)-1*H*-benzo[*d*]imidazole (**3pz**)

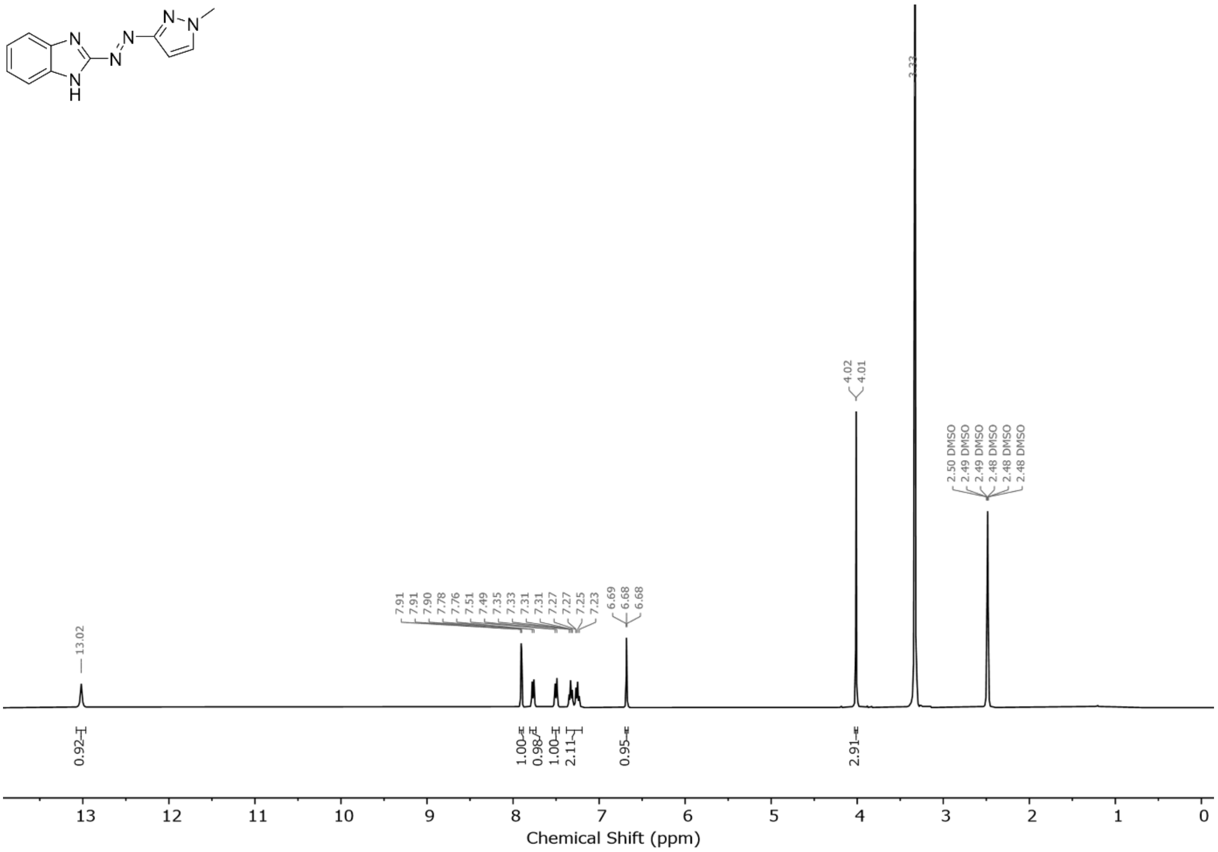

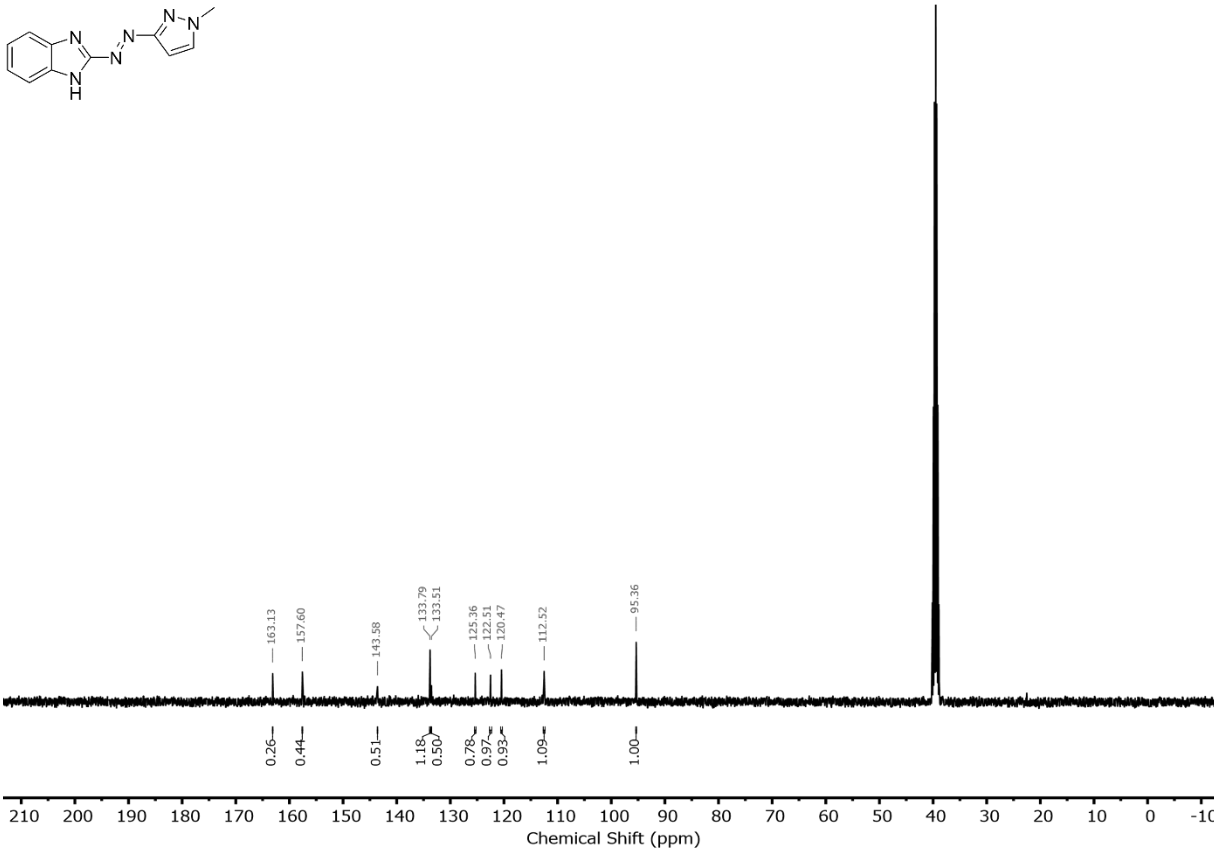

(*E*)-1-methyl-2-(phenyldiazenyl)-1*H*-benzo[*d*]imidazole (**8a**)

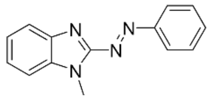

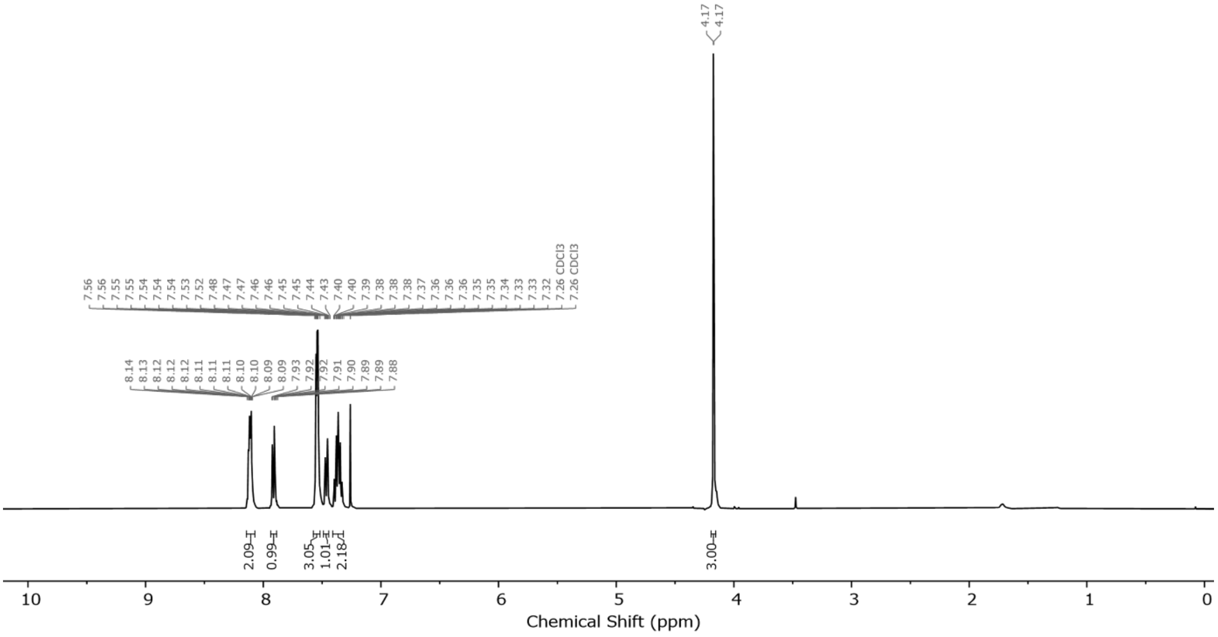

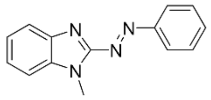

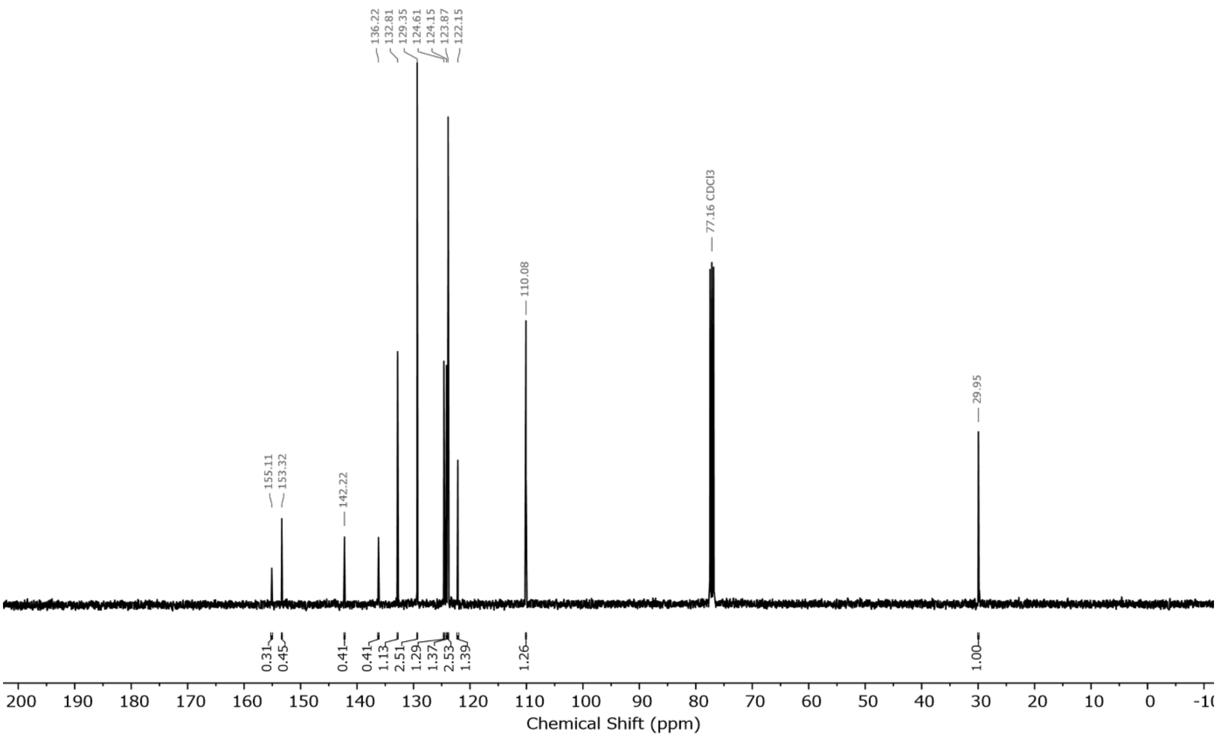

(*E*)-2-((4-ethoxyphenyl)diazenyl)-1-methyl-1*H*-benzo[*d*]imidazole (**8b**)

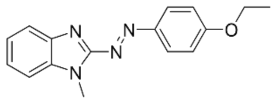

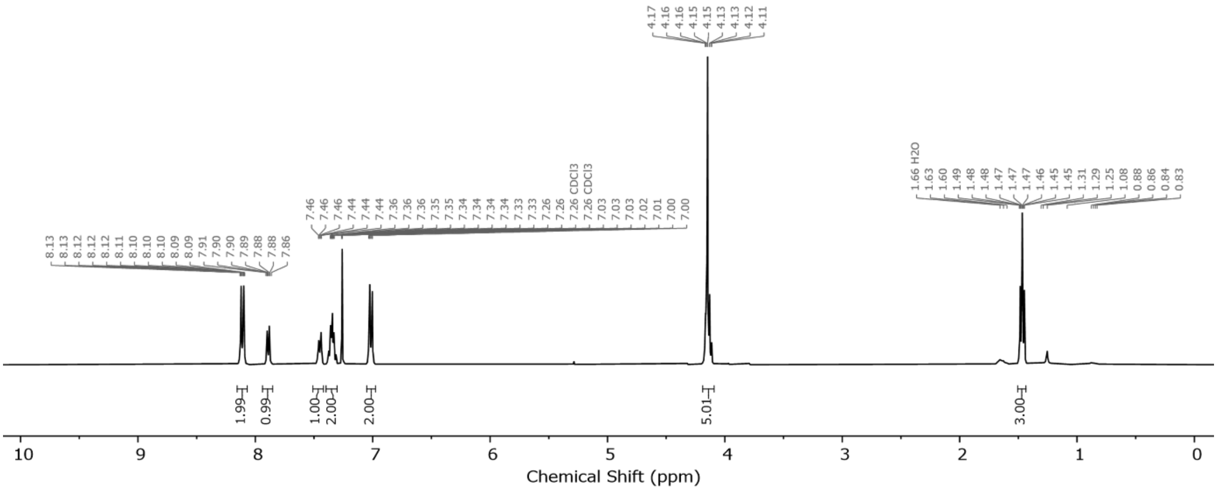

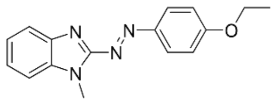

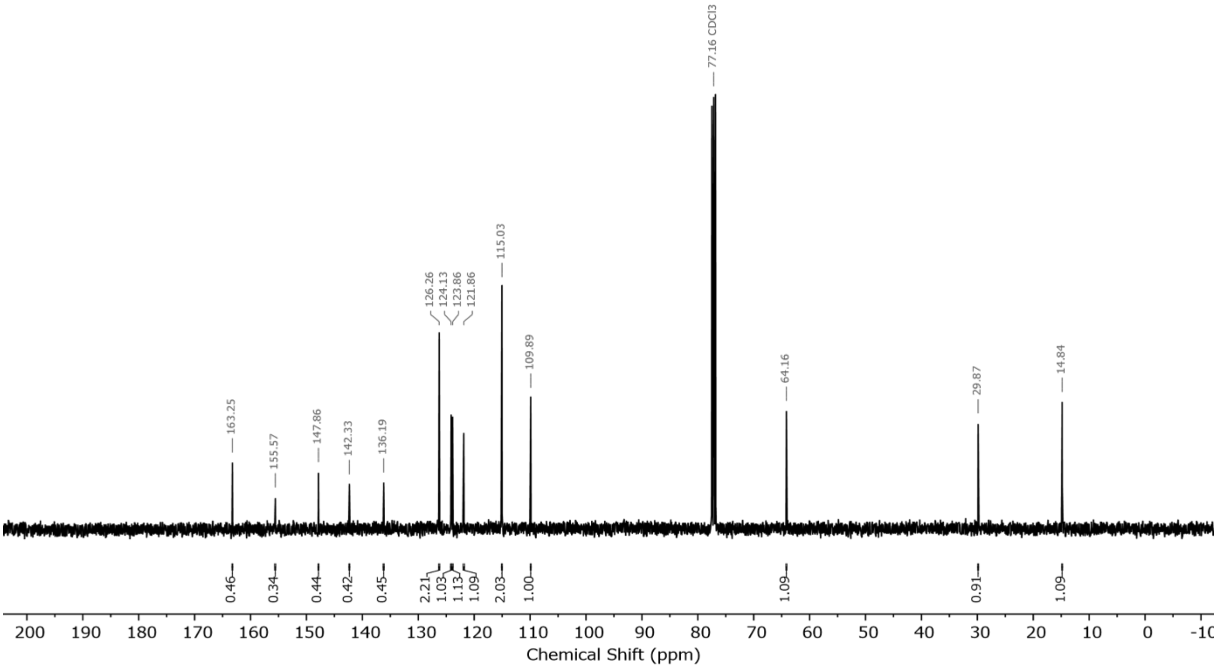

(*E*)-2-((4-chlorophenyl)diazenyl)-1-methyl-1*H*-benzo[*d*]imidazole (**8d**)

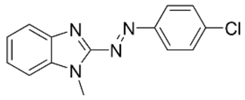

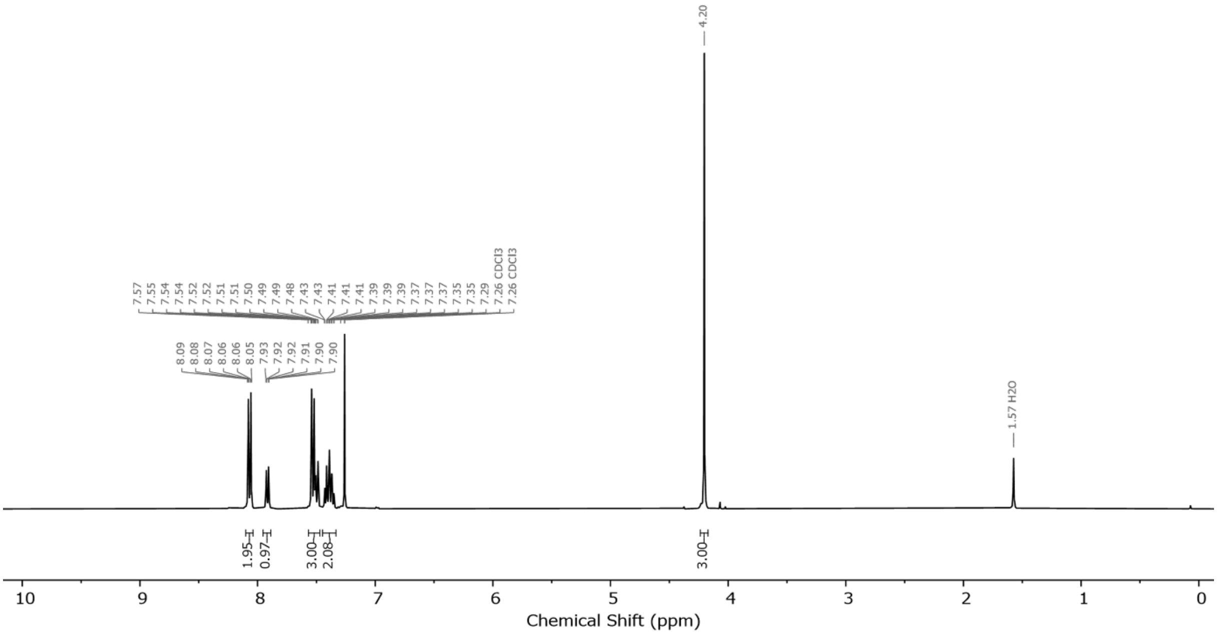

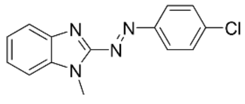

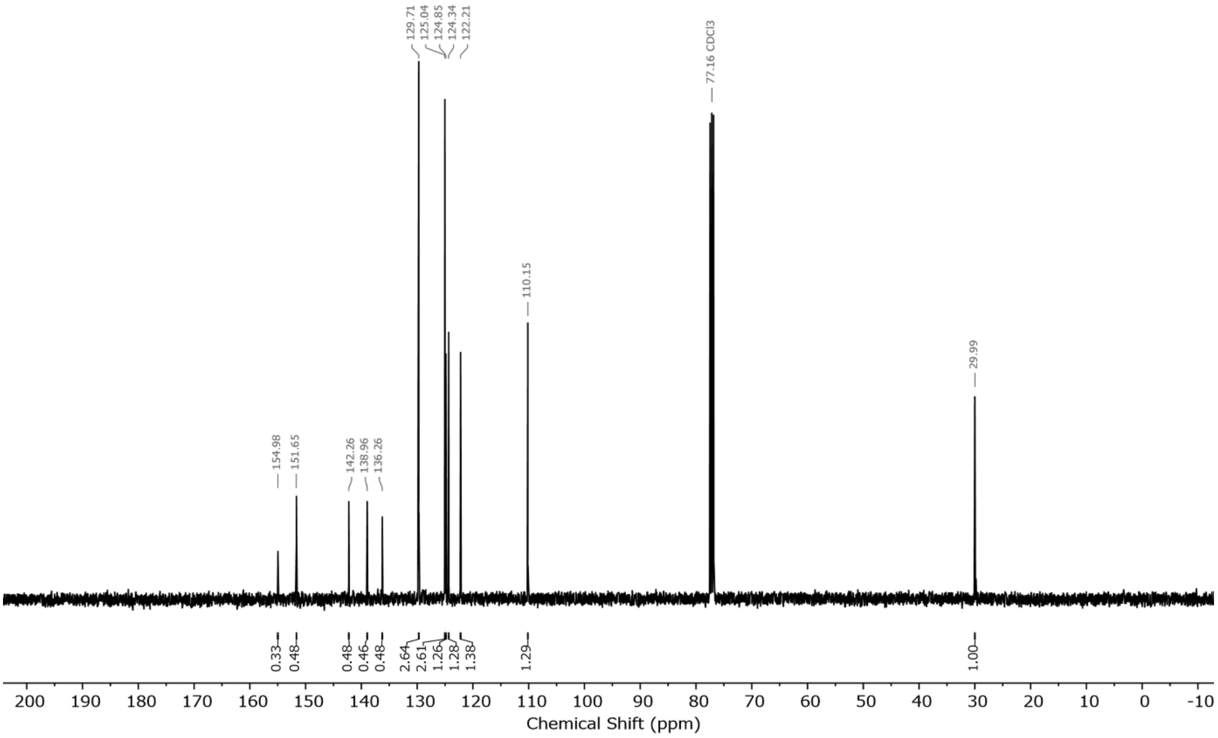

(*E*)-1-methyl-2-((4-nitrophenyl)diazenyl)-1*H*-benzo[*d*]imidazole (**8e**)

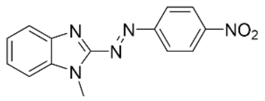

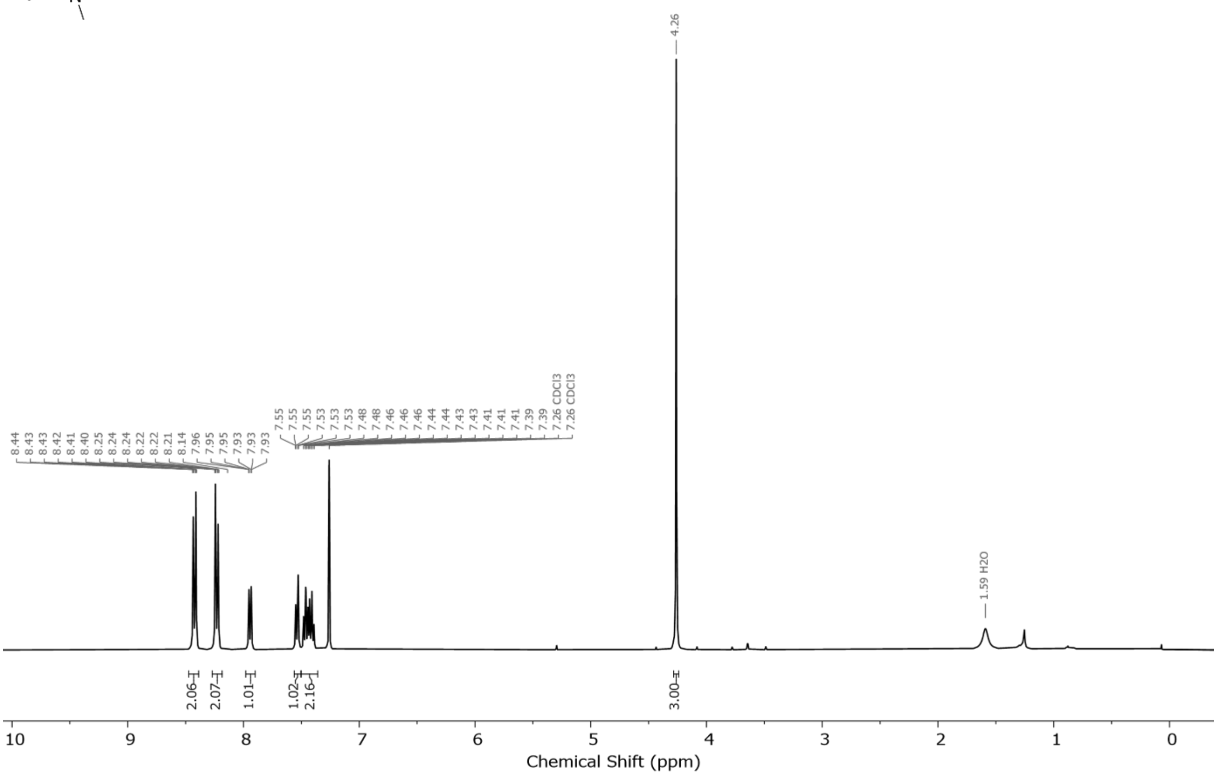

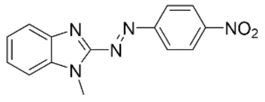

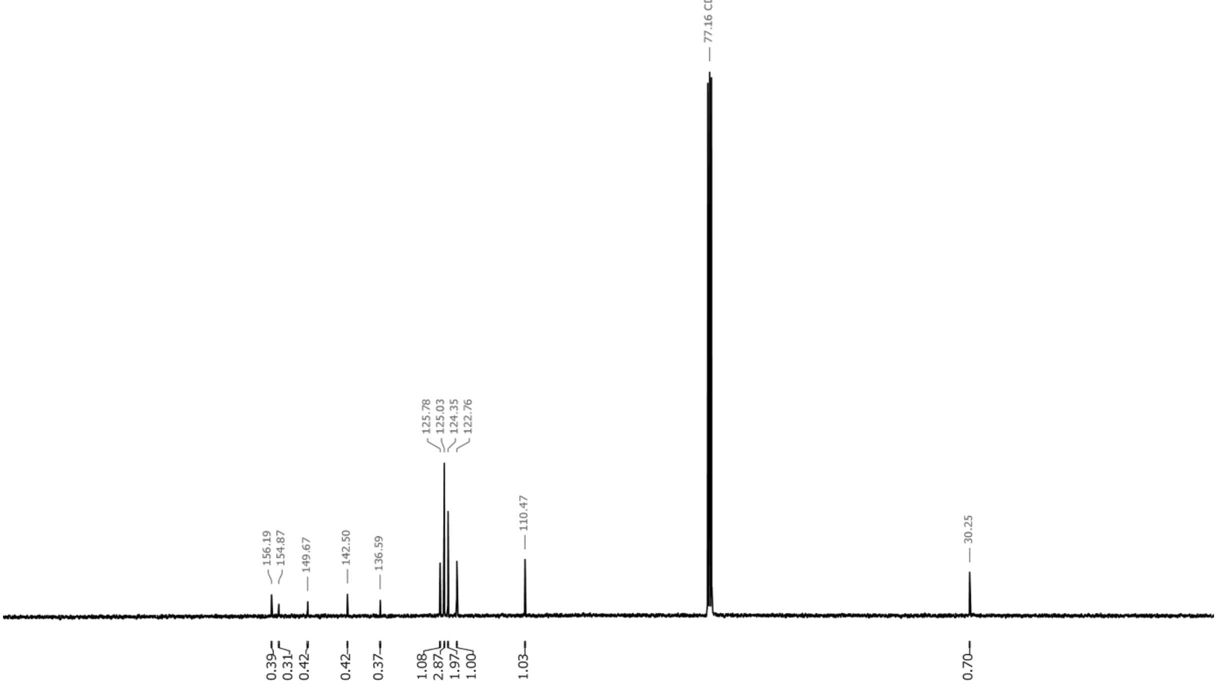

(*E*)-1-methyl-2-((1-methyl-1*H*-pyrazol-3-yl)diazenyl)-1*H*-benzo[*d*]imidazole (**8pz**)

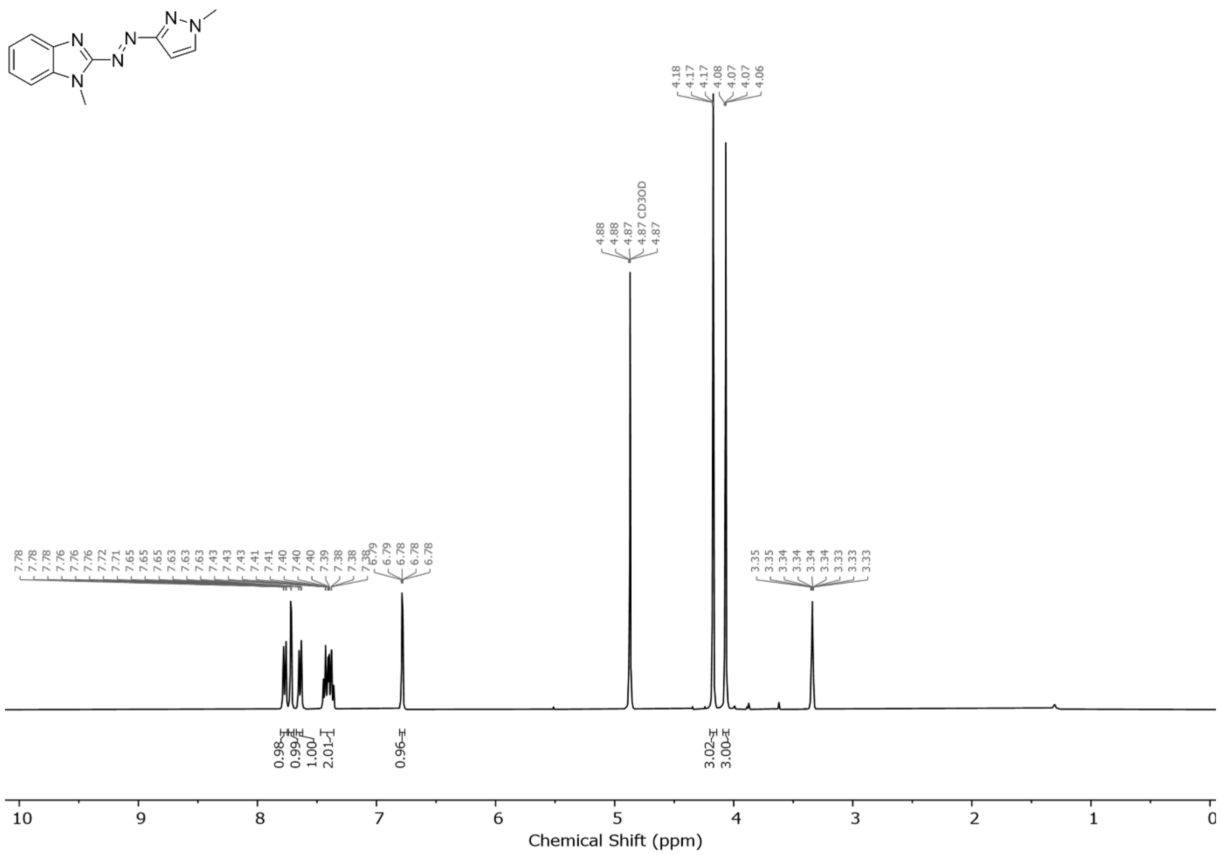

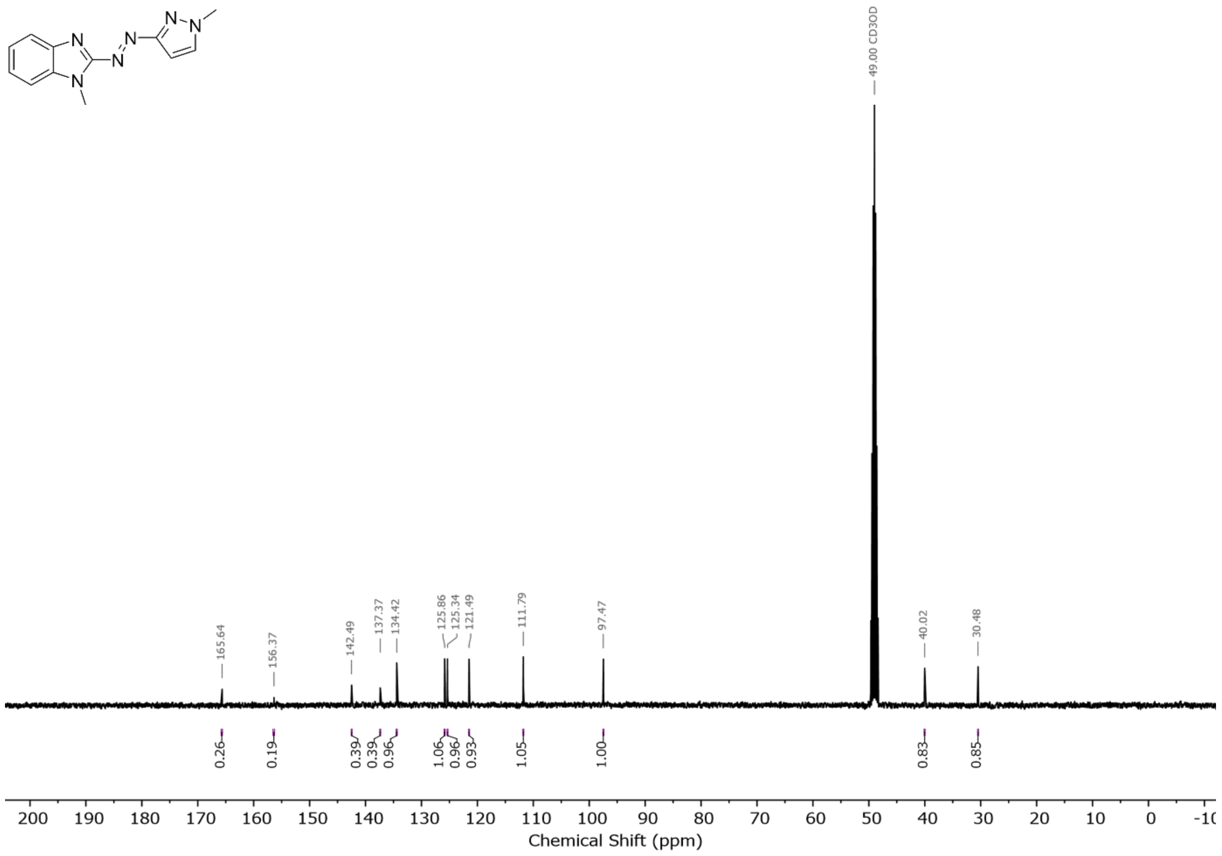

(*E*)-5-methoxy-1-methyl-2-(phenyldiazenyl)-1*H*-benzo[*d*]imidazole (**13a**)

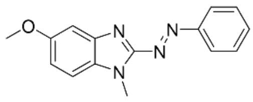

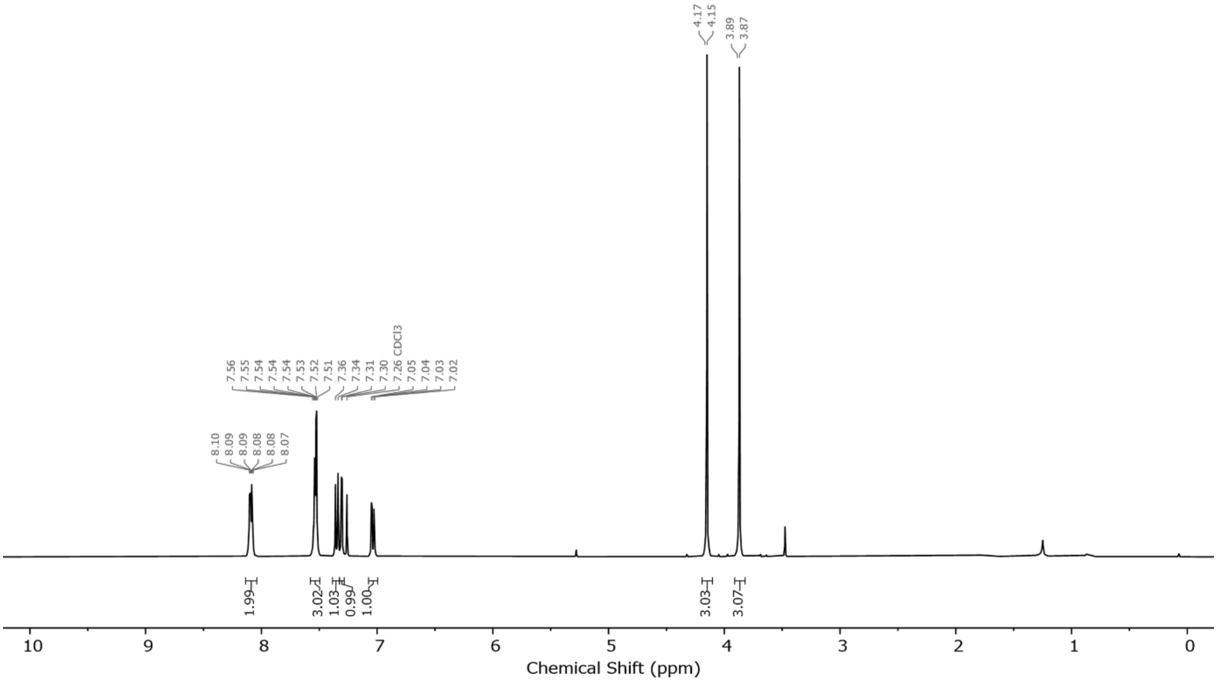

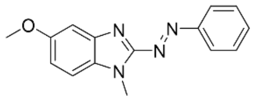

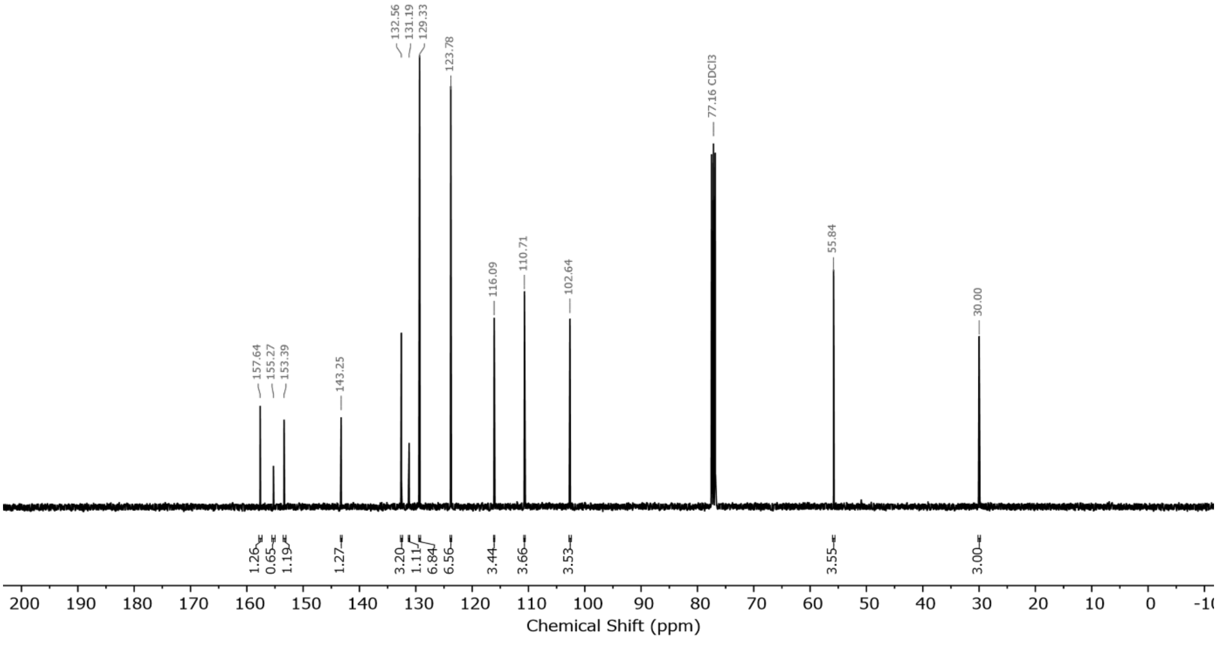

(*E*)-2-((4-ethoxyphenyl)diazenyl)-5-methoxy-1-methyl-1*H*-benzo[*d*]imidazole (**13b**)

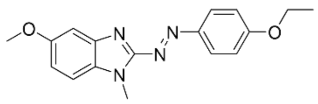

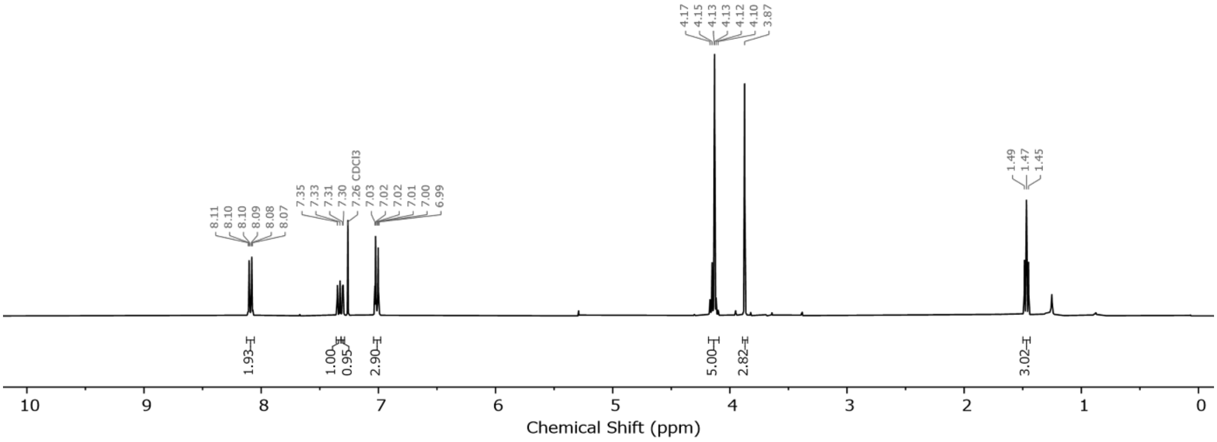

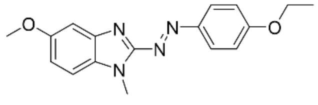

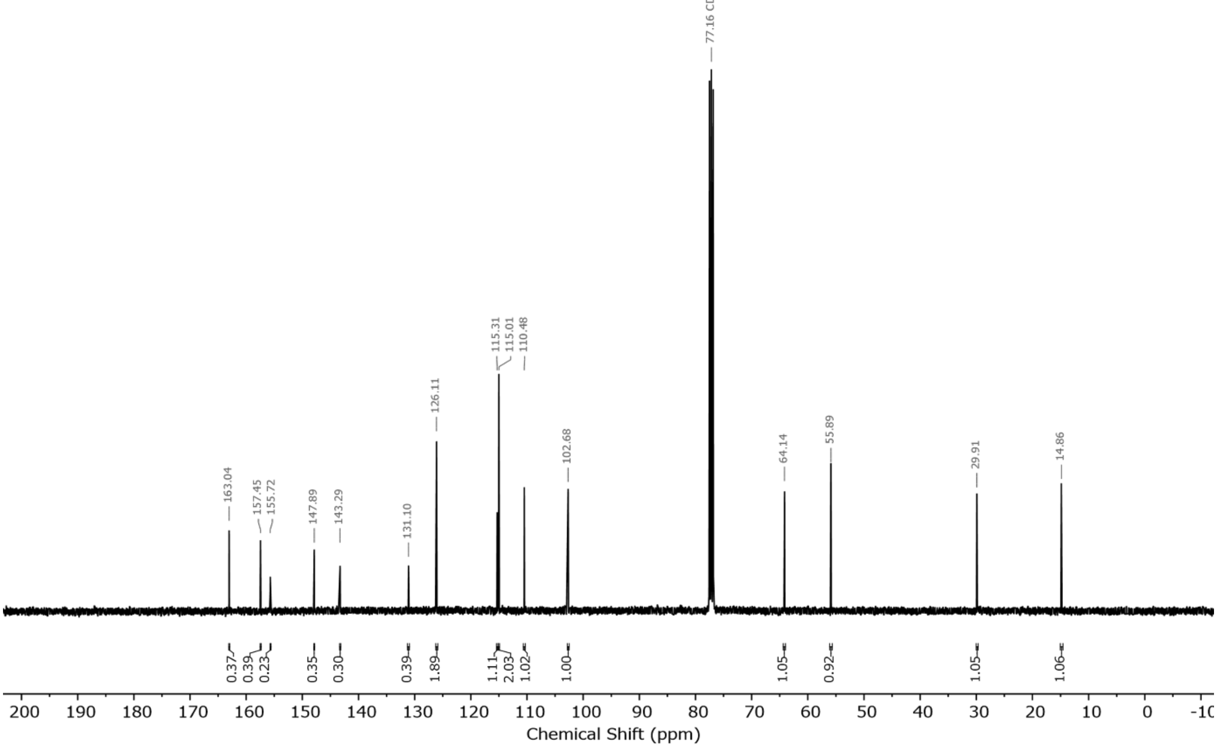

(*E*)-2-((3-ethoxyphenyl)diazenyl)-5-methoxy-1-methyl-1*H*-benzo[*d*]imidazole (**13c**)

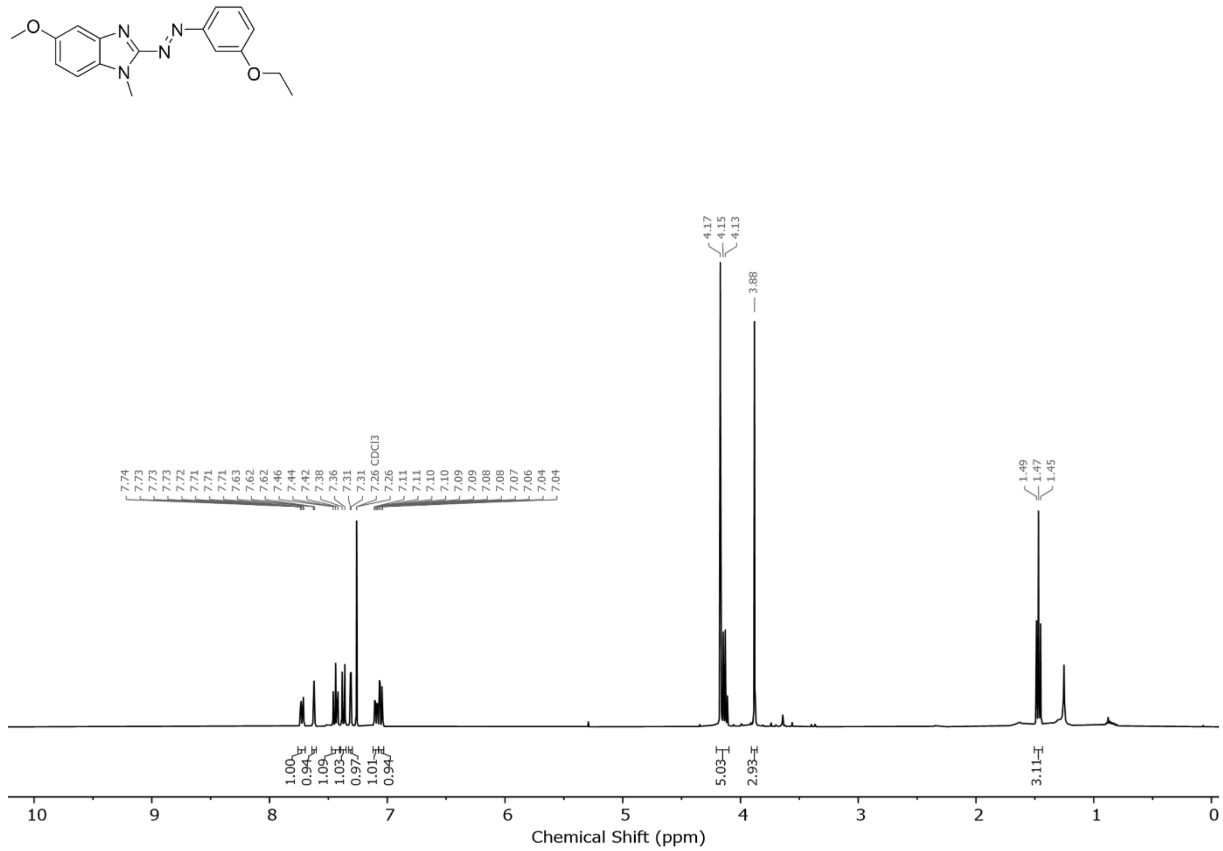

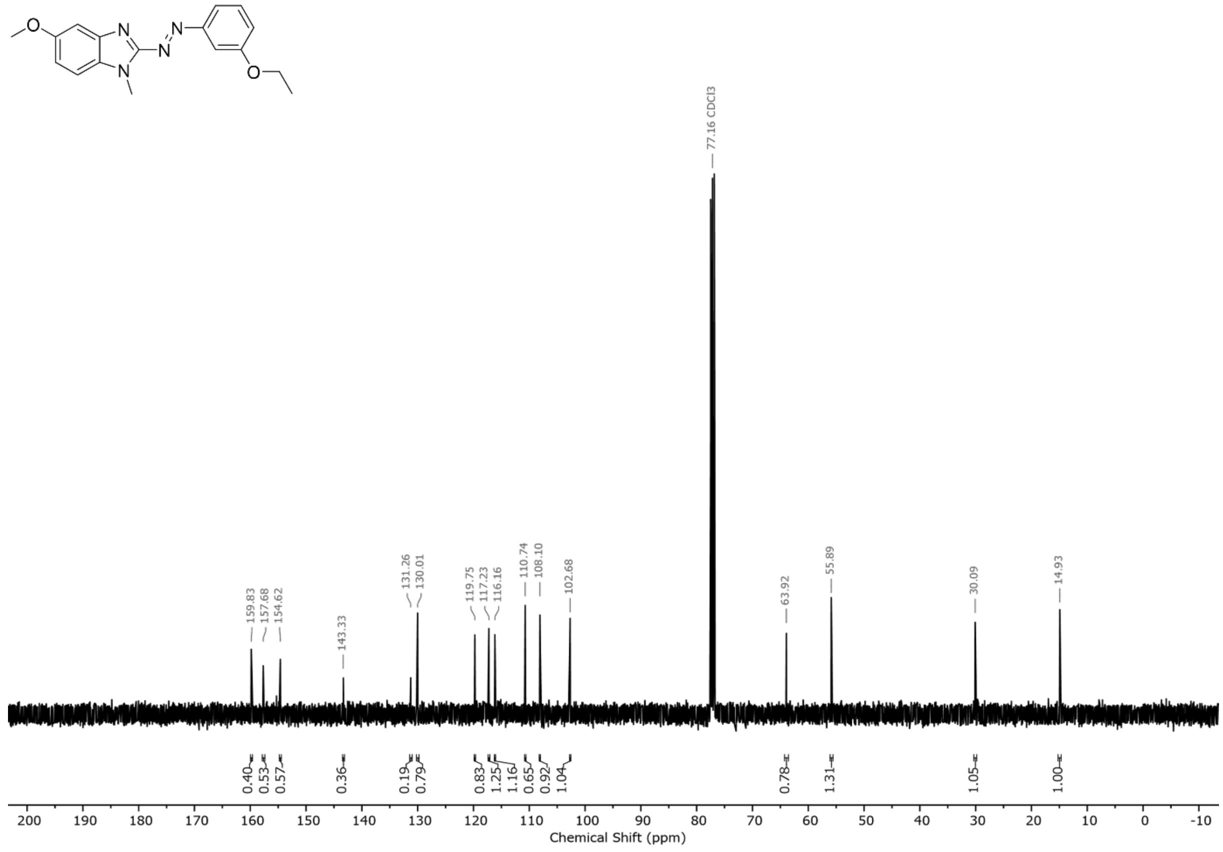

(*E*)-2-((4-chlorophenyl)diazenyl)-5-methoxy-1-methyl-1*H*-benzo[*d*]imidazole **(13d)**

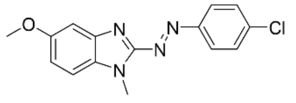

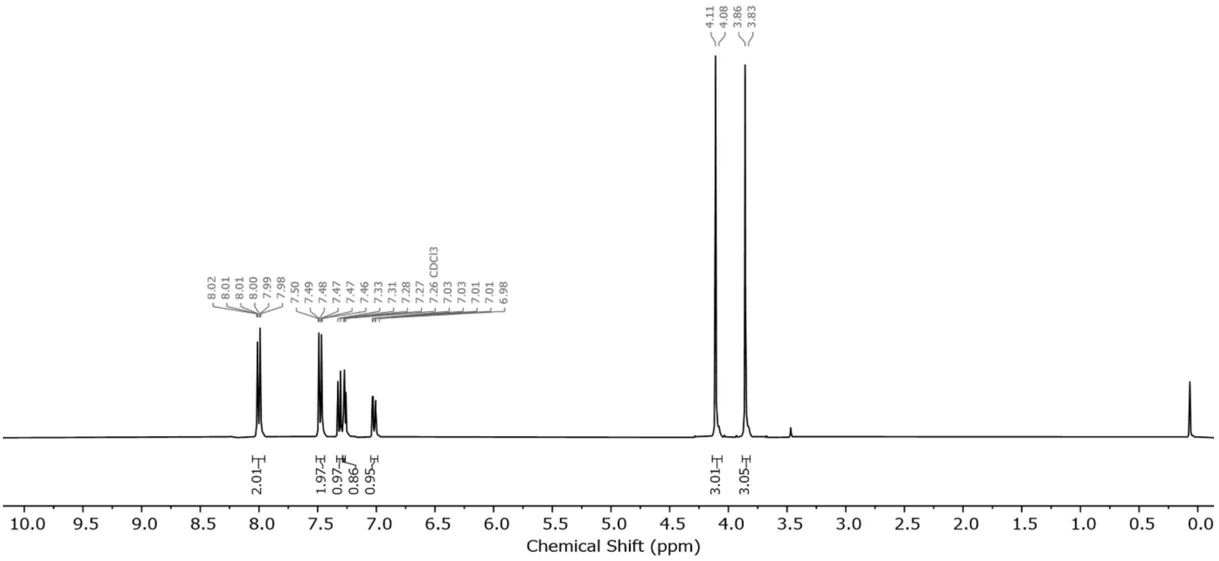

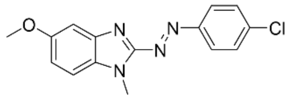

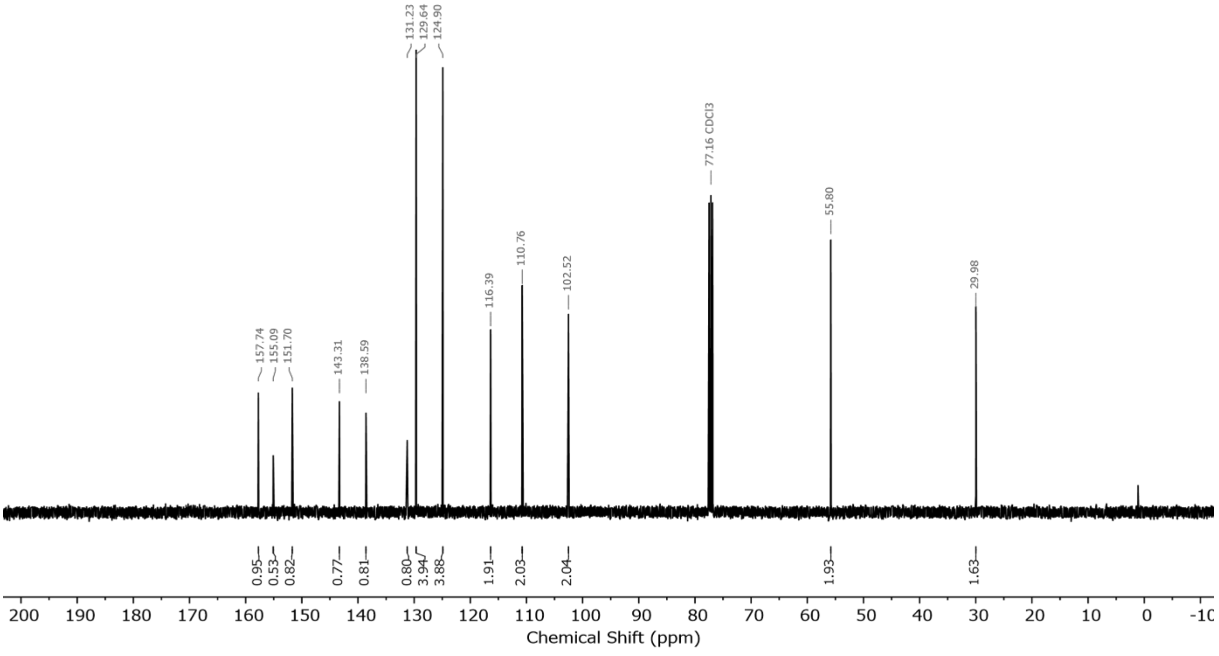

(*E*)-5-methoxy-1-methyl-2-((4-nitrophenyl)diazenyl)-1*H*-benzo[*d*]imidazole (**13e**)

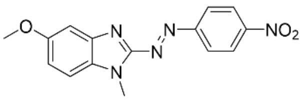

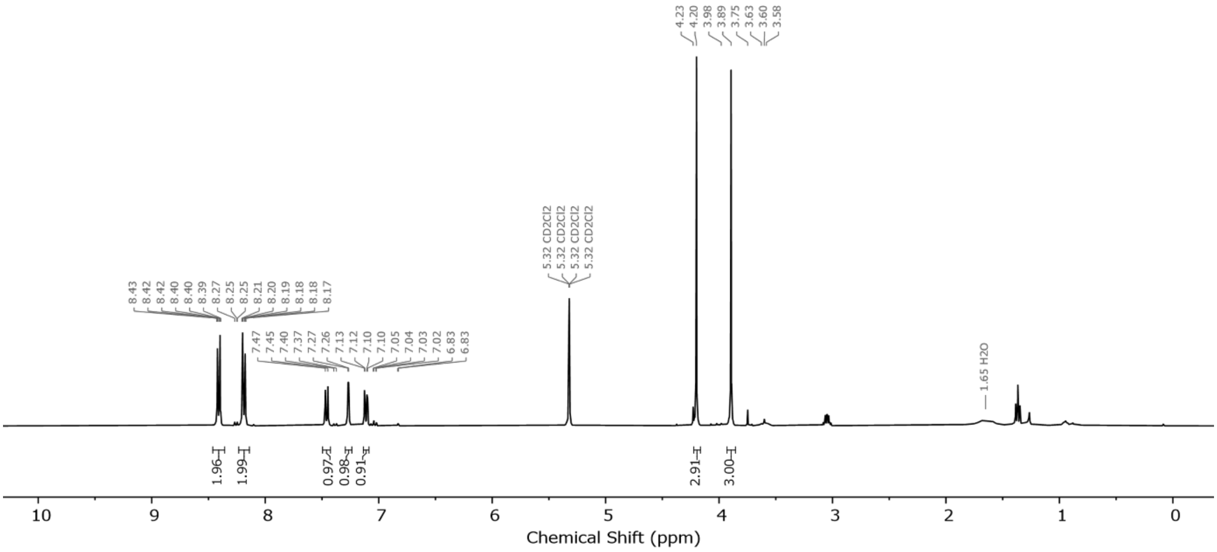

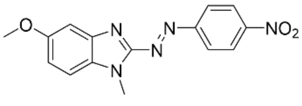

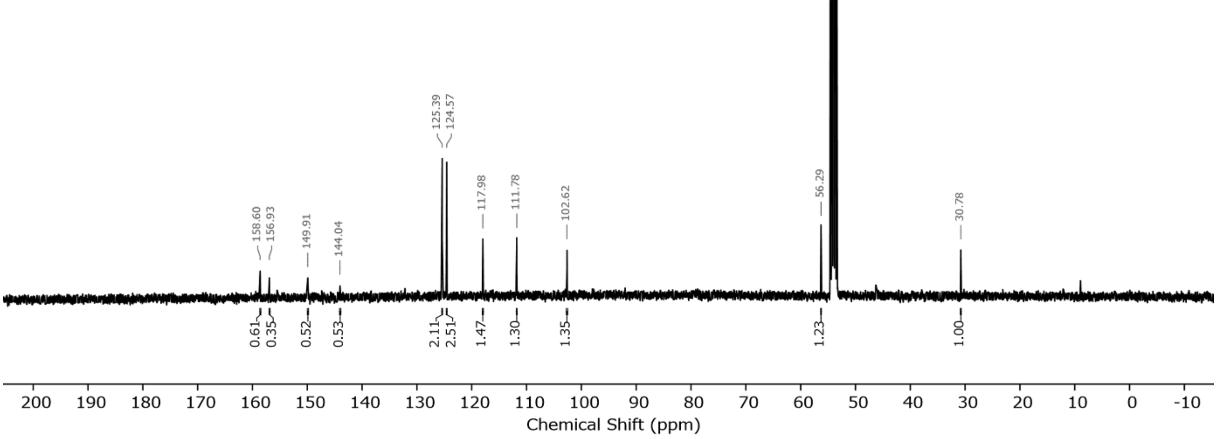

(*E*)-5-methoxy-1-methyl-2-((3-nitrophenyl)diazenyl)-1*H*-benzo[*d*]imidazole (**13f**)

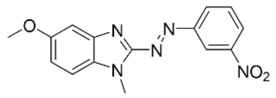

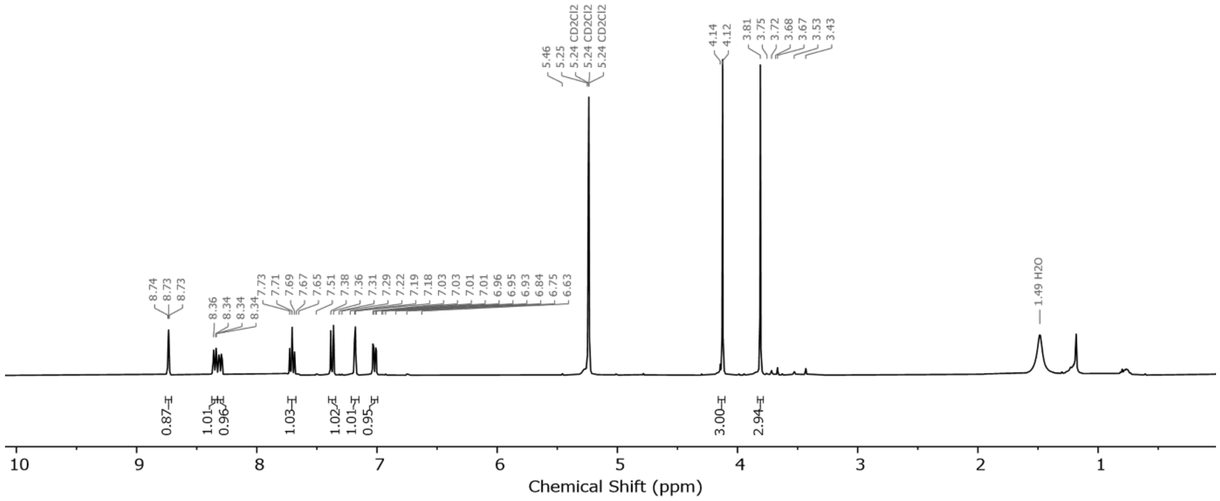

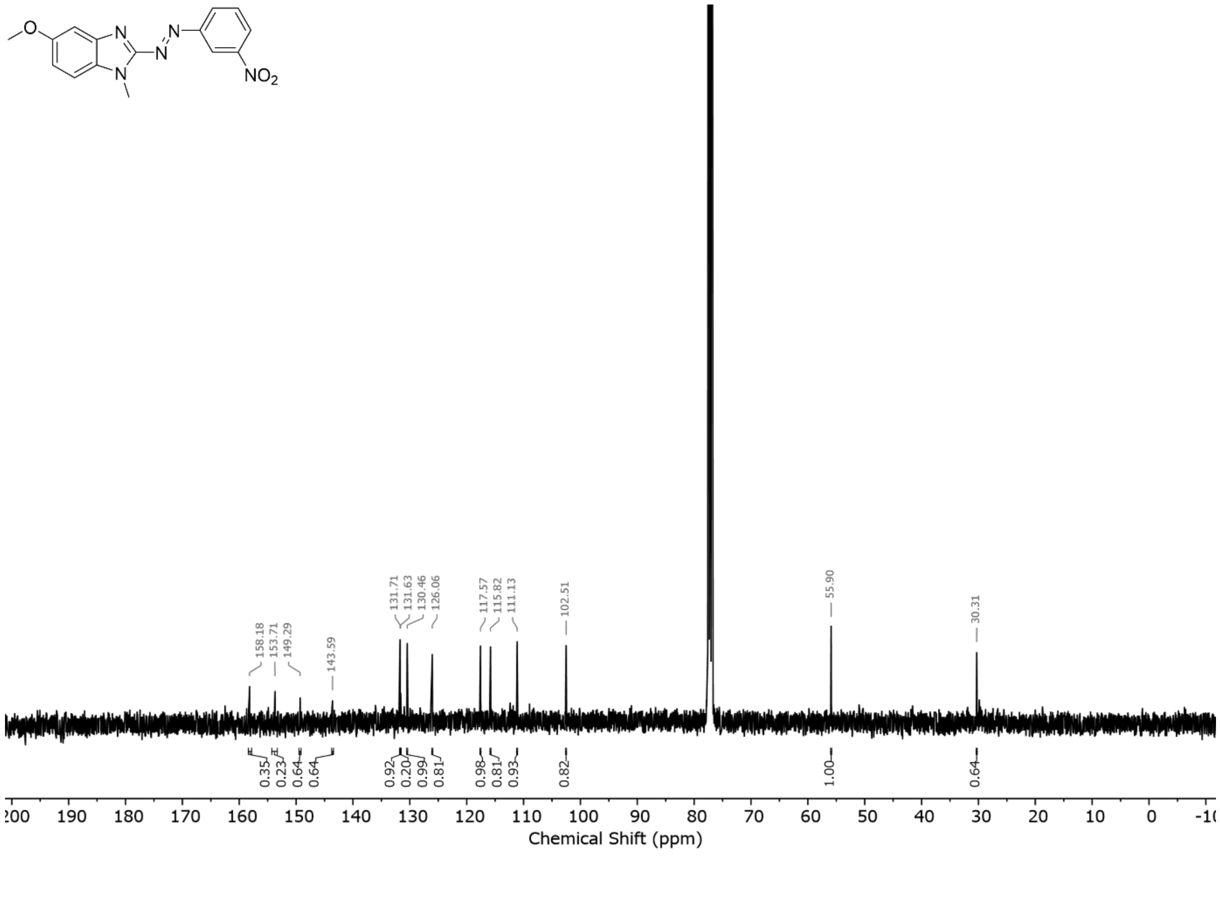

(*E*)-5-methoxy-1-methyl-2-((1-methyl-1*H*-pyrazol-3-yl)diazenyl)-1*H*-benzo[*d*]imidazole (**13pz**)

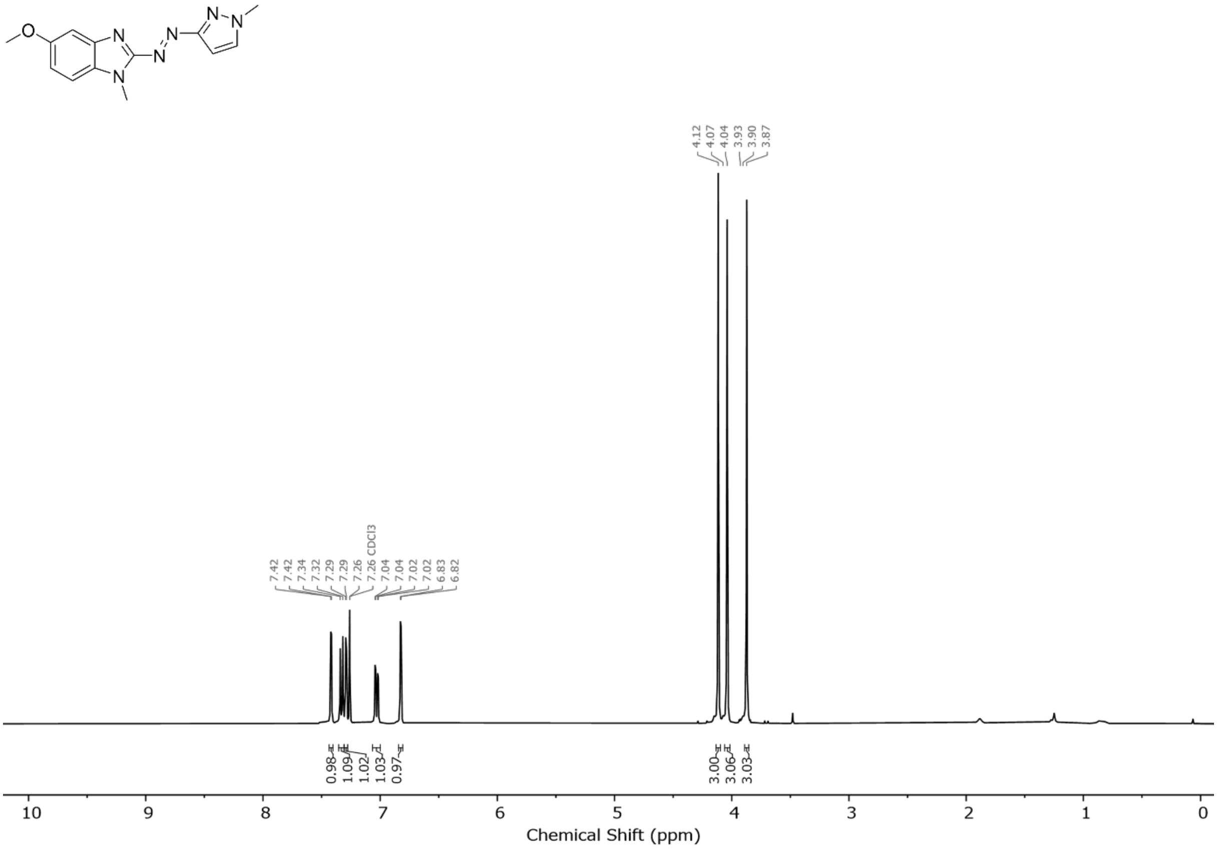

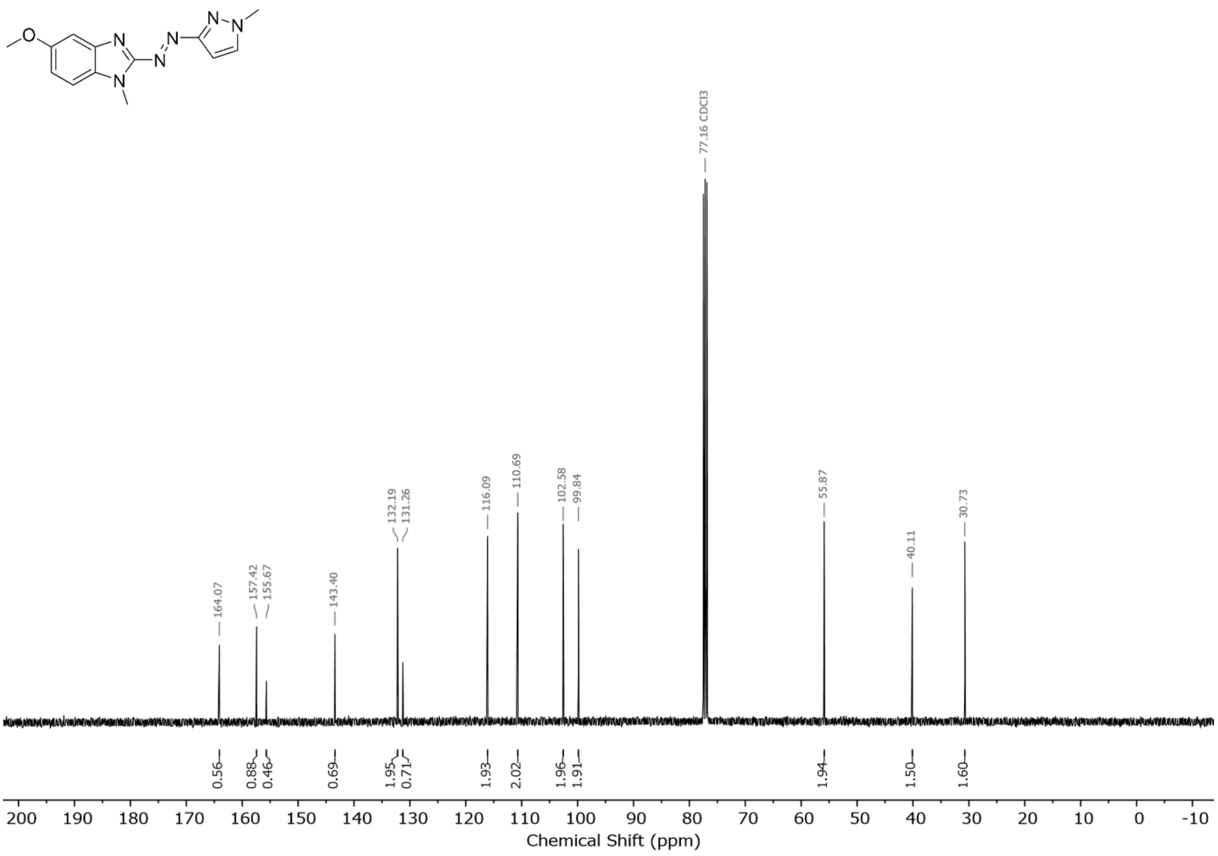

(*E*)-6-methoxy-1-methyl-2-(phenyldiazenyl)-1*H*-benzo[*d*]imidazole (**18a**)

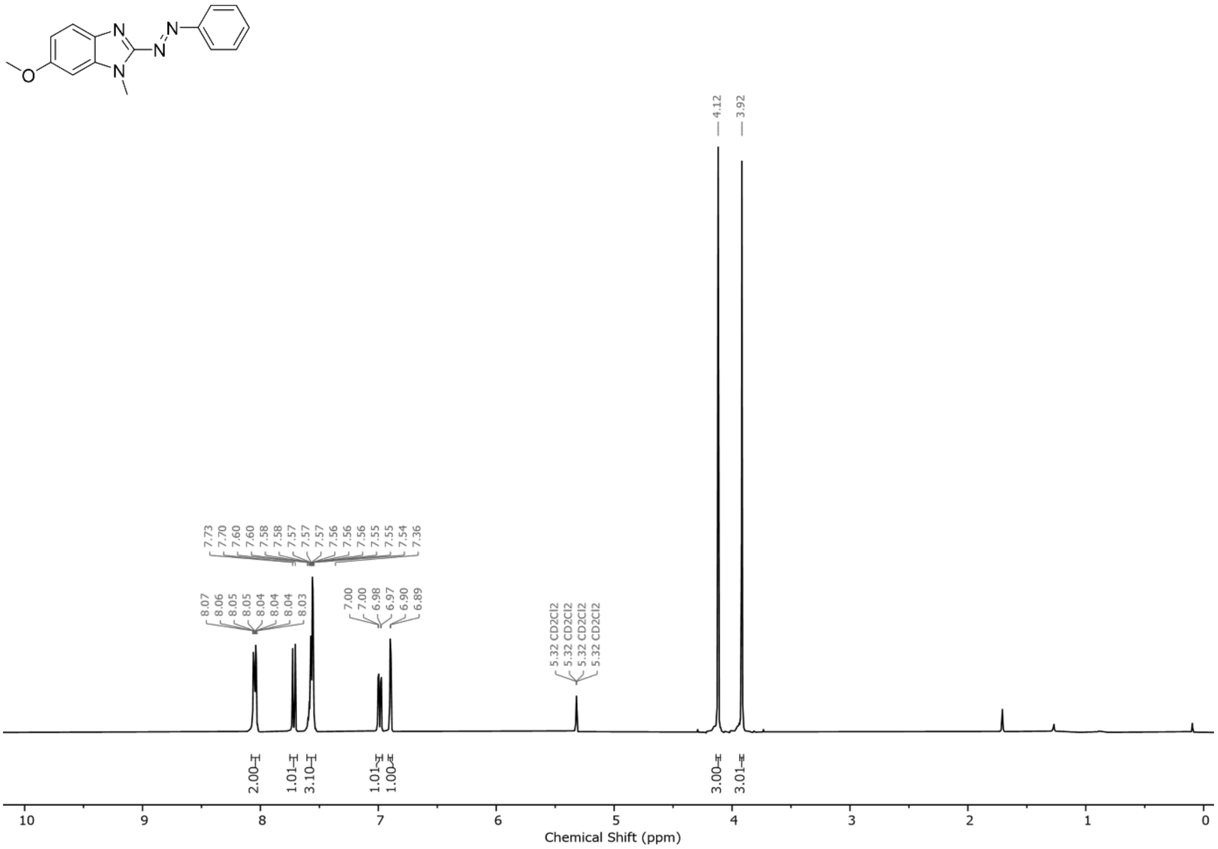

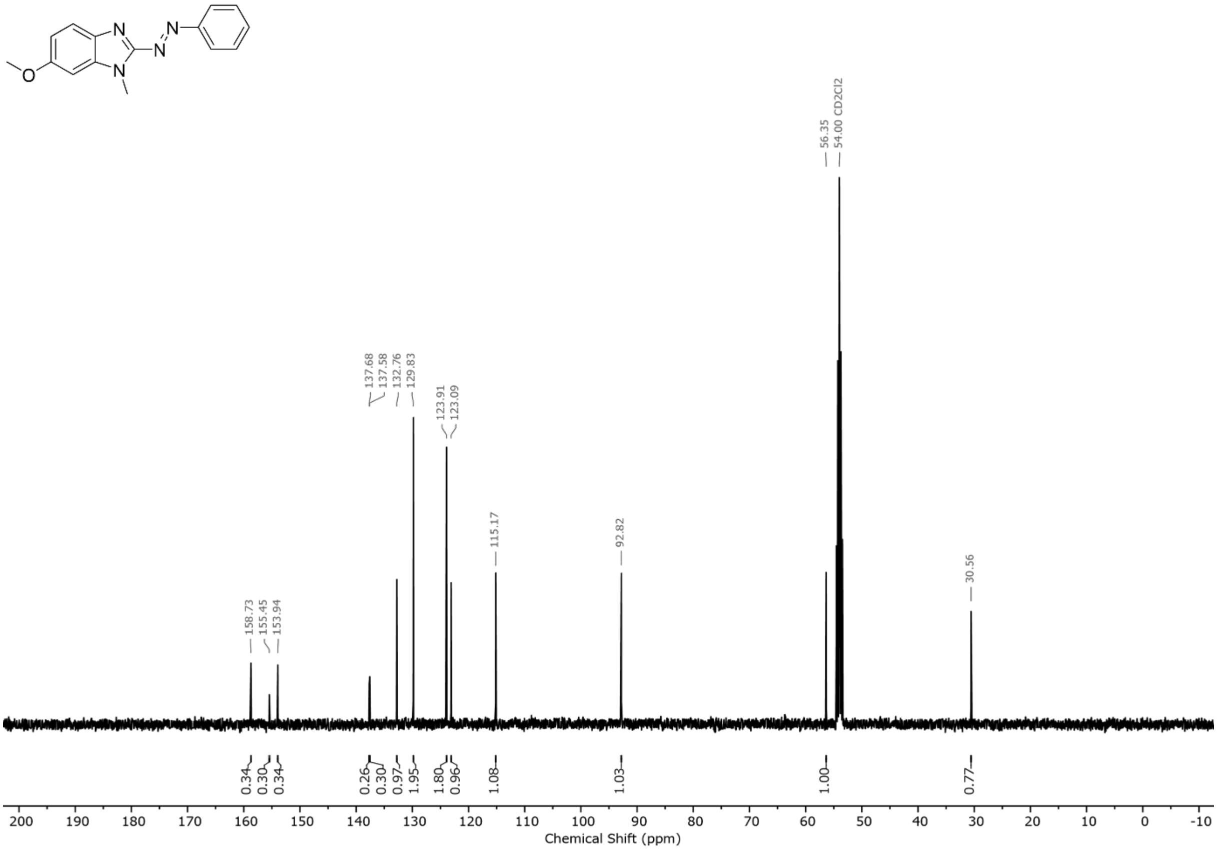

(*E*)-2-((4-ethoxyphenyl)diazenyl)-6-methoxy-1-methyl-1*H*-benzo[*d*]imidazole (**18b**)

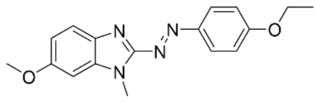

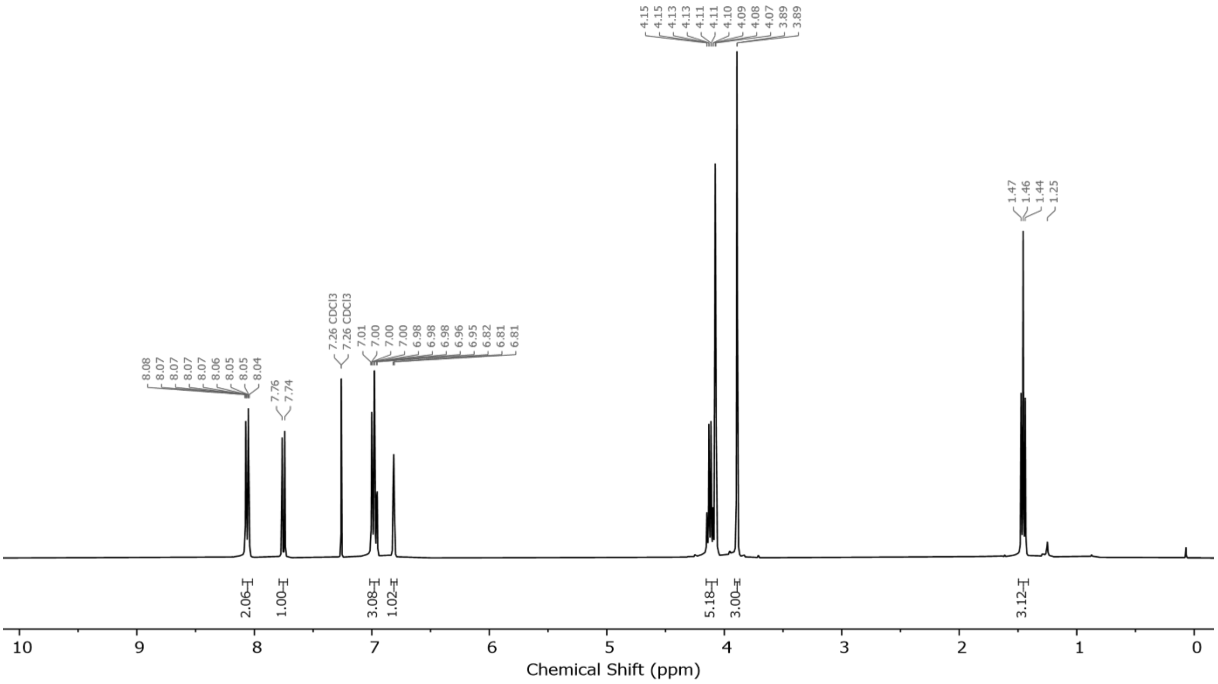

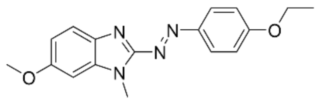

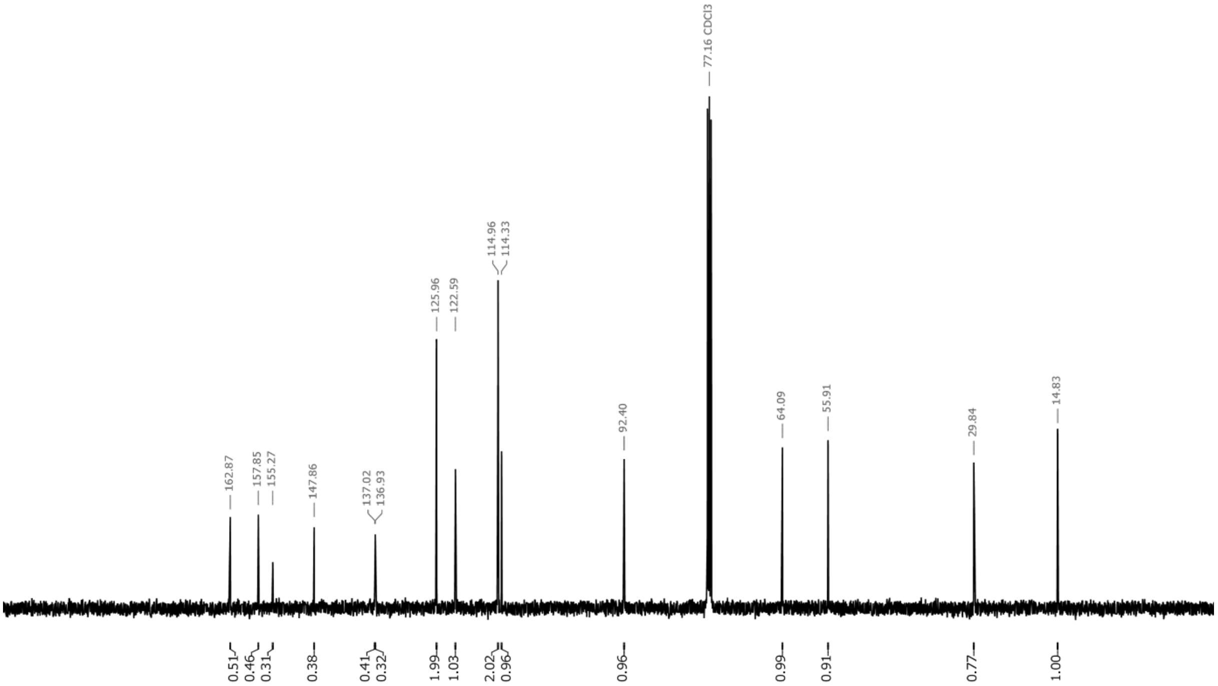

(*E*)-2-((4-chlorophenyl)diazenyl)-6-methoxy-1-methyl-1*H*-benzo[*d*]imidazole (**18d**)

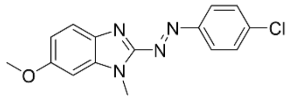

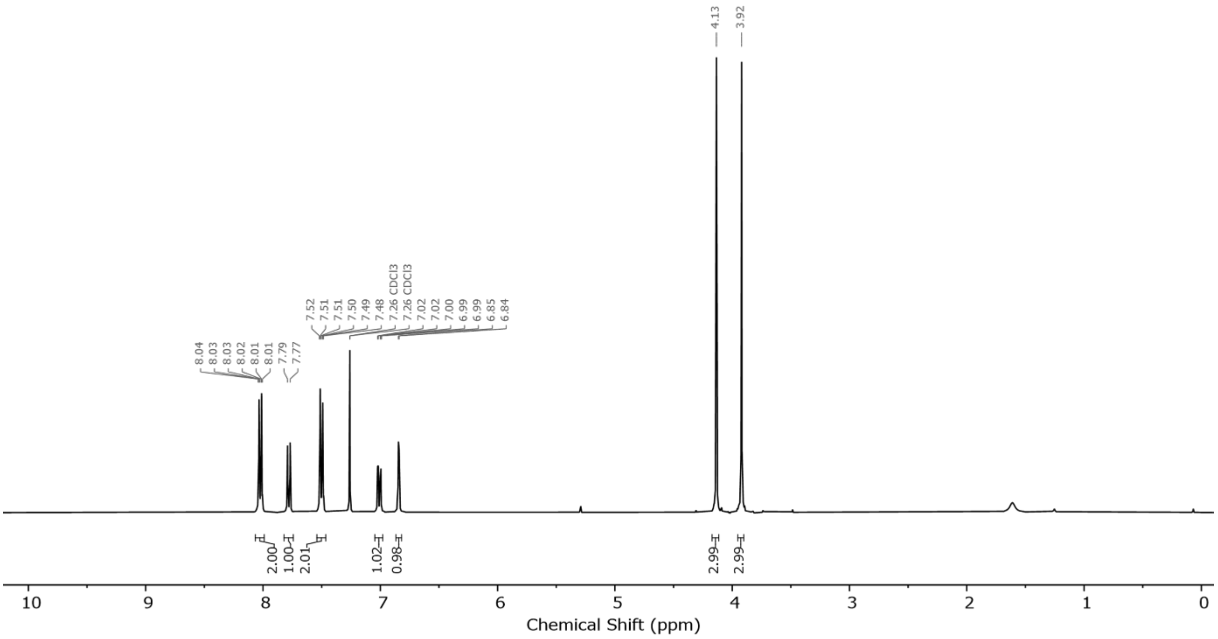

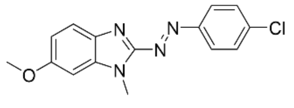

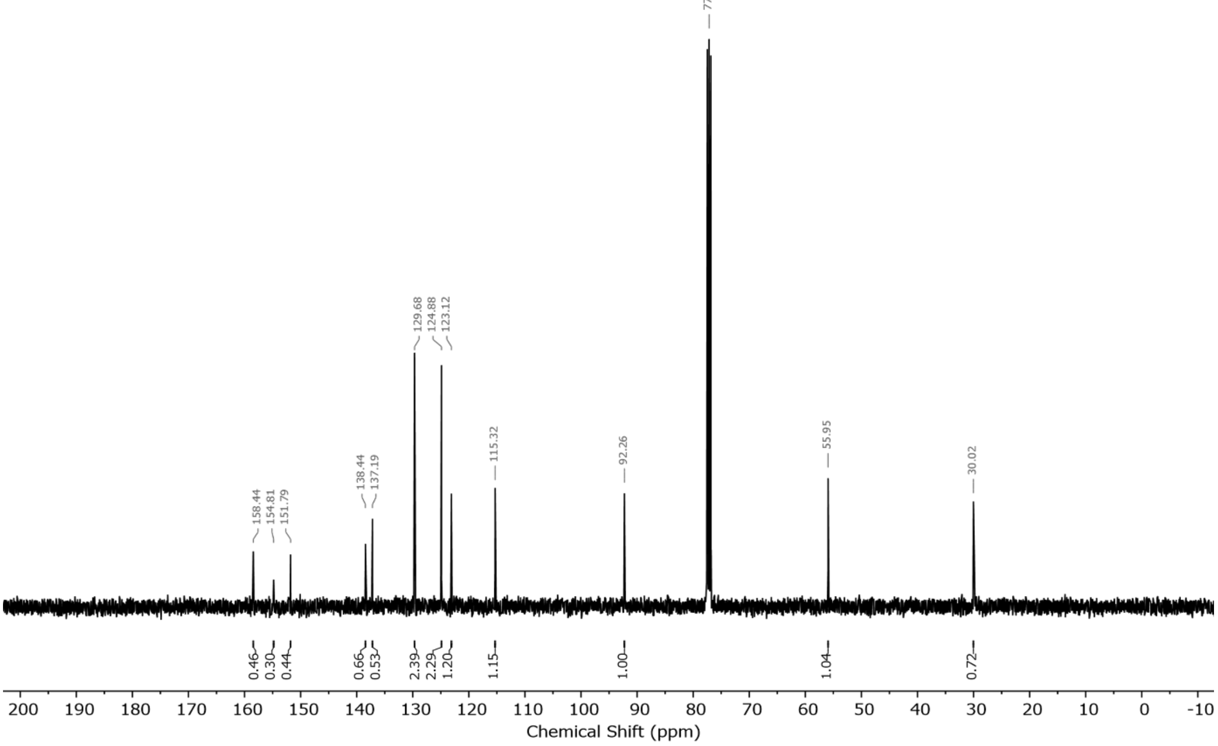

(*E*)-6-methoxy-1-methyl-2-((1-methyl-1*H*-pyrazol-3-yl)diazenyl)-1*H*-benzo[*d*]imidazole (**18pz**)

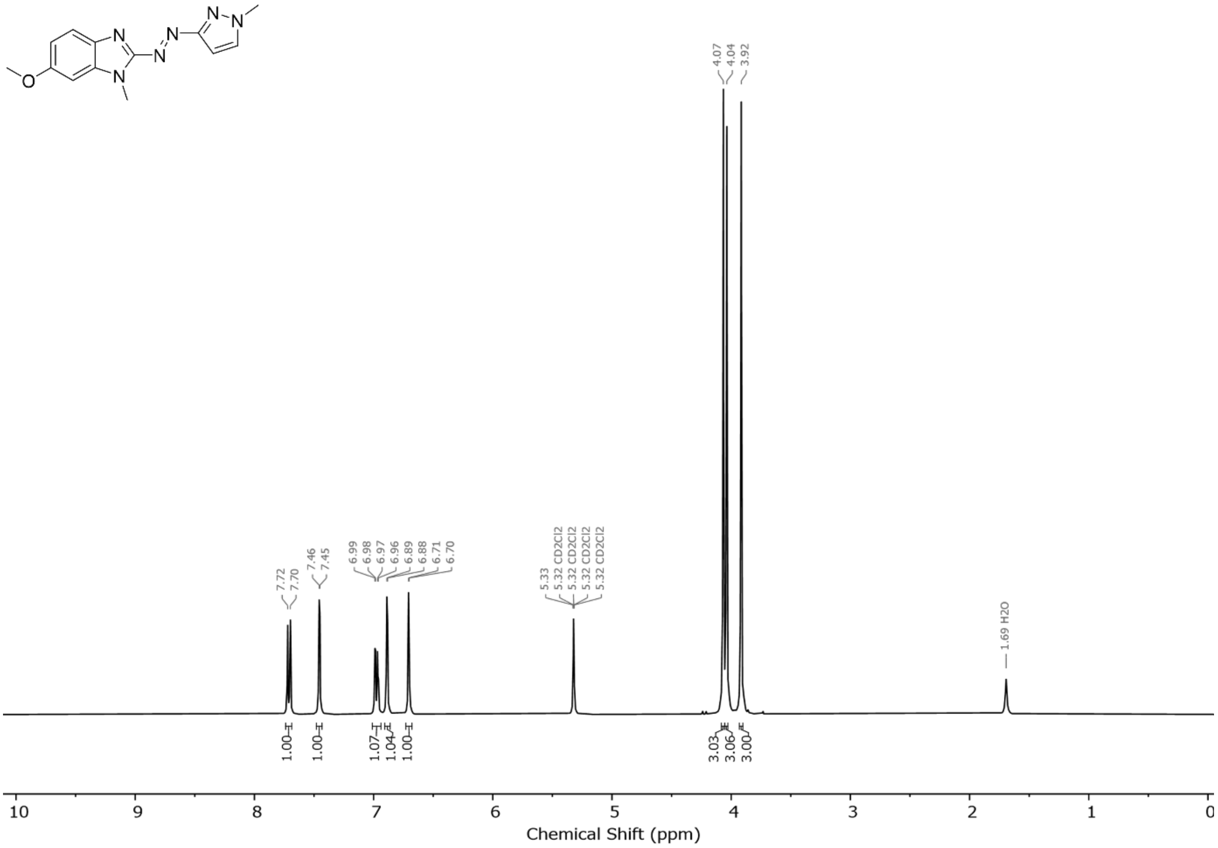

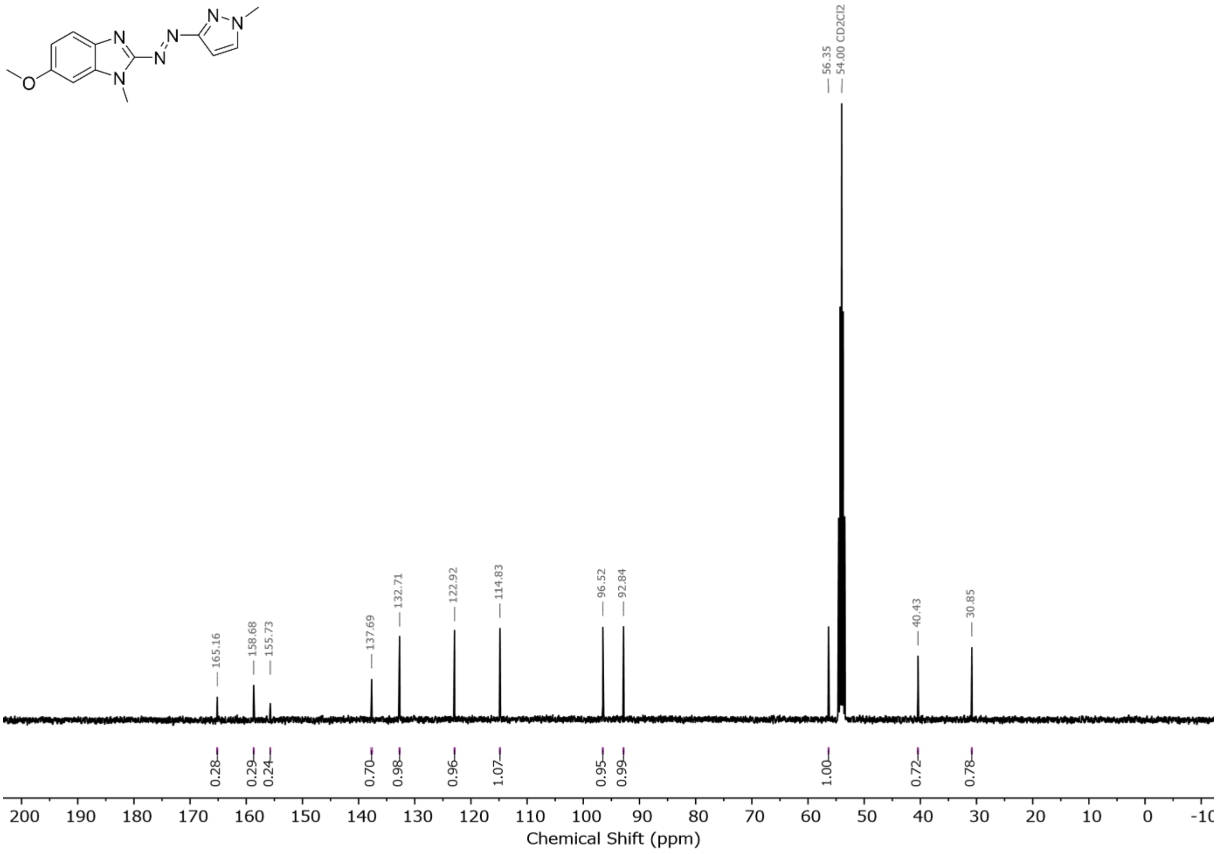

(*E*)-1-methyl-5-nitro-2-(phenyldiazenyl)-1*H*-benzo[*d*]imidazole (**23a**)

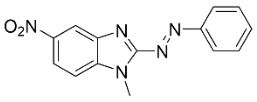

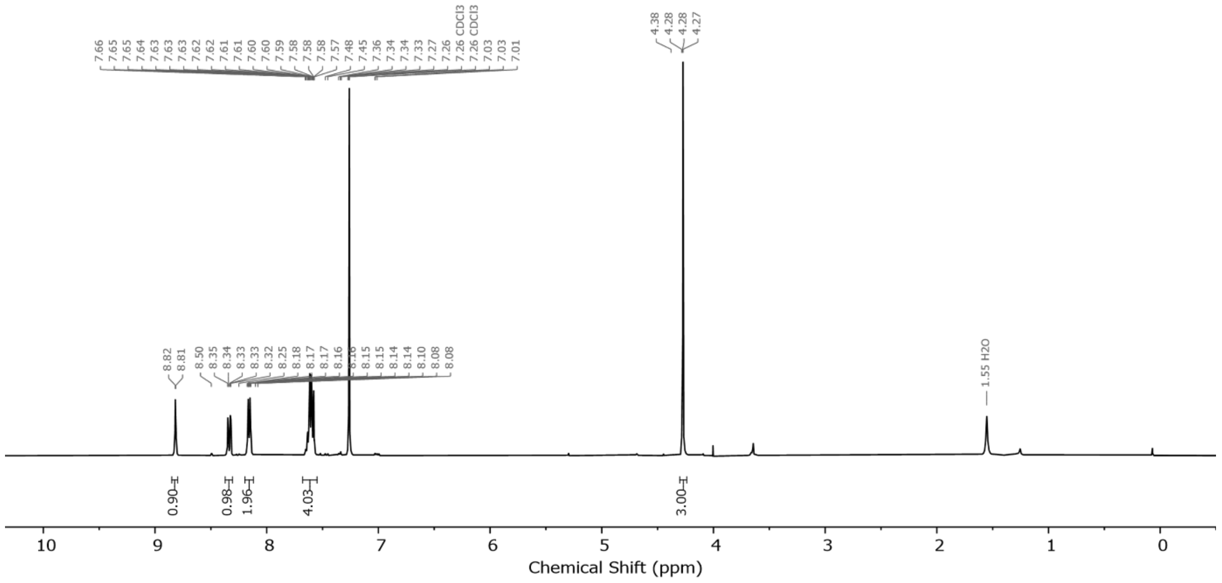

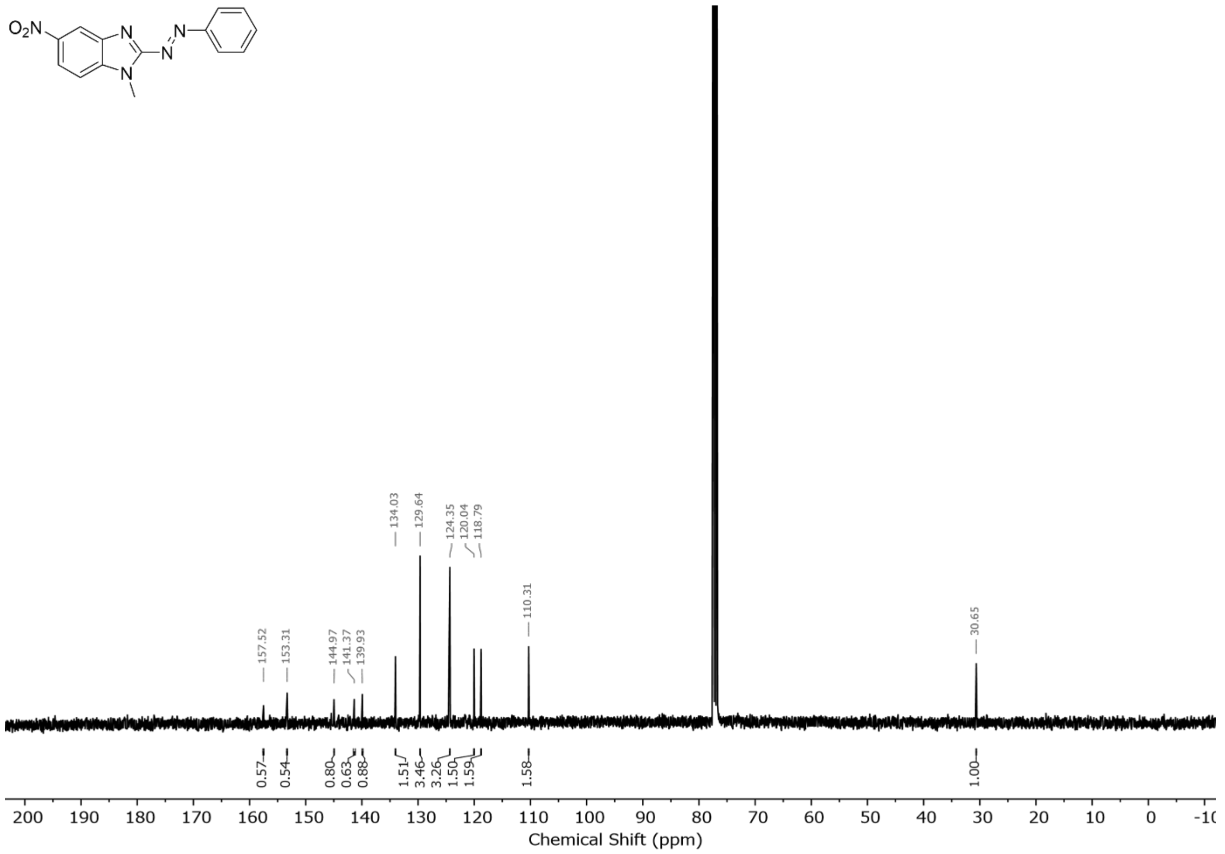

(*E*)-2-((4-ethoxyphenyl)diazenyl)-1-methyl-5-nitro-1*H*-benzo[*d*]imidazole (**23b**)

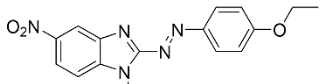

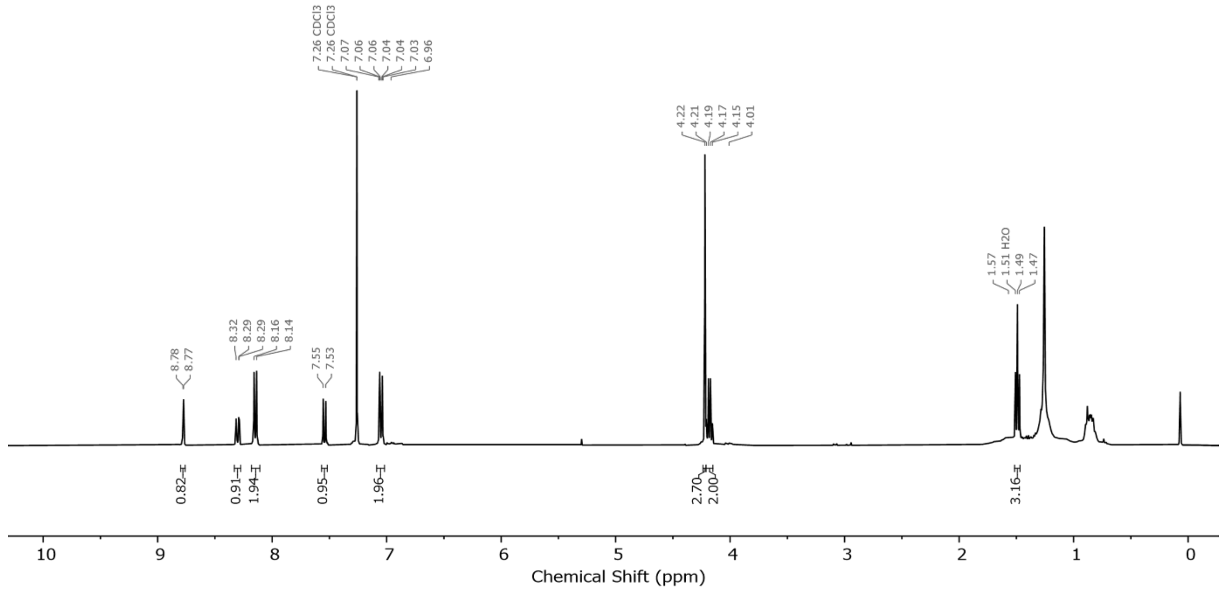

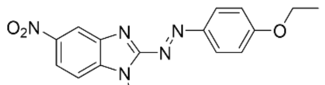

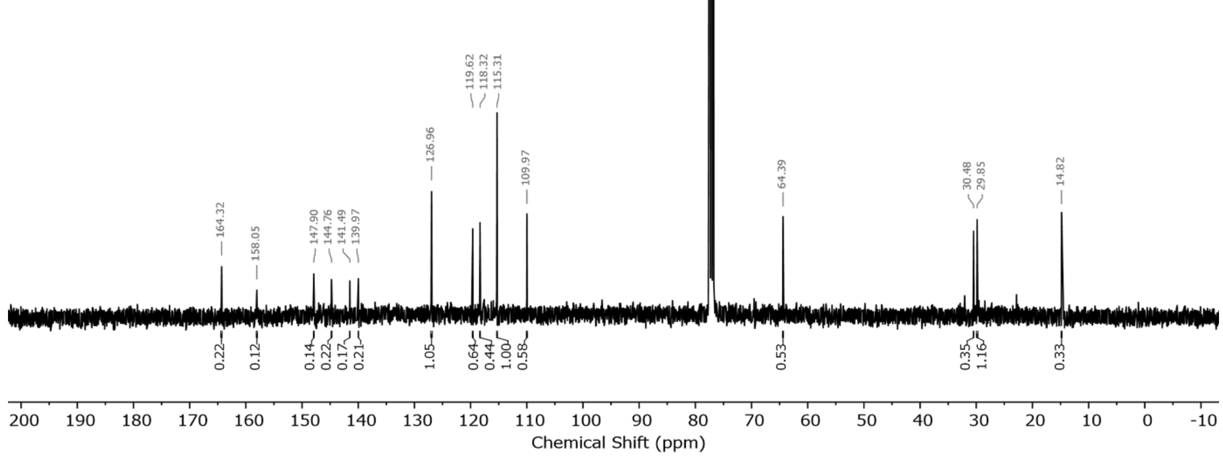

(*E*)-2-((3-ethoxyphenyl)diazenyl)-1-methyl-5-nitro-1*H*-benzo[*d*]imidazole (**23c**)

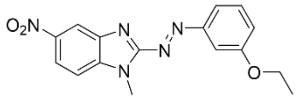

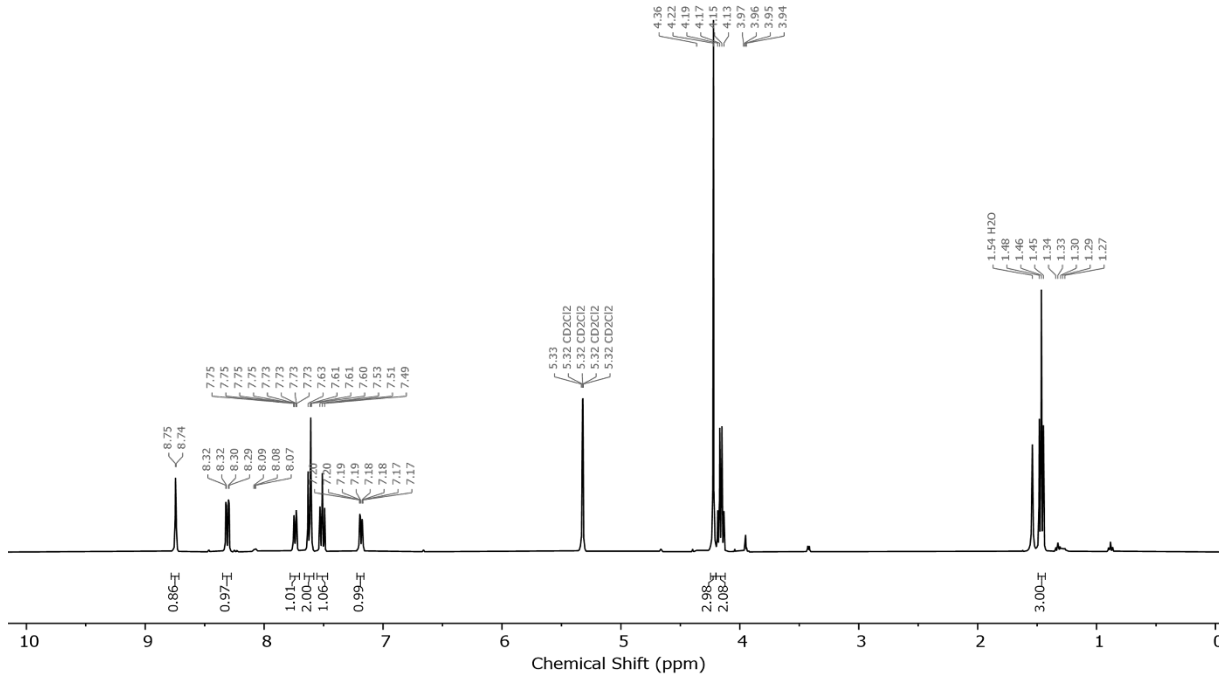

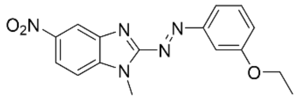

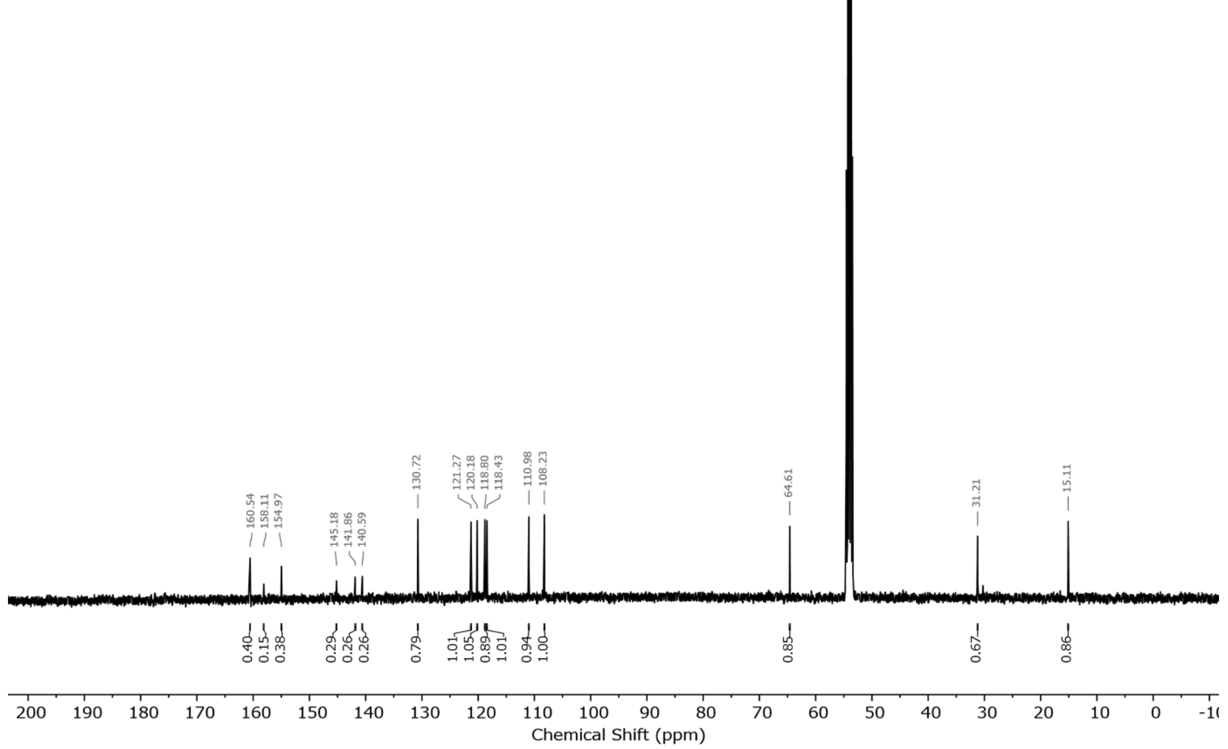

(*E*)-2-((4-chlorophenyl)diazenyl)-1-methyl-5-nitro-1*H*-benzo[*d*]imidazole (**23d**)

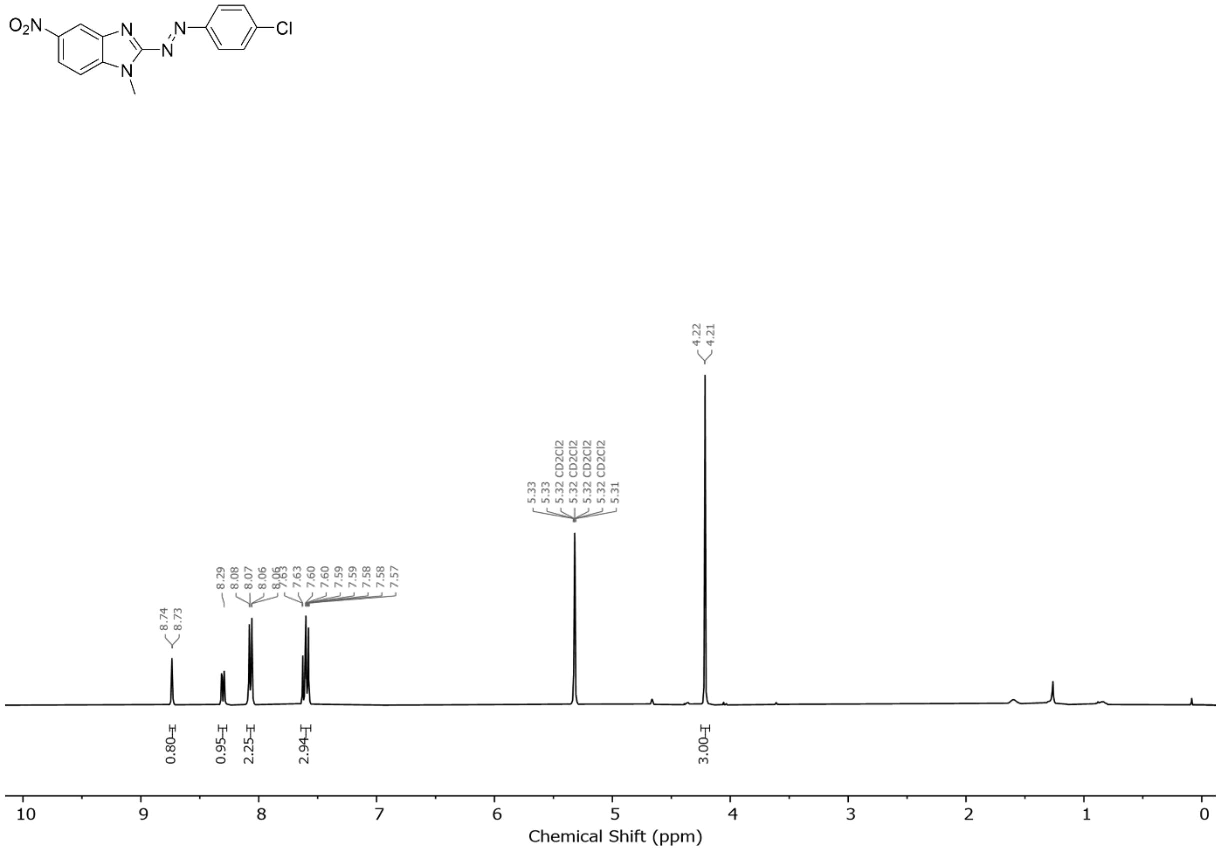

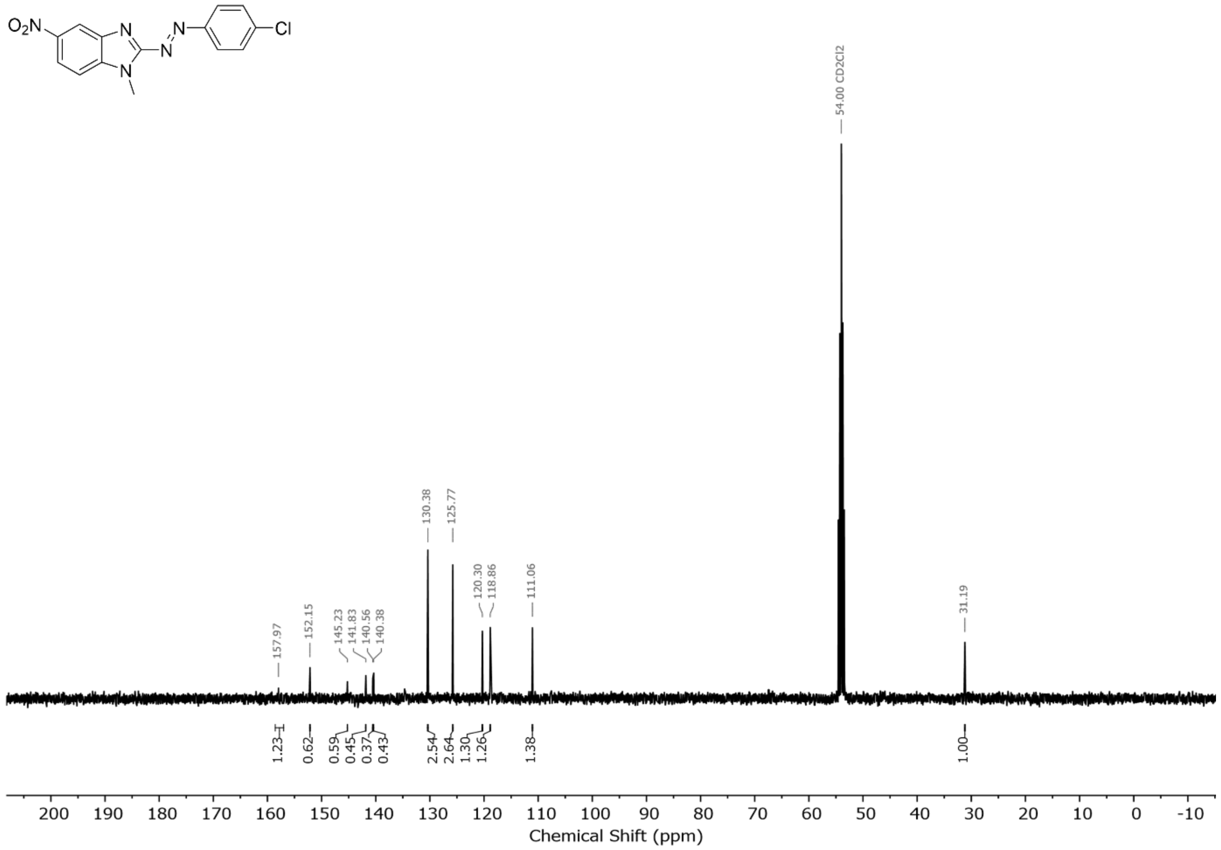

(*E*)-1-methyl-6-nitro-2-(phenyldiazenyl)-1*H*-benzo[*d*]imidazole (**26a**)

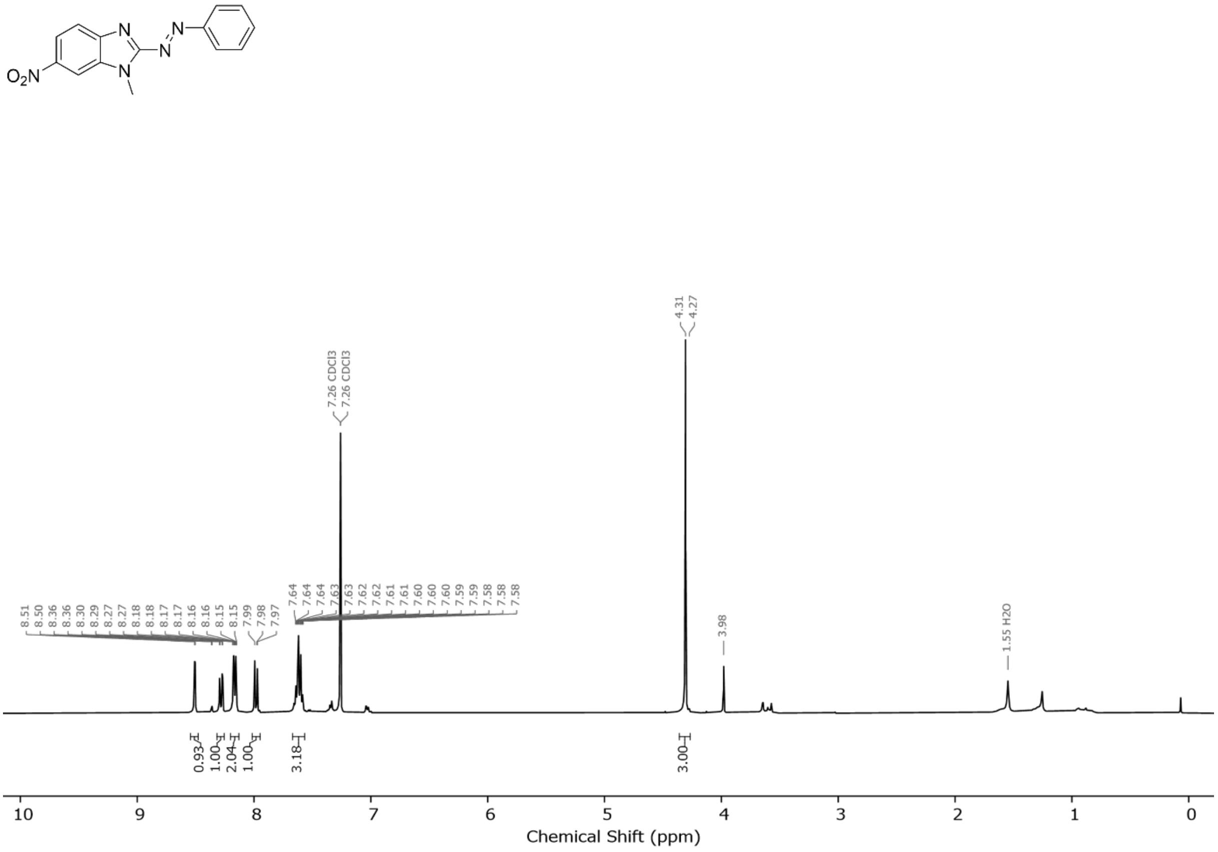

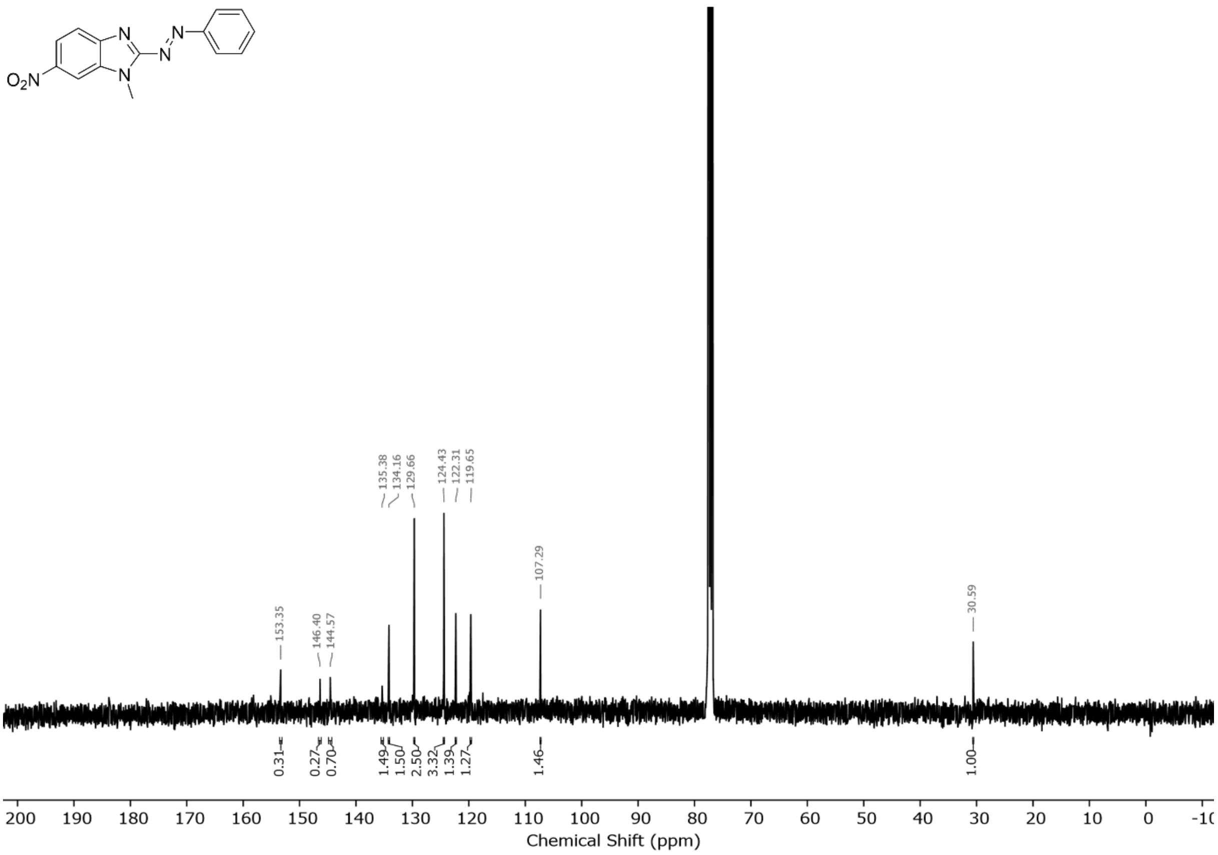

(*E*)-2-((4-ethoxyphenyl)diazenyl)-1-methyl-6-nitro-1*H*-benzo[*d*]imidazole (**26b**)

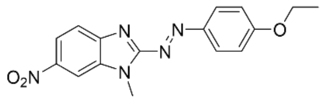

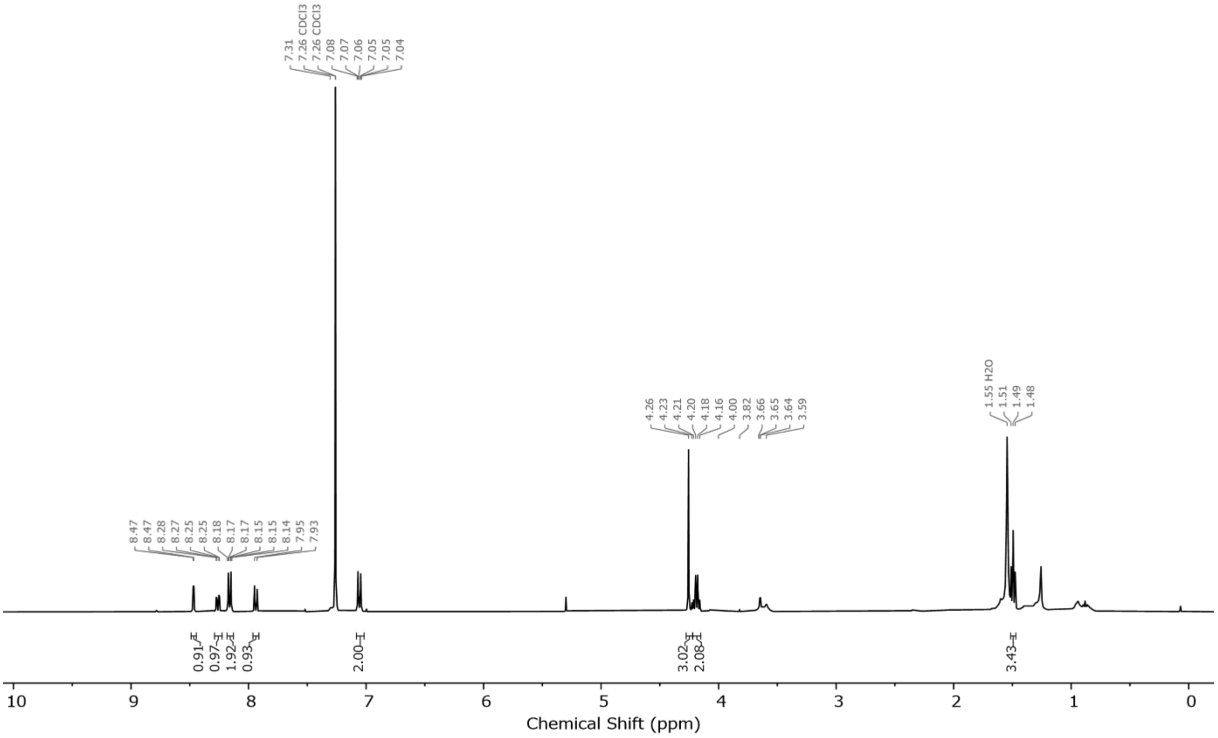

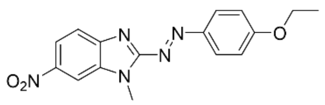

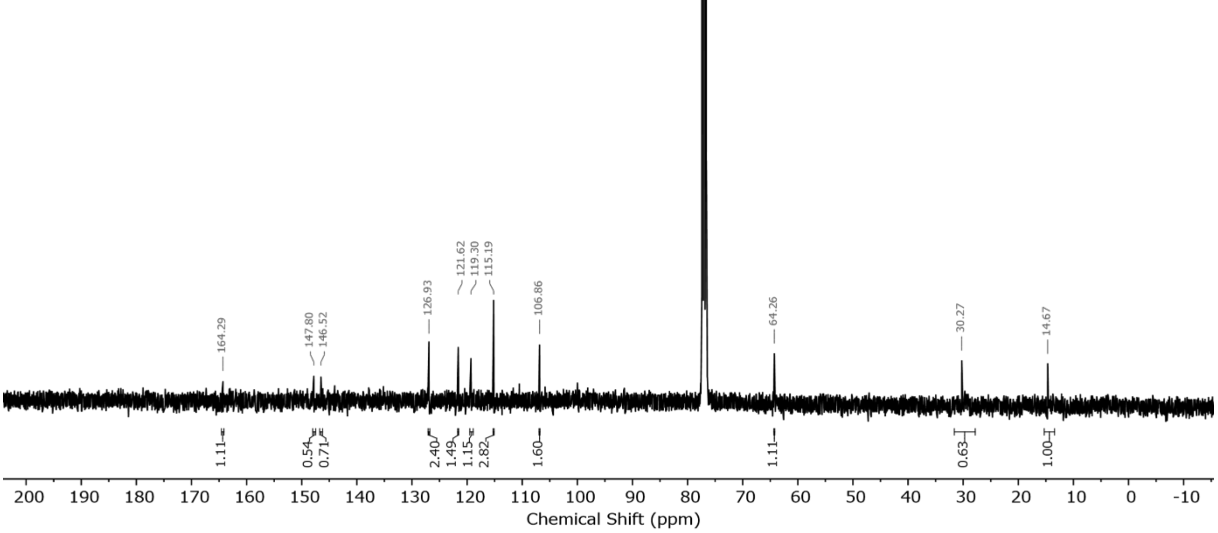

Computational

General Methods

Theoretical calculations were carried out in Gaussian16.[5] All geometry optimisations and frequency calculations were performed in the density functional theory (DFT) framework using the hybrid exchange−correlation PBE0[6] functional together with the GD3BJ version of Grimme's dispersion correction[7] and the split-valence Pople's 6-31G(d,p) basis set.[8] All geometry optimisations were carried out in gas phase until convergence, followed by a frequency calculation in order to ensure a true local minimum on the potential energy surface (PES) was achieved. Time-dependant DFT (TD-DFT) calculations were performed at the PBE0/6-31G(d,p)[6, 8] and the CAM-B3LYP/6-31G+(d,p)[9] levels of theory in the gas phase. The excitation energies and oscillation strengths of the 20 lowest singlet excited states were computed for all available *E* and *Z* conformers. Visualisation and subsequent analysis of the energy-minimised structures was performed using Avogadro 1.2.0 software.

Optimised Geometries

Two conformers were obtained for each of the *E* and *Z* isomers of arylazobenzimidazoles **3a**, **8a**, **8e**, **23a** and **26a**. Four conformers were obtained for each of the *E* and *Z* isomers of arylazobenzimidazoles **13a**, **18a**, and **3pz**. Four available conformers were also identified for *E*-**8pz** whereas *Z*-**8pz** was characterised with three ground state conformations. All *E*-arylazobenzimidazoles conformers were predicted to adopt a planar disposition. The majority of the *Z*-arylazobenzimidazole conformers exhibited twisted geometry with the exception of the two pyrazole analogues **3pz** and **8pz** that demonstrated available planar conformations (1-*Z*-**3pz**, 2-*Z*-**3pz** and 1-*Z*-**8pz**). The free energies are computed relative to the lowest energy conformer of the isomer of the compound. Population of each conformer was obtained according to the Boltzmann distribution. The free energy and population are computed at 25 °C.

Table S2. Calculated data for the optimised geometries of *E*-arylazobenzimidazoles considered at the PBE0-D3/6-31G(d,p) level of theory in the gas phase. The lowest energy conformers are highlighted.

compound	conformation	geometry	Δ Free energy/ kJ mol^{-1}	population/ %
**3a**	1	planar	13.4	0.4
	**2**	**planar**	**0**	**99.6**
**3pz**	1	planar	17.5	0.08
	2	planar	20.2	0.03
	3	planar	9.13	2.45
	**4**	**planar**	**0**	**97.44**
**8a**	**1**	**planar**	**0**	**72.9**
	2	planar	2.45	27.1
**8e**	**1**	**planar**	**0**	**80.1**
	2	planar	3.45	19.9
**8pz**	1	planar	1.51	30.3
	2	planar	3.74	12.4
	3	planar	8.99	1.5
	**4**	**planar**	**0**	**55.8**
**13a**	**1**	**planar**	**0**	**71.2**
	2	planar	6.18	5.9
	3	planar	3.07	20.6
	4	planar	8.57	2.3
**18a**	1	planar	2.18	20.7
	**2**	**planar**	**0**	**49.9**
	3	planar	2.05	21.8
	4	planar	4.66	7.6
**23a**	**1**	**planar**	**0**	**70.6**
	2	planar	2.17	29.4
**26a**	**1**	**planar**	**0**	**81.7**
	2	planar	3.71	18.3

Table S3. Calculated data for the optimised geometries of *Z*-arylazobenzimidazoles considered at the PBE0-D3/6-31G(d,p) level of theory in the gas phase. The lowest energy conformers are highlighted.

compound	conformation	geometry	Δ Free energy/ kJ mol^{-1}	population/ %
**3a**	**1**	**twisted**	**0**	**85.6**
	2	twisted	4.41	14.4
**3pz**	1	planar	1.38	35.3
	**2**	**planar**	**0**	**61.6**
	3	twisted	7.72	2.7
	4	twisted	12.3	0.4
**8a**	**1**	**twisted**	**0**	**98.6**
	2	twisted	10.5	1.4
**8e**	**1**	**twisted**	**0**	**99.97**
	2	twisted	20.5	0.03
**8pz**	**1**	**planar**	**0**	**91.4**
	2	twisted	6.30	7.2
	3	twisted	10.3	1.4
**13a**	**1**	**twisted**	**0**	**90.2**
	2	twisted	5.74	8.9
	3	twisted	11.9	0.8
	4	twisted	16.3	0.1
**18a**	1	twisted	2.65	25.4
	**2**	**twisted**	**0**	**74.1**
	3	twisted	14.9	0.2
	4	twisted	13.5	0.3
**23a**	**1**	**twisted**	**0**	**97.2**
	2	twisted	8.83	2.8
**26a**	**1**	**twisted**	**0**	**96.1**
	2	twisted	7.92	3.9

Cartesian coordinates for all optimised geometries

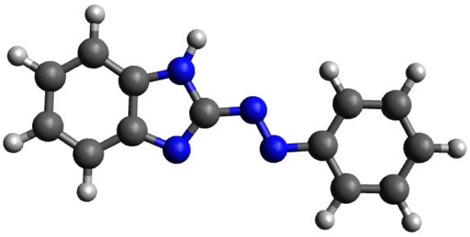

1-*E*-**3a**

C	-2.65803	0.66738	0.00002
C	-2.83485	-0.74176	-0.00002
N	-1.32621	0.99462	-0.00003
C	-0.70517	-0.16271	-0.00008
N	-1.56076	-1.23904	-0.00010
N	0.64628	-0.47144	-0.00016
N	1.40725	0.52926	-0.00015
C	-4.09384	-1.34080	0.00002
C	-5.18570	-0.48718	0.00009
C	-5.03193	0.91363	0.00012
C	-3.78105	1.50466	0.00008
H	-4.21773	-2.41909	-0.00001
H	-6.18639	-0.90834	0.00012
H	-5.91966	1.53853	0.00017
H	-3.65452	2.58197	0.00011
H	5.68021	1.98301	-0.00002
C	5.01354	1.12631	0.00001
C	3.63709	1.31622	-0.00007
H	3.19402	2.30693	-0.00015
C	2.77787	0.21402	-0.00005
C	3.30199	-1.08652	0.00006
H	2.61621	-1.92645	0.00007
C	4.67500	-1.26815	0.00014
H	5.08737	-2.27289	0.00022
C	5.53312	-0.16505	0.00011
H	6.60840	-0.31739	0.00017
H	-1.26195	-2.20044	-0.00010

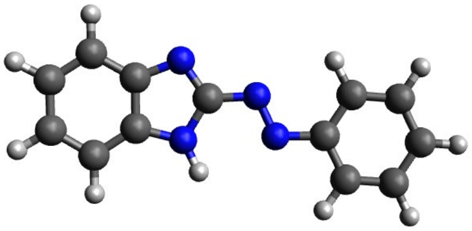

2-*E*-**3a**

C	-2.81277	-0.76666	-0.00001
C	-2.61382	0.63982	0.00005
N	-1.60998	-1.42676	0.00012
C	-0.71174	-0.46792	0.00003
N	-1.25402	0.79353	0.00016
N	0.64908	-0.71415	-0.00003
N	1.33013	0.34657	-0.00016
C	-3.67447	1.54374	0.00007
C	-4.95109	1.00174	-0.00001
C	-5.16862	-0.38997	-0.00009
C	-4.11337	-1.28512	-0.00009
H	-3.51489	2.61716	0.00015
H	-5.80751	1.66920	0.00000
H	-6.18826	-0.76232	-0.00013
H	-4.27018	-2.35850	-0.00012
H	5.48084	2.13651	-0.00002
C	4.88126	1.23159	-0.00002
C	3.49454	1.31631	-0.00009
H	2.98264	2.27352	-0.00016
C	2.72215	0.15199	-0.00007
C	3.34176	-1.10559	-0.00000
H	2.71979	-1.99396	-0.00000
C	4.72482	-1.18126	0.00006
H	5.21121	-2.15213	0.00011
C	5.49716	-0.01680	0.00006
H	6.58082	-0.08699	0.00011
H	-0.69566	1.63207	0.00007

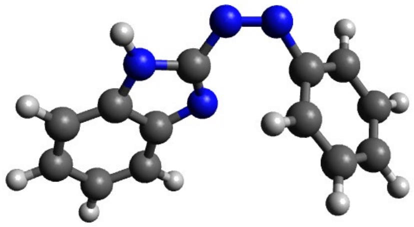

1-*Z*-**3a**

C	-1.69646	-0.42740	-0.36448
C	-2.48328	0.61848	0.18344
N	-0.39440	-0.02243	-0.52694
C	-0.37816	1.21850	-0.09920
N	-1.60773	1.66146	0.32456
N	0.61642	2.20336	-0.16791
N	1.83518	1.94760	-0.24915
C	-3.84061	0.46728	0.46308
C	-4.39880	-0.76772	0.17191
C	-3.63428	-1.81757	-0.37402
C	-2.28661	-1.66471	-0.64863
H	-4.43459	1.27290	0.88259
H	-5.45394	-0.93047	0.36903
H	-4.11878	-2.76665	-0.58130
H	-1.68975	-2.46807	-1.06684
H	4.70455	-1.35595	-1.47346
C	3.98181	-0.99842	-0.74634
C	3.33605	0.21353	-0.95686
H	3.54698	0.82199	-1.83031
C	2.37813	0.65365	-0.04347
C	2.12717	-0.07695	1.12167
H	1.41212	0.29556	1.84823
C	2.80790	-1.26426	1.34346
H	2.62069	-1.82745	2.25278
C	3.72328	-1.73804	0.40438
H	4.24580	-2.67346	0.57935
H	-1.79612	2.60347	0.62637

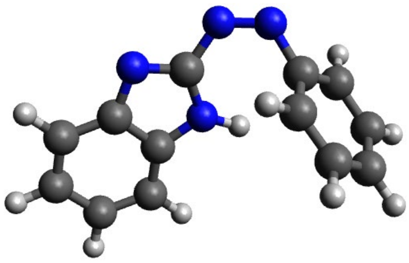

2-*Z*-**3a**

C	-2.81277	-0.76666	-0.00001
C	-2.61382	0.63982	0.00005
N	-1.60998	-1.42676	0.00012
C	-0.71174	-0.46792	0.00003
N	-1.25402	0.79353	0.00016
N	0.64908	-0.71415	-0.00003
N	1.33013	0.34657	-0.00016
C	-3.67447	1.54374	0.00007
C	-4.95109	1.00174	-0.00001
C	-5.16862	-0.38997	-0.00009
C	-4.11337	-1.28512	-0.00009
H	-3.51489	2.61716	0.00015
H	-5.80751	1.66920	0.00000
H	-6.18826	-0.76232	-0.00013
H	-4.27018	-2.35850	-0.00012
H	5.48084	2.13651	-0.00002
C	4.88126	1.23159	-0.00002
C	3.49454	1.31631	-0.00009
H	2.98264	2.27352	-0.00016
C	2.72215	0.15199	-0.00007
C	3.34176	-1.10559	-0.00000
H	2.71979	-1.99396	-0.00000
C	4.72482	-1.18126	0.00006
H	5.21121	-2.15213	0.00011
C	5.49716	-0.01680	0.00006
H	6.58082	-0.08699	0.00011
H	-0.69566	1.63207	0.00007

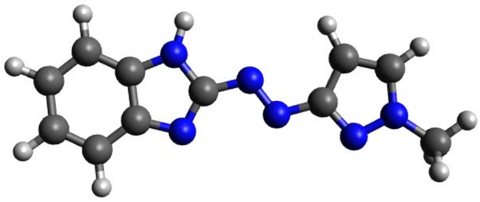

1-*E*-**3pz**

C	2.75874	-0.69960	-0.00200
C	2.99866	0.69992	0.00386
N	1.41315	-0.96697	-0.00453
C	0.84475	0.21653	-0.00042
N	1.74787	1.25378	0.00481
N	-0.49098	0.58915	-0.00043
N	-1.29909	-0.37728	-0.00553
C	4.28303	1.24181	0.00742
C	5.33613	0.34022	0.00495
C	5.11960	-1.05181	-0.00085
C	3.84296	-1.58600	-0.00436
H	4.45509	2.31353	0.01188
H	6.35461	0.71643	0.00755
H	5.97816	-1.71630	-0.00258
H	3.66794	-2.65652	-0.00882
H	-6.12458	-1.40036	1.03375
C	-5.94656	-1.00871	0.02837
H	-6.76616	-0.34680	-0.25426
C	-4.55047	1.08364	-0.00910
N	-4.70799	-0.27022	-0.01743
H	-5.40301	1.74759	-0.01219
C	-3.20116	1.32653	-0.00233
N	-3.55667	-0.92626	-0.01024
C	-2.62288	0.03211	-0.00407
H	-5.88932	-1.84090	-0.67427
H	-2.68546	2.27287	-0.00455
H	1.49185	2.22719	0.00813

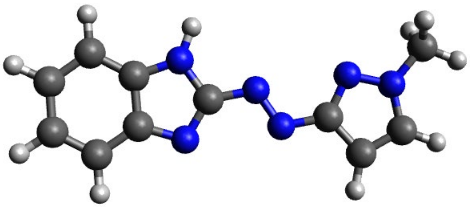

2-*E*-**3pz**

C	-2.79744	-0.63165	-0.00156
C	-2.88331	0.78614	0.00149
N	-1.48921	-1.04414	-0.00191
C	-0.79525	0.07155	0.00081
N	-1.58024	1.20006	0.00294
N	0.57203	0.30251	0.00190
N	1.26817	-0.74591	0.00003
C	-4.10124	1.46466	0.00245
C	-5.24634	0.68350	0.00027
C	-5.18298	-0.72399	-0.00276
C	-3.97228	-1.39420	-0.00370
H	-4.15509	2.54875	0.00478
H	-6.21772	1.16855	0.00091
H	-6.10894	-1.29083	-0.00437
H	-3.91558	-2.47749	-0.00603
H	5.33898	2.09868	0.84319
C	5.51156	1.43773	-0.00852
H	6.51268	1.01056	0.05750
C	4.79460	-0.96951	0.00362
N	4.54384	0.36745	0.00387
H	5.80414	-1.35321	0.00541
C	3.57092	-1.59398	0.00188
N	3.25071	0.66263	0.00104
C	2.63984	-0.53121	0.00054
H	5.42040	2.01398	-0.93222
H	3.35120	-2.64996	0.00354
H	-1.21554	2.13849	0.00509

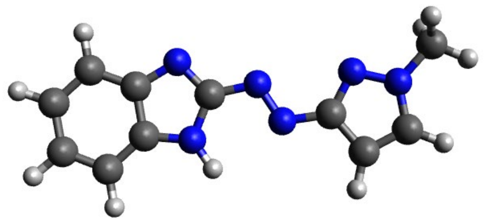

3-*E*-**3pz**

C	-2.87696	-0.81367	-0.00115
C	-2.75423	0.60140	0.00121
N	-1.64021	-1.40823	-0.00255
C	-0.79417	-0.40327	-0.00112
N	-1.40478	0.82761	0.00117
N	0.58004	-0.57577	-0.00188
N	1.18900	0.52982	-0.00045
C	-3.86215	1.44663	0.00300
C	-5.10823	0.83709	0.00236
C	-5.25019	-0.56399	0.00003
C	-4.14770	-1.40092	-0.00174
H	-3.76045	2.52716	0.00480
H	-5.99933	1.45759	0.00370
H	-6.24821	-0.99108	-0.00038
H	-4.24642	-2.48123	-0.00354
H	5.49557	-1.92680	-0.85422
C	5.60403	-1.26453	0.00690
H	6.56573	-0.75304	-0.04268
C	4.68273	1.07332	-0.00290
N	4.54880	-0.27954	-0.00270
H	5.65461	1.54425	-0.00428
C	3.40895	1.58948	-0.00186
N	3.28642	-0.68537	-0.00061
C	2.57457	0.44894	-0.00064
H	5.55094	-1.85790	0.92238
H	3.10439	2.62432	-0.00351
H	-0.89175	1.69428	0.00242

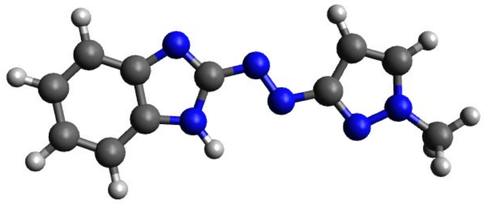

4-*E*-**3pz**

C	2.97608	0.72031	0.00367
C	2.69454	-0.67186	-0.00194
N	1.81369	1.45040	0.00535
C	0.86127	0.54580	0.00095
N	1.32798	-0.74527	-0.00355
N	-0.48505	0.86857	0.00083
N	-1.21734	-0.16026	-0.00401
C	3.69988	-1.63675	-0.00466
C	5.00670	-1.17171	-0.00161
C	5.30583	0.20441	0.00394
C	4.30490	1.16048	0.00663
H	3.47645	-2.69874	-0.00894
H	5.82209	-1.88877	-0.00357
H	6.34566	0.51618	0.00613
H	4.52525	2.22268	0.01090
H	-5.91474	-1.63997	1.01758
C	-5.79246	-1.19891	0.02451
H	-6.66991	-0.59746	-0.21585
C	-4.58044	1.00479	-0.00815
N	-4.62162	-0.35730	-0.01596
H	-5.48677	1.59305	-0.01096
C	-3.25716	1.36483	-0.00161
N	-3.41683	-0.91200	-0.00919
C	-2.57213	0.12535	-0.00313
H	-5.68522	-1.99767	-0.71080
H	-2.82103	2.35064	-0.00300
H	0.72035	-1.54939	-0.00727

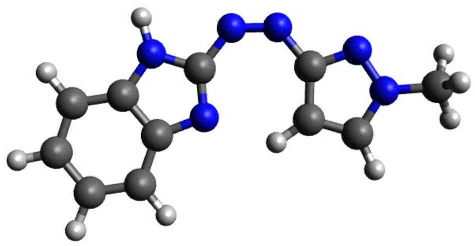

1-*Z*-**3pz**

C	2.14053	0.55077	-0.00107
C	2.86060	-0.67047	0.00459
N	0.78873	0.31265	-0.00620
C	0.66700	-1.00115	-0.00384
N	1.89189	-1.63076	0.00248
N	-0.38057	-1.91664	-0.00623
N	-1.61889	-1.70376	-0.00839
C	4.25483	-0.71612	0.01059
C	4.91553	0.50188	0.01073
C	4.21647	1.72512	0.00512
C	2.83333	1.76724	-0.00081
H	4.79787	-1.65566	0.01491
H	6.00112	0.51468	0.01528
H	4.78069	2.65253	0.00551
H	2.28931	2.70591	-0.00509
H	-6.05965	-0.17170	-0.48214
C	-3.23252	1.50241	-0.02273
C	-2.00504	0.89465	-0.01949
H	-5.88604	1.60314	-0.42889
N	-4.16788	0.50975	-0.01719
C	-2.30360	-0.49865	-0.00839
H	-3.51821	2.54461	-0.03269
H	-1.02290	1.33594	-0.03102
C	-5.60184	0.66340	0.04734
N	-3.64137	-0.69609	-0.00438
H	-5.94488	0.66343	1.08610
H	1.98758	-2.63340	0.00507

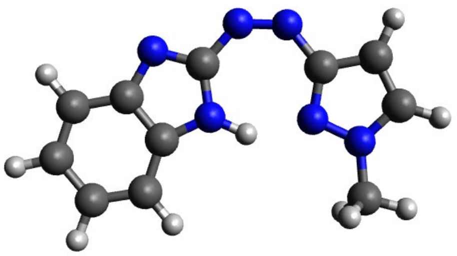

2-*Z*-**3pz**

C	-2.62496	-0.72417	-0.00009
C	-1.85498	0.47006	0.00011
N	-1.81211	-1.81577	-0.00005
C	-0.57871	-1.32548	0.00013
N	-0.55928	0.05357	0.00023
N	0.46068	-2.23923	0.00024
N	1.70426	-2.04665	0.00021
C	-2.44356	1.73796	0.00014
C	-3.82668	1.77869	-0.00003
C	-4.60863	0.60211	-0.00023
C	-4.02734	-0.65030	-0.00026
H	-1.84948	2.64633	0.00029
H	-4.32774	2.74216	-0.00002
H	-5.69048	0.69272	-0.00036
H	-4.61886	-1.55963	-0.00041
H	2.57223	2.93748	-0.89158
C	4.27845	0.36730	-0.00038
C	3.84681	-0.93537	-0.00036
H	4.11264	2.98800	0.00024
N	3.16865	1.14727	0.00001
C	2.43444	-0.85400	0.00003
H	5.26670	0.80288	-0.00062
H	4.42948	-1.84264	-0.00056
C	3.09841	2.58839	0.00006
N	2.04699	0.43344	0.00026
H	2.57197	2.93736	0.89158
H	0.30977	0.59583	0.00038

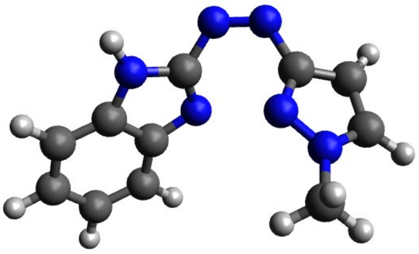

3-*Z*-**3pz**

C	1.72707	0.12215	-0.63866
C	2.32558	-0.54708	0.45845
N	0.48159	-0.39709	-0.90620
C	0.32335	-1.34031	-0.01051
N	1.40442	-1.50021	0.81626
N	-0.67563	-2.32454	0.04787
N	-1.88279	-2.07397	-0.15171
C	3.58077	-0.20345	0.95290
C	4.23996	0.83486	0.30917
C	3.66584	1.50722	-0.78474
C	2.41476	1.16265	-1.27131
H	4.02827	-0.72160	1.79523
H	5.22395	1.13346	0.65756
H	4.21929	2.31412	-1.25535
H	1.96568	1.67776	-2.11392
H	-2.97244	2.24022	2.24082
C	-3.65906	0.97538	-0.55490
C	-3.46559	-0.32613	-0.95624
H	-3.20660	3.21312	0.76514
N	-2.75266	1.22140	0.42578
C	-2.37866	-0.76197	-0.17151
H	-4.36010	1.73144	-0.87648
H	-3.99224	-0.87923	-1.71770
C	-2.61797	2.41663	1.22164
N	-1.96777	0.17942	0.68023
H	-1.56761	2.71076	1.25221
H	1.47542	-2.19477	1.54107

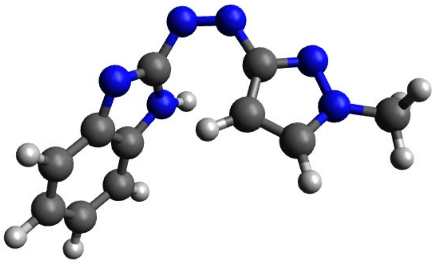

4-*Z*-**3pz**

C	2.29407	0.44281	-0.53452
C	1.97589	-0.10355	0.73053
N	1.38854	1.43118	-0.88236
C	0.54376	1.45591	0.11120
N	0.84386	0.57753	1.12266
N	-0.52247	2.37124	0.25956
N	-1.70949	1.98016	0.26550
C	2.73310	-1.10654	1.32758
C	3.83051	-1.56702	0.61091
C	4.15954	-1.04059	-0.64909
C	3.40294	-0.03588	-1.23506
H	2.48139	-1.51484	2.30135
H	4.44747	-2.35297	1.03555
H	5.02770	-1.42959	-1.17220
H	3.65444	0.37836	-2.20565
H	-5.09185	-1.40623	1.09415
C	-2.38778	-1.39074	-0.76766
C	-1.43674	-0.40207	-0.73790
H	-4.62546	-2.64055	-0.10468
N	-3.48112	-0.91117	-0.11482
C	-2.07325	0.65970	-0.03861
H	-2.37519	-2.38213	-1.19699
H	-0.45188	-0.43078	-1.17661
C	-4.75165	-1.57176	0.07134
N	-3.32339	0.33051	0.31324
H	-5.49516	-1.17218	-0.62326
H	0.31459	0.45313	1.96945

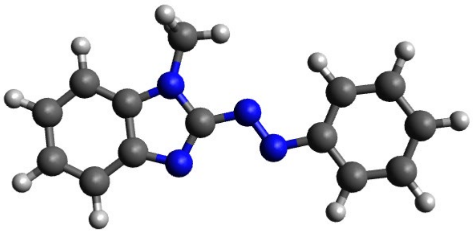

1-*E*-**8a**

C	2.55073	-0.90085	0.00000
C	2.77123	0.50003	0.00004
N	1.21134	-1.17998	-0.00002
C	0.62983	0.00017	0.00001
N	1.52127	1.05668	0.00004
N	-0.71577	0.32486	0.00000
N	-1.49000	-0.66600	-0.00003
C	1.21452	2.46540	0.00007
H	0.13148	2.57715	0.00006
H	1.63041	2.94569	-0.89096
H	1.63040	2.94565	0.89113
C	4.04766	1.06262	0.00007
C	5.11336	0.17636	0.00006
C	4.91592	-1.21950	0.00002
C	3.64755	-1.77256	-0.00001
H	4.20315	2.13689	0.00009
H	6.12683	0.56587	0.00008
H	5.78408	-1.87142	0.00002
H	3.48889	-2.84569	-0.00003
H	-5.77478	-2.07985	-0.00011
C	-5.10116	-1.22857	-0.00008
C	-3.72640	-1.42989	-0.00007
H	-3.29159	-2.42426	-0.00009
C	-2.85695	-0.33547	-0.00004
C	-3.37109	0.96903	-0.00001
H	-2.68071	1.80503	0.00002
C	-4.74258	1.16242	-0.00002
H	-5.14612	2.17076	0.00000
C	-5.61025	0.06695	-0.00006
H	-6.68417	0.22844	-0.00006

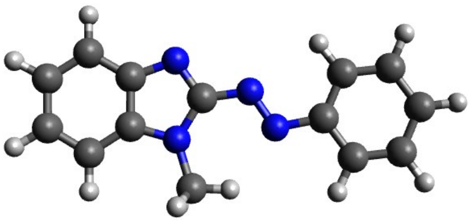

2-*E*-**8a**

C	-2.71268	-0.94418	-0.00013
C	-2.67192	0.46971	0.00005
N	-1.44602	-1.45240	-0.00015
C	-0.64986	-0.39955	0.00001
N	-1.34182	0.80495	0.00013
N	0.71411	-0.62313	0.00003
N	1.46291	0.38823	-0.00012
C	-0.87679	2.17680	0.00042
H	0.20871	2.18121	0.00046
H	-1.25097	2.69205	0.89092
H	-1.25087	2.69240	-0.88992
C	-3.82591	1.25341	0.00015
C	-5.03433	0.57333	0.00003
C	-5.09514	-0.83501	-0.00017
C	-3.94712	-1.60694	-0.00025
H	-3.78663	2.33794	0.00030
H	-5.96029	1.14050	0.00009
H	-6.06725	-1.31814	-0.00026
H	-3.98277	-2.69116	-0.00040
H	5.74647	1.84135	-0.00075
C	5.07708	0.98670	-0.00043
C	3.70128	1.18180	-0.00045
H	3.27042	2.17825	-0.00079
C	2.83842	0.08323	-0.00006
C	3.35614	-1.21918	0.00038
H	2.66470	-2.05467	0.00069
C	4.72896	-1.40509	0.00041
H	5.13582	-2.41197	0.00076
C	5.59214	-0.30647	0.00000
H	6.66679	-0.46264	0.00004

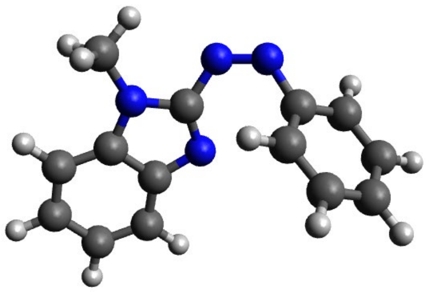

1-*Z*-**8a**

C	-1.50921	-0.76466	-0.43141
C	-2.35640	0.25108	0.07743
N	-0.24816	-0.27390	-0.65070
C	-0.31989	0.98952	-0.29484
N	-1.57380	1.37500	0.13177
N	0.61309	2.01485	-0.47880
N	1.84596	1.82563	-0.52281
C	-1.97810	2.69295	0.55519
H	-1.20078	3.39684	0.25714
H	-2.11079	2.73521	1.64098
H	-2.92002	2.96182	0.06951
C	-3.69046	0.02038	0.41121
C	-4.16497	-1.26659	0.20852
C	-3.33989	-2.28821	-0.30211
C	-2.01466	-2.05542	-0.62748
H	-4.32842	0.80428	0.80695
H	-5.19875	-1.49417	0.44986
H	-3.75828	-3.28048	-0.43987
H	-1.37276	-2.83761	-1.01844
H	4.96069	-1.40332	-1.23624
C	4.18907	-1.00916	-0.58195
C	3.47749	0.12231	-0.96074
H	3.68326	0.63296	-1.89588
C	2.45888	0.60558	-0.13942
C	2.20880	0.00639	1.09889
H	1.44471	0.41603	1.75193
C	2.95264	-1.09732	1.48694
H	2.76559	-1.55729	2.45263
C	3.93195	-1.61943	0.64260
H	4.50435	-2.48990	0.94808

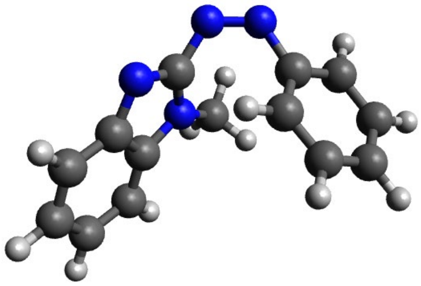

2-*Z*-**8a**

C	2.17234	0.52669	-0.61769
C	1.75131	-0.07785	0.58774
N	1.27132	1.49501	-1.01132
C	0.32890	1.44293	-0.10704
N	0.55886	0.53814	0.90563
N	-0.77118	2.33221	-0.09044
N	-1.94742	1.91702	-0.08582
C	-0.23932	0.27525	2.07672
H	-0.90507	1.11864	2.26272
H	0.41932	0.15803	2.94098
H	-0.84200	-0.62973	1.94956
C	2.48427	-1.07428	1.22637
C	3.66615	-1.46598	0.61077
C	4.09876	-0.88004	-0.59125
C	3.36496	0.11652	-1.21789
H	2.14857	-1.53485	2.15024
H	4.26733	-2.24656	1.06681
H	5.02993	-1.21752	-1.03593
H	3.69592	0.57556	-2.14346
H	-4.70090	-1.53690	0.90602
C	-3.84201	-1.19812	0.33501
C	-3.40766	0.11568	0.45164
H	-3.91569	0.82734	1.09501
C	-2.28271	0.54483	-0.25784
C	-1.63899	-0.32143	-1.15087
H	-0.80019	0.02711	-1.74398
C	-2.10417	-1.62293	-1.28625
H	-1.61354	-2.29374	-1.98468
C	-3.18961	-2.06986	-0.53417
H	-3.53821	-3.09251	-0.64104

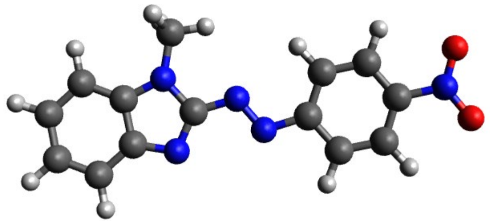

1-*E*-**8e**

C	-3.68719	-0.88075	-0.00002
C	-3.87611	0.52617	0.00005
N	-2.35726	-1.18955	-0.00012
C	-1.74864	-0.02142	-0.00013
N	-2.61617	1.05525	-0.00002
N	-0.39885	0.26674	-0.00023
N	0.35293	-0.74209	-0.00031
C	-2.27953	2.45804	0.00000
H	-1.19471	2.54896	-0.00007
H	-2.68540	2.94572	0.89127
H	-2.68552	2.94577	-0.89119
C	-5.14077	1.11701	0.00016
C	-6.22346	0.25365	0.00021
C	-6.05742	-1.14813	0.00015
C	-4.80362	-1.72957	0.00003
H	-5.27341	2.19415	0.00022
H	-7.22841	0.66442	0.00030
H	-6.94057	-1.77932	0.00019
H	-4.66827	-2.80574	-0.00002
H	4.64100	-2.20430	0.00033
C	3.95339	-1.36756	0.00015
C	2.57796	-1.54097	0.00002
H	2.13049	-2.52904	0.00008
C	1.72564	-0.43162	-0.00017
C	2.25675	0.86729	-0.00027
H	1.57929	1.71295	-0.00046
C	3.62681	1.04942	-0.00019
H	4.07605	2.03523	-0.00027
C	4.45415	-0.07213	0.00002
N	5.90331	0.12376	0.00015
O	6.31672	1.27262	0.00033
O	6.60752	-0.87314	0.00007

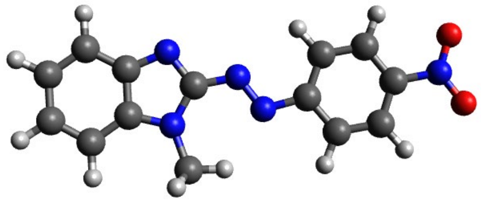

2-*E*-**8e**

N	5.89718	-0.24999	-0.00006
O	6.32904	-1.39159	0.00016
O	6.58520	0.75860	-0.00032
H	-2.47996	2.71133	0.89098
C	3.92610	1.21110	-0.00035
C	4.44524	-0.07707	-0.00003
C	2.54857	1.36507	-0.00031
H	4.60208	2.05722	-0.00065
C	3.63349	-1.20966	0.00036
C	1.71329	0.24361	0.00007
C	2.26043	-1.04821	0.00043
H	2.09513	2.35063	-0.00056
N	0.33040	0.51536	0.00012
C	-2.08510	2.21264	0.00036
H	4.09519	-2.18970	0.00065
N	-0.38499	-0.52124	-0.00001
H	-1.00095	2.26300	0.00037
C	-1.75186	-0.35218	-0.00002
N	-2.49389	0.82207	0.00013
H	1.58971	-1.89983	0.00073
C	-3.80682	0.43065	0.00005
C	-4.99375	1.16481	0.00014
N	-2.50465	-1.43929	-0.00020
C	-3.78837	-0.98536	-0.00016
C	-6.17018	0.43278	0.00001
H	-5.00171	2.24985	0.00030
C	-4.99461	-1.70138	-0.00028
C	-6.17218	-0.97859	-0.00020
H	-7.11972	0.95931	0.00007
H	-4.98385	-2.78600	-0.00044
H	-7.12352	-1.50110	-0.00030
H	-2.47995	2.71161	-0.89012

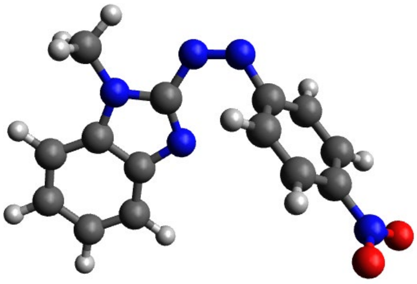

1-*Z*-**8e**

C	1.98985	0.90036	-0.36067
C	3.16352	0.24091	0.08484
N	0.95923	0.00935	-0.48459
C	1.47741	-1.14794	-0.13220
N	2.81276	-1.07599	0.20603
N	0.92218	-2.42581	-0.17898
N	-0.30477	-2.62755	-0.27746
C	3.66988	-2.16263	0.61726
H	3.13407	-3.09736	0.45605
H	3.93226	-2.06965	1.67547
H	4.58494	-2.15689	0.01900
C	4.36423	0.91598	0.31152
C	4.35890	2.27932	0.06914
C	3.20191	2.95192	-0.37859
C	2.01350	2.28123	-0.59897
H	5.25629	0.40402	0.65754
H	5.26953	2.84855	0.22818
H	3.25239	4.02244	-0.55061
H	1.11905	2.79089	-0.94049
H	-3.84348	-0.24701	-1.93173
C	-3.17200	-0.46744	-1.11089
C	-2.13452	-1.37571	-1.23955
H	-1.96767	-1.90235	-2.17266
C	-1.27406	-1.59945	-0.16311
C	-1.49314	-0.97288	1.06886
H	-0.83476	-1.18495	1.90452
C	-2.54450	-0.08589	1.20878
H	-2.74282	0.42232	2.14458
C	-3.36250	0.16531	0.11188
O	-5.16437	1.31522	-0.72425
N	-4.46126	1.11165	0.25401
O	-4.61159	1.64602	1.34265

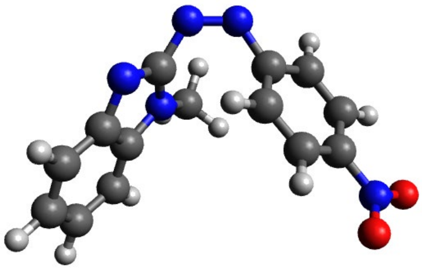

2-*Z*-**8e**

C	2.89484	-0.09212	-0.66825
C	2.33858	-0.30329	0.61317
N	2.41822	1.08242	-1.21188
C	1.57673	1.53060	-0.31840
N	1.48867	0.76257	0.82222
N	0.88678	2.75468	-0.47329
N	-0.35576	2.80840	-0.39178
C	0.75861	1.02636	2.03784
H	0.45620	2.07364	2.06508
H	1.40693	0.83283	2.89624
H	-0.13416	0.39703	2.10726
C	2.67034	-1.39819	1.40731
C	3.57644	-2.30077	0.86776
C	4.13391	-2.11223	-0.40969
C	3.80497	-1.01500	-1.19028
H	2.23511	-1.55224	2.38959
H	3.85724	-3.17691	1.44388
H	4.83887	-2.84547	-0.78881
H	4.23543	-0.86101	-2.17396
H	-4.04917	0.70377	1.24281
C	-3.18241	0.67752	0.59375
C	-2.29424	1.73720	0.50757
H	-2.44730	2.64532	1.08128
C	-1.17145	1.64513	-0.32134
C	-0.97497	0.51918	-1.13275
H	-0.12747	0.47265	-1.80816
C	-1.87874	-0.52946	-1.07285
H	-1.76198	-1.41595	-1.68438
C	-2.95651	-0.44166	-0.19889
O	-4.82730	-1.45897	0.66036
N	-3.89529	-1.56079	-0.12111
O	-3.68329	-2.52406	-0.83907

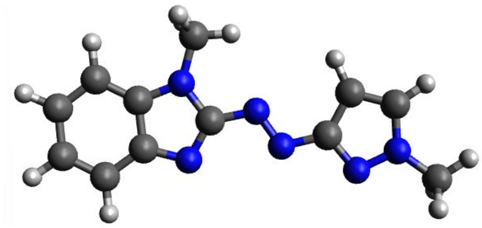

1-*E*-**8pz**

C	-2.63426	-0.94016	0.00193
C	-2.91872	0.44895	-0.00244
N	-1.28299	-1.15799	0.00413
C	-0.75616	0.04689	0.00124
N	-1.69504	1.06191	-0.00280
N	0.57214	0.43739	0.00174
N	1.39657	-0.51539	0.00526
C	-1.45090	2.48247	-0.00670
H	-0.37340	2.64009	-0.00633
H	-1.88817	2.94704	0.88259
H	-1.88679	2.94188	-0.89933
C	-4.21929	0.95250	-0.00541
C	-5.24394	0.01854	-0.00390
C	-4.98277	-1.36636	0.00044
C	-3.68993	-1.86071	0.00338
H	-4.42349	2.01865	-0.00874
H	-6.27412	0.36156	-0.00609
H	-5.82001	-2.05763	0.00147
H	-3.48213	-2.92543	0.00672
H	6.23008	-1.47741	-1.03226
C	6.05217	-1.07955	-0.02934
H	6.86378	-0.40357	0.24280
C	4.62743	0.99322	0.01107
N	4.80373	-0.35802	0.01667
H	5.47049	1.66918	0.01518
C	3.27473	1.21728	0.00508
N	3.66136	-1.02992	0.00860
C	2.71388	-0.08504	0.00447
H	6.01104	-1.90643	0.68077
H	2.74879	2.15796	0.00915

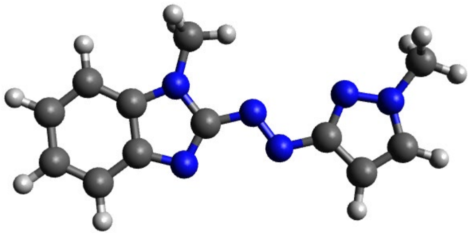

2-*E*-**8pz**

C	-2.70925	-0.85446	-0.00006
C	-2.82008	0.55990	0.00005
N	-1.39548	-1.23778	-0.00010
C	-0.72455	-0.10547	-0.00001
N	-1.53100	1.01683	0.00009
N	0.63835	0.12951	0.00001
N	1.34833	-0.90958	-0.00008
C	-1.10339	2.39446	0.00020
H	-0.01386	2.40754	0.00019
H	-1.47715	2.90823	0.89128
H	-1.47715	2.90838	-0.89080
C	-4.04903	1.21979	0.00011
C	-5.18098	0.41969	0.00005
C	-5.09284	-0.98712	-0.00007
C	-3.87108	-1.63726	-0.00012
H	-4.11948	2.30301	0.00020
H	-6.16099	0.88721	0.00009
H	-6.00910	-1.56966	-0.00011
H	-3.79690	-2.71958	-0.00021
H	5.40301	1.97516	0.88980
C	5.54753	1.35833	0.00010
H	6.55844	0.94957	0.00006
C	4.87970	-1.06291	-0.00010
N	4.60209	0.26849	0.00001
H	5.89681	-1.42615	-0.00013
C	3.66890	-1.71234	-0.00015
N	3.30264	0.53707	0.00003
C	2.71638	-0.66899	-0.00006
H	5.40301	1.97531	-0.88951
H	3.47086	-2.77259	-0.00023

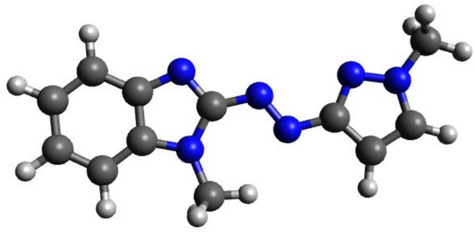

3-*E*-**8pz**

C	-2.75233	-0.99711	-0.00189
C	-2.79025	0.41670	0.00132
N	-1.45948	-1.43430	-0.00298
C	-0.72137	-0.34016	-0.00075
N	-1.48087	0.82504	0.00205
N	0.65471	-0.48886	-0.00140
N	1.33027	0.57475	-0.00241
C	-1.09674	2.22173	0.00611
H	-0.01350	2.29003	0.00941
H	-1.50388	2.71272	0.89604
H	-1.49806	2.71664	-0.88431
C	-3.98604	1.13482	0.00324
C	-5.15536	0.38888	0.00177
C	-5.13769	-1.02027	-0.00146
C	-3.94805	-1.72704	-0.00333
H	-4.00715	2.21993	0.00563
H	-6.11130	0.90400	0.00309
H	-6.08125	-1.55717	-0.00250
H	-3.92334	-2.81162	-0.00580
H	5.45356	-2.18752	-0.84849
C	5.60858	-1.53219	0.01086
H	6.60455	-1.09108	-0.03948
C	4.85644	0.86515	-0.00664
N	4.62676	-0.47438	-0.00199
H	5.85930	1.26600	-0.00958
C	3.62229	1.47050	-0.00728
N	3.33793	-0.78927	0.00144
C	2.70877	0.39241	-0.00235
H	5.51295	-2.11800	0.92781
H	3.39318	2.52472	-0.01241

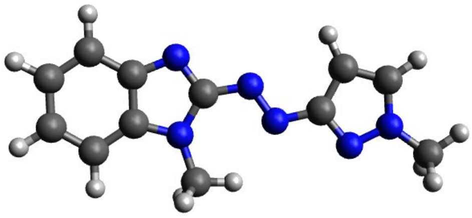

4-*E*-**8pz**

C	2.88824	-0.89796	-0.00311
C	2.76220	0.51086	0.00042
N	1.65406	-1.48164	-0.00346
C	0.79660	-0.47837	-0.00024
N	1.41422	0.76583	0.00222
N	-0.55380	-0.77981	0.00028
N	-1.34678	0.20008	0.00384
C	0.86957	2.10947	0.00589
H	-0.21483	2.05616	0.00610
H	1.21506	2.64703	-0.88300
H	1.21552	2.64242	0.89738
C	3.86686	1.36247	0.00152
C	5.11462	0.75699	-0.00105
C	5.26015	-0.64473	-0.00458
C	4.16037	-1.48453	-0.00565
H	3.76179	2.44263	0.00426
H	6.00449	1.37929	-0.00030
H	6.25951	-1.06884	-0.00648
H	4.26185	-2.56464	-0.00836
H	-6.13076	1.35760	-1.01476
C	-5.98472	0.91473	-0.02569
H	-6.81943	0.25033	0.20082
C	-4.62157	-1.19867	0.00859
N	-4.75838	0.15688	0.01461
H	-5.48449	-1.84903	0.01182
C	-3.27610	-1.46533	0.00290
N	-3.59436	0.79456	0.00758
C	-2.67987	-0.18113	0.00305
H	-5.94018	1.71078	0.71916
H	-2.77125	-2.41777	0.00544

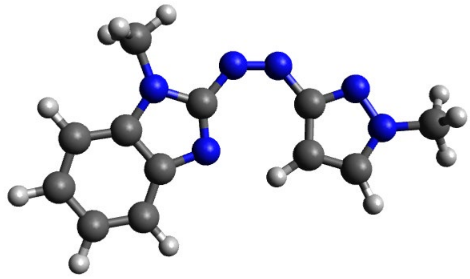

1-*Z*-**8pz**

C	-1.94486	-0.84694	-0.12602
C	-2.72596	0.31837	0.05742
N	-0.62022	-0.51751	-0.23512
C	-0.57596	0.79849	-0.12768
N	-1.83558	1.35436	0.04179
N	0.43369	1.74173	-0.22575
N	1.67996	1.57892	-0.23442
C	-2.15961	2.75366	0.18046
H	-1.24701	3.32339	0.01165
H	-2.54376	2.96143	1.18405
H	-2.91628	3.03461	-0.55791
C	-4.11257	0.27568	0.20876
C	-4.70237	-0.97771	0.16431
C	-3.94053	-2.14867	-0.02110
C	-2.56499	-2.10141	-0.16794
H	-4.70180	1.17559	0.35307
H	-5.77920	-1.06110	0.27518
H	-4.44934	-3.10730	-0.04818
H	-1.97388	-2.99997	-0.31118
H	6.19865	0.21332	-0.38370
C	3.42863	-1.53613	0.13037
C	2.17718	-0.99053	0.01886
H	6.09883	-1.55187	-0.14543
N	4.31852	-0.50414	0.07539
C	2.41335	0.40991	-0.09742
H	3.75963	-2.55973	0.23334
H	1.21826	-1.47861	-0.01242
C	5.75322	-0.58135	0.21421
N	3.73994	0.67017	-0.05956
H	6.04861	-0.45465	1.25989

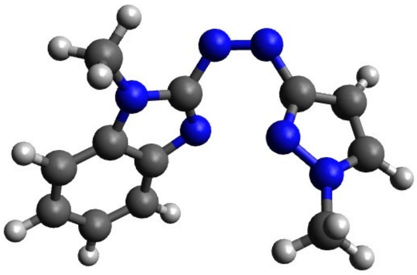

2-*Z*-**8pz**

C	-1.58131	-0.53574	-0.73689
C	-2.24105	0.30774	0.19063
N	-0.36128	-0.00975	-1.08546
C	-0.28345	1.10575	-0.39883
N	-1.39473	1.37591	0.36322
N	0.66105	2.13080	-0.54689
N	1.88485	1.91243	-0.66817
C	-1.59622	2.52137	1.21332
H	-0.88885	3.29604	0.91408
H	-1.43177	2.26966	2.26602
H	-2.61584	2.89560	1.08882
C	-3.48555	0.00038	0.73484
C	-4.07418	-1.18306	0.31014
C	-3.44012	-2.02919	-0.61794
C	-2.19840	-1.72046	-1.15128
H	-3.97564	0.65069	1.45277
H	-5.04690	-1.46245	0.70334
H	-3.93850	-2.94553	-0.91955
H	-1.70456	-2.37032	-1.86612
H	3.10141	-1.67908	2.66784
C	3.82023	-1.04730	-0.33130
C	3.57509	0.11597	-1.02357
H	3.44349	-2.94459	1.45950
N	2.89838	-1.11244	0.66350
C	2.44553	0.65942	-0.37888
H	4.56658	-1.81750	-0.45882
H	4.09618	0.50872	-1.88224
C	2.79626	-2.10254	1.70709
N	2.05613	-0.08410	0.65853
H	1.76260	-2.44526	1.77727

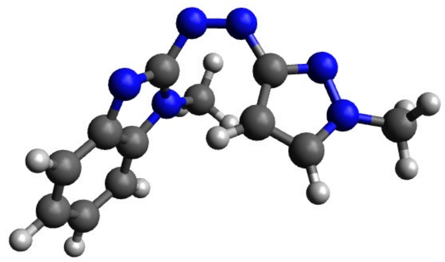

3-*Z*-**8pz**

C	2.28459	0.37469	-0.72667
C	1.96313	-0.07557	0.57346
N	1.38295	1.33713	-1.14171
C	0.54013	1.43327	-0.14847
N	0.83646	0.63452	0.93308
N	-0.52514	2.36003	-0.08962
N	-1.71355	1.97091	-0.07543
C	0.10700	0.50414	2.16884
H	-0.55321	1.36287	2.29335
H	0.80943	0.48016	3.00593
H	-0.49633	-0.40978	2.17337
C	2.71060	-1.04041	1.24249
C	3.80679	-1.55814	0.56350
C	4.14121	-1.12437	-0.73016
C	3.39174	-0.15959	-1.38878
H	2.45078	-1.38079	2.24007
H	4.41666	-2.31772	1.04296
H	5.00741	-1.55559	-1.22268
H	3.64770	0.18156	-2.38637
H	-5.07482	-1.35859	1.04462
C	-2.35737	-1.49004	-0.78451
C	-1.41412	-0.49465	-0.84370
H	-4.59736	-2.68536	-0.04628
N	-3.46361	-0.95614	-0.19932
C	-2.06872	0.62707	-0.26421
H	-2.33172	-2.51935	-1.11198
H	-0.42452	-0.55840	-1.26789
C	-4.73053	-1.60648	0.03986
N	-3.32088	0.32389	0.10331
H	-5.47444	-1.27082	-0.68723

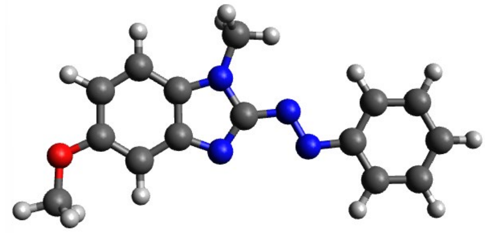

1-*E*-**13a**

C	-1.92640	-0.31704	-0.00010
C	-1.95152	1.09789	-0.00008
N	-0.64053	-0.77706	-0.00003
C	0.10065	0.31408	0.00003
N	-0.63723	1.48050	0.00000
N	1.47540	0.45070	0.00014
N	2.10951	-0.63639	0.00003
C	-0.14170	2.83410	0.00007
H	0.94646	2.79672	0.00009
H	-0.48834	3.36655	0.89116
H	-0.48831	3.36663	-0.89097
C	-3.14703	1.81985	-0.00013
C	-4.31746	1.08872	-0.00019
C	-4.31083	-0.32848	-0.00021
C	-3.12556	-1.04697	-0.00018
H	-3.16655	2.90499	-0.00010
H	-5.28356	1.58187	-0.00021
O	-5.54797	-0.88912	-0.00033
H	-3.09092	-2.12896	-0.00023
H	6.16764	-2.60970	-0.00048
C	5.61387	-1.67595	-0.00027
C	4.22456	-1.69192	-0.00024
H	3.66112	-2.61949	-0.00042
C	3.50823	-0.49151	0.00002
C	4.19266	0.73234	0.00028
H	3.62026	1.65315	0.00050
C	5.57779	0.74098	0.00025
H	6.11211	1.68661	0.00045
C	6.29181	-0.46019	-0.00002
H	7.37769	-0.44355	-0.00004
C	-5.61735	-2.29482	0.00064
H	-6.67823	-2.54800	0.00107
H	-5.14273	-2.72169	0.89372
H	-5.14316	-2.72296	-0.89208

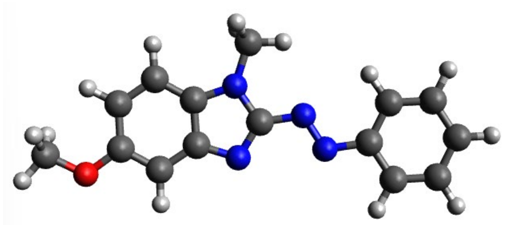

2-*E*-**13a**

C	1.83364	-0.52763	-0.00002
C	1.92633	0.88848	0.00003
N	0.52333	-0.92403	-0.00003
C	-0.16053	0.20097	0.00001
N	0.62988	1.33272	0.00005
N	-1.52963	0.40381	0.00001
N	-2.21201	-0.65267	-0.00003
C	0.19966	2.70786	0.00009
H	-0.88905	2.72345	0.00009
H	0.57144	3.22365	-0.89089
H	0.57144	3.22359	0.89111
C	3.14940	1.54946	0.00006
C	4.29563	0.76611	0.00003
C	4.21918	-0.64522	-0.00002
C	2.99488	-1.30196	-0.00004
H	3.22016	2.63252	0.00010
H	5.26142	1.25674	0.00005
O	5.31702	-1.44915	-0.00004
H	2.95526	-2.38485	-0.00008
H	-6.35367	-2.44323	-0.00010
C	-5.75874	-1.53519	-0.00006
C	-4.37149	-1.61306	-0.00006
H	-3.84965	-2.56465	-0.00009
C	-3.60299	-0.44550	-0.00002
C	-4.23166	0.80785	0.00002
H	-3.61855	1.70206	0.00005
C	-5.61496	0.87814	0.00002
H	-6.10684	1.84649	0.00005
C	-6.38151	-0.29030	-0.00002
H	-7.46557	-0.22530	-0.00002
C	6.58486	-0.84198	-0.00002
H	6.74293	-0.22380	0.89376
H	6.74293	-0.22374	-0.89375
H	7.31034	-1.65664	-0.00005

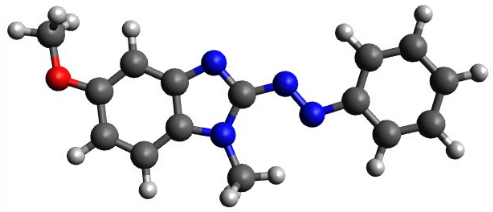

3-*E*-**13a**

C	-2.03688	-0.46967	-0.00009
C	-1.84420	0.92860	-0.00004
N	-0.83448	-1.10979	-0.00011
C	0.07365	-0.14668	-0.00007
N	-0.48552	1.12219	-0.00003
N	1.40366	-0.51494	-0.00007
N	2.25965	0.40864	0.00004
C	0.12320	2.43645	-0.00000
H	1.20301	2.32445	-0.00005
H	-0.19353	2.98906	0.89043
H	-0.19361	2.98913	-0.89036
C	-2.91714	1.82269	-0.00002
C	-4.18468	1.27544	-0.00005
C	-4.39482	-0.12601	-0.00007
C	-3.33140	-1.01410	-0.00012
H	-2.77455	2.89840	0.00002
H	-5.06478	1.90966	-0.00004
H	-3.45896	-2.08915	-0.00017
H	6.67781	1.37953	0.00029
C	5.91805	0.60406	0.00018
C	4.57230	0.94997	0.00017
H	4.25399	1.98790	0.00028
C	3.59268	-0.04614	0.00003
C	3.96396	-1.39776	-0.00011
H	3.18431	-2.15159	-0.00022
C	5.30783	-1.73428	-0.00011
H	5.60099	-2.77999	-0.00022
C	6.28733	-0.73805	0.00003
H	-7.07070	-1.96137	0.00048
C	-5.98381	-1.87107	0.00042
O	-5.70286	-0.49206	-0.00018
H	-5.57945	-2.36501	0.89359
H	-5.57946	-2.36577	-0.89233
H	7.33818	-1.01206	0.00004

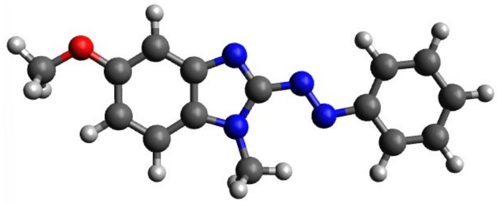

4-*E*-**13a**

C	-1.95179	-0.71216	-0.00009
C	-1.83435	0.69825	-0.00012
N	-0.71252	-1.28554	-0.00008
C	0.13809	-0.27448	-0.00008
N	-0.48614	0.96392	-0.00011
N	1.48823	-0.57095	-0.00006
N	2.29045	0.39862	0.00005
C	0.04914	2.30909	-0.00016
H	1.13345	2.25813	-0.00038
H	-0.29777	2.84369	0.89025
H	-0.29815	2.84376	-0.89039
C	-2.94911	1.52895	-0.00014
C	-4.19684	0.91846	-0.00014
C	-4.32817	-0.48855	-0.00014
C	-3.21144	-1.31436	-0.00008
H	-2.86436	2.61080	-0.00015
H	-5.08109	1.54440	-0.00014
H	-3.32661	-2.39187	-0.00009
H	6.64675	1.61734	0.00035
C	5.93181	0.80039	0.00023
C	4.56868	1.06986	0.00021
H	4.19221	2.08812	0.00032
C	3.64730	0.01978	0.00006
C	4.09371	-1.30884	-0.00008
H	3.35767	-2.10533	-0.00020
C	5.45439	-1.56898	-0.00006
H	5.80616	-2.59641	-0.00017
C	6.37591	-0.51884	0.00010
H	-6.76387	0.29590	-0.89320
C	-6.69707	-0.33868	0.00058
O	-5.53128	-1.12427	-0.00012
H	-6.76281	0.29595	0.89440
H	-7.53355	-1.03885	0.00110
H	7.44043	-0.73315	0.00010

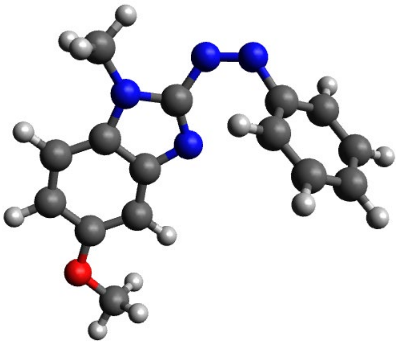

1-*Z*-**13a**

C	-1.15604	0.04104	-0.26518
C	-1.64916	1.31090	0.11452
N	0.18708	0.10362	-0.51561
C	0.50691	1.36452	-0.30130
N	-0.56647	2.14956	0.05994
N	1.69792	2.04061	-0.56805
N	2.81482	1.48404	-0.62676
C	-0.55385	3.56577	0.33106
H	0.39832	3.96665	-0.01661
H	-0.66259	3.76253	1.40242
H	-1.37387	4.05023	-0.20610
C	-2.99000	1.51548	0.44651
C	-3.82518	0.41885	0.37580
C	-3.34807	-0.85835	-0.00946
C	-2.01605	-1.06536	-0.33392
H	-3.36942	2.48751	0.74479
H	-4.87941	0.50466	0.61609
O	-4.30014	-1.82650	-0.02366
H	-1.62240	-2.03012	-0.62743
H	4.75731	-2.57973	-1.22737
C	4.17976	-1.93239	-0.57435
C	3.82259	-0.66111	-1.00645
H	4.11928	-0.29219	-1.98296
C	3.04756	0.15700	-0.18439
C	2.69976	-0.26648	1.10199
H	2.13252	0.39303	1.75103
C	3.09614	-1.52071	1.54004
H	2.83422	-1.84572	2.54255
C	3.82171	-2.36443	0.69965
H	4.12128	-3.34909	1.04529
C	-3.89659	-3.12080	-0.40161
H	-4.78980	-3.74487	-0.35488
H	-3.13790	-3.52300	0.28238
H	-3.49688	-3.13643	-1.42389

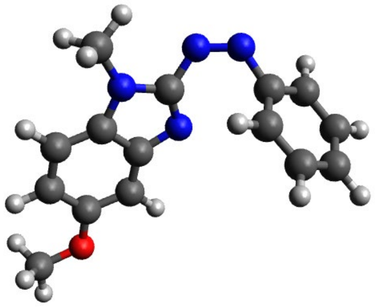

2-*Z*-**13a**

C	-1.04696	-0.04634	-0.36369
C	-1.55779	1.18982	0.10751
N	0.29894	0.05417	-0.60714
C	0.59764	1.29840	-0.30115
N	-0.48347	2.04450	0.11390
N	1.78324	2.00444	-0.52583
N	2.90850	1.46626	-0.57763
C	-0.48783	3.43783	0.48263
H	0.45800	3.87510	0.16209
H	-0.59701	3.55984	1.56520
H	-1.31490	3.94817	-0.01891
C	-2.89474	1.35208	0.45051
C	-3.72830	0.25109	0.30247
C	-3.23379	-0.98434	-0.17161
C	-1.89472	-1.14446	-0.50825
H	-3.28579	2.29561	0.81758
H	-4.77566	0.35335	0.55936
O	-4.01380	-2.08792	-0.32772
H	-1.53099	-2.10011	-0.86687
H	4.95632	-2.53617	-1.22453
C	4.34124	-1.92104	-0.57478
C	3.97657	-0.64311	-0.97964
H	4.30361	-0.23650	-1.93114
C	3.15264	0.13230	-0.16370
C	2.76300	-0.33886	1.09378
H	2.15863	0.28846	1.74125
C	3.16683	-1.59906	1.50698
H	2.87207	-1.96194	2.48705
C	3.94132	-2.40095	0.66931
H	4.24558	-3.39105	0.99460
C	-5.37819	-1.98785	-0.00335
H	-5.80887	-2.97035	-0.20108
H	-5.89253	-1.24158	-0.62330
H	-5.53003	-1.73821	1.05520

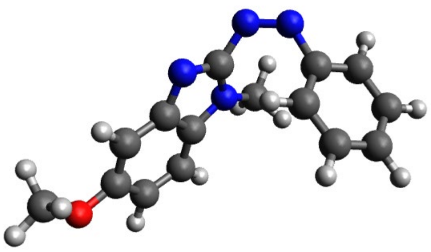

3-*Z*-**13a**

C	-1.52166	-0.71253	-0.34120
C	-0.98350	-0.29306	0.89145
N	-0.61485	-1.49676	-1.02010
C	0.44257	-1.51014	-0.24644
N	0.28860	-0.82861	0.93740
N	1.59767	-2.26792	-0.54372
N	2.72977	-1.74464	-0.58868
C	1.21562	-0.70147	2.03332
H	1.96489	-1.49146	1.97291
H	0.67365	-0.81094	2.97621
H	1.72265	0.26863	2.01229
C	-1.70795	0.49060	1.78758
C	-2.98657	0.85744	1.40879
C	-3.53792	0.45024	0.17359
C	-2.81723	-0.33717	-0.71586
H	-1.29445	0.81694	2.73658
H	-3.60266	1.47545	2.05284
H	-3.21689	-0.66902	-1.66574
H	5.32669	1.71963	0.73829
C	4.42565	1.41007	0.21784
C	4.10965	0.06105	0.12342
H	4.75190	-0.70403	0.54850
C	2.93405	-0.33810	-0.51811
C	2.11248	0.61200	-1.13864
H	1.22806	0.29894	-1.68350
C	2.45678	1.95542	-1.06816
H	1.82687	2.69285	-1.55599
C	3.59845	2.35980	-0.37755
H	-6.40953	0.96204	-1.25161
C	-5.41360	0.51731	-1.26052
O	-4.80373	0.89275	-0.04960
H	-4.85717	0.89516	-2.12838
H	-5.50577	-0.57331	-1.34622
H	3.85224	3.41389	-0.32056

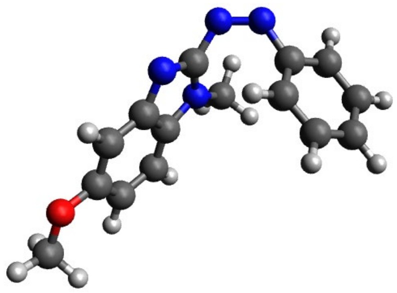

4-*Z*-**13a**

C	-1.41496	-0.89512	-0.53017
C	-1.01313	-0.38106	0.72401
N	-0.40547	-1.65926	-1.08091
C	0.57207	-1.57042	-0.21626
N	0.27696	-0.84048	0.91088
N	1.79013	-2.27587	-0.35825
N	2.88924	-1.68613	-0.35826
C	1.09812	-0.59553	2.06877
H	1.89906	-1.33423	2.11303
H	0.48727	-0.69440	2.97000
H	1.54172	0.40498	2.03510
C	-1.85105	0.40377	1.50236
C	-3.11678	0.68420	0.99634
C	-3.52716	0.18290	-0.25469
C	-2.68258	-0.60955	-1.02841
H	-1.54194	0.80224	2.46352
H	-3.78560	1.30365	1.58131
H	-3.01804	-0.99062	-1.98583
H	5.14803	2.02582	0.91435
C	4.31635	1.62180	0.34547
C	4.09211	0.25148	0.32547
H	4.73977	-0.43822	0.85779
C	3.00328	-0.26765	-0.38009
C	2.18311	0.58008	-1.13570
H	1.37102	0.17182	-1.72793
C	2.43837	1.94516	-1.13685
H	1.81053	2.60351	-1.72922
C	3.48859	2.47095	-0.38577
H	-5.27442	2.22128	0.12684
C	-5.65677	1.20714	-0.05092
O	-4.75254	0.42900	-0.79402
H	-6.56582	1.27120	-0.65057
H	-5.89761	0.74334	0.91494
H	3.67270	3.54095	-0.38677

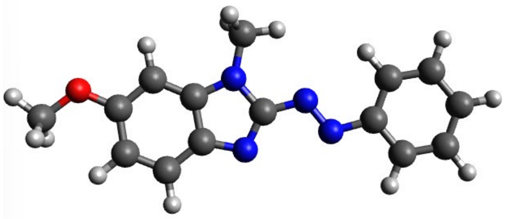

1-*E*-**18a**

C	1.73366	-0.95684	-0.00005
C	1.97032	0.44262	-0.00004
N	0.39187	-1.22367	-0.00016
C	-0.17878	-0.03913	-0.00006
N	0.72755	1.01071	-0.00008
N	-1.51803	0.30007	-0.00011
N	-2.30776	-0.67988	-0.00013
C	0.43706	2.42230	-0.00015
H	-0.64470	2.54631	-0.00038
H	0.85882	2.89824	-0.89095
H	0.85845	2.89827	0.89080
C	3.24414	0.99797	-0.00000
C	4.31250	0.10508	0.00002
C	4.10414	-1.29382	-0.00000
C	2.82651	-1.82803	-0.00004
H	3.43363	2.06542	-0.00003
O	5.54408	0.67592	0.00004
H	4.95431	-1.96514	-0.00002
H	2.66829	-2.90117	-0.00008
H	-6.61555	-2.02344	0.00009
C	-5.92825	-1.18308	0.00008
C	-4.55699	-1.40685	-0.00001
H	-4.13878	-2.40833	-0.00008
C	-3.66894	-0.32724	-0.00002
C	-4.16254	0.98530	0.00006
H	-3.45865	1.80997	0.00005
C	-5.53076	1.20116	0.00016
H	-5.91746	2.21615	0.00022
C	-6.41660	0.12045	0.00017
H	-7.48776	0.29946	0.00024
C	6.67055	-0.16879	0.00025
H	7.54029	0.48943	0.00032
H	6.70263	-0.80497	-0.89340
H	6.70233	-0.80470	0.89410

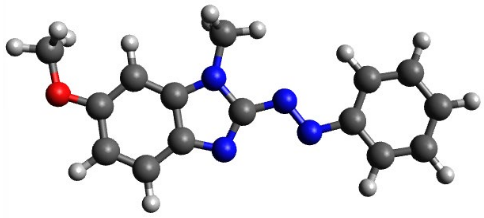

2-*E*-**18a**

C	1.71995	-1.19100	0.00005
C	2.03389	0.18925	-0.00004
N	0.36769	-1.38315	0.00015
C	-0.13899	-0.16840	0.00006
N	0.82715	0.82722	-0.00003
N	-1.45466	0.24665	0.00008
N	-2.30187	-0.68478	0.00017
C	0.61552	2.25264	0.00009
H	-0.45790	2.43547	-0.00079
H	1.06247	2.70513	-0.89076
H	1.06095	2.70486	0.89185
C	3.34074	0.68409	-0.00009
C	4.35093	-0.27080	-0.00007
C	4.06184	-1.65851	0.00002
C	2.76694	-2.12638	0.00007
H	3.54501	1.74767	-0.00013
O	5.67368	0.02495	-0.00013
H	4.90658	-2.33894	0.00006
H	2.54942	-3.18901	0.00017
H	-6.68327	-1.76539	-0.00035
C	-5.94654	-0.96800	-0.00022
C	-4.59132	-1.27436	-0.00010
H	-4.23469	-2.29940	-0.00013
C	-3.63923	-0.25069	0.00006
C	-4.05301	1.08916	0.00013
H	-3.30081	1.87000	0.00029
C	-5.40570	1.38751	0.00002
H	-5.73015	2.42411	0.00009
C	-6.35544	0.36260	-0.00016
H	-7.41382	0.60602	-0.00024
C	6.03738	1.38401	-0.00007
H	7.12761	1.40864	-0.00022
H	5.66466	1.90133	0.89378
H	5.66451	1.90149	-0.89378

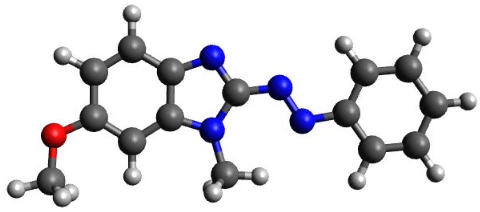

3-*E*-**18a**

C	-1.86656	1.37337	-0.00005
C	-1.96811	-0.03562	0.00004
N	-0.55974	1.75507	-0.00004
C	0.13318	0.63014	0.00004
N	-0.68156	-0.50124	0.00010
N	1.50720	0.71308	0.00006
N	2.15423	-0.36876	-0.00009
C	-0.35379	-1.91178	0.00025
H	0.72649	-2.02101	0.00064
H	-0.77565	-2.38883	-0.89058
H	-0.77633	-2.38877	0.89078
C	-3.18683	-0.71810	0.00006
C	-4.32799	0.07742	0.00000
C	-4.25024	1.49315	-0.00010
C	-3.03904	2.14641	-0.00012
H	-3.23284	-1.79993	0.00012
H	-2.97968	3.22947	-0.00019
H	6.27717	-2.23038	-0.00034
C	5.69395	-1.31464	-0.00020
C	4.30581	-1.37539	-0.00022
H	3.78044	-2.32549	-0.00038
C	3.55209	-0.19887	-0.00005
C	4.19501	1.04677	0.00015
H	3.58798	1.94547	0.00028
C	5.57949	1.09877	0.00016
H	6.08185	2.06167	0.00031
C	6.33268	-0.07781	-0.00001
H	-5.30523	-2.26665	-0.89393
C	-5.75002	-1.81039	-0.00004
O	-5.59044	-0.41187	-0.00002
H	-6.82476	-1.99460	-0.00001
H	-5.30518	-2.26670	0.89381
H	7.41746	-0.02657	-0.00001
H	-5.18691	2.03981	-0.00015

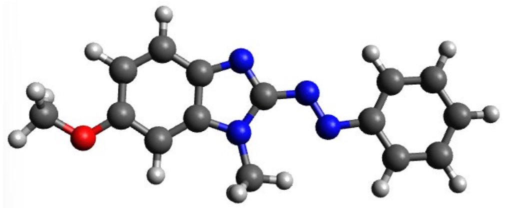

4-*E*-**18a**

C	1.86499	1.15468	-0.00001
C	1.89515	-0.26123	-0.00003
N	0.57681	1.60181	0.00000
C	-0.16906	0.51275	-0.00002
N	0.58461	-0.65963	-0.00005
N	-1.53861	0.66736	-0.00001
N	-2.23849	-0.38042	0.00001
C	0.18811	-2.05257	-0.00007
H	-0.89592	-2.11033	-0.00021
H	0.58787	-2.54915	0.89012
H	0.58811	-2.54917	-0.89015
C	3.07631	-0.99222	-0.00004
C	4.26190	-0.26013	-0.00004
C	4.25791	1.15462	-0.00001
C	3.06966	1.86421	0.00000
H	3.11324	-2.07555	-0.00005
H	3.06438	2.94899	0.00002
H	-6.44869	-2.03480	0.00008
C	-5.82060	-1.14927	0.00005
C	-4.43719	-1.27900	0.00005
H	-3.95950	-2.25391	0.00007
C	-3.62625	-0.14121	0.00002
C	-4.20613	1.13498	-0.00002
H	-3.55502	2.00226	-0.00004
C	-5.58627	1.25575	-0.00001
H	-6.04030	2.24234	-0.00003
C	-6.39683	0.11788	0.00002
H	6.75860	0.29535	-0.89387
C	6.63513	-0.32886	0.00009
O	5.39747	-1.00133	0.00003
H	7.39975	-1.10657	0.00022
H	6.75844	0.29553	0.89394
H	-7.47767	0.22296	0.00001
H	5.19575	1.69661	0.00001

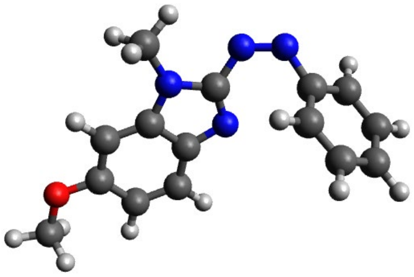

1-*Z*-**18a**

C	-0.89704	-0.35966	-0.47065
C	-1.59058	0.78254	0.00474
N	0.43632	-0.08497	-0.62761
C	0.55885	1.17296	-0.26884
N	-0.63846	1.76021	0.10362
N	1.64997	2.03354	-0.39559
N	2.83767	1.64814	-0.42886
C	-0.84338	3.12558	0.51868
H	0.05850	3.68964	0.28068
H	-1.03796	3.18520	1.59439
H	-1.69390	3.55071	-0.02146
C	-2.95343	0.78380	0.27494
C	-3.63563	-0.40813	0.04381
C	-2.96776	-1.55856	-0.43544
C	-1.60722	-1.54137	-0.69461
H	-3.49105	1.64983	0.64407
O	-4.96558	-0.37462	0.31238
H	-3.52317	-2.47332	-0.60322
H	-1.09502	-2.42509	-1.05980
H	5.36323	-2.03599	-1.26817
C	4.67040	-1.53868	-0.59607
C	4.15637	-0.29339	-0.93605
H	4.44190	0.20306	-1.85784
C	3.23652	0.32915	-0.09244
C	2.89350	-0.25787	1.12934
H	2.20887	0.25319	1.79882
C	3.44324	-1.48175	1.47865
H	3.18415	-1.93312	2.43177
C	4.31926	-2.13452	0.61200
H	4.73917	-3.09716	0.88721
C	-5.71927	-1.54398	0.09513
H	-6.74584	-1.29528	0.36698
H	-5.37321	-2.37406	0.72413
H	-5.69245	-1.85579	-0.95670

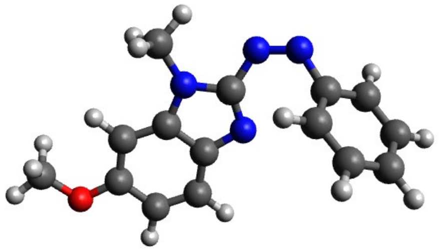

2-*Z*-**18a**

C	-0.83450	-0.58931	-0.47761
C	-1.65270	0.49025	-0.07013
N	0.46981	-0.19293	-0.58757
C	0.45929	1.08235	-0.26509
N	-0.80660	1.55738	0.03871
N	1.46154	2.04497	-0.36352
N	2.68364	1.78306	-0.38005
C	-1.16141	2.90698	0.40094
H	-0.30190	3.54611	0.19919
H	-1.42413	2.97307	1.46173
H	-2.01304	3.23903	-0.19970
C	-3.02863	0.37521	0.14520
C	-3.57475	-0.88378	-0.07703
C	-2.77516	-1.97832	-0.49114
C	-1.41963	-1.84627	-0.69412
H	-3.62580	1.22079	0.46333
O	-4.88840	-1.17797	0.07763
H	-3.27706	-2.92819	-0.64060
H	-0.80960	-2.68603	-1.00894
H	5.56883	-1.62133	-1.24510
C	4.82683	-1.20321	-0.57152
C	4.19177	-0.01216	-0.90169
H	4.43032	0.51999	-1.81693
C	3.21063	0.50649	-0.05682
C	2.92296	-0.12540	1.15680
H	2.18636	0.30643	1.82682
C	3.59103	-1.29168	1.49662
H	3.37362	-1.77737	2.44328
C	4.53211	-1.84416	0.62841
H	5.04493	-2.76296	0.89589
C	-5.74531	-0.13979	0.48752
H	-6.74333	-0.57455	0.55078
H	-5.75588	0.68440	-0.23771
H	-5.46002	0.25384	1.47194

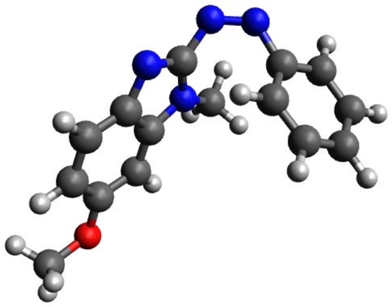

3-*Z*-**18a**

C	-1.32611	-1.30309	-0.60489
C	-1.11321	-0.40738	0.46782
N	-0.21748	-2.10410	-0.79316
C	0.64157	-1.67721	0.09298
N	0.16313	-0.67148	0.91204
N	1.90740	-2.27531	0.28026
N	2.96059	-1.60555	0.25556
C	0.80661	-0.03927	2.03599
H	1.62758	-0.66487	2.38768
H	0.08285	0.07020	2.84788
H	1.20302	0.94417	1.76367
C	-2.07113	0.49727	0.90287
C	-3.28451	0.50112	0.21511
C	-3.51857	-0.37712	-0.86242
C	-2.54680	-1.27905	-1.27560
H	-1.91501	1.19185	1.72059
H	-2.73090	-1.95816	-2.10132
H	4.83364	2.48975	0.72859
C	4.10468	1.88221	0.20123
C	3.95820	0.53981	0.52505
H	4.56792	0.07007	1.29062
C	2.99793	-0.23648	-0.12945
C	2.23759	0.31329	-1.16982
H	1.53048	-0.30228	-1.71583
C	2.41743	1.64772	-1.50935
H	1.83600	2.07377	-2.32113
C	3.33298	2.43862	-0.81652
H	-5.33293	1.73111	-1.05412
C	-5.44082	1.46640	0.00571
O	-4.19552	1.40707	0.65850
H	-5.98671	0.51729	0.08291
H	-6.01041	2.24700	0.51178
H	3.45745	3.48356	-1.08368
H	-4.46945	-0.35603	-1.38085

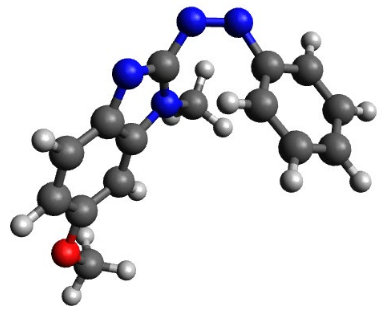

4-*Z*-**18a**

C	-1.34079	-1.50767	-0.50948
C	-1.20684	-0.41429	0.36922
N	-0.21417	-2.30107	-0.47052
C	0.58642	-1.68518	0.35891
N	0.04292	-0.54869	0.92901
N	1.84864	-2.19851	0.72614
N	2.88772	-1.51101	0.63957
C	0.60177	0.30486	1.94682
H	1.41305	-0.21568	2.45643
H	-0.17200	0.54089	2.68235
H	0.99367	1.23163	1.51562
C	-2.20416	0.54125	0.56889
C	-3.36926	0.37007	-0.17393
C	-3.52220	-0.71390	-1.06720
C	-2.52464	-1.65183	-1.24199
H	-2.06643	1.37353	1.24811
H	-2.64856	-2.48611	-1.92412
H	4.65441	2.65400	0.42791
C	3.97095	1.93603	-0.01485
C	3.82554	0.67731	0.55354
H	4.39238	0.38132	1.43084
C	2.92344	-0.23823	0.00470
C	2.22354	0.07982	-1.16670
H	1.56321	-0.64891	-1.62505
C	2.40320	1.32855	-1.74689
H	1.86931	1.57315	-2.66001
C	3.25861	2.26407	-1.16630
H	-4.22320	1.97821	1.80406
C	-4.35247	2.30430	0.76344
O	-4.43386	1.21028	-0.11603
H	-3.52771	2.97846	0.49618
H	-5.29705	2.84167	0.67160
H	3.38379	3.24092	-1.62334
H	-4.45839	-0.78125	-1.61082

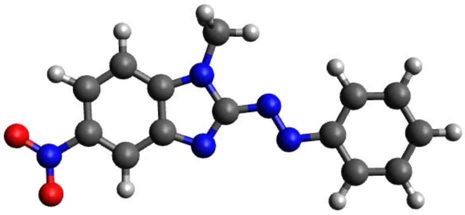

1-*E*-**23a**

C	-1.64006	-0.33335	0.00004
C	-1.68019	1.08627	-0.00002
N	-0.34722	-0.78200	0.00004
C	0.37755	0.31299	-0.00002
N	-0.37324	1.47683	-0.00006
N	1.75563	0.46508	-0.00005
N	2.38953	-0.62055	-0.00001
C	0.11405	2.83595	-0.00013
H	1.20221	2.80477	-0.00015
H	-0.23578	3.36417	0.89156
H	-0.23581	3.36409	-0.89187
C	-2.87443	1.81034	-0.00003
C	-4.04696	1.07893	0.00002
C	-4.00385	-0.32650	0.00009
C	-2.83061	-1.06063	0.00010
H	-2.89145	2.89495	-0.00008
H	-2.84894	-2.14300	0.00015
H	6.43219	-2.61750	0.00001
C	5.88319	-1.68121	-0.00003
C	4.49400	-1.68920	0.00000
H	3.92458	-2.61301	0.00005
C	3.78696	-0.48305	-0.00004
C	4.47648	0.73833	-0.00011
H	3.90982	1.66261	-0.00015
C	5.86099	0.73828	-0.00014
H	6.40198	1.67984	-0.00019
C	6.56651	-0.46853	-0.00009
H	7.65241	-0.45812	-0.00011
O	-6.30173	-0.39001	0.00013
O	-5.23088	-2.26996	0.00020
N	-5.27275	-1.04976	0.00014
H	-5.01588	1.56208	0.00002

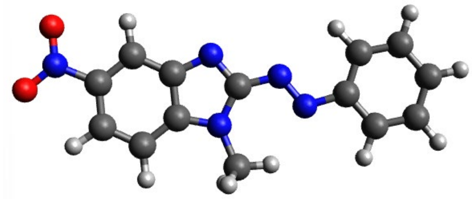

2-*E*-**23a**

N	5.44971	-0.71133	-0.00004
O	5.58963	-1.92413	0.00081
O	6.36859	0.09455	-0.00080
H	-0.04666	2.94408	-0.89040
C	-6.17592	0.67457	-0.00013
C	-6.57450	-0.65919	-0.00009
C	-4.82281	0.98933	-0.00009
H	-6.91809	1.46659	-0.00017
C	-5.61943	-1.67931	0.00002
C	-3.86816	-0.03097	-0.00002
C	-4.26849	-1.37452	0.00005
H	-4.48058	2.01952	-0.00012
N	-2.52616	0.39257	0.00000
C	-0.36982	2.39678	0.00035
H	-5.93764	-2.71741	0.00010
N	-1.68961	-0.54625	0.00000
H	-1.45036	2.29588	-0.00014
C	-0.35072	-0.19456	0.00001
N	0.22560	1.07364	0.00010
H	-3.50717	-2.14680	0.00014
C	1.57685	0.86776	0.00007
C	2.65103	1.76093	0.00011
N	0.53950	-1.16534	-0.00012
C	1.75206	-0.53807	-0.00007
C	3.91903	1.21063	0.00001
H	2.50884	2.83611	0.00032
C	3.03574	-1.08449	-0.00020
C	4.08742	-0.18542	-0.00012
H	4.80533	1.83274	0.00014
H	3.21252	-2.15254	-0.00036
H	-0.04743	2.94345	0.89176
H	-7.63151	-0.90887	-0.00033

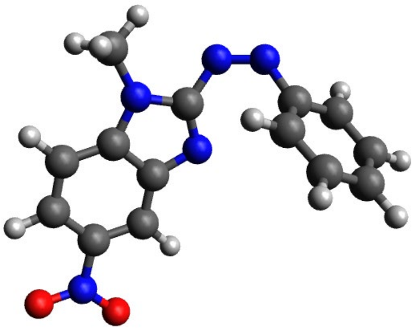

1-*Z*-**23a**

C	-0.91949	0.14218	-0.37697
C	-1.30006	1.41477	0.12371
N	0.41581	0.12636	-0.68958
C	0.83320	1.33462	-0.39496
N	-0.16114	2.16922	0.07779
N	2.06044	1.93601	-0.70186
N	3.14078	1.31370	-0.72932
C	-0.01852	3.55706	0.45016
H	0.92829	3.91814	0.04770
H	-0.02121	3.67548	1.53795
H	-0.84022	4.13701	0.02270
C	-2.59993	1.70407	0.54186
C	-3.53001	0.68605	0.43902
C	-3.14990	-0.57050	-0.06163
C	-1.86510	-0.87685	-0.47758
H	-2.87778	2.67831	0.92949
H	-1.61993	-1.86135	-0.85501
H	4.92768	-2.84668	-1.02021
C	4.34581	-2.14367	-0.43236
C	4.08057	-0.87729	-0.93721
H	4.45219	-0.56472	-1.90755
C	3.29614	0.00963	-0.19893
C	2.85226	-0.33683	1.08126
H	2.28890	0.37903	1.67163
C	3.15958	-1.58727	1.59459
H	2.82658	-1.85454	2.59276
C	3.88952	-2.50020	0.83387
H	4.11750	-3.48180	1.23750
N	-4.17004	-1.61258	-0.14352
O	-5.30103	-1.32925	0.22271
O	-3.83232	-2.70464	-0.57266
H	-4.56053	0.82856	0.73900

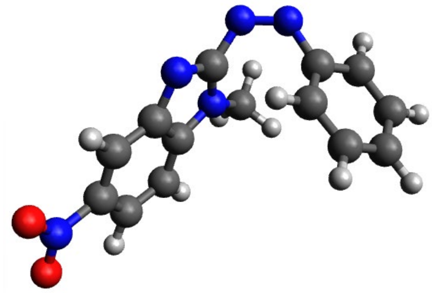

2-*Z*-**23a**

C	-1.23572	-0.85583	-0.41915
C	-0.77975	-0.41612	0.84720
N	-0.25459	-1.58257	-1.05967
C	0.75816	-1.54556	-0.23684
N	0.50779	-0.88330	0.94864
N	1.96302	-2.25528	-0.45163
N	3.06660	-1.67684	-0.48830
C	1.39179	-0.69790	2.07459
H	2.17296	-1.45820	2.05211
H	0.82306	-0.81444	2.99994
H	1.85788	0.29180	2.04974
C	-1.57166	0.32947	1.71913
C	-2.85058	0.64152	1.29066
C	-3.29629	0.20802	0.03359
C	-2.52235	-0.53837	-0.84255
H	-1.20972	0.66550	2.68491
H	-3.52664	1.22359	1.90407
H	-2.91300	-0.85206	-1.80218
H	5.39907	1.97199	0.82277
C	4.54287	1.59278	0.27380
C	4.31414	0.22494	0.20658
H	4.98110	-0.48752	0.68185
C	3.19307	-0.26103	-0.47223
C	2.34556	0.61749	-1.15957
H	1.51337	0.23526	-1.74084
C	2.60597	1.98099	-1.11606
H	1.95977	2.66456	-1.65779
C	3.68776	2.47249	-0.38695
O	-5.03141	0.19177	-1.47299
N	-4.65147	0.56700	-0.37484
O	-5.32499	1.22298	0.40616
H	3.87609	3.54109	-0.35251

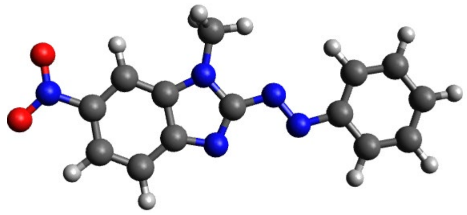

1-*E*-**26a**

C	-1.46164	-1.09227	-0.00014
C	-1.75535	0.29809	-0.00013
N	-0.11345	-1.29912	-0.00013
C	0.40473	-0.08730	-0.00020
N	-0.53589	0.92016	-0.00023
N	1.73475	0.30411	-0.00038
N	2.54821	-0.65463	-0.00039
C	-0.30407	2.34520	-0.00052
H	0.77141	2.51339	-0.00054
H	-0.74546	2.80036	0.89076
H	-0.74545	2.80001	-0.89199
C	-3.05007	0.79740	-0.00004
C	-4.05110	-0.16193	0.00008
C	-3.80687	-1.54646	0.00008
C	-2.51096	-2.02264	-0.00010
H	-3.30066	1.85098	0.00005
H	-4.65857	-2.21505	0.00017
H	-2.29726	-3.08551	-0.00003
H	6.87744	-1.91475	0.00025
C	6.17314	-1.08894	0.00023
C	4.80678	-1.33955	-0.00004
H	4.40752	-2.34864	-0.00023
C	3.89988	-0.27537	-0.00008
C	4.36524	1.04786	0.00015
H	3.64568	1.85880	0.00010
C	5.72841	1.28968	0.00041
H	6.09658	2.31124	0.00058
C	6.63387	0.22460	0.00046
H	7.70124	0.42461	0.00066
O	-5.63050	1.50653	0.00043
O	-6.31651	-0.54586	0.00023
N	-5.43483	0.29932	0.00027

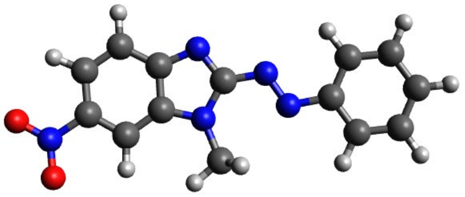

2-*E*-**26a**

N	5.29983	0.69941	0.00000
O	6.31362	0.01885	0.00001
O	5.28414	1.92212	-0.00000
H	-0.67355	2.08183	-0.88382
C	-5.90640	1.33387	-0.00003
C	-6.57483	0.11267	0.00000
C	-4.51751	1.35849	-0.00003
H	-6.46681	2.26340	-0.00005
C	-5.85443	-1.08489	0.00003
C	-3.79727	0.16089	-0.00001
C	-4.46964	-1.06944	0.00002
H	-3.96534	2.29305	-0.00006
N	-2.39781	0.29757	-0.00002
C	-0.07309	1.86472	0.00003
H	-6.38309	-2.03328	0.00005
N	-1.77046	-0.79376	0.00001
H	-0.67361	2.08179	0.88386
C	-0.39201	-0.68239	0.00000
N	0.35978	0.48127	0.00002
H	-3.88662	-1.98386	0.00004
C	1.67114	0.07154	0.00001
C	2.86639	0.77926	0.00001
N	0.33884	-1.78142	-0.00001
C	1.62879	-1.34685	-0.00001
C	4.01571	0.00495	-0.00000
H	2.94628	1.85923	0.00002
C	2.81985	-2.08756	-0.00002
C	4.01626	-1.40069	-0.00002
H	2.78612	-3.17119	-0.00003
H	4.96984	-1.91331	-0.00002
H	0.81625	2.49620	0.00007
H	-7.66048	0.08945	0.00000

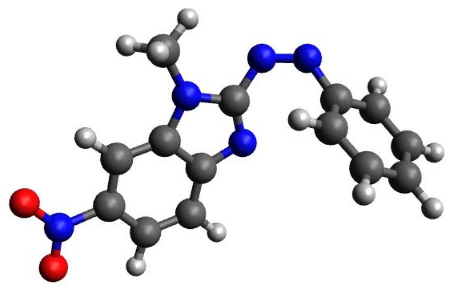

1-*Z*-**26a**

C	-0.66756	-0.38671	-0.68882
C	-1.34792	0.70494	-0.08678
N	0.65095	-0.08115	-0.88263
C	0.77382	1.14555	-0.42053
N	-0.39876	1.68693	0.04918
N	1.86305	2.01560	-0.55757
N	3.04975	1.63471	-0.52107
C	-0.58938	3.01610	0.57982
H	0.27302	3.61999	0.29587
H	-0.67810	2.99643	1.67021
H	-1.49595	3.45255	0.15352
C	-2.69325	0.66356	0.24655
C	-3.34118	-0.52389	-0.06183
C	-2.70894	-1.62492	-0.66275
C	-1.36629	-1.56499	-0.98266
H	-3.23462	1.47740	0.71288
H	-3.30189	-2.50891	-0.86133
H	-0.85506	-2.40206	-1.44447
H	5.72659	-2.00929	-0.88750
C	4.94832	-1.50292	-0.32515
C	4.46358	-0.27914	-0.76779
H	4.85267	0.19484	-1.66295
C	3.43370	0.34977	-0.06686
C	2.95085	-0.20076	1.12488
H	2.18995	0.32295	1.69513
C	3.47202	-1.40122	1.58205
H	3.10856	-1.82436	2.51343
C	4.45681	-2.06447	0.85054
H	4.85393	-3.00904	1.20912
N	-4.75943	-0.62854	0.26236
O	-5.29420	0.33863	0.78583
O	-5.32902	-1.67472	-0.00708

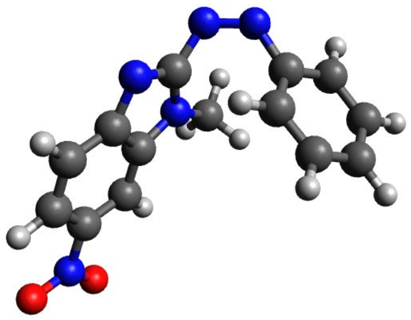

2-*Z*-**26a**

C	-1.06175	-1.50185	-0.54811
C	-0.97097	-0.49595	0.44555
N	0.10245	-2.22915	-0.60131
C	0.87698	-1.66232	0.29130
N	0.29151	-0.63014	0.98328
N	2.14978	-2.16693	0.64296
N	3.18120	-1.47333	0.55199
C	0.85288	0.17703	2.04001
H	1.67326	-0.36312	2.51345
H	0.08372	0.36671	2.79201
H	1.22938	1.12775	1.65036
C	-2.00397	0.38237	0.72639
C	-3.14812	0.21536	-0.04364
C	-3.27954	-0.76541	-1.03745
C	-2.23494	-1.63417	-1.29807
H	-1.95981	1.16386	1.47483
H	-4.21219	-0.81797	-1.58507
H	-2.31360	-2.40197	-2.05947
H	4.83123	2.73867	0.47443
C	4.16603	2.01721	0.01059
C	4.07140	0.73142	0.52597
H	4.65915	0.41701	1.38268
C	3.18890	-0.18624	-0.05081
C	2.46319	0.15443	-1.19958
H	1.82876	-0.57894	-1.68572
C	2.59420	1.43208	-1.72776
H	2.04353	1.69743	-2.62476
C	3.42610	2.36902	-1.11647
O	-4.13021	1.97185	1.06299
N	-4.26643	1.11868	0.19697
O	-5.27332	0.97200	-0.47862
H	3.51291	3.36800	-1.53241

Time-Dependent DFT

Table S4. TD-DFT excitation energies in nm and corresponding oscillator strengths for the n-π* and π -π* transitions of *E*-arylazobenzimidazoles calculated at the PBE0/6-31G(d,p) level of theory in the gas phase. The lowest energy conformers are highlighted.

compound	conformation	n-π* λ_{max}/ nm	f	π-π* λ_{max}/ nm	f
**3a**	1	484	0	370	0.6831
	**2**	**454**	**0**	**364**	**0.5375**
**3pz**	1	459	0	356	0.7267
	2	481	0	358	0.6918
	3	454	0	353	0.503
	**4**	**433**	**0**	**352**	**0.5068**
**8a**	**1**	**488**	**0**	**363**	**0.5747**
	2	485	0	363	0.8514
**8e**	**1**	**514**	**0**	**400**	**0.8496**
	2	509	0	395	1.059
**8pz**	1	462	0	350	0.5216
	2	485	0	353	0.5703
	3	485	0	353	0.8003
	**4**	**462**	**0**	**351**	**0.7990**
**13a**	**1**	**486**	**0**	**372**	**0.6021**
	2	489	0	374	0.8595
	3	485	0	360	0.7894
	4	487	0	363	0.9612
**18a**	1	488	0	419	0.5883
	**2**	**485**	**0**	**406**	**0.8194**
	3	480	0	393	1.0179
	4	483	0	401	0.8207
**23a**	**1**	**495**	**0**	**366**	**0.8568**
	2	491	0	359	1.0233
**26a**	**1**	**499**	**0**	**371**	**0.8648**
	2	489	0	362	0.6316

Table S5. TD-DFT excitation energies in nm and corresponding oscillator strengths for the n-π* and π -π* transitions of *Z*-arylazobenzimidazoles calculated at the PBE0/6-31G(d,p) level of theory in the gas phase. The lowest energy conformers are highlighted.

compound	conformation	n-π* λ_{max}/ nm	f	π-π* λ_{max}/ nm	f
**3a**	**1**	**508**	**0.0667**	**325**	**0.2168**
	2	481	0.0467	323	0.3610
**3pz**	1	510	0.0014	333	0.7739
	**2**	**532**	**0.0014**	**351**	**0.6129**
	3	486	0.0809	332	0.1338
	4	459	0.03	257	0.2684
**8a**	**1**	**509**	**0.0724**	**331**	**0.285**
	2	489	0.044	278	0.1131
**8e**	**1**	**496**	**0.0535**	**339**	**0.3885**
	2	522	0.0674	278	0.2921
**8pz**	**1**	**518**	**0.0043**	**338**	**0.7612**
	2	488	0.0810	338	0.1538
	3	464	0.0280	257	0.2686
**13a**	**1**	**511**	**0.0846**	**344**	**0.2255**
	2	511	0.0835	342	0.2596
	3	501	0.0648	279	0.1235
	4	493	0.0547	276	0.0965
**18a**	1	513	0.0936	328	0.2364
	**2**	**512**	**0.0928**	**367**	**0.2334**
	3	509	0.0707	278	0.159
	4	511	0.0742	278	0.1382
**23a**	**1**	**514**	**0.0747**	**310**	**0.1978**
	2	480	0.0373	280	0.2316
**26a**	**1**	**515**	**0.092**	**330**	**0.2186**
	2	479	0.042	308	0.2295

Table S6. TD-DFT excitation energies in nm and corresponding oscillator strengths for the n-π* and π -π* transitions of *E*-arylazobenzimidazoles calculated at the CAM-B3LYP/6-31+G(d,p) level of theory in the gas phase. The lowest energy conformers are highlighted.

compound	conformation	n-π* λ_{max}/ nm	f	π-π* λ_{max}/ nm	f
**3a**	1	452	0	348	0.9823
	**2**	**427**	**0**	**353**	**0.9619**
**3pz**	1	433	0	338	0.9672
	2	452	0	340	0.9503
	3	430	0	345	0.918
	**4**	**412**	**0**	**343**	**0.9213**
**8a**	**1**	**459**	**0**	**354**	**0.9485**
	2	455	0	349	1.0118
**8e**	**1**	**473**	**0**	**378**	**1.0411**
	2	469	0	370	1.134
**8pz**	1	440	0	343	0.9317
	2	459	0	346	0.9176
	3	458	0	341	0.9645
	**4**	**438**	**0**	**340**	**0.9734**
**13a**	**1**	**457**	**0**	**370**	**0.9434**
	2	460	0	365	0.7712
	3	455	0	374	0.7255
	4	458	0	335	0.6816
**18a**	1	458	0	380	0.8817
	**2**	**455**	**0**	**377**	**0.9771**
	3	450	0	370	1.0961
	4	453	0	369	1.1017
**23a**	**1**	**463**	**0**	**346**	**1.0013**
	2	459	0	340	1.0998
**26a**	**1**	**466**	**0**	**350**	**1.1499**
	2	455	0	347	1.1376

Table S7. TD-DFT excitation energies in nm and corresponding oscillator strengths for the n-π* and π -π* transitions of *Z*-arylazobenzimidazoles calculated at the CAM-B3LYP/6-31+G(d,p) level of theory in the gas phase. The lowest energy conformers are highlighted.

compound	conformation	n-π* λ_{max}/ nm	f	π-π* λ_{max}/ nm	f
**3a**	**1**	**489**	**0.0561**	**315**	**0.2253**
	2	466	0.0394	304	0.265
**3pz**	1	484	0.0018	318	0.846
	**2**	**506**	**0.0018**	**336**	**0.6814**
	3	471	0.0625	314	0.1798
	4	439	0.0212	249	0.2887
**8a**	**1**	**493**	**0.0606**	**317**	**0.2051**
	2	464	0.0317	262	0.2091
**8e**	**1**	**476**	**0.0439**	**320**	**0.2909**
	2	481	0.0462	272	0.2386
**8pz**	**1**	**496**	**0.0047**	**323**	**0.8164**
	2	472	0.0627	318	0.1419
	3	442	0.0198	252	0.3003
**13a**	**1**	**494**	**0.0693**	**330**	**0.336**
	2	495	0.0668	317	0.2989
	3	469	0.0408	264	0.1982
	4	464	0.0345	263	0.1921
**18a**	1	495	0.0734	338	0.276
	**2**	**494**	**0.0753**	**333**	**0.3723**
	3	473	0.0448	263	0.2219
	4	476	0.0477	262	0.2010
**23a**	**1**	**496**	**0.0606**	**313**	**0.184**
	2	458	0.0278	262	0.2514
**26a**	**1**	**496**	**0.0726**	**318**	**0.2995**
	2	457	0.0312	297	0.278

References

[1] Weston, C. E.; Richardson, R. D.; Haycock, P. R.; White, A. J.; Fuchter, M. J. Arylazopyrazoles: Azoheteroarene Photoswitches Offering Quantitative Isomerization and Long Thermal Half-Lives. *J. Am. Chem. Soc.* **2014**, *136* (34), 11878-11881.

[2] Heath, H. A New Sensitive Chemical Actinometer - Ii. Potassium Ferrioxalate as a Standard Chemical Actinometer. *Proceedings of the Royal Society of London. Series A. Mathematical and Physical Sciences* **1997**, *235* (1203), 518-536.

[3] Stranius, K.; Borjesson, K. Determining the Photoisomerization Quantum Yield of Photoswitchable Molecules in Solution and in the Solid State. *Sci. Rep.* **2017**, *7*, 41145.

[4] Fischer, E. Calculation of Photostationary States in Systems A .Dblarw. B When Only a Is Known. *The Journal of Physical Chemistry* **1967**, *71* (11), 3704-3706.

[5] Frisch, M. J.; Trucks, G. W.; Schlegel, H. B.; Scuseria, G. E.; Robb, M. A.; Cheeseman, J. R.; Scalmani, G.; Barone, V.; Petersson, G. A.; Nakatsuji, H.; Li, X.; Caricato, M.; Marenich, A. V.; Bloino, J.; Janesko, B. G.; Gomperts, R.; Mennucci, B.; Hratchian, H. P.; Ortiz, J. V.; Izmaylov, A. F.; Sonnenberg, J. L.; Williams; Ding, F.; Lipparini, F.; Egidi, F.; Goings, J.; Peng, B.; Petrone, A.; Henderson, T.; Ranasinghe, D.; Zakrzewski, V. G.; Gao, J.; Rega, N.; Zheng, G.; Liang, W.; Hada, M.; Ehara, M.; Toyota, K.; Fukuda, R.; Hasegawa, J.; Ishida, M.; Nakajima, T.; Honda, Y.; Kitao, O.; Nakai, H.; Vreven, T.; Throssell, K.; Montgomery Jr., J. A.; Peralta, J. E.; Ogliaro, F.; Bearpark, M. J.; Heyd, J. J.; Brothers, E. N.; Kudin, K. N.; Staroverov, V. N.; Keith, T. A.; Kobayashi, R.; Normand, J.; Raghavachari, K.; Rendell, A. P.; Burant, J. C.; Iyengar, S. S.; Tomasi, J.; Cossi, M.; Millam, J. M.; Klene, M.; Adamo, C.; Cammi, R.; Ochterski, J. W.; Martin, R. L.; Morokuma, K.; Farkas, O.; Foresman, J. B.; Fox, D. J. *Gaussian 16 Rev. C.01*, Wallingford, CT, 2016.

[6] Adamo, C.; Barone, V. Toward Reliable Density Functional Methods without Adjustable Parameters: The Pbe0 Model. *The Journal of Chemical Physics* **1999**, *110* (13), 6158-6170.

[7] Grimme, S.; Antony, J.; Ehrlich, S.; Krieg, H. A Consistent and Accurate Ab Initio Parametrization of Density Functional Dispersion Correction (Dft-D) for the 94 Elements H-Pu. *J. Chem. Phys.* **2010**, *132* (15).

[8] Francl, M. M.; Pietro, W. J.; Hehre, W. J.; Binkley, J. S.; Gordon, M. S.; DeFrees, D. J.; Pople, J. A. Self-Consistent Molecular Orbital Methods. Xxiii. A Polarization-Type Basis Set for Second-Row Elements. *J. Chem. Phys.* **1982**, *77* (7), 3654-3665.

[9] Yanai, T.; Tew, D. P.; Handy, N. C. A New Hybrid Exchange–Correlation Functional Using the Coulomb-Attenuating Method (Cam-B3lyp). *Chem. Phys. Lett.* **2004**, *393* (1-3), 51-57.
